# An open-source, wireless vest for measuring autonomic function in infants

**DOI:** 10.3758/s13428-020-01394-4

**Published:** 2020-04-24

**Authors:** Charles Maitha, Jesse C. Goode, Danielle P. Maulucci, Suha M. S. Lasassmeh, Chen Yu, Linda B. Smith, Jeremy I. Borjon

**Affiliations:** 1 Department of Psychological and Brain Sciences, Indiana University, 1101 E. 10th St., Bloomington, IN 47405 USA; 2 School of Psychology, University of East Anglia, Norwich, UK

**Keywords:** Autonomic nervous system, Cardiorespiratory activity, Movement, Sustained attention

## Abstract

Infant behavior, like all behavior, is the aggregate product of many nested processes operating and interacting over multiple time scales; the result of a tangle of inter-related causes and effects. Efforts in identifying the mechanisms supporting infant behavior require the development and advancement of new technologies that can accurately and densely capture behavior’s multiple branches. The present study describes an open-source, wireless autonomic vest specifically designed for use in infants 8–24 months of age in order to measure cardiac activity, respiration, and movement. The schematics of the vest, instructions for its construction, and a suite of software designed for its use are made freely available. While the use of such autonomic measures has many applications across the field of developmental psychology, the present article will present evidence for the validity of the vest in three ways: (1) by demonstrating known clinical landmarks of a heartbeat, (2) by demonstrating an infant in a period of sustained attention, a well-documented behavior in the developmental psychology literature, and (3) relating changes in accelerometer output to infant behavior.

**Electronic supplementary material:**

The online version of this article (10.3758/s13428-020-01394-4) contains supplementary material, which is available to authorized users.

## Introduction

Behavior is multi-causal. It is the aggregate product of many nested processes operating and interacting over multiple time scales (Byrge, Sporns, & Smith, 2014; Smith & Thelen, 2003). Such complexity gives rise to a tangle of inter-related causes and effects. For example, a child’s ability to visually sustain their attention to objects is not just about looking. It is a complex state that includes holding objects and body stillness (Yu & Smith, 2012), likely inducing changes in heart rate and breathing (Lansink, Mintz, & Richards, 2000). Thus, efforts in identifying the mechanisms supporting infant behavior require the development and advancement of new technologies that can accurately and densely capture its multiple branches.

The development of a wireless autonomic vest for use in infants 8–24 months of age is described below. The vest wirelessly captures cardiac activity, respiration, and movement in a non-invasive manner. This is a broadly applicable method to the field of child development and behavioral research in general. This publication details how to construct and use the vest, so that other researchers across the field of behavioral science can benefit from it. The present article will present proof for the vest’s utility by demonstrating clinically relevant landmarks in the cardiac signal as well as an instance of an infant in a state of sustained attention (Ruff & Lawson, 1990; Ruff & Rothbart, 1996), a well-documented behavior in the developmental psychology literature known to be a predictor of later cognitive achievements (G. J. Duncan et al., 2007; Kannass, Oakes, & Shaddy, 2006; McClelland & Cameron, 2019; Welsh, Nix, Blair, Bierman, & Nelson, 2010). The authors also provide a six-minute video of a subject interacting with their caregiver to relate changes in accelerometer output to the child’s behavior.

### Why measure autonomic activity in infants?

The autonomic nervous system (ANS) is critical for maintaining homeostatic control in the mammalian body (Loewy & Spyer, 1990). The ANS pathways represent one of three general motor outputs of the central nervous system; the other two outputs are the somatic-motor pathways and the neuroendocrine output of the pituitary (Gibbins, 2013; Langley, 1921; Sharkey & Pittman, 1996). These pathways have been traditionally divided into two functional systems: sympathetic and parasympathetic (Cannon, 1932). Recent molecular physiology evidence, however, has cast doubt on the tenability of such a clean division between these overlapping systems (Ernsberger & Rohrer, 2018; Gibbins, 2013; Saper, 2002). The ANS is broadly responsible for the maintenance of cardiovascular, thermal, and gastrointestinal homeostasis, with dynamic patterns of activity in the ANS underlying many behaviors, from the expression of emotion to motor movements (Berntson, Cacioppo, & Quigley, 1993; Pfaff, 2006; Saper, 2002; Sharkey & Pittman, 1996).

Autonomic function can be measured in many ways. Cardiac activity, respiratory rate, pupil dilation, galvanic skin response, and movement patterns are a subset of these indices (Pfaff, 2006). Some of these components have a reliable and predictable relation to one another (Morin & Viala, 2002; Obrist, Light, McCubbin, Hutcheson, & Hoffer, 1979; Potts, 2002), such as the yoked relation between increased motor activity and increased heart rate (Obrist, Webb, Sutterer, & Howard, 1970). Other indices are less directly related, possessing different time constants, such as the relationship between galvanic skin response and heart rate (Tursky, Schwartz, & Crider, 1970), or are uncorrelated in a natural setting (Lacey, 1967). The influence of autonomic activity on behavior, and vice versa, can therefore be best understood through the simultaneous measurement of its multiple branches (Ghazanfar, Takahashi, Zhang, & Borjon, 2017; Ghazanfar & Zhang, 2016; Pfaff, 2006). For example, the nonhuman primate literature has provided a clear demonstration of the utility in measuring the autonomic nervous system’s multiple branches. The production of vocalizations in marmoset monkeys is tied to rhythmic changes in cardiac activity and breathing (Borjon, Takahashi, Cervantes, & Ghazanfar, 2016) which is then modulated by the presence of social partners (Liao, Zhang, Cai, & Ghazanfar, 2018). The autonomic activity of marmoset monkeys is genetically inherited (Zhang & Ghazanfar, 2016), and the development of vocal production occurs in tandem with autonomic development (Gustison, Borjon, Takahashi, & Ghazanfar, 2019).

Together, changes in cardiac activity, breathing, and movement are often considered to be indicative of the autonomic arousal state of an animal (Pfaff, 2006). Functionally, a high arousal state indicates an animal is more alert to sensory stimuli, more motorically active, and more reactive (Garey et al., 2003). There is a large overlap in neural structures considered to underlie social and cognitive behavior as well as those underlying motivation and arousal (Cardinal, Parkinson, Hall, & Everitt, 2002; Syal & Finlay, 2011). In humans, changes in arousal influence cognitive behaviors such as language production (Kleinow & Smith, 2006) and can be reflected in the acoustic features of vocalizations such as prosody (Wiethoff et al., 2008) and fundamental frequency (McRoberts, Studdert-Kennedy, & Shankweiler, 1995). Changes in cardiorespiratory activity have been documented during attention (Lansink & Richards, 1997; Wass, Clackson, & de Barbaro, 2016), motor planning (Jennings & van der Molen, 2002; Jennings, van der Molen, Brock, & Somsen, 1991), and language production (Peters & Hulstijn, 1984). For this reason, the autonomic vest described below was designed to simultaneously capture cardiac activity, respiration, and movement patterns in a dense and accurate manner.

In infant research, ANS measurement has been in the service of understanding a variety of processes, from clinical disorders to social, emotional, and cognitive behaviors. Autonomic dysfunction has been reported in clinical populations such as children with attention deficit disorder (Imeraj et al., 2011, 2012), autism spectrum disorders (Bal et al., 2010; Heilman, Harden, Zageris, Berry-Kravis, & Porges, 2011), and implicated in sudden infant death syndrome (J. R. Duncan, 2018). Differences in autonomic profiles have been used to predict a child’s temperament: fearful children exhibit a lower variability in their heart rate than less inhibited children (Fox, 1989; Kagan, Reznick, & Snidman, 1987; Stifter & Jain, 1996) and are also indicative of later neuropsychiatric outcomes (Mulkey & du Plessis, 2019). The ANS is also responsive to the social environment: physiological synchrony between an infant and their caregiver has been commonly reported during many forms of interactions (Feldman, Magori-Cohen, Galili, Singer, & Louzoun, 2011; Ham & Tronick, 2006; Moriceau & Sullivan, 2005; Palumbo et al., 2016; Van Puyvelde et al., 2015). Further, as infants have a limited behavioral repertoire, indirect measurements of cognition are useful in understanding the development of cognitive processes such as attention (de Barbaro, Clackson, & Wass, 2017; Wass et al., 2016). Thus, the ability to wirelessly measure cardiac activity, respiration, and movement patterns would serve a large subset of psychological and neuroscientific research questions.

The authors will demonstrate the utility, and provide evidence for the validity, of the autonomic vest described below by measuring three processes. First, the cardiac data collected will be analyzed to ensure the measured heartbeats exhibit known physiological markers, such as the QRS complex as well as the R and P waves. Second, the authors will demonstrate a period of sustained attention in an infant, a well-documented behavior in developmental psychology. Individual differences in the frequency and duration of sustained attention predicts much later individual differences in not just visual attention (Lansink & Richards, 1997; Richards & Casey, 1992; Ruff, 1986), but also self-control (Kochanska, Murray, & Harlan, 2000; Reck & Hund, 2011; Ruff, 1986), language (Welsh et al., 2010), and later school achievement (G. J. Duncan et al., 2007; Kannass et al., 2006; McClelland & Cameron, 2019). For infants, sustained visual attention to an object is not just about looking. It is a more complex state that includes holding objects and body stillness (Yu & Smith, 2012) which likely induces changes in heart rate and breathing (Lacey, 1972; Norman & Shallice, 1986; Porges & Raskin, 1969; Richards, 1985, 1987). Finally, changes in movement will be related to a six-minute video of naturalistic play provided by the authors (Video 1).

### Why design a vest for use in developmental populations?

Developmental populations present two challenges in the study of autonomic activity: flexibility and accuracy. First, the surface electrodes commonly used in most measures of autonomic function require the application of conductive gel and are placed across the body, from the chest to the hip or feet. These surface electrodes tend to be wired and connected to a data acquisition system, making the participant effectively tethered. Experimental designs that require freely moving and unrestrained activity then become unfeasible. Even when wireless solutions are available, the wired electrodes are generally attached to a large transponder that has to be mounted to the infant’s body or hidden in clothing. Second, it is not uncommon for proprietary solutions to have the raw, cardiorespiratory data hidden behind proprietary algorithms which only release a derived heart rate or respiration rate value. Such algorithms make it difficult to verify the recorded measures. It is important to note, however, that reliable open-source analysis programs for analyzing raw echocardiogram data are available (Thorson, West, & Mendes, 2018; Vest et al., 2018).

Wireless devices are commonly used in adults to measure autonomic function, yet it is difficult to find comparable devices in children. Existing solutions appropriate to developmental populations include wristbands and traditional lead setups. For example, the Empatica E4 is a wireless wristband that can be easily worn by developmental populations. The device records blood volume pulse, movement with a three-axis accelerometer, galvanic skin responses, and peripheral skin temperature. Its lightweight and portable design allows for recordings of up to 24 hours in an unobtrusive fashion, and an event mark button provides the ability to link physiological signals to experimental events. While the Empatica E4 is an excellent option for many studies, it measures heart rate variability and the inter-beat interval indirectly using a photoplethysmograph, which is very sensitive to noise caused by motion. Inter-beat intervals are calculated using a proprietary algorithm, which makes manual correction and verification of data difficult. Devices produced by Biopac, a leader in physiology measurement tools, offer an alternative to a wristband. Their wireless solutions for measuring autonomic function include the Bioharness and Bionomadix Bioshirt, which are similar in principle to the vest described below. These devices, however, are designed for use in adults and are unable to be adjusted to accommodate developmental populations without expensive customizations. Traditional electrode lead designs are available; however, they require the application of electrode gel and are generally tethered to a data acquisition system. While an ultra-portable wireless solution such as the Empatica E4 provides convenience, it does not easily lend itself to data verification. Traditional wired setups are accurate and reliable; however, they are not optimized for use with freely moving children.

The autonomic vest described below represents several technological advantages and alternative solutions to currently available devices. The vest is lightweight and portable, making it easy to use in various experimental settings. The use of four-way-stretch material and straps make the vest accommodating to children between the ages of 8 and 24 months. Cardiac activity is measured using conductive cloth electrodes which do not require electrode gel or water. Respiration is measured using a force sensitive resistor, which allows the vest to be resized. Motion is measured using a three-axis accelerometer embedded in the chest. When applied correctly, and securely, the vest is also resistant to noise caused by motion. The raw data are minimally processed, allowing for an accurate and temporally precise calculation of autonomic function. Thus, the vest is optimally designed to be used in freely moving studies of infant behavior.

## Methods

### Overview

**Fig. 1:**
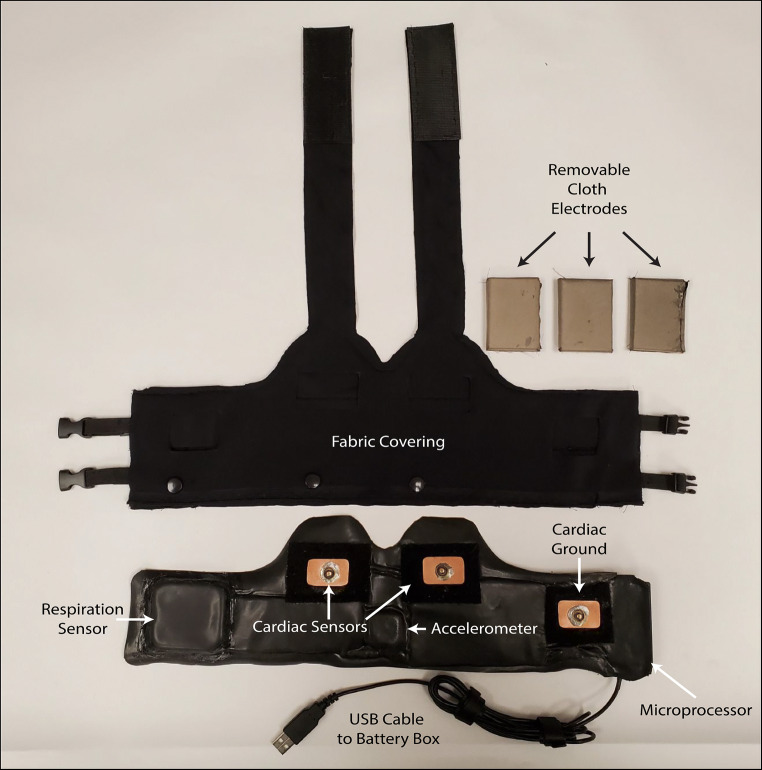
*The autonomic vest*. The vest comprises two components. The sensors are mounted on a rubber mat (bottom) with a fabric covering equipped with snap buttons to secure the sensors (top). Arrows indicate the position of the cardiac sensors, cardiac ground, respiration sensor, accelerometer, and the microprocessor

A labeled image of the completed vest can be seen in Fig. 1. The autonomic vest comprises two components: (1) the electronics (Supplementary Material 1), which are mounted between two rubber sheets, and (2) the fabric covering (Supplementary Material 2), which covers the electronics and is tied around the infant. The mounted electronics weigh 173 g, and there is an external battery box that weighs 98 g. Without the electronics, the fabric cover weighs 70 g, and the detachable electrodes weigh 60 g. Therefore, without the battery box, the completed vest weighs 303 g and is 40.64 cm long and 12.1 cm wide. A set of buckles gives an extra 9.53 cm for size adjustment, and cloth straps allow it to be secured safely around the neck of the subject. The fabric covering is made of a four-way stretch material comprising nylon and spandex. Cardiac activity is recorded using three removable and replaceable patches of conductive cloth: two on the ventral side of the chest and one on the dorsal to serve as a ground. Trunk motion and movement is measured using a three-axis accelerometer located in the center of the chest. Respiration is recorded using a force sensitive resistor. Data is both written locally to an SD card and wirelessly streamed from the vest to a computer using Bluetooth. An in-house MATLAB algorithm starts the recording of data on the SD card and stores a backup version of the data on the computer running the software. The instructions for constructing the vest will be summarized and discussed below. Thorough, step-by-step instructions with pictures can be found in the supplementary materials to this article. The relevant suite of software and associated files can be found in the Open Science Framework (osf.io/24gp5). A full table of the materials and sensors needed for this vest, as well as their respective cost, can be found in Table 1. At the time of publication, the authors estimate the cost of construction for a single vest to be approximately $285 USD.

**Table 1.** Electronics and materials. The above table describes the list of materials and electronics necessary for completion of the vest. Prices were calculated at time of publication and may be outdated

CATEGORY	ITEM	NOTES	PRICE
Electronics	Teensy 3.6	DigiKey #1568-1442-ND	$32.50
Electronics	SparkFun AD8232 single lead heart rate monitor		$19.95
Electronics	Accelerometer LISD3H	DigiKey #1528-1516-ND	$4.95
Electronics	Bluetooth module HC-05	Cost for 2	$8.88
Electronics	3.3 V Battery charger	DigiKey #1528-1833-ND	$6.95
Electronics	LiPO battery 2500 mAH	DigiKey #1528-1840-ND	$6.89
Electronics	Switch slide SPDT	DigiKey #679-3535-ND	$1.37
Electronics	Cobaltex near-field magnetic and electric shielding fabric	Conductive cloth for electrodes	$13.99
Electronics	Electronics Force-sensitive resistor	DigiKey #FSR03CE-ND	$13.97
Electronics	Flexible stranded electrical wire 16 AWG	10 ft (5 ft black and 5 ft red)	$5.99
		**Electronics total**	**$115.44**
Materials	Lycra matte milliskin nylon spandex fabric four-way stretch	58 inches wide, black	$8.99
Materials	Coats & Clark dual duty all-purpose thread	1 spool of yarn, black	$4.30
Materials	Singer 07051 pearlized head straight pins	Size 24, 120-count, white	$4.70
Materials	Schmetz universal (130/705 H) sewing machine needles	Size 90/14	$6.19
Materials	Dritz notions 1 ½-inch black elastic soft waistband		$7.02
Materials	Dritz 9332B braided elastic	½ inch × 1 ½ yard, black	$4.42
Materials	Velcro brand one wrap thin ties	8 × ½ inches, black	$10.00
Materials	Dritz 495 parachute buckle	for ½-inch strap, black	$5.04
Materials	Aleene's platinum bond adhesive super fabric	2 oz.	$7.51
Materials	Ruspepa 12-inch ×15-inch silicone pad	¼-inch rubber silicone sheet	$18.99
Materials	1 ½-inch Velcro band	45-foot roll	$16.97
Materials	½-inch Velcro 1801-OW-PB/B	10-foot roll	$6.20
Materials	1/32-inch-thick rubber sheet	McMaster-Carr #1290N52	$19.75
Materials	12.5-mm metal button snaps kit		$14.90
Materials	Craftown snaps all-in-one starter kit		$14.99
Materials	Arteza 45-mm quilting rotary cutter replacement blades		$14.43
Materials	Rubber cement		$5.97
		**Materials total**	**$170.37**
		**FINAL TOTAL**	**$285.81**

### Electronics

**Fig. 2:**
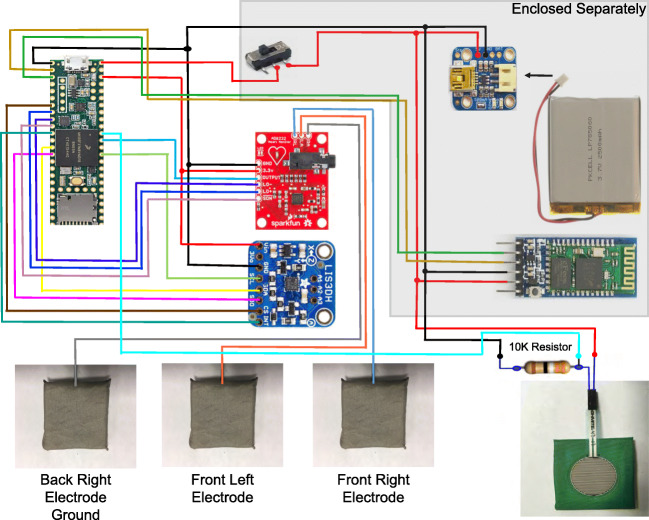
*Wiring diagram for the autonomic vest.* A diagram listing all the connections between the electronic components of the vest. Colored lines indicate soldered connections between the components. Components in the top right of the figure are enclosed separately

To capture changes in cardiac activity, respiration, and movement, the autonomic vest utilizes a suite of sensors that are easily acquired and connected for use. A full list of the sensors needed can be found in Table 1. The wiring diagram for the vest can be seen in Fig. 2. In-depth instructions for the creation and soldering of the sensors, as well as information for programming the necessary components, can be found in Supplementary Material 1. The software necessary for flashing the electronic components and the .stl files for 3D printing the containers of the electronics can be found in the Open Science Framework (osf.io/24gp5). The sensors chosen for data acquisition and the detection of cardiac activity, respiratory activity, and movement are detailed below. To protect the electronics from any messes made by the child subject, all sensors are encased in two rubber mats which are sealed with rubber cement.

### Data acquisition

#### Microprocessor - Kinetis ARM^®^ Cortex^®^-M4 microprocessor (K66 Family) /Teensy 3.6

For use in developmental populations, the microprocessor must be small in size with a low profile. Processors must also incorporate enough ports to accomplish recording from multiple sensors and allow for ease of programming through the integrated development environment (IDE) or a development toolchain. One of the more readily available IDE devices is Arduino, which is free with a wide range of already developed application programming interfaces (APIs) for its users. Using the Arduino IDE will ensure that most researchers can program the microprocessor used in this vest.

The most convenient microprocessor is the Kinetis ARM^®^ Cortex^®^-M4 (K66 Family), which is also available in an evaluation board, Teensy 3.6. Apart from having a powerful microprocessor and a wide range of peripherals like analog-to-digital converters (ADCs) and assorted communication buses, this evaluation board comes with an SD card module embedded in it. Evaluation boards and breakout boards provide the easy connection of sensors or external peripherals to the microprocessor and provide well laid-out circuitry for powering the chip and its ports. Without the ports and laid-out circuitry, high-tech soldering and custom circuit design would be required.

Another major goal for the vest is the ability to sample data at a constant sampling rate of 1000 Hz. Such a sampling rate ensures that the fast dynamic of the heartbeat wave form can be captured, allowing for accurate analyses. All devices related to this vest must therefore have a throughput that is at least twice the targeted sample frequency. The issue of throughput versus the number of ports needed is not a limitation when using Teensy 3.6 since its 57 ports which are considerably more than needed for this project.

The inclusion of an SD card module makes data acquisition more convenient and accurate. Data is collected from the peripheral sensors, processed, and written locally to the SD card. This presents no opportunities for drops in data and minimizes the opportunity for delays in writing the data. However, it is still important to have a way to visualize the data being collected in order to determine whether the signals are capturing verifiable cardiorespiratory and movement data. This is accomplished using a Bluetooth module.

#### Bluetooth module - BLE v5.0

To facilitate data visualization, a Bluetooth module was incorporated into the vest. Bluetooth communication is used to transfer live data to the graphical user interface (GUI) display for monitoring and backup data collection.

The Bluetooth Low Energy module version 5.0 (BLE v5.0) is capable of being configured to be either the master or slave in communication. It easily enables a choice of private communication where it can only pair with a specified modular Bluetooth device. This private communication ability will ensure that not every Bluetooth device in the vicinity can pair with the vest. This Bluetooth module is readily available on evaluation boards and can easily be purchased from Amazon. BLE v5.0 supports 2.4 GHz communication, at 2.1 Mbps (Max) asynchronously and 1 Mbps/1 Mbps synchronously. Its ability to transfer transistor-transistor logic (TTL) signals or convert received messages into TTL signals makes it easy to use with any microprocessor having universal asynchronous receiver/transmitter (UART) capability.

#### Battery and charger - 2500 mAH

The power consumption of the vest is low, with an approximate maximum of 0.99 W. Assuming an extreme case where the power consumption is 0.99 W at 3.3 V, the current drawn would be 300 mA. Thus, the battery chosen for this vest must be able to supply the vest with a maximum of 3.3 V at 300 mA for the entire duration of the experiment. A 2500 mAH-3.3 V battery is suitable for this purpose. Given the anticipated demands, this battery can last at most eight hours at maximum power. This time period is sufficient for multiple short experiments or long at-home recordings.

The battery charger was chosen based on its safety, size, voltage, and current output. The charger for the vest battery is small in size (less than 900 mm^2^) and rated 100 mA at default and 500 mA with minor modification. The charger is used at a default setting: 3.3 V output at 100 mA.

### Cardiac activity

#### Heart rate monitor - AD8232 Single Lead Heart Rate Monitor

The heart rate monitor is located behind the respiratory sensor, on the back left side of the vest. It must be able to collect accurate data with little to no electrical noise. It must also be easy to use and able to withstand any electrostatic discharge (ESD) that may arise when wearing the vest. The ability to detect a leads-off situation will enable reliable data collection. The AD8232 Single Lead Heart Rate Monitor is a complete analog front-end (AFE) device, meaning it does not require other devices for amplification, signal conditioning, or filtering. This chip is manufactured by Analog Devices and its evaluation board is readily available.

#### Cardiac sensor – conductive cloth electrodes

The cardiac electrodes are located on the front of the vest, in line with the child’s heart. The ground is located on the back right side of the child’s chest, just past the armpit. Heartbeats are very low-voltage electrical signals in the range of millivolts. One challenge in detecting such a low-voltage signal is avoiding voltage loss due to the resistance of leads. Any resistance in the circuitry will result in a voltage drop across it. To avoid weakening the cardiac signals, the electrodes must have very low resistance. Contemporary electrodes use gel for enhancing electrical conductivity. For developmental populations, the use of gel is commonplace, but may result in discomfort as well as increased preparation time. Commercially available conductive cloth is designed for electromagnetic interference (EMI) protection and is produced in a variety of resistance per square area. For the vest, frequency attenuation at the electrodes is not a goal or concern, but electrical conductivity is. The targeted total resistance from the electrode to the connection point on the heart rate monitor should be below 0.5 Ω. Conductive cloth does not have an even distribution of resistance, thus the units for sheet resistance are measured in ohms/square, with a lower value indicating a more conductive material. More conductive material helps to offset the resistance due to connecting the electrode wires to the electrode. Any conductive cloth with less than 0.1 Ω/square sheet resistance will work for this vest. However, please note that some of the best conducting cloths lack a silky or soft feel and may not be suitable for constant skin contact in developmental populations. The cardiac electrodes will detect voltages as low as 0.0081 mV. As this is a highly sensitive electrode, a reference ground is used to subtract ambient noise. In our tests, ambient noise tends to be between 0 and 0.4 mV, and the average amplitude of a recorded heartbeat is around 1 mV, with an upper limit of around 2.5–3.0 mV.

### Respiratory activity

#### Respiratory sensor - Ohmite FSR03CE

The respiratory sensor is located on the back left side of the vest, parallel to the cardiac ground. One challenge is to detect respiration while ignoring heartbeats and other disturbances created by body movement. Publicly available devices typically record respiration with stretch sensors. Stretch sensors present two hurdles for developmental populations: they prevent a vest from being resized, and they may require a subject to breathe hard in order to detect a signal. To solve these two issues, a force-sensitive resistor (FSR) was chosen as a simple sensor that can detect breathing while ignoring most of the disturbances created by a subject’s body movements. The force sensor is separated mechanically from any external forces by an enclosure and held on to the same enclosure by a nylon fabric (see Fig. 2). When the enclosure is pushed from the sides or behind, most of the force is directed to the walls of the enclosure, which is made of neoprene foam for ergonomics and force damping. When the nylon fabric holding the FSR is pushed, however, a force is exerted on the FSR, which in turn changes its resistance. FSR resistance is linearly proportional to the force exerted on it, hence its variation in the signal will indicate the variation in force exerted. This sensor is placed on the lower back of the subject. The FSR thus captures force from the movement of the diaphragm during breathing, translating the force into resistance which is further converted into an electrical signal using a resistor network or a voltage divider (see Supplementary Material 1). Choosing an FSR requires consideration of its size and sensitivity. The selected Ohmite FSR03CE has a diameter of 25.42 mm, an actuation force of 0.0981 N (10 g), and force ranges up to 49.0333 N (5 kg). This means that any force greater than 0.0981N (10g) will cause a change in the FSR and will be detected. This FSR is small enough to use for a small respiration sensor that can fit on an infant without getting in the way of the shoulder blade or compromising ergonomics or comfort.

### Movement

#### Accelerometer - LISD3H

The accelerometer is located in the center of the front of the vest, below the cardiac electrodes. The mode of communication between the microcontroller and the accelerometer determines the speed at which the data can be collected. SPI (Serial Peripheral Interface) will support faster data collection than I^2^C (Inter-Integrated Circuit) communication, but SPI requires more ports than I^2^C. Any accelerometer that will guarantee 2000 samples per second (2 ksps) regardless of the mode of communication is the right candidate for this project and will guarantee a sampling rate of 1000 Hz. The LISD3H is a triple-axis accelerometer with a data rate ranging from 1 Hz to 5 KHz. The accelerometer is made by STMicroelectronics and has a low-profile breakout board courtesy of Adafruit Industries, LLC. This accelerometer is fabricated using MEM (microelectromechanical) systems technology, resulting in very low energy consumption. It has 10-bit resolution, which is sufficient for our task, and its sensitivity can be selected as ±2g/±4g/±8g/±16g. For this vest, ±2g sensitivity (roughly equivalent to 19.6 m/s^2^ was chosen. Any force that will move the ball bearing inside the accelerometer faster than 19.6 m/s^2^ will result in a detectable signal.

### Additional sensors

The microprocessor chosen will have a number of remaining pins. It is therefore possible and feasible for users to add other sensors such as galvanic skin response or a universal time clock. Instructions for adding sensors can be found at the end of Supplementary Material 1. The authors caution, however, that the provided GUI and firmware will not automatically collect data from new sensors. The user will have to edit the firmware and GUI themselves to record this new data.

### Fabric and materials

The procedure for stitching and assembling the fabric and materials for the vest has been designed to be as minimally involved as possible. Full instructions, with pictures, can be found in Supplementary Material 2 and will be summarized below. The vest is made of a breathable four-way stretch fabric that allows it to be form fitting, but sturdy enough to maintain the sensors against the skin and support the weight of the electronics. Despite being called four-way stretch fabric, the material purchased may exhibit greater stretch along one axis. For the purpose of this vest, the stretch should be along the left–right axis of the chest. The authors also recommend using two-sided or back-to-back Velcro, as it has a lower profile than regular Velcro, making it easier to attach to the buckles.

A stitch pattern is provided as a standard .dwg file in the Open Science Framework (osf.io/24gp5). This file can be downloaded and printed with a poster printer at 100% scale to trace the outline of the vest, the sew lines, and locations of the electrodes. While the authors used a sewing machine to stitch the vest together, it is possible to complete this task using a fabric adhesive. Care would need to be taken to prevent the glue from clumping and to ensure it dries before applying any of the electronic components.

Typically, developing populations exhibit a wide range of chest circumferences. Measurements taken from local subject populations aged 8 to 24 months at Indiana University revealed a range of chest sizes between 44.45 and 60.96 cm. The constructed vest width without straps is 40.64 cm. The vest will stretch comfortably to a length of 45.72 cm and maximally to a length of 49.53 cm. Straps provide an extra 10.16 cm, making the length of the vest approximately 50.8 cm. With straps the vest will stretch past 60.96 cm to accommodate larger children. Therefore, this vest will be able to measure the average child between the ages of 8 and 24 months of age.

The measurements for the vest may change depending on the age being studied and the constitution of the local population. As head circumference is highly correlated, and approximately equal, to chest circumference (Illingworth & Eid, 1971), the authors recommend referencing the World Health Organization’s growth charts for head circumference as a starting point and measuring a subset of children in their local subject pool. Pilot experiments using this vest have been successful in a cohort of 22 children as young as 8 months and as old as 24 months.

### Applying the vest

The application of the vest to a subject should be done in a private space in the presence of the parent. The vest is designed so that an experimenter or a parent can apply the device easily and with no training. The subject’s shirt should first be removed. While their arms are raised towards the air, the vest should be buckled around the child’s torso and tightened. The straps should be snugly secured over the shoulders and attached to the back of the vest. By design, the front of this vest will not go near the child’s neck and does not present a choking hazard. To ensure optimal data quality, the vest should fit snugly against the skin, and the sensors should maintain constant contact with the skin. If the experimenter notices any rashes or scabs on the child they are about to place the vest on, they should not apply the vest.

The vest has been piloted in a separate experiment on 22 infants with a 100% compliance rate. None of the infants exhibited discomfort or tugged at the vest. When solicited for feedback, parents expressed no concern about the fit or comfort of the vest. In a separate experiment using this vest, sessions lasted on average 1114.21 seconds (minimum 611 seconds, maximum 1676 seconds, standard deviation 269.23 seconds). These pilot experiments were terminated not due to infant fussiness, but because the experimental protocol had been completed. For comparison, in the same experiment, 9 out of the 22 infants (40.9%) rejected wearing a head-mounted eye-tracker. These infants ranged in age from 8 to 24 months.

One consideration when using this vest is that the rubber casing for the electronics may prevent body heat from escaping. None of the infants tested with the vest rejected it; however, these experiments were conducted in an air-conditioned laboratory environment. For researchers conducting studies with this vest outside, or in a hot room without proper ventilation, consideration would need to be made to ensure a participant does not overheat.

For the below data, the research protocol was approved by the Human Subjects and Institutional Review Board at Indiana University (Protocol #1901954827). The parents volunteering their infant for the study were fully informed of the study procedures and completed written informed consent and permission forms in advance.

### Cleaning the vest and safety

The vest has been designed to maximize its ability to be sterilized and cleaned for multiple uses. First, the electronics are isolated between two rubber mats which can be washed with soapy water or wiped with a disinfectant wipe. The cloth electrodes are attached to snap buttons and can be removed and cleaned with disinfectant wipes or discarded and replaced with new ones. The fabric cover is removable and can be washed separately in a washing machine. The authors recommend cleaning the electrodes and vest after each experiment. The authors have repeatedly cleaned the vest over 30 times in the span of two months using disinfectant wipes. There was no noticeable reduction in signal quality. Nonetheless, the authors recommend checking the signal quality frequently by starting the vest and laying one’s hands on the cloth cardiac electrodes. This skin contact is usually sufficient to detect a heartbeat. After visually inspecting the output and confirming signal quality, the vest should be disinfected and prepared for the study. If signal quality appears deteriorated, the authors recommend replacing the cloth electrodes. Assuming the vest is not unreasonably handled, there should be no degradation in either the respiration sensor or the accelerometer.

There is no risk of shock from using this vest. The only electronic components in direct contact with the participant’s body are the three cloth electrodes for measuring cardiac activity. Each cardiac electrode is connected to a 180 KΩ resistor. The ground electrode is connected to a 360 KΩ resistor. These resistors restrict the maximum flow of current to 10 μA. For comparison, licking a 9 V battery would result in a current of roughly 1.3 mA, which is equivalent to 1300 μA. Further, all components of the autonomic vest are either enclosed in a plastic casing or wrapped in a rubber foam pad, increasing both electrical and heat insulation. The battery pack is also enclosed outside the vest in a strong plastic casing and is connected to the vest via a USB port that is easily detachable.

## Software

### Graphical user interface

The authors provide a GUI for the use of the autonomic vest as an .m file that can be run using any current version of MATLAB. This GUI was developed using MATLAB 2019b. The interface allows the user to view the data with a ~1–2-second delay, which facilitates electrode placement and ensures data quality is sufficient to continue the experiment. The GUI can also save the displayed data, which will be at a variable sampling rate, as a backup to the 1000 Hz data in the SD card. The vest will not start writing data until the Start button is pressed and will stop data writing once the Stop button is pressed. To ensure accurate analyses, the authors recommend using the data written onto the SD card and suggest using the GUI-collected data for pilot purposes only. For example, the visualized cardiac waveforms will be missing a spike or will exhibit two heartbeats in close proximity to each other. These errors are due solely to data transmission delays with Bluetooth and are not reflected in the data written on the SD card. The authors have also included an event marker button, which can manually mark the onset of events relevant to future data analysis.

As the vest’s firmware is designed to interface with MATLAB, it is possible to directly interface with the vest using Psychtoolbox through Bluetooth. The MATLAB GUI provided by the authors communicates to the vest through a COM port. To interface with the vest using Psychtoolbox, the user would need to identify that same COM port. From there, the ability to collect the data gathered by the vest is straightforward and will closely mimic the code used in the GUI. The authors refer interested researchers to read the provided GUI’s .m file and adapt the code for their purposes.

### Calculating heart rate

While there are many reliable open-source analysis programs for analyzing raw echocardiogram data (Thorson et al., 2018; Vest et al., 2018), the authors chose to directly compute heart rate using MATLAB. First, motion artifacts or signal cutoffs were manually identified and isolated. In this study, regions exhibiting signal loss or motion artifacts were replaced with NaNs (not-a-number; MATLAB). To remove drift in the signal, the 1000 Hz cardiac data was high-pass-filtered at 5 Hz to preserve the rapid waveform of the heartbeat. Heartbeats were detected using an adaptive threshold of one second duration to find cardiac spikes greater than the 95th percentile of the amplitude at each second of the signal (Borjon et al., 2016; Gustison et al., 2019). To calculate heart rate, we constructed a binary series of heartbeat counts and convolved the resulting series with a five-second Gaussian window. This method is often used in estimating the instantaneous rate of action potential occurrences in neurophysiological data (Shimazaki & Shinomoto, 2010).

### Calculating respiration rate

Similar to the calculation of heart rate, the authors chose to directly calculate respiration rate using MATLAB. For this study, segments of respiratory signals that did not clearly demonstrate respiration were excluded from further analysis by being replaced with NaNs. Respiratory signals were down-sampled from 1000 Hz to 50 Hz. To remove drift in the signal, the resulting data was high-pass-filtered at 5 Hz. The phase coordinates of the respiratory signal were then determined by calculating the angle of the Hilbert transform of the detrended signal (Borjon et al., 2016; Gustison et al., 2019). To calculate respiratory rate, the points in time where the angle of the Hilbert transform was 0 were detected. These time points represent the beginning of expiration and were converted into a binary representation and convolved with a 15-s Gaussian window. Again, this method is often used in estimating the instantaneous rate of spike occurrences (Shimazaki & Shinomoto, 2010).

## Results

**Fig. 3:**
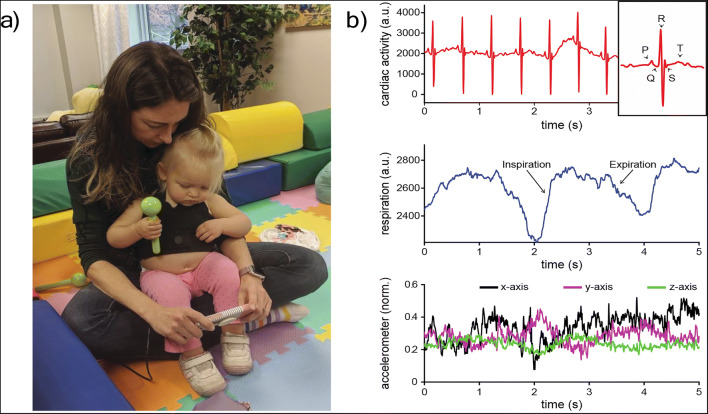
*An exemplar of raw data from the autonomic vest.* (**a**) An image of the vest applied to a 16.7-month-old child. She is sitting on her parent’s lap and watching a video on a phone, sustaining her attention to the object. (**b**) Five seconds of the raw data from the autonomic vest. Cardiac activity is in the top row with clear P, Q, R, S, and T waveforms identified in the inset. Respiratory activity is in the middle row with moments of inspiration and expiration identified. The third row demonstrates normalized data from the *x*-, *y*-, and *z*-axis of the accelerometer

To demonstrate evidence for the validity of this vest, the authors applied the vest to two infants 16.7 and 16.2 months of age. An image of one of the subjects wearing the vest can be seen in Fig. 3a, and five seconds of the collected raw data in Fig. 3b. One verification of the vest is to compare the collected data to known clinical landmarks. Heartbeats are electrical impulses that travel across the four chambers of the heart, which contract and relax in a coordinated and stereotyped fashion. The P, Q, R, S, and T components of the heartbeat can be clearly identified in Fig. 3b. The P wave corresponds to atrial contraction, and the electrical impulse spreads through the ventricles to form the QRS complex. Finally, the ventricles repolarize, resulting in a delayed T wave. A subset of 500 randomly chosen heartbeats were visually inspected by a research assistant. Of these randomly chosen heartbeats, 496 (99.2%) exhibited identifiable P, Q, R, S, and T waveforms. In addition to these physiological components, clear inhalation and exhalation can be observed along with variations in movement across the *x*-, *y*-, and *z*-axis of the accelerometer.

**Fig. 4:**
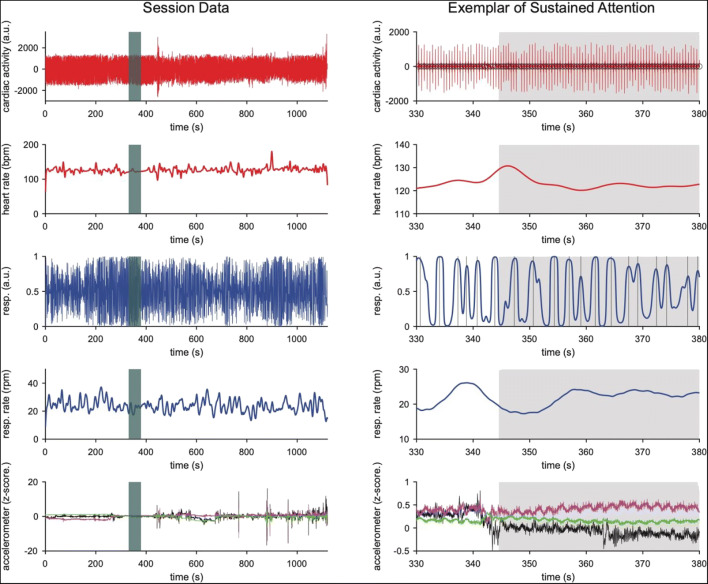
*A period of sustained attention.* At left is the entire session of data from the subject pictured in Fig. 3, with the shaded region in green indicating the exemplar period of time at right. At right is 50 seconds of data from the autonomic vest, exhibiting a period of time in which the subject was sustaining attention to a video on a cell phone. The shaded region in gray indicates the onset of attention. For both columns, the first row exhibits the filtered cardiac data, and the second row demonstrates the calculated heart rate. The third and fourth rows demonstrate the filtered respiratory signal and the calculated respiratory rate, respectively. The fifth row demonstrates the normalized signal from the three-axis accelerometer

The filtered data, including calculated heart rate and respiration rate, can be observed in Fig. 4. This exemplar data was collected for 1121.2 seconds, and Fig. 4a plots the data across the entire session. The average heart rate for this session was 126.45 beats per minute (bpm), with a standard deviation of 8.169 bpm. The average respiration rate was 23.88 respirations per minute (rpm), with a standard deviation of 4.24 rpm. These session-level analyses demonstrate that the derived heart and respiratory rate measures exhibit minimal variability and accurately reflect the collected heartbeats and respiration. The unfiltered session data and the calculated heart and respiratory rates are provided as supplementary data to this article. Due to a data corruption error, this data does not have a corresponding video. Nonetheless, there is enough data to see the variability of the signal over a long period of time and during a session where the infant was walking around as well as sitting. This session is not curated or edited, meaning there are periods of noise where the vest shifted during play and was then readjusted by the researchers or the caregiver. The majority of the session, however, has reliable and identifiable heartbeats and respiratory activity.

A second verification was to capture a period of sustained attention. To achieve this, the child sat in her mother’s lap and watched a video on a cell phone. This period of quiescence facilitates attention, beginning with a brief shift in posture (bottom row, right column of Fig. 4). This shift of movement occurs during a period of low respiratory rate and a simultaneous increase in heart rate. Movement, heart rate, and respiratory rate stabilize shortly after these changes.

To further illustrate the use of the accelerometer in this vest, the authors provide a six-minute video of a subject wearing the vest while playing at a tabletop with their caregiver (Video 1). The collected accelerometer data is plotted beneath the video and, for convenience, the authors have indicated the onset of several behaviors of interest for the first minute of the video. At 0:17, the caregiver bounces their child on their knee, resulting in very clear oscillations along the *y*-axis. At 0:25, the child leans over to reach for a toy, changing the *x*-axis output, then reaches across the table at 0:30 and at 0:50, eliciting changes in the *z*-axis. There is a vibrating, light-up toy that the child turns on at 0:35, which causes vibrations that can be seen on all three axes of the accelerometer. At 0:40, the child presses the vibrating light-up toy against their chest, resulting in very large oscillations in the accelerometer. While uncoded, the remaining five minutes provide other examples of naturalistic child interaction. The authors conclude that the use of the accelerometer is sufficient for identifying movement, and changes in its output are consistent with the infant’s movement.

## Discussion

The authors have described the development of a wireless vest designed to measure autonomic function in infants 8–24 months of age. The components of the vest, from its material to sensors, were chosen to optimally and accurately capture these biological signals. Evidence for the validity of the vest has been provided in three ways. First, the components of a heartbeat were detected. Second, during a period of quiescence, changes in autonomic function were observed consistent with a period of sustained attention. Third, a six-minute video is provided in which changes in the child’s movement relate directly to changes in the accelerometer. Complete instructions for constructing this lightweight and inexpensive vest are provided in the supplementary materials. The relevant template, software, and firmware are provided in the Open Science Framework. As updates to MATLAB will necessitate changes in the firmware and GUI, the resources in the Open Science Framework (osf.io/24gp5) will be updated for at least a year and a half from the date of publication.

The instructions provided in the supplementary materials should be sufficient for interested parties to develop their own wireless autonomic vest and adapt the design for use in other populations. To ensure the collected data is reliable, it is possible to collect the same physiological data across multiple sensors. For example, both the cardiac electrodes and the accelerometer can pick up breathing patterns (Fig. 4, bottom row, right column). There is also some indication in the accelerometer that it can pick up heartbeats; however, the accelerometer may be too sensitive to other movement to be reliably used alone for the detection of cardiorespiratory activity. It is also possible to use the vest in tandem with other methodologies. The provided event marker button in the GUI or interfacing with the provided Arduino software or through Psychtoolbox will support the synchronization of the autonomic vest with other resources such as video recording or eye-tracking.

One avenue for future research supported by the present vest and software is the opportunity for researchers to design real-time triggering experiments. By reading the data from the device through programs such as Psychtoolbox, it is possible to design experiments where stimuli are presented or change their properties based on the calculated autonomic activity. For example, it would be possible to advance through an experimental condition only if the child were able to maintain their heart rate variability within a certain range. Similar experiments for infants have been designed around gaze-contingent responses in eye-tracking (Keemink, Keshavarzi-Pour, & Kelly, 2019; Wang et al., 2012).

A second avenue for this vest to advance child development research is its ability to be used in many contexts. Any laptop running MATLAB can connect to this vest, so long as the Bluetooth dongle is connected to the machine and the vest is within range. Researchers are thus able to collect dense physiology data from the lab, home, and even outdoors. However, as mentioned in the Methods section above, exposure to the outdoor sun may make the vest uncomfortable due to the rubber encasing. Nonetheless, the ability to tractably study the autonomic nervous system activity supporting infant behavior in multiple contexts is a fruitful avenue of future research, particularly whether laboratory-controlled studies of infant behavior reflect the variability of natural infant behavior “in the wild”.

Behavior is the product of nested systems operating at multiple time scales. It is therefore critical to support the development and advancement of new technologies that can accurately and densely capture the multiple scales at which behavior emerges. The vest described here supports the measurement of three indices of autonomic function through time. The use of this vest to study child behavior has implications across the field of developmental psychology and behavior research in general.

## Electronic supplementary material

ESM 1 (PDF 21079 kb)

ESM 2 (PDF 22733 kb)

ESM 3 (MAT 42893 kb)

ESM 4 (MP4 140582 kb)
