# Supplementary material for: An open-source, wireless vest for measuring autonomic function in infants
Source: Behav Res Methods. 2020 Apr 24;52(6):2324–37. doi: 10.3758/s13428-020-01394-4 (PMC7584781; doi:10.3758/s13428-020-01394-4)
Supplement: Supplementary file 1 — (PDF 21079 kb) [file 13428_2020_1394_MOESM1_ESM.pdf]

An open-source, wireless vest for measuring autonomic function in infants:

Electronics

Table of Contents:

02 – Materials, Tools & Equipment
03 – Cutting Rubber Sheets
07 – Embedding Electrode Bases
11 – Connecting Heart Monitor
16 – Connecting Accelerometer
19 – Making & Connecting Respiration Sensor
26 – Connecting Micro-controller
33 – Testing
34 – Sealing the Components
44 – Adding more Sensors
47 – Programming the microcontroller
56 – Programming the Bluetooth modules
76 – Assembling the battery unit

All templates, firmware, and 3D printable files can be found on the Open Science Framework (osf.io/24gp5)

MATERIALS, TOOLS & EQUIPMENT

MATERIALS

- Rubber sheet
- Rubber cement
- Rubber pads
- Carpet tape
- Metallic snap buttons
- PCB board
- Super-glue
- Conductive fabric
- Solder
- Electronics wire stranded 22 AWG and above
- Accelerometer - LIS3DH
- Heart Monitor - AD8232
- Force Sensor
- 3D-printed Heart Monitor Case
- 3D-printed Micro-controller Case
- Teensy 3.6 Micro-controller

TOOLS & EQUIPMENT

- Hacksaw blade
- Utility knife
- Soldering iron
- Wire/ stiff brush
- Clamps/ weights
- Multimeter

CUTTING RUBBER SHEETS

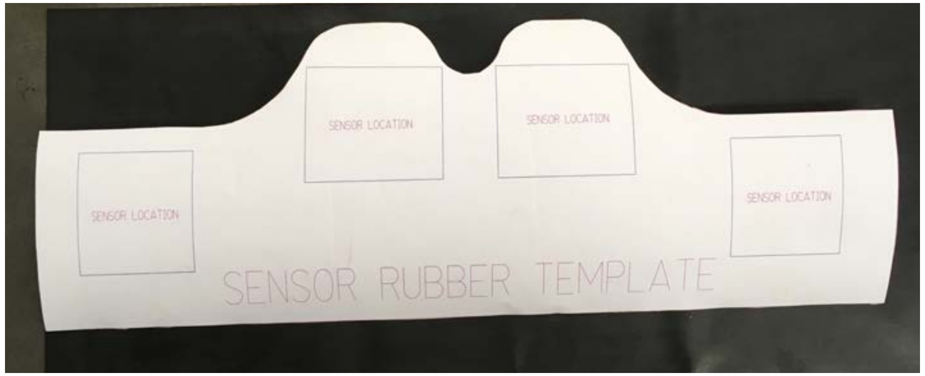

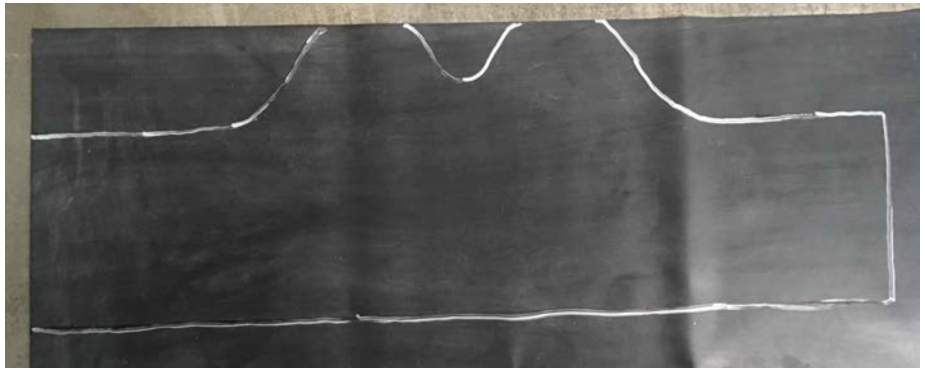

- Acquire rubber sheet, clean it with alcohol, or rubber cleaner, or dish soap & water and let it dry.
- Lay the clean rubber sheet on a flat surface and mark the outline using the Sensor Rubber Template.
- Using a pair of scissors cut along the marking.

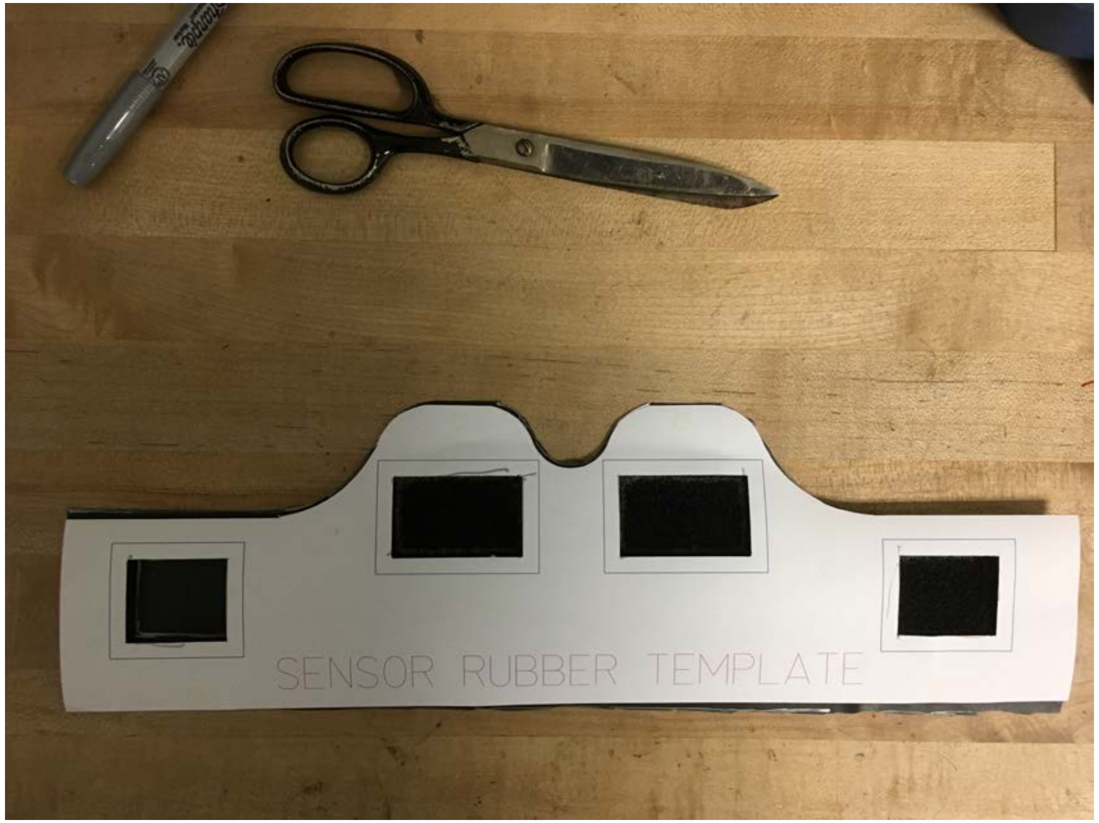

- Using the Sensor Rubber Template with electrode areas cut out, mark the Electrodes & Respiratory Sensor areas.

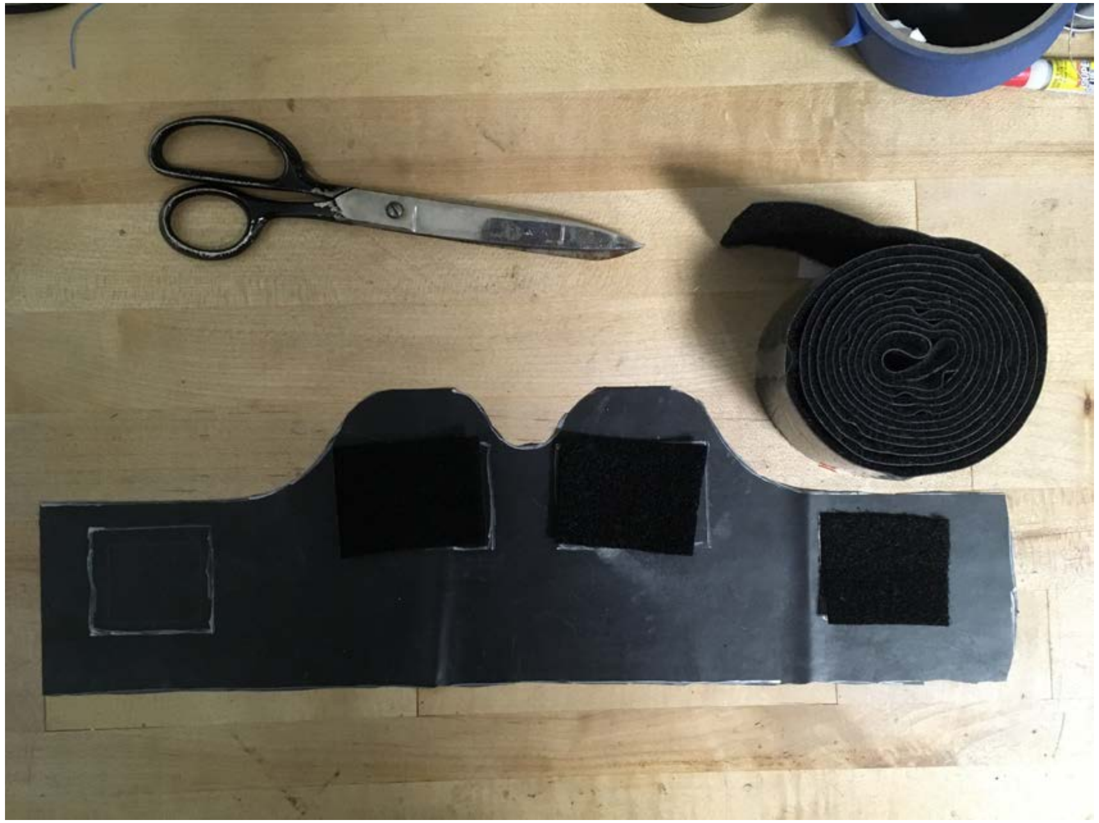

- Measure & cut adhesive-backed Velcro that fits the electrode areas as shown (65 mm x 45 mm).
- Peel and stick the Velcro pads on the electrode areas.

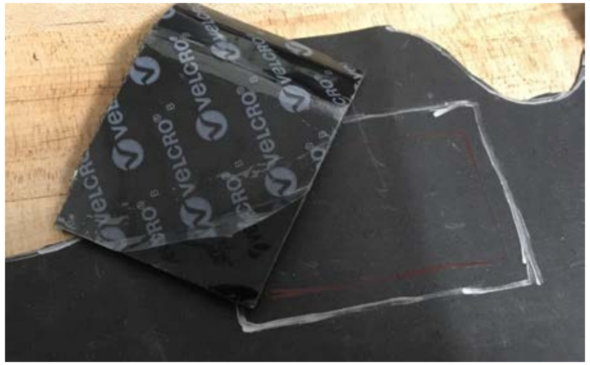

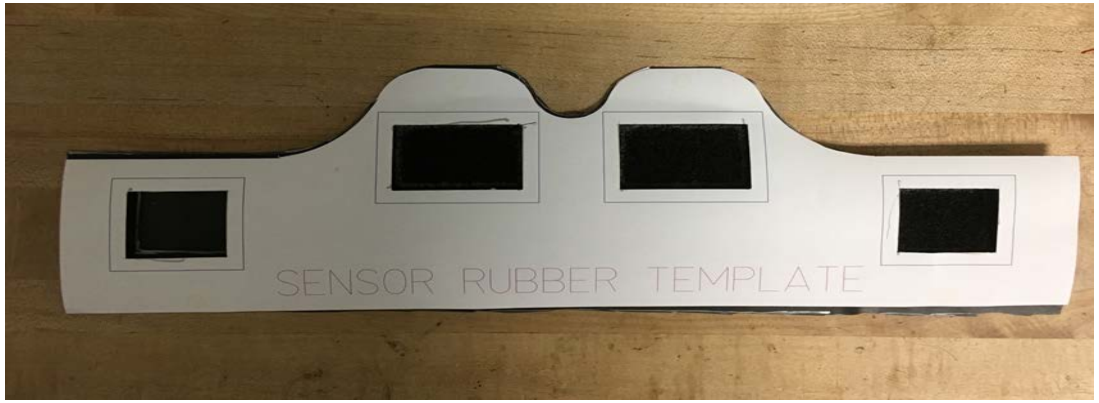

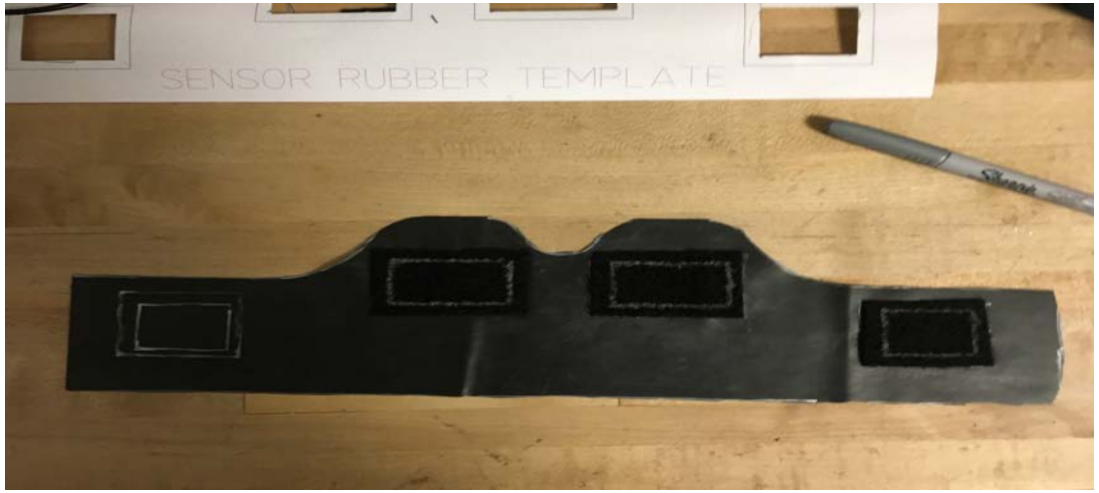

- Using the template, mark the electrode base areas as shown.

EMBEDDING ELECTRODE BASES

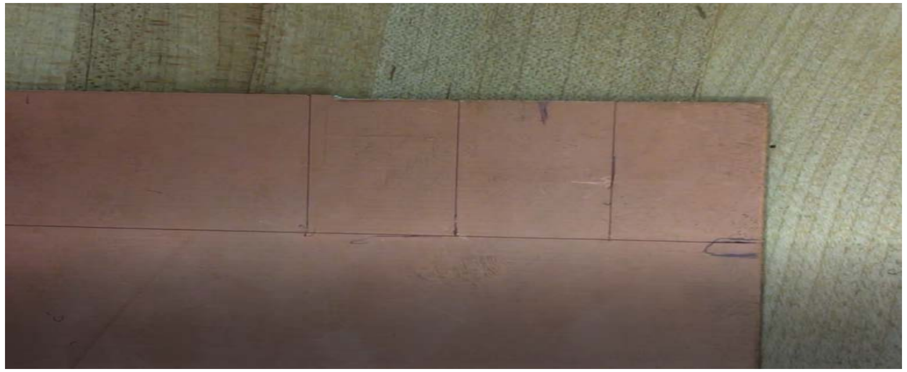

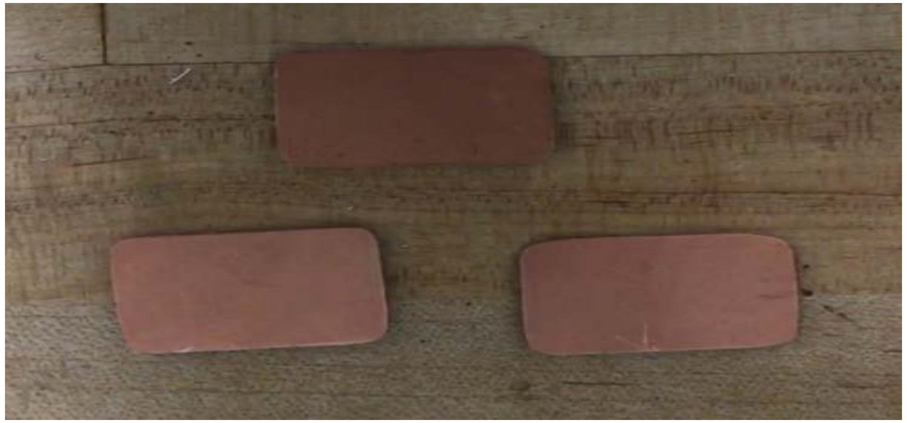

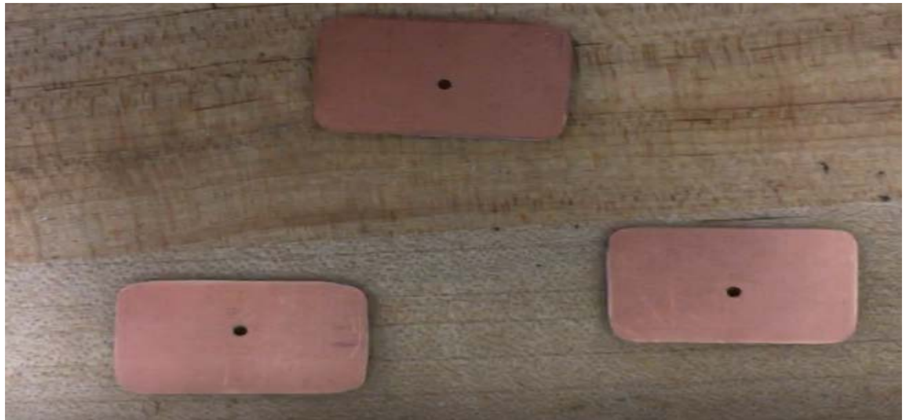

Measure & cut PCB board for electrodes

- Acquire a piece of PCB board, measure and mark three (20 mm x 30 mm) pieces.
- Using a hacksaw, cut out the pieces.
- File the edges of the cut pieces.
- Using a drill, bore a hole in the middle of the cut PCB boards as shown.

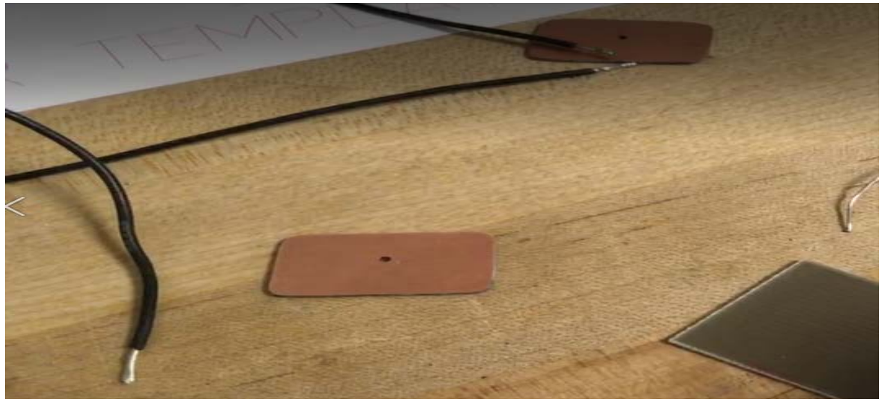

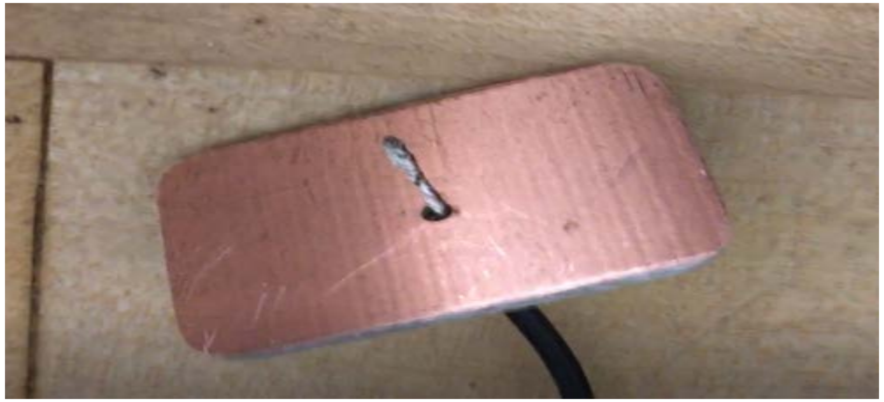

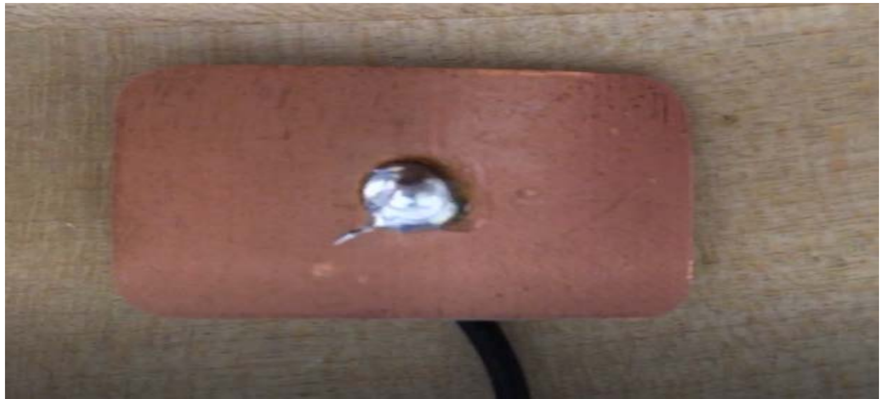

Solder electrode wire to the PCB base

- Measure and cut three (300 mm long) wires for the electrodes.
- Strip the ends about 10 mm and solder.
- Insert the soldered end in the PCB hole as shown and solder.
- Repeat for the other two wires and PCB boards.

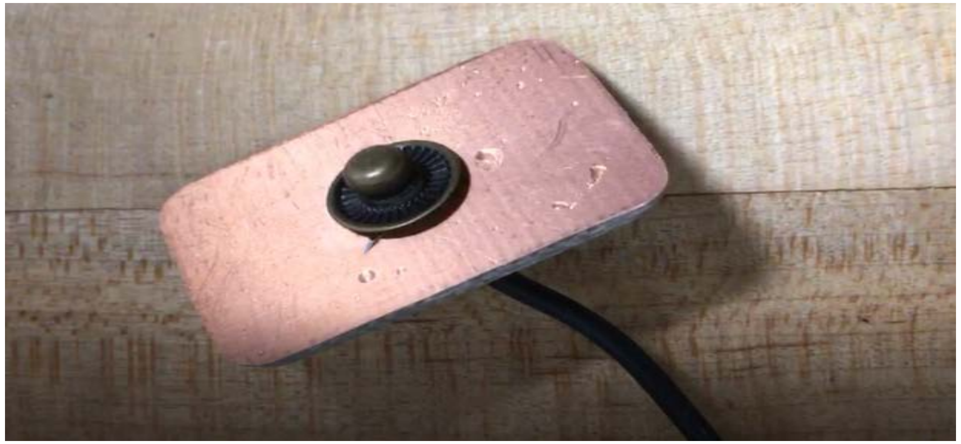

Solder the snap button

- File the metallic snap buttons on the surfaces that will be soldered (the base).
- Place a filed snap button as shown in the picture.
- Solder the snap button to the PCB surface.
- Using a wire/stiff brush, clean the soldered button and the PCB surface.
- Repeat for the other two PCB boards.

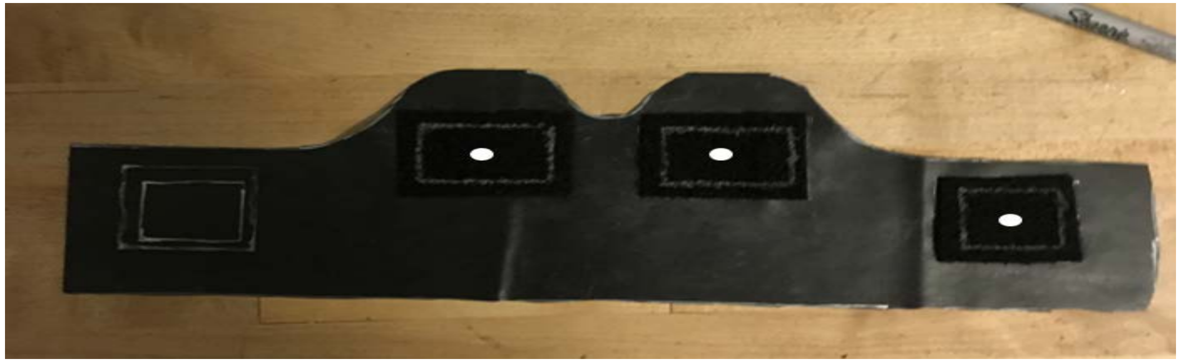

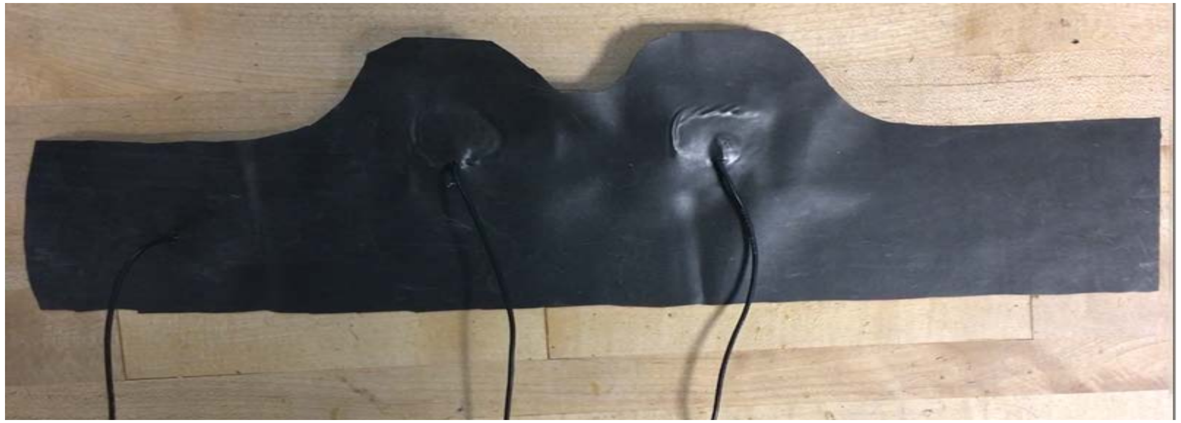

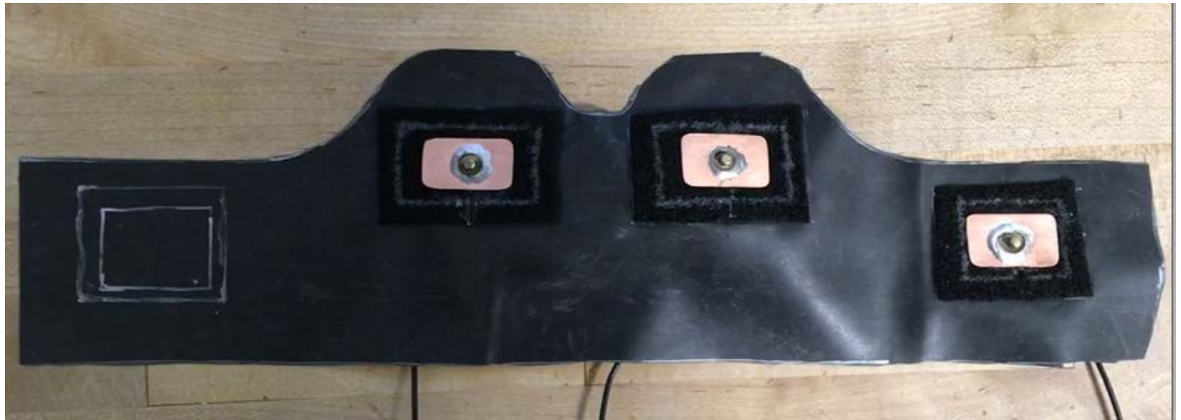

Attach the electrode bases

- Using soldering iron, make a hole (2 mm diameter) at the center of the Velcro pads.
- Insert the electrode wires through the holes as shown.
- Using super-glue, attach the PCB to the Velcro pads as shown.

CONNECTING HEART MONITOR

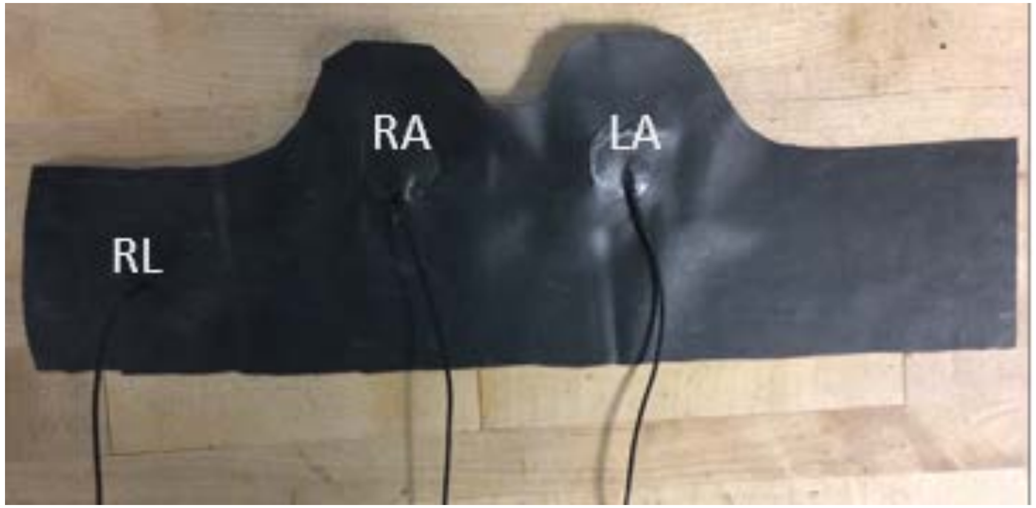

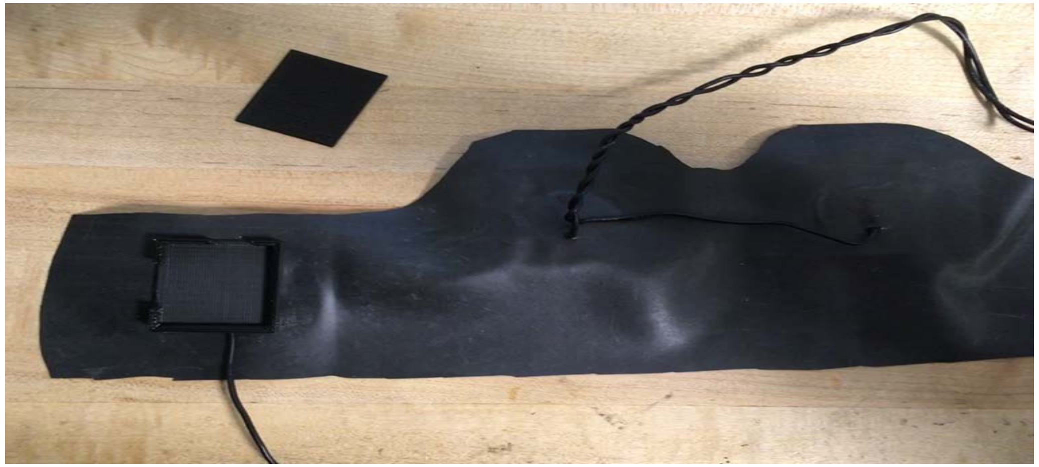

Attach the heart monitor case

- Mark the ends of the electrode wires to remember which wire is connected to which electrode.
- Twist the two front electrode wires together as shown in the second picture.
- Using super-glue, attach the Heart Monitor case right on top of the RL electrode’s spot, as shown in the second picture.
- Firmly press the case onto the rubber for at least a minute to let the super-glue set.

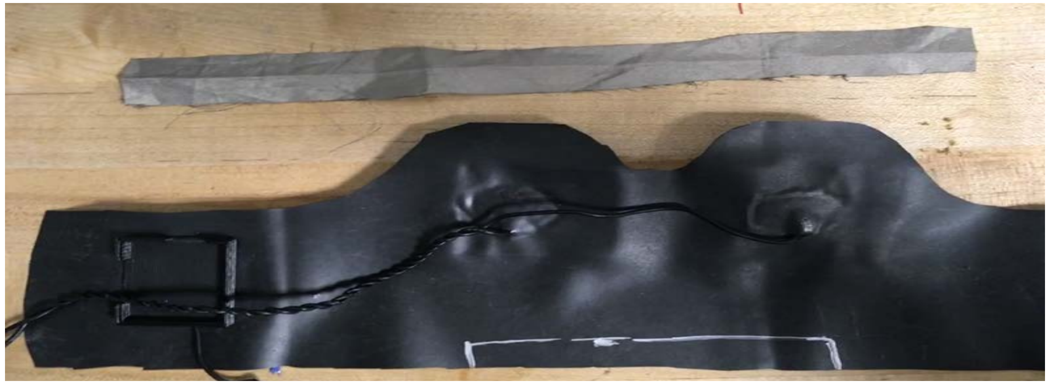

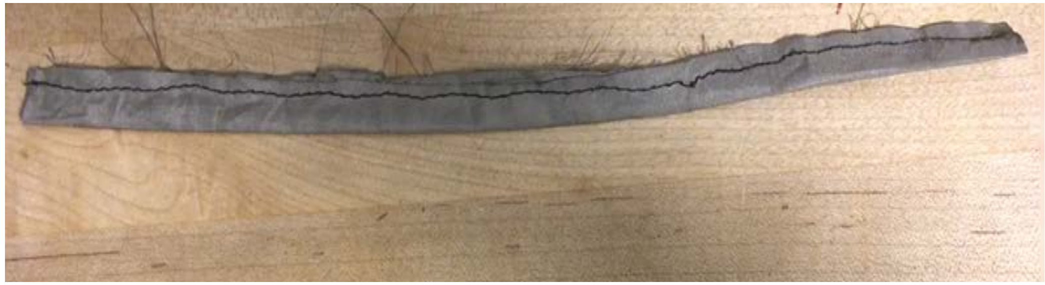

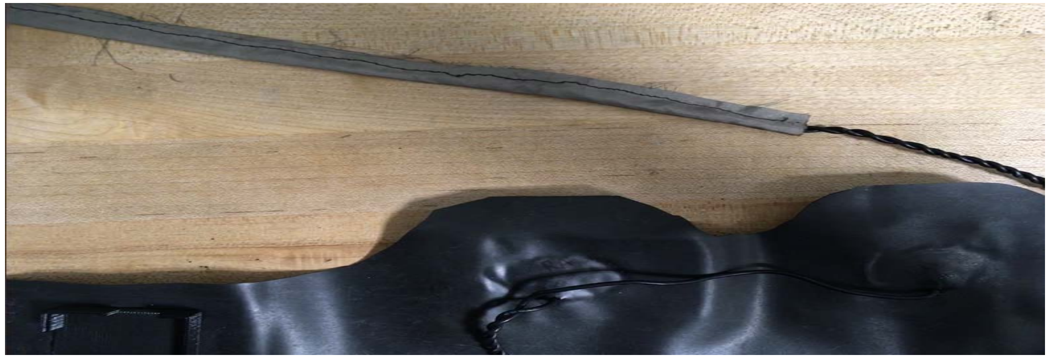

Shield electrode wires

- Measure and cut a strip of conductive material (approx. 200 mm x 20 mm).
- Sew the long edges together as shown.
- Run the twisted pair of electrode wires through the conductive material tube, as shown.

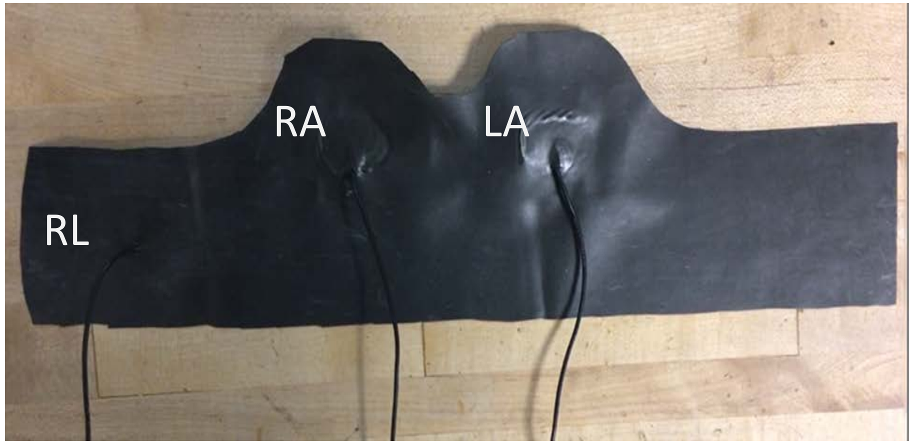

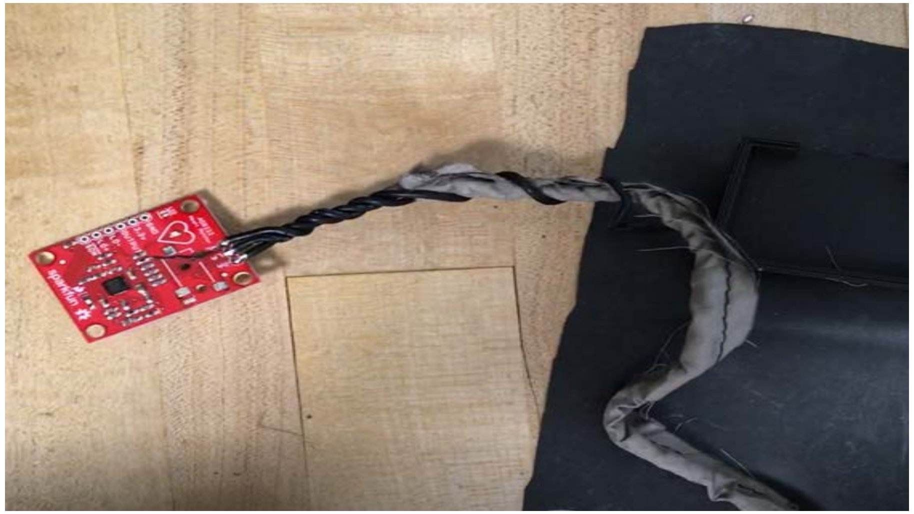

Connect electrode wires to heart monitor

- Solder the electrode wires to their respective leads on the Heart Monitor.
  RA → RA lead, LA → LA Lead, RL → RL lead

*** The Heart Monitor on the picture has the auxiliary jack port removed to maintain a low profile. If you choose to keep the auxiliary jack, you may need to adjust the 3D printed case to your needs. ***

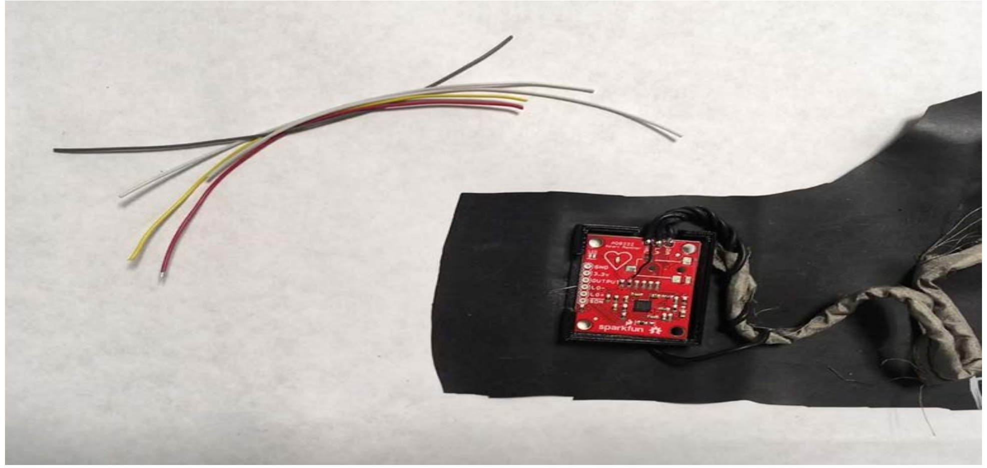

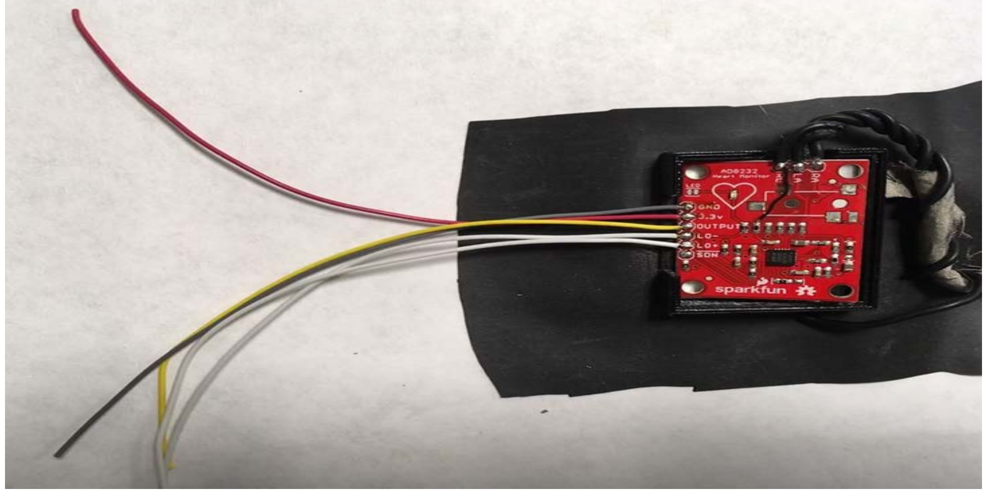

Connect power & output wires

- Cut five different colored (approx. 90 mm long) cables for interfacing the Heart Monitor to the Micro-controller.
- Strip the ends and solder them to the Heart Monitor.
- Record the color code (e.g. Red – Vcc, if red is connected to the Vcc lead).

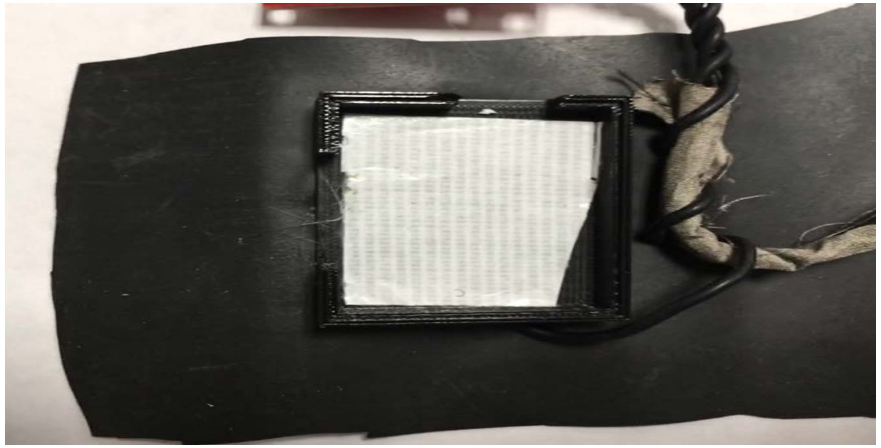

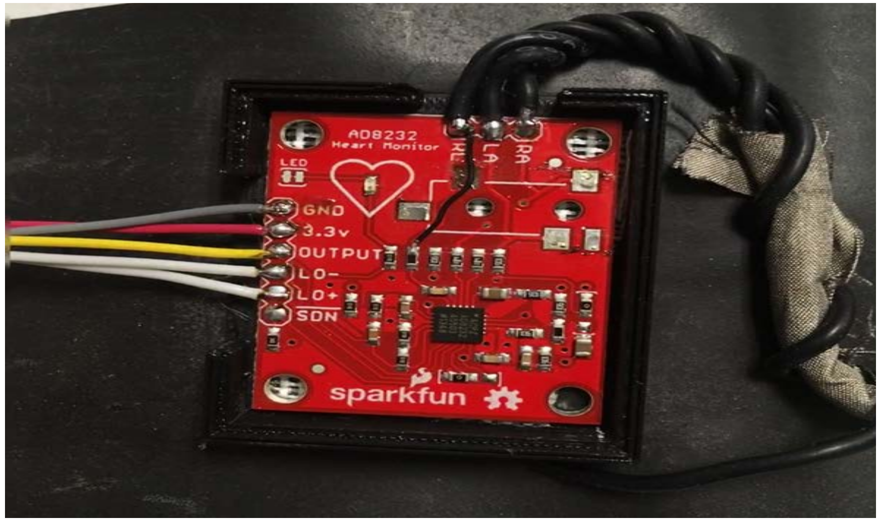

Attach the heart monitor

- Cut a piece of carpet tape and stick it inside the Heart Monitor case.
- Peel and stick the Heart Monitor as shown.

CONNECTING ACCELEROMETER

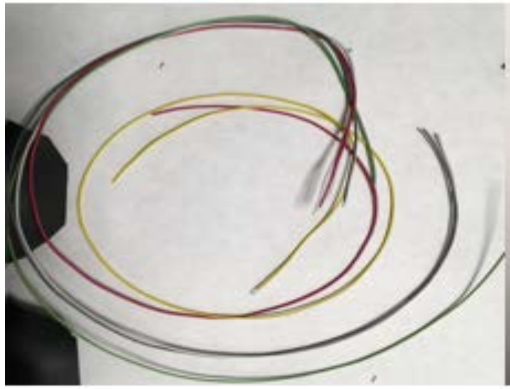

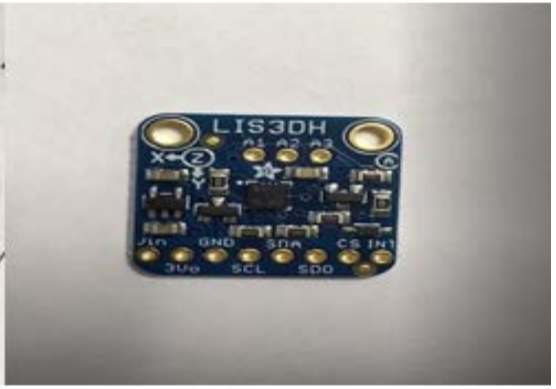

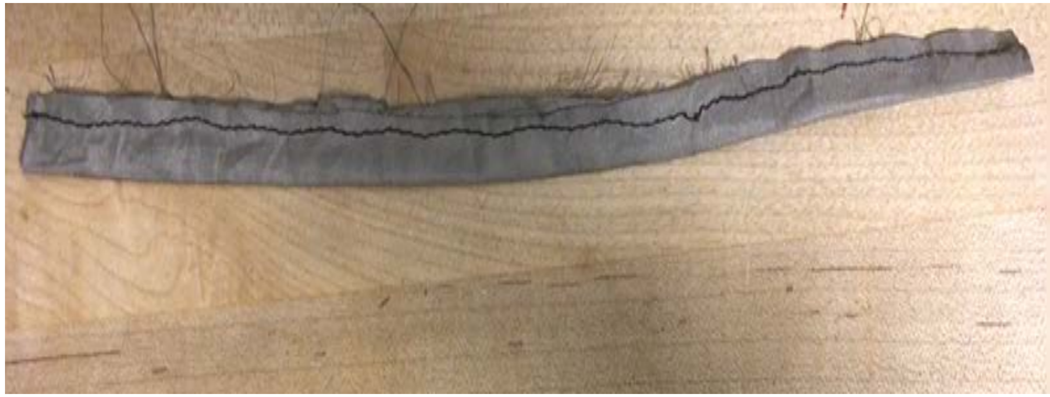

- Cut seven (300 mm long) different colored wires.
- Measure and cut a strip of conductive material (approx. 250 mm x 20 mm).
- Sew the long edges together to make a tube for shielding the wires, as shown.

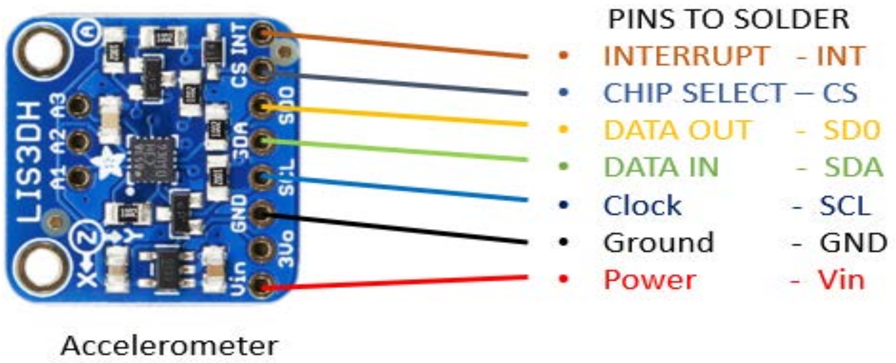

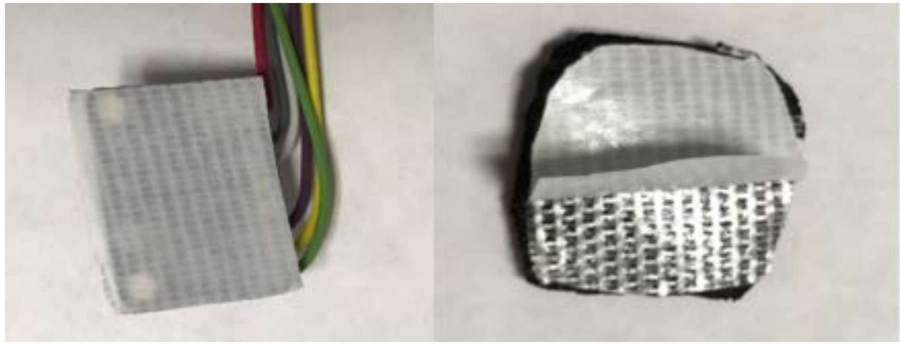

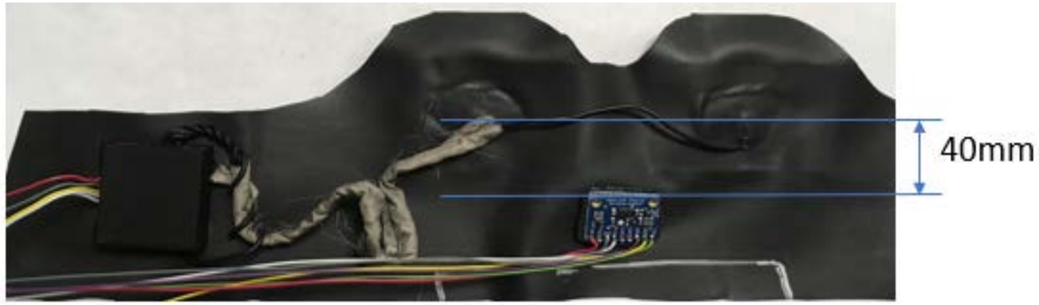

Solder the wires

- Strip and solder the wires, as shown (you can use a different color code).
- Cut a piece of 6.35 mm thickness Neoprene foam (25 mm x 25 mm).
- Put carpet tape on one side.
- Peel the tape and stick the Neoprene onto the vest 40 mm below the center of Right Arm (RA) and Left Arm (LA) electrodes.

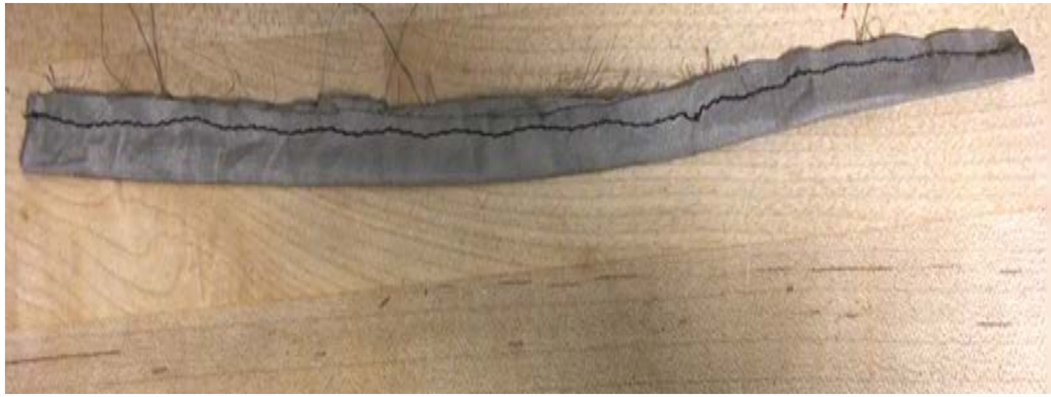

- Run the soldered wires through the conductive fabric shielding sewn earlier.

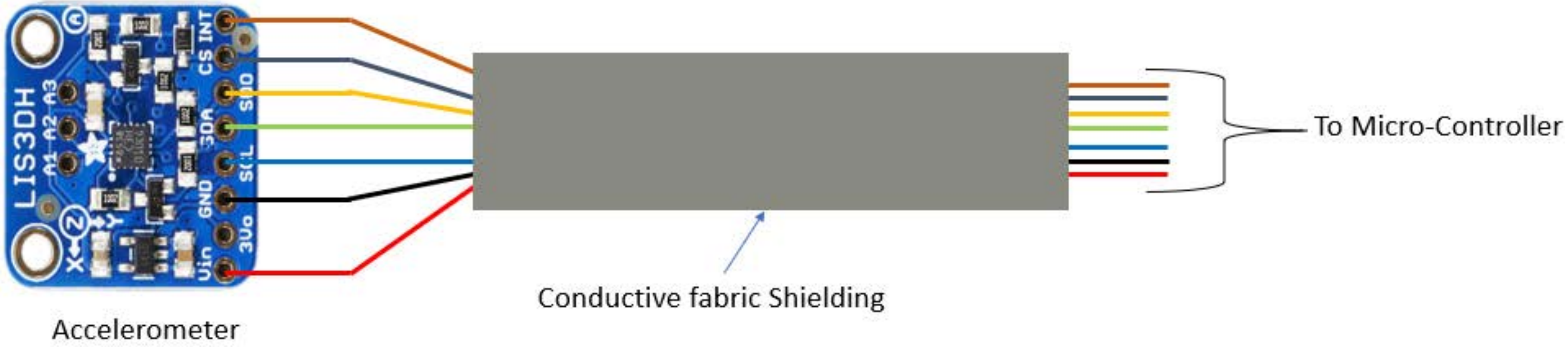

MAKING & CONNECTING RESPIRATION SENSOR

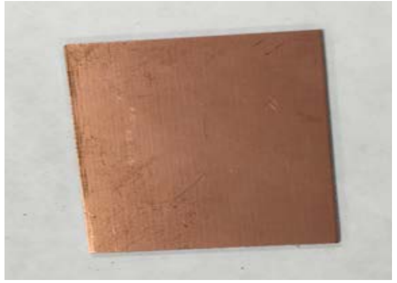
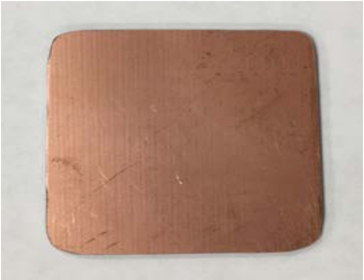
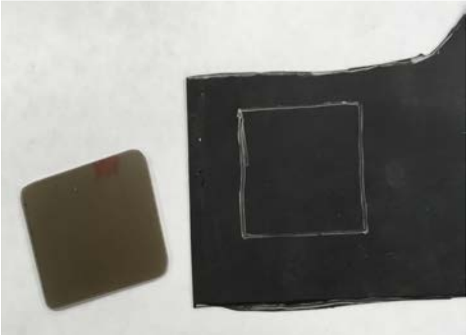
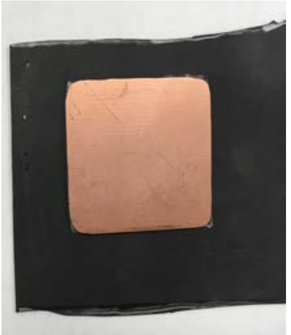

Cut & glue the back board

- Using the template, measure and cut a PCB board, the same size as Respiration Sensor area (50 mm x 45 mm).
- File to smooth the edges and round the corners.
- Using super-glue, stick the PCB board on the sensor area on the rubber, as shown.
- Press firmly and hold for a minute to let the glue set.

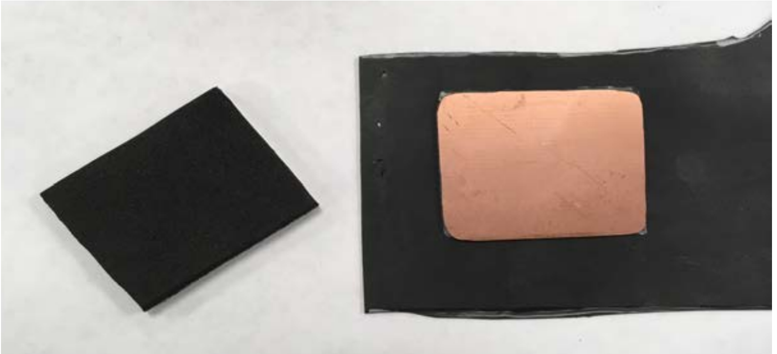

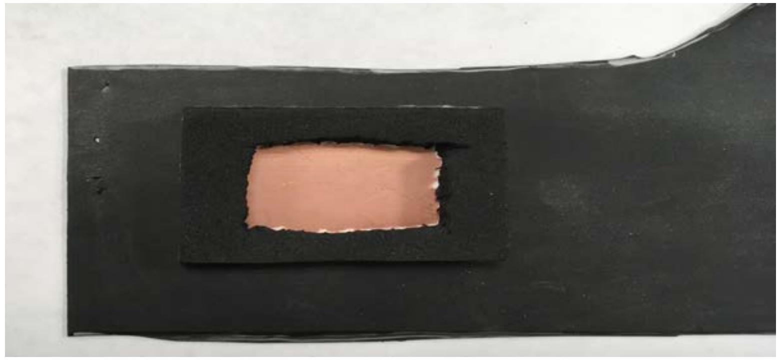

Cut & glue neoprene foam

- Measure and cut Neoprene-foam (50 mm x 45 mm).
- Cut a rectangular hole (16 mm x 16 mm) roughly at the center.
- Peel the back tape and stick it to the PCB board.

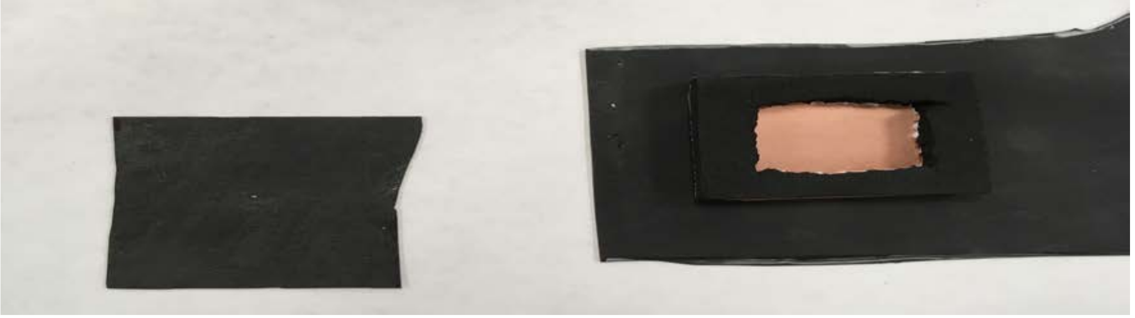

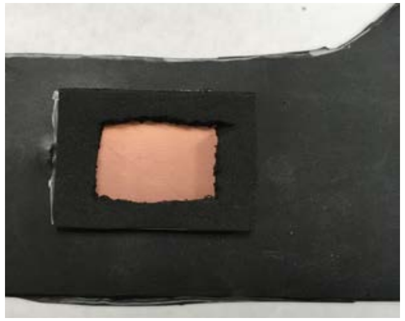

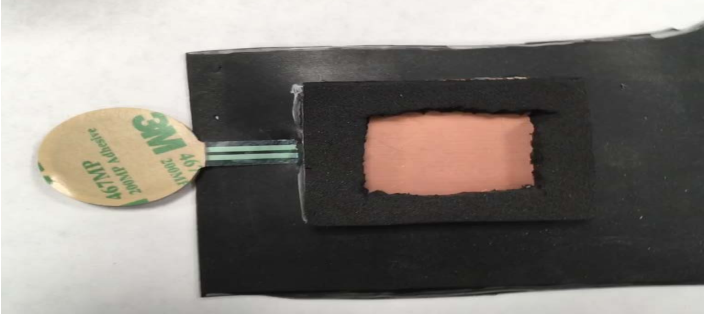

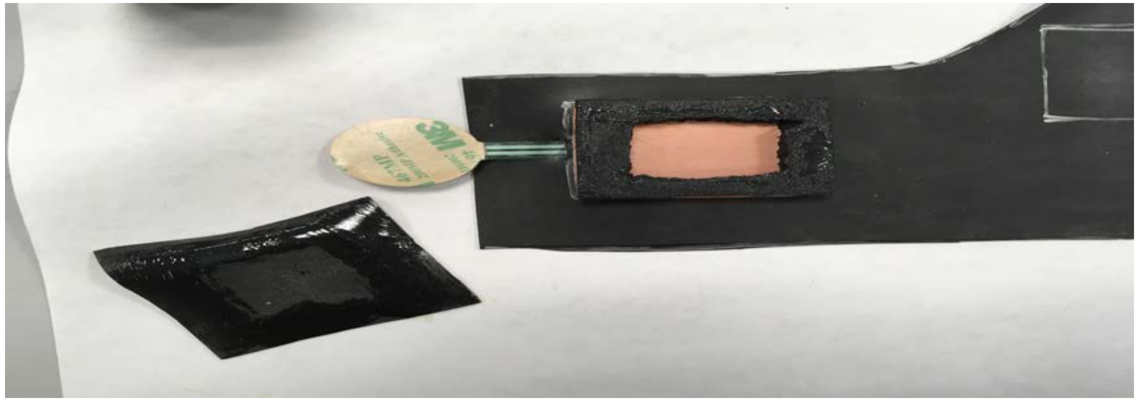

Attach force sensor

- Measure and cut a piece of rubber sheet (65 mm x 60 mm).
- Using a hacksaw blade, roughen the surface of the rubber.
- Cut a 5 mm slit, as shown, and run the force sensor wire through.
- Apply rubber cement about 10 mm from the edges around the rubber sheet.
- Apply rubber cement on top of the Neoprene foam and its sides.
- Leave the rubber cement to become tacky.
- Stick the rubber sheet to one end and stretch it while moving to the other end.

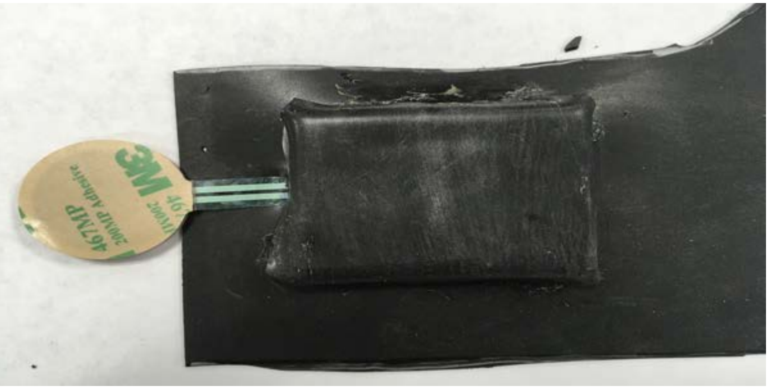

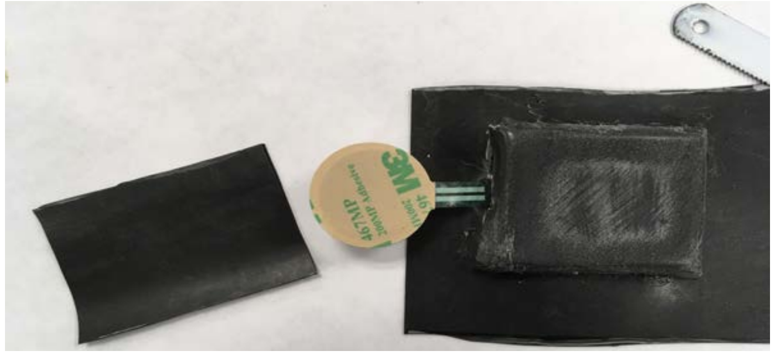

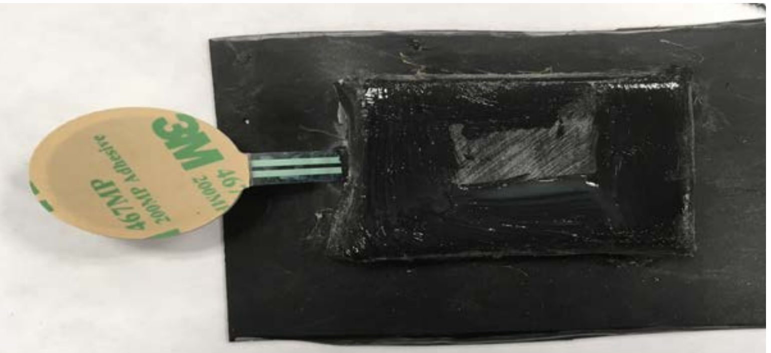

- Tack the sides of the rubber sheet to the sides of the neoprene foam.
- Ensure that the rubber sheet tension keeps it from collapsing (top flat surface).
- After 10 min, roughen the top and sides of the rubber.
- Measure and cut a piece of rubber sheet (65 mm x 60 mm).
- Roughen the surface of the piece.
- Apply rubber cement to the roughened surfaces and wait for it to become tacky.

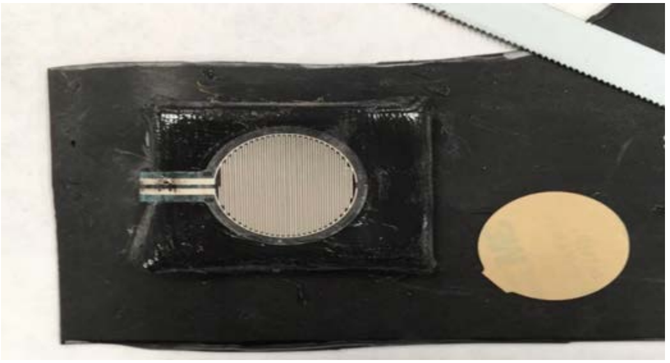

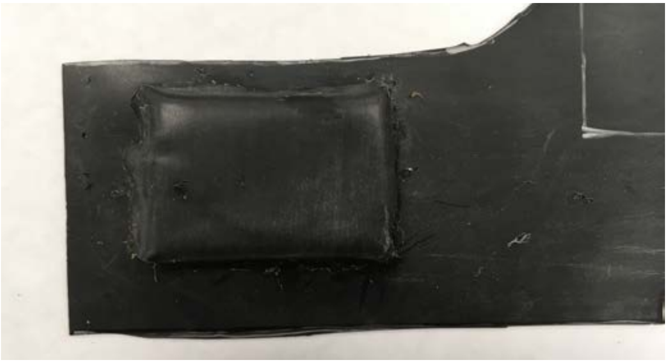

- Once the cement on both surfaces is tacky, peel and stick the Force Sensor at the center as shown.

- Cover the sensor with the rubber sheet and tack the sides.

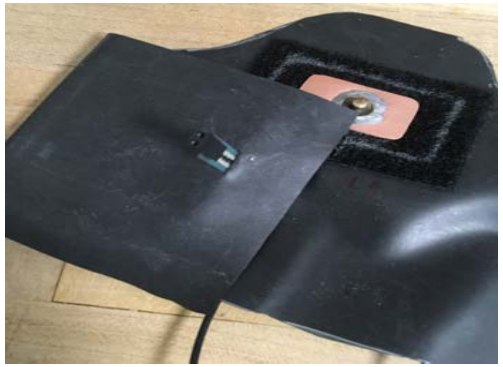

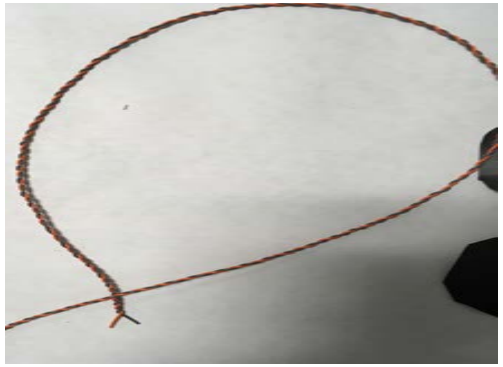

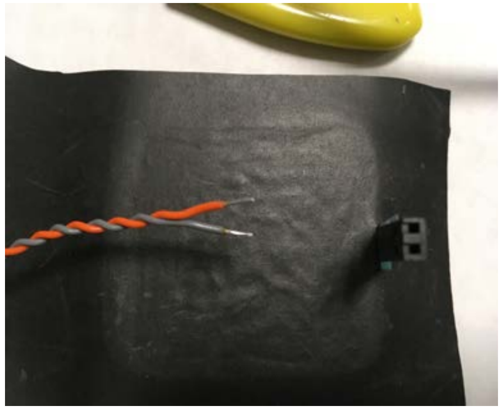

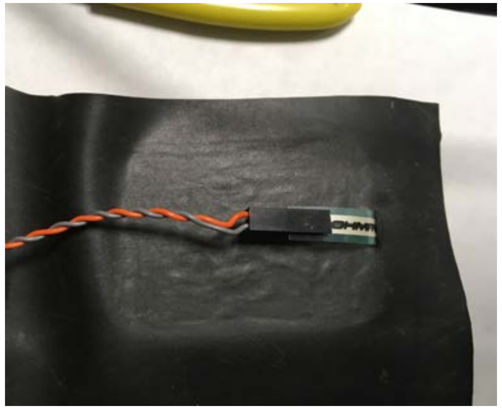

- Cut two wires 180 mm long and strip their ends about 5 mm.
- Solder one end of the twisted pair to keep the strands form fraying.
- Connect the soldered ends to the Respiration Sensor, as shown.

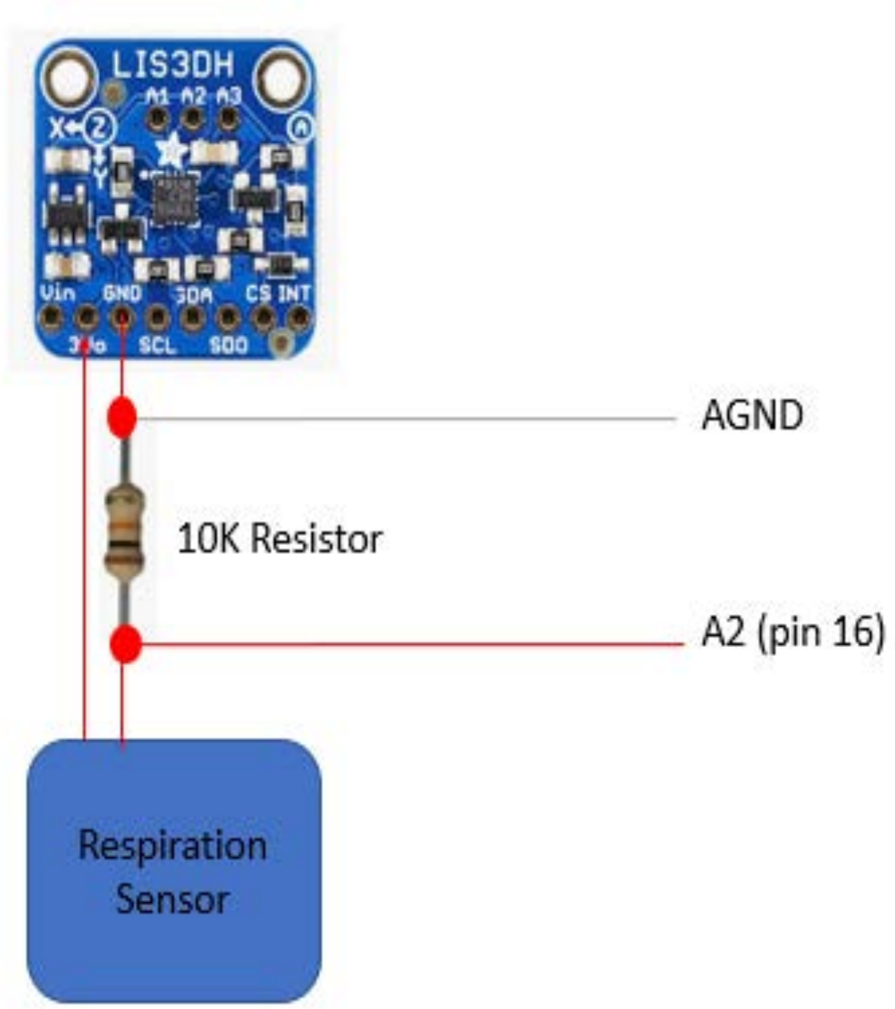

- Connect one of the Respiration Sensor wires to the 3Vo pin of the Accelerometer.
- Connect the other one to the GND via a 10K Ohm resistor.
- Cut two wires 180 mm, twist them and strip the ends 5 mm.
- Connect two wires as shown, one to the Accelerometer's GND and the other to the 10K resistor & Respiration Sensor. These wires will terminate at the Micro-controller's pin shown in the diagram.
- Record the color code for the two wires.

CONNECTING MICRO-CONTROLLER

DEVICE	SIGNAL NAME	TEENSY PIN NO.
	3.3V	3.3V
HEART MONITOR	LO-	7
	LO+	6
	OUTPUT	15 (A1)
ACCELEROMETER	SCL	13
	SDA	11
	SDO	12
	CS	5
	INT	10
BLUETOOTH	TX	1
	RX	0
RESPIRATION SENSOR		16 (A2)

Connection check list

- This table shows where the wires designated for each indicated signal should be terminated (connected to) at the Micro-controller (Teensy).

*** The Heart Monitor 3.3V VCC (power) wire will be connected on the Teensy 3.3V pin. This is because this module doesn't come with a voltage regulator. ***

- Print this list, cross off every connection made.

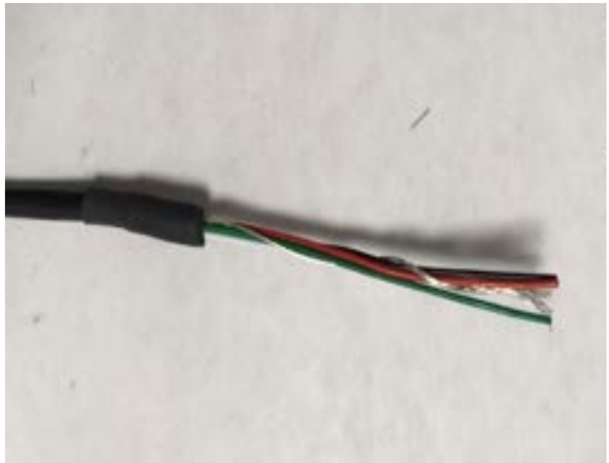

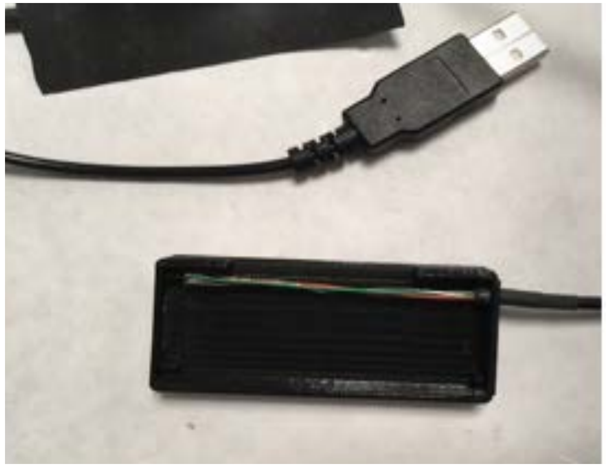

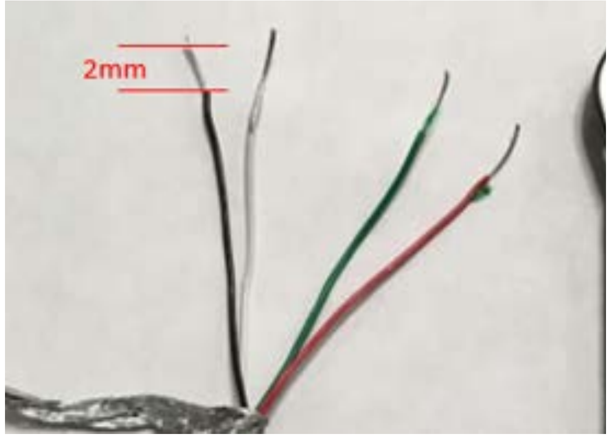

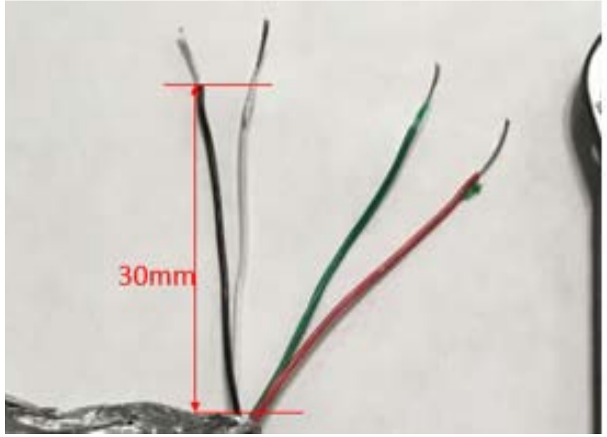

Prep the USB cable

- Strip USB cable about 35 mm long. You will find 4 wires and the shielding (uninsulated wire & aluminum foil).
- Insert a shrink tube to the end if the cable is smaller than the designated hole (4.5 mm diameter) on the casing.
- If the cable diameter is larger than the hole, drill the hole to resize it.
- Insert the cable through the hole as shown.
- Strip the ends of the wires 2 mm long and solder.

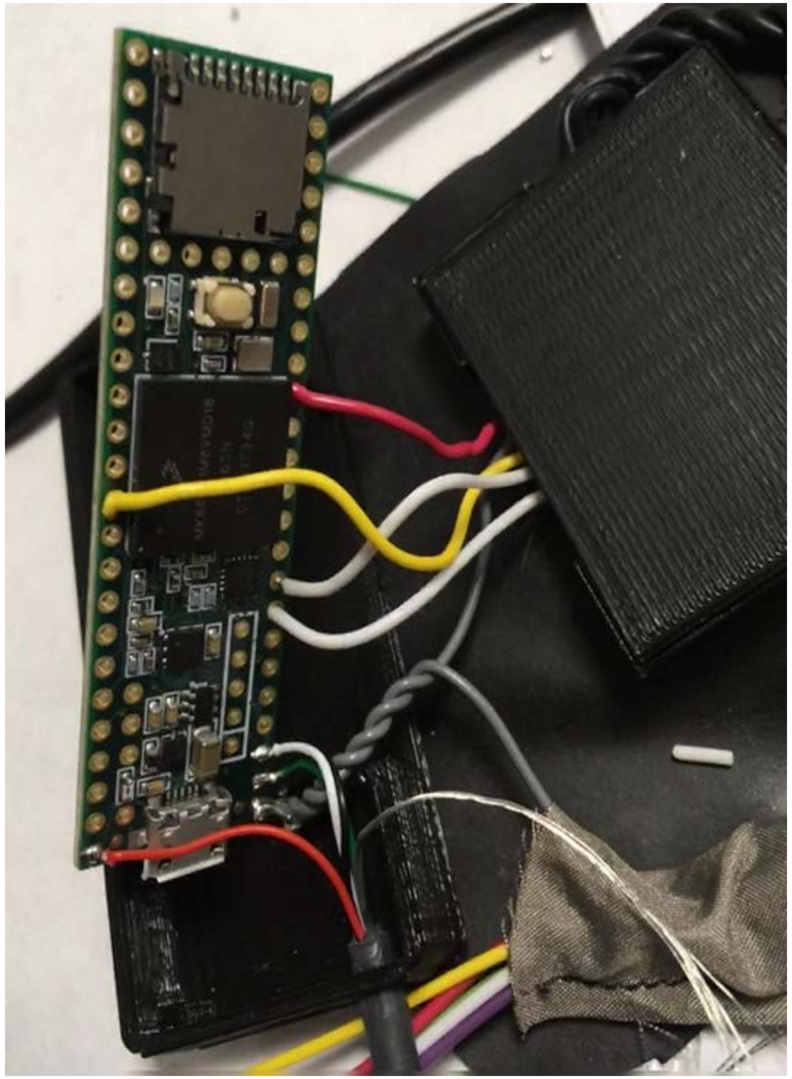

Solder power wires

- Strip the Accelerometer GND wire, connect it to Heart Monitor-GND, and USB GND (black wire) by twisting and soldering them together.

- Solder the connected GND wires to the Micro-controller’s (Teensy 3.6) GND.

- Get the Accelerometer Vin wire, strip it and twist it together with the red wire from USB cable.

- Solder their ends to make them connected and solder the connected ends to the Micro-controller’s Vin.

- Using the Connection Check list, connect the rest of the wires to their respective leads. Be sure to cross out the name of the wire as soon as it has been soldered to the right lead.

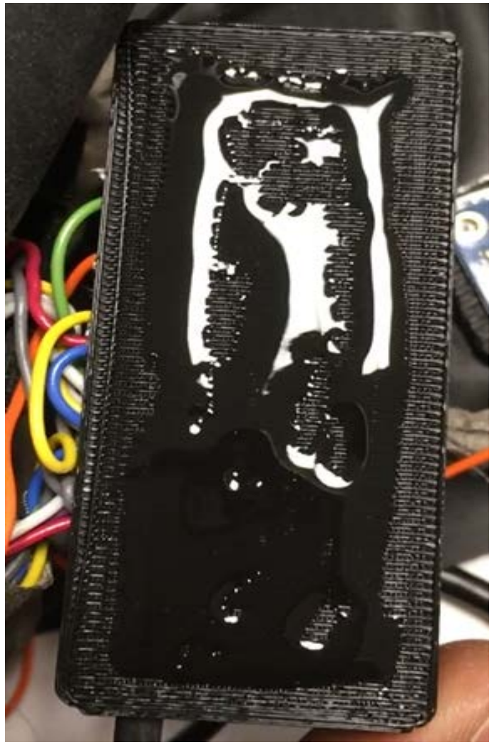

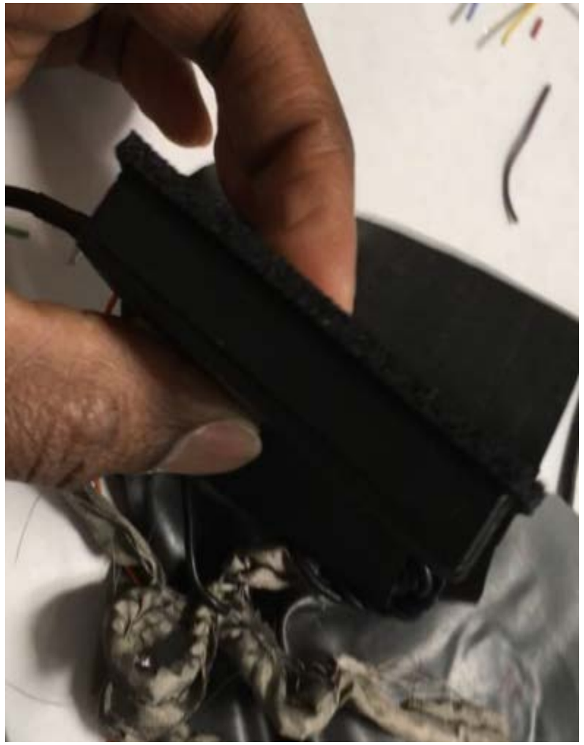

Attach Micro-controller case

- Cut a piece of ¼ thick Neoprene foam pad slightly bigger than the micro-controller's case (70 mm x 35 mm).
- Apply super-glue on the case as shown.
- Stick the pad onto the case with the pad edges overlapping the case edges.
- Press and hold firmly for the glue to set.

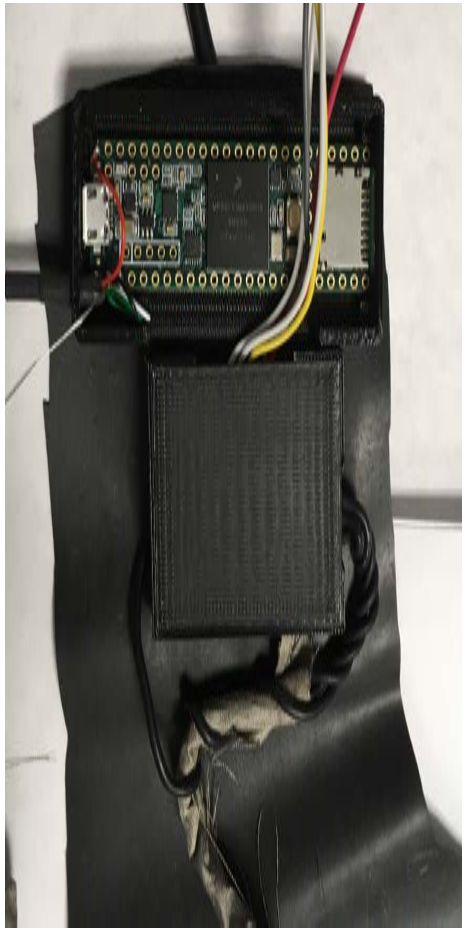

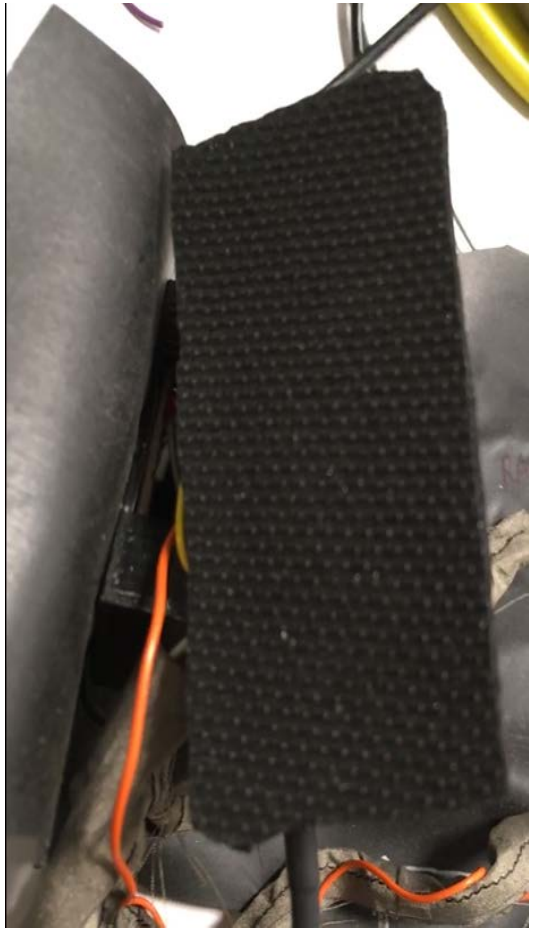

- Using a hacksaw blade, roughen the surface where the Micro-controller Case is going to be glued (between vest's left edge and the Heart Monitor's left side wall).

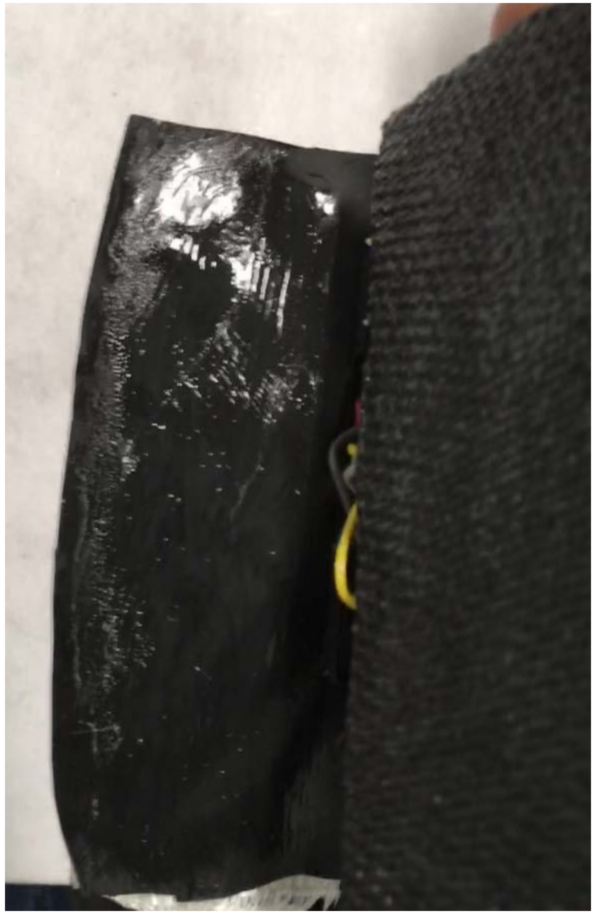

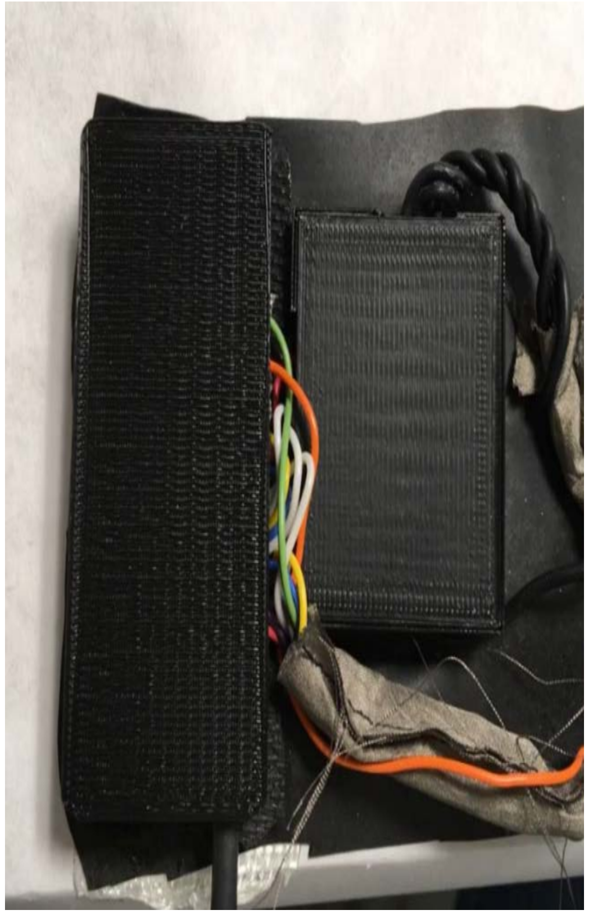

- Apply rubber cement on the roughened surface and the pad stuck on the case.
- Wait until the rubber cement is tacky.
- On a flat surface, stick the pad onto the rubber sheet.
- Press firmly to make a good joint.

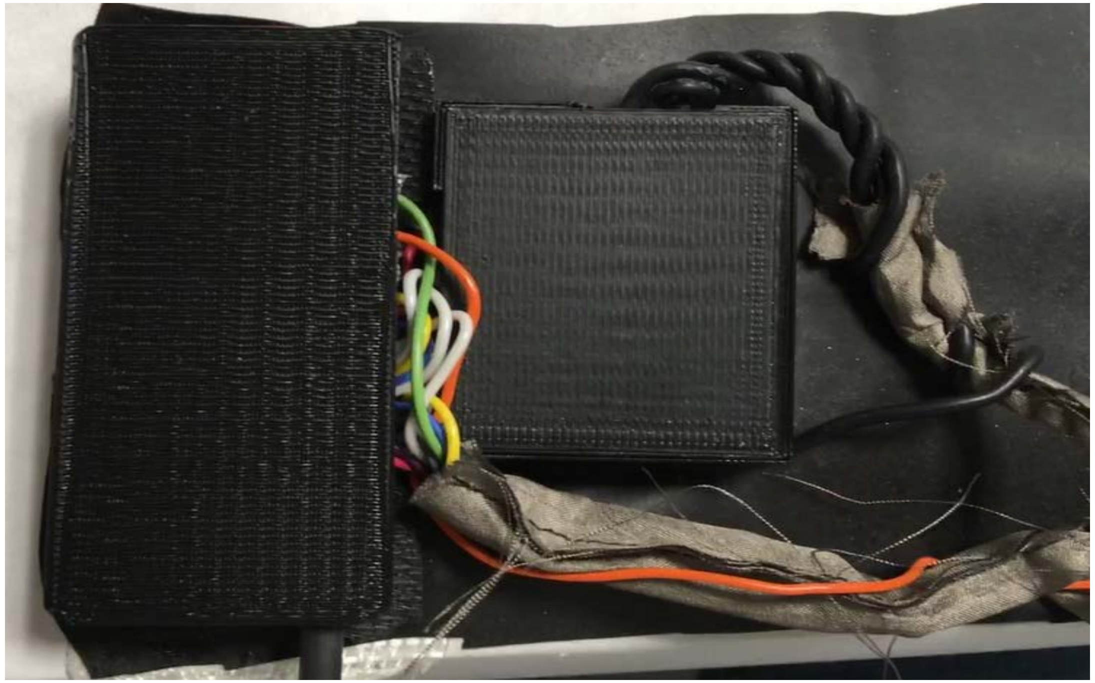

- Now connect the remaining wires to their respective pins/leads. Make sure you check and cross off the list.

DEVICE	SIGNAL NAME	TEENSY PIN NO.
	~~3.3V~~	~~3.3V~~
HEART MONITOR	~~LO~~	~~7~~
	~~LO+~~	~~6~~
	~~OUTPUT~~	~~15 (A1)~~
	~~SDN~~	~~8~~
ACCELEROMETER	~~SCL~~	~~13~~
	~~SDA~~	~~11~~
	~~SDO~~	~~12~~
	~~CS~~	~~5~~
	~~INT~~	~~9~~
BLUETOOTH	~~TX~~	~~1~~
	~~RX~~	~~0~~
RESPIRATION SENSOR		~~16 (A2)~~

*** Use cold-shrink tape/ electrical tape/ insulating paint to insulate any connections with bare wire or solder showing. ***

TESTING

Before sealing the components, carry out a test to ensure that all components are working as they are supposed to.

In order to carry out this test, the microcontroller must be programmed as if it was complete and ready for deployment.

All the procedures for programming the Autonomic Vest can be found in the support section.

Don’t forget to insert the SD card before starting the Autonomic Vest for testing and connecting the Autonomic Vest dongle to the computer.

When the program is running, watch for all variables presented on the Graphical User Interface (GUI) to see any changes when the electrodes are touched with bare hands, when the vest it moved and when Respiration Sensor is pressed.

A good test for the Heart Monitor will be touching the left electrode base with any left-hand finger, the right one with any right-hand finger and the far-right electrode (virtual ground) with any finger. Observe the screen for the heartbeat pulse.

If there is a problem, check the connections against the connection list for possible miswiring or loose connections.

SEALING THE COMPONENTS

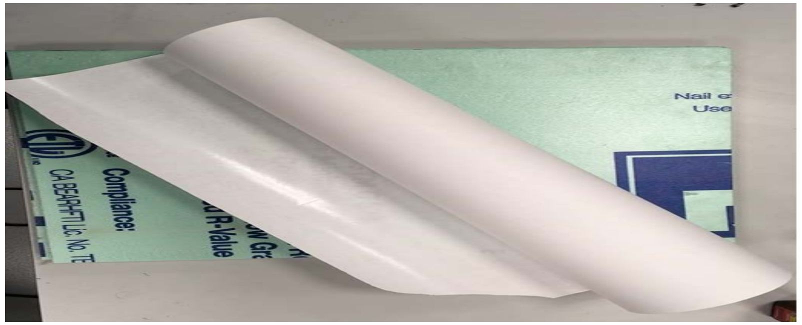

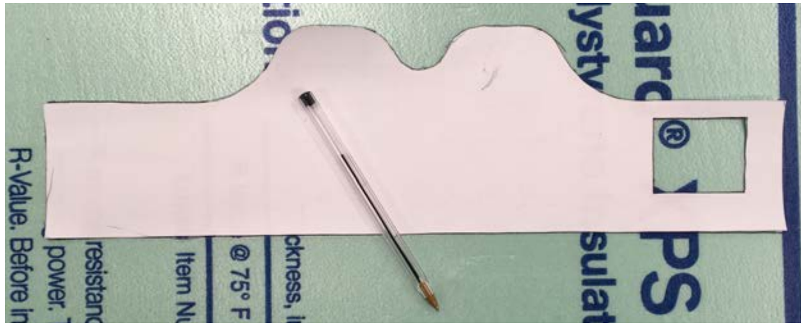

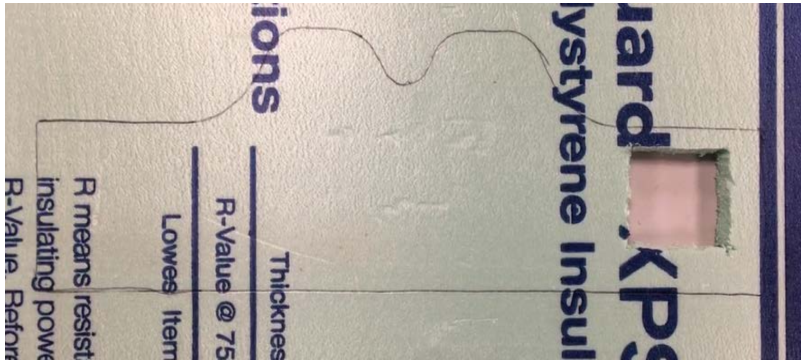

Prep the foam board

- Acquire a piece of plywood/ construction foam-board/ any rigid but easy to cut material measuring at least 500 mm x 200 mm x 10 mm.

- Use the rubber sheet template to draw the outline of the vest and the Respiration Sensor hole.

- Enlarge the Respiration Area on the board to be 60 mm x 50 mm (length x height).

- Cut out the respiration area.

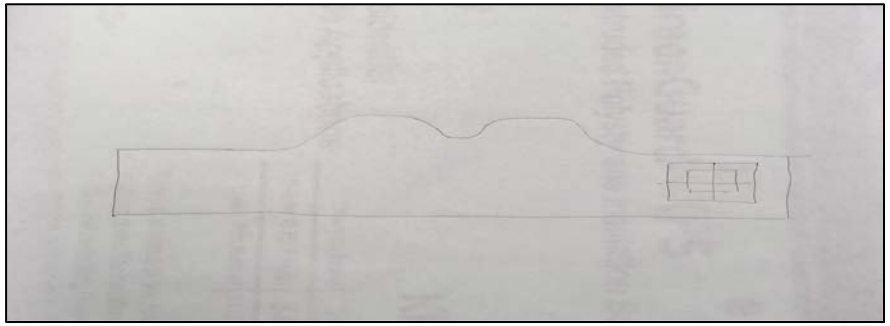

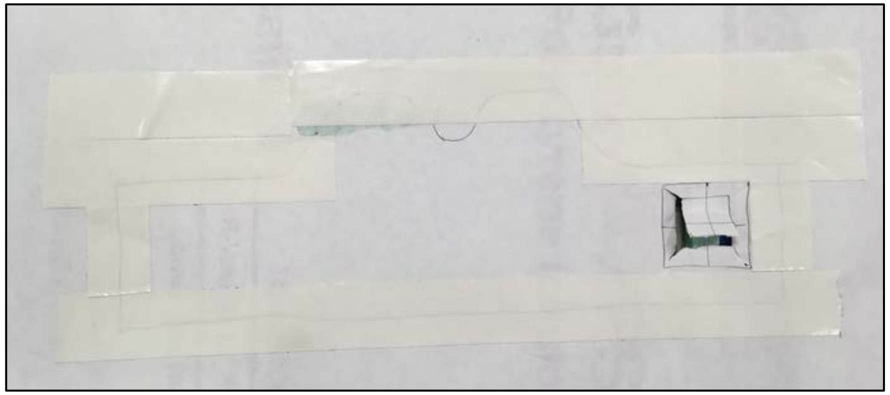

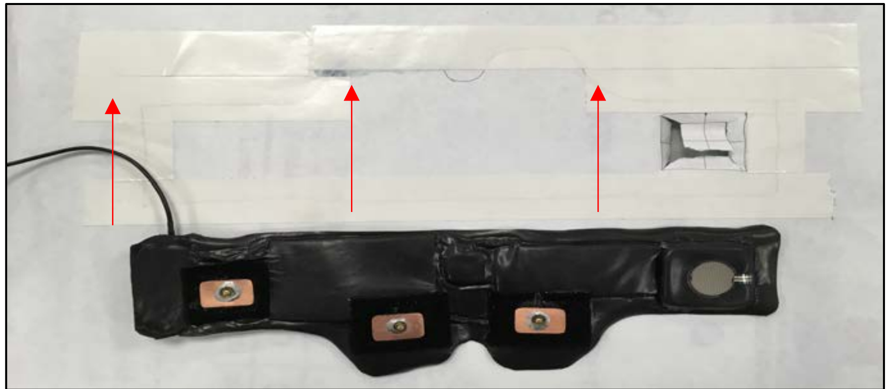

- Stick a piece of paper on the board using tape and redraw the outline now on the paper.
- Cut the respiration hole.
- Put carpet tape along the outline as shown in the second picture.
- Ensure that the Respiration Sensor fits in the hole.
- Peel the carpet tape and lay the vest with the sensor side facing down.

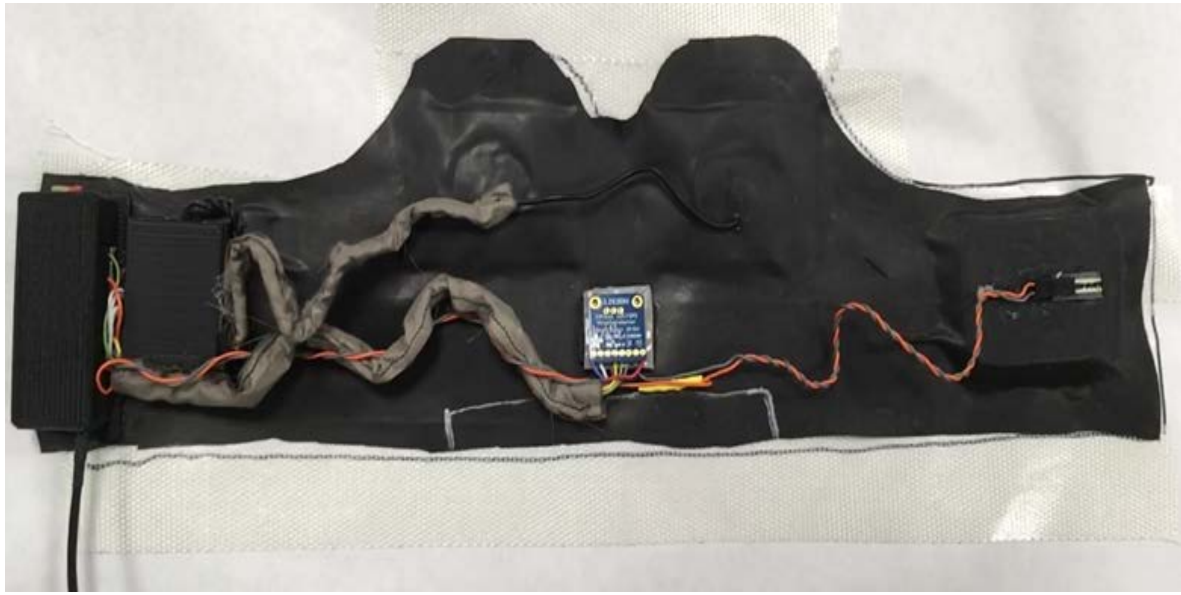

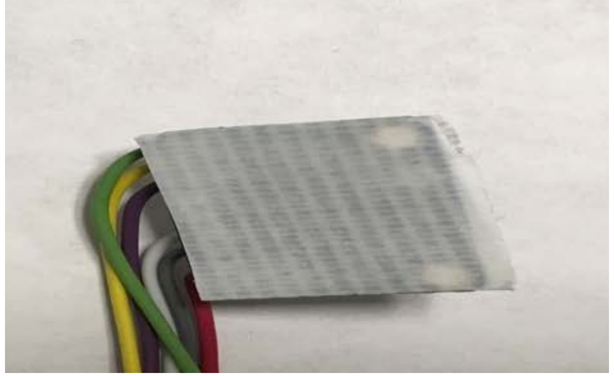

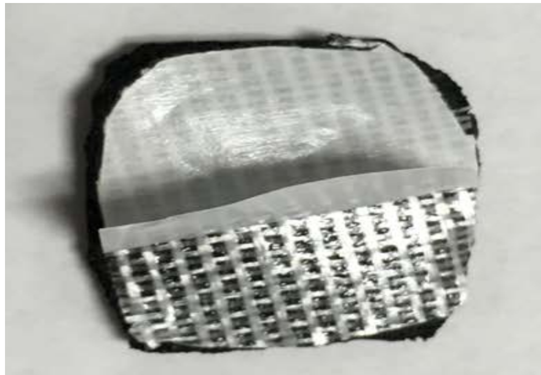

Lay the vest on the foam board

- Make sure the Respiration Sensor lays inside the hole and the rest of the vest lays flush to the surface.
- Using hands, press the vest to stick well onto the carpet tape.
- Using fingers, ensure that the vest sticks to the carpet tape flat (without slack or creases).
- Attach the Accelerometer to the rubber sheet using carpet tape and a piece Neoprene rubber.

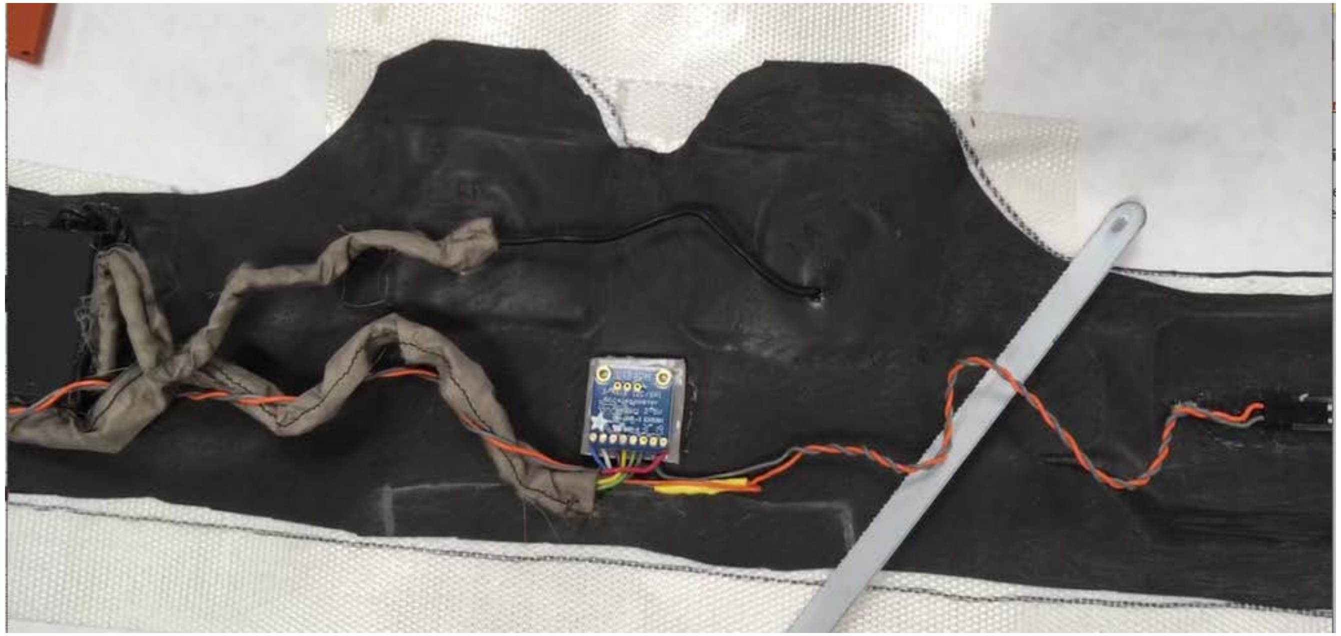

Prep the vest surface

- Using a hacksaw blade angled as shown, carefully roughen the surface of the rubber by moving the blade back and forth in a slanted direction.

- Do not touch or wipe the roughened surface.

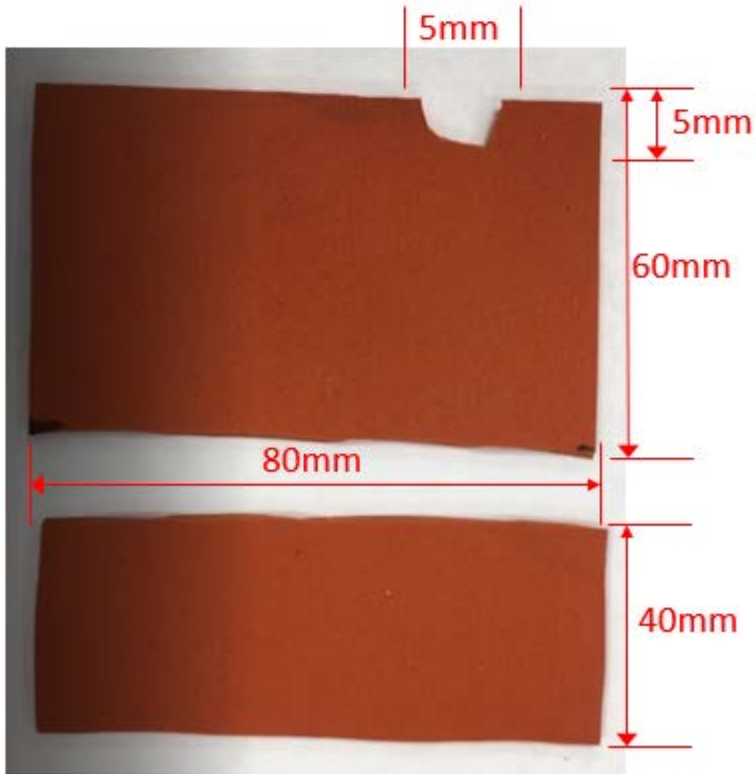

Padding for the vest

- Measure and cut two pieces of thin rubber as shown.

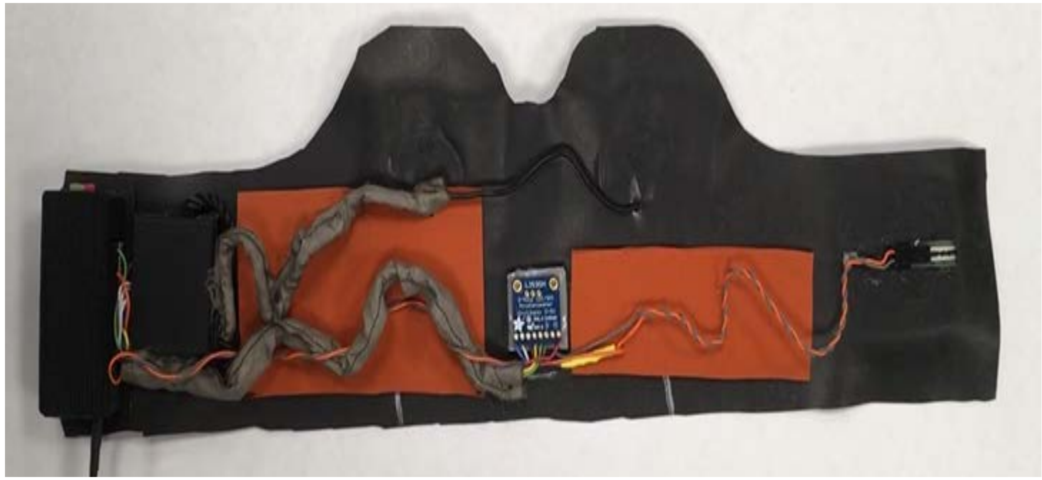

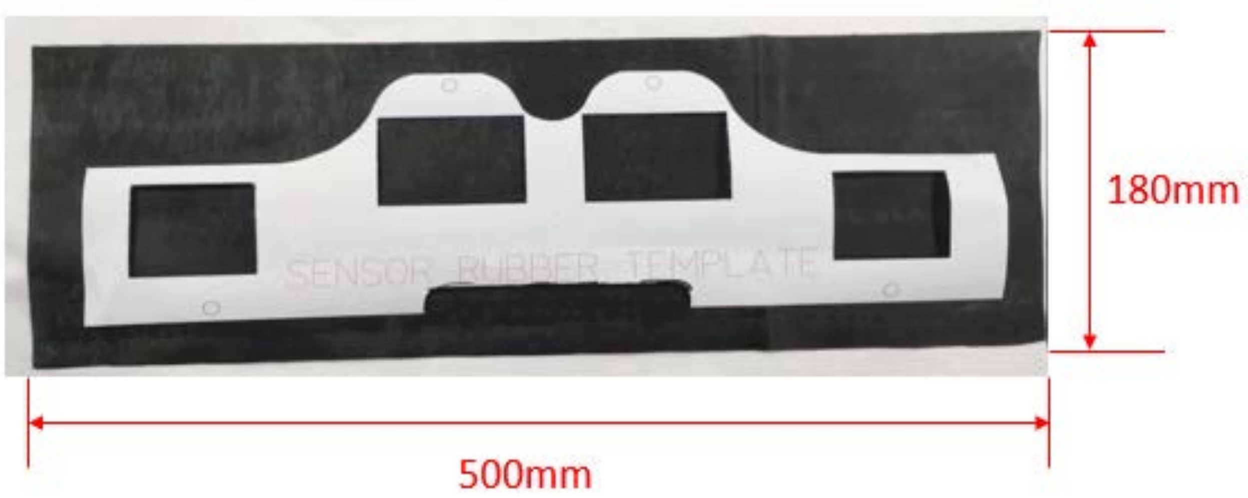

Cut & prep the front rubber sheet

- Secure a 500 mm x 180 mm rubber sheet on a flat surface using carpet tape.
- Carefully roughen the rubber sheet with the hacksaw.

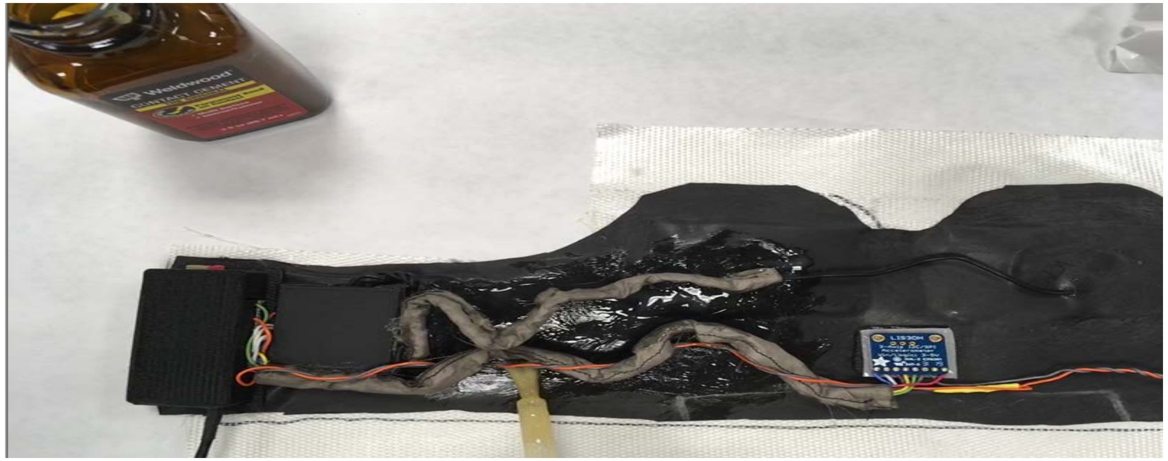

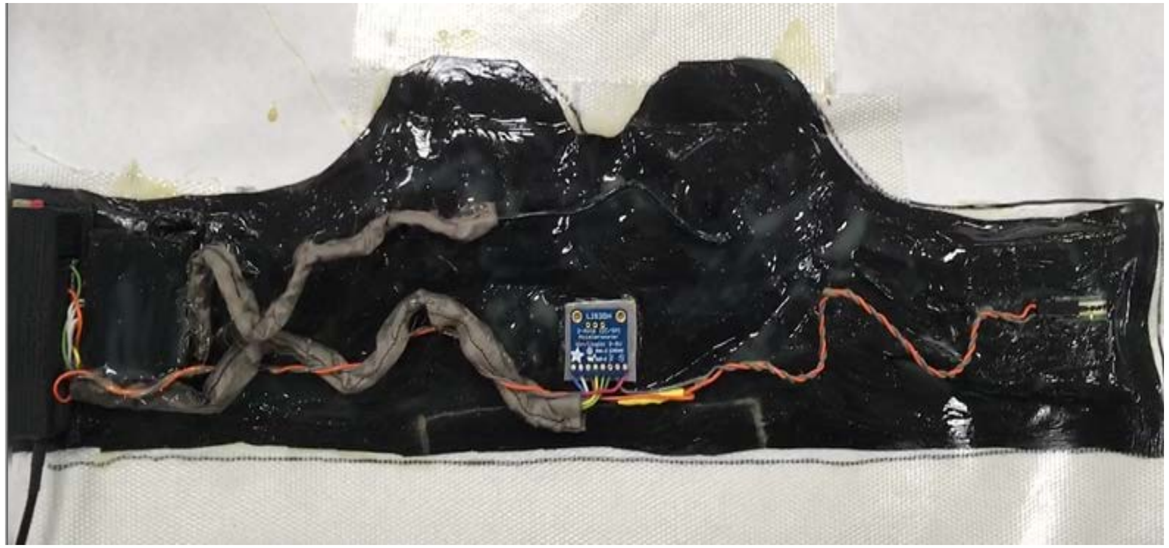

- Apply rubber cement to the prepared surfaces using the cement applicator.
- Also apply rubber cement on the Heart Monitor case.
- Apply rubber cement on the other rubber sheet (the front piece).

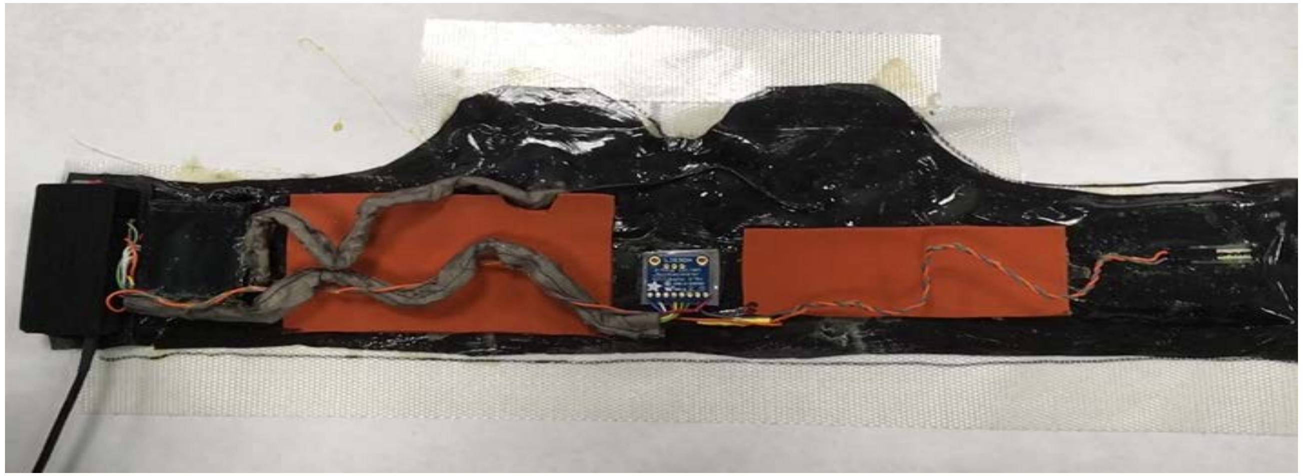

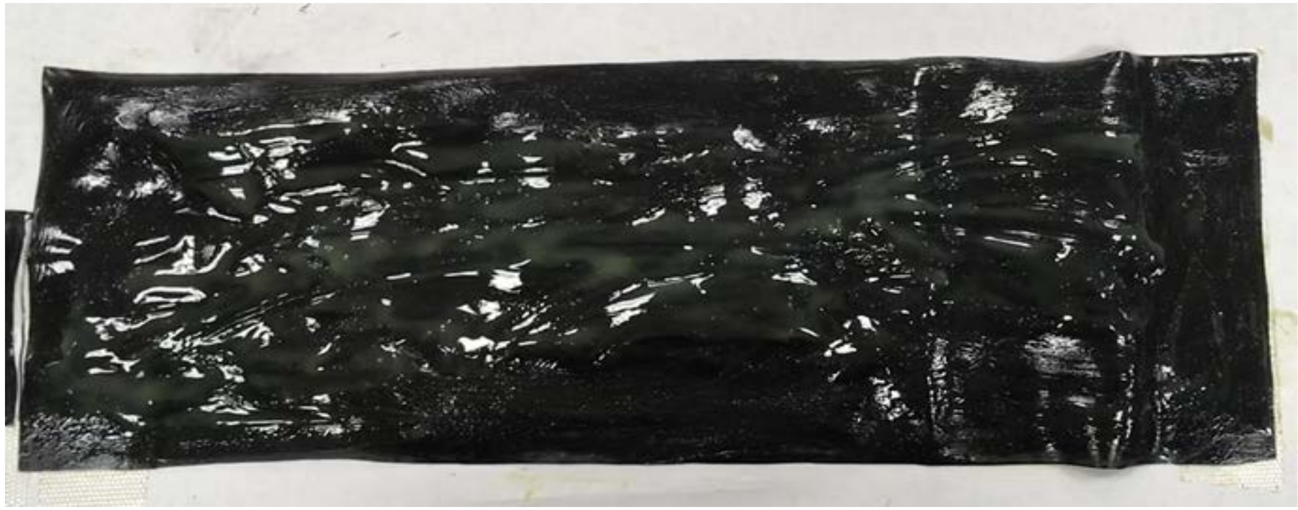

- Wait for the rubber cement to become tacky before sticking anything to it.
- Once the rubber cement is tacky, stick on the padding as shown.
- From this step at least two people are required to proceed with the project.
- Lift the rectangular rubber sheet off the carpet tape gently, without touching the glue part 10 mm from the edges.
- Stick it to the other sheet with the sensors, starting from one end and moving slowly towards the other while pressing the two sheets where they come in contact.

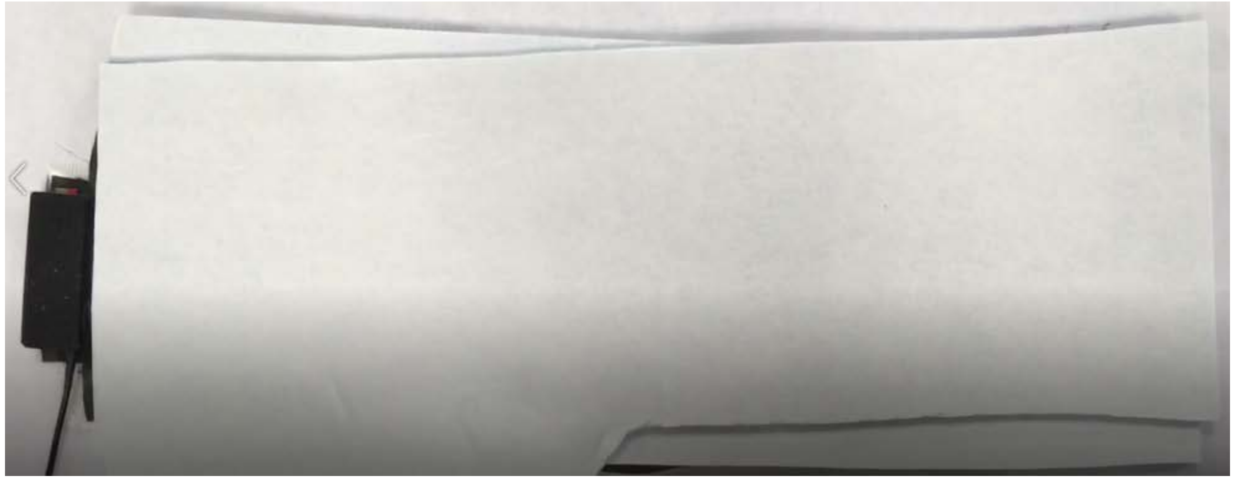

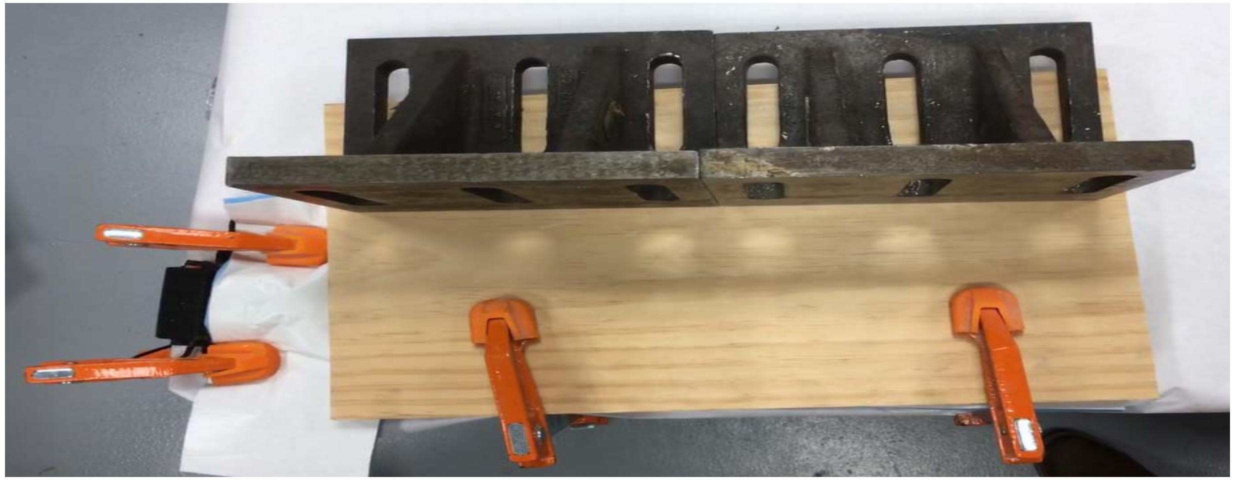

Clamping

- Cut two foam pieces measuring about 550 mm x 200 mm.
- Stack them on top of the vest without covering the Micro-controller case.
- Put a piece of wood measuring at least 550 mm x 200 mm on the foam pieces .
- Clamp the wooden piece as close to the center as possible. If the clamps won’t reach the center, use weights to press the side further from the clamps.
- Wait at least 72 hours before unclamping.

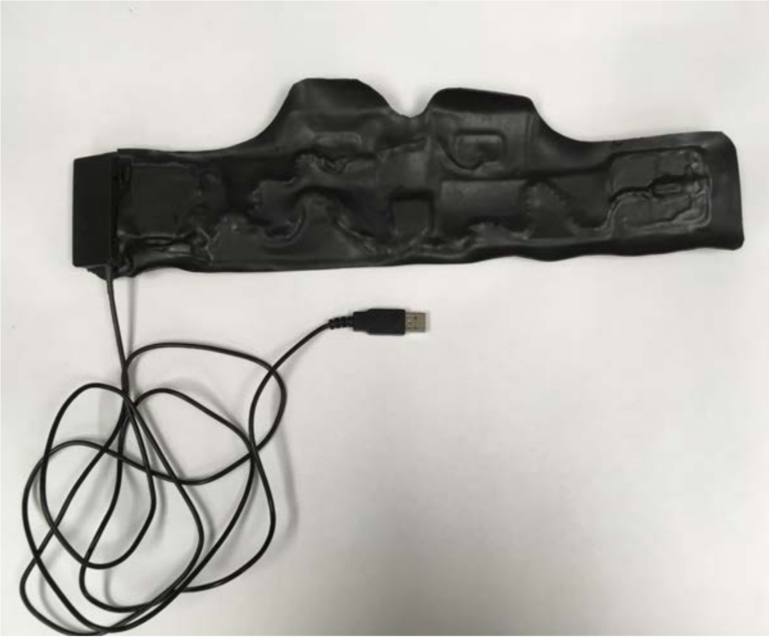

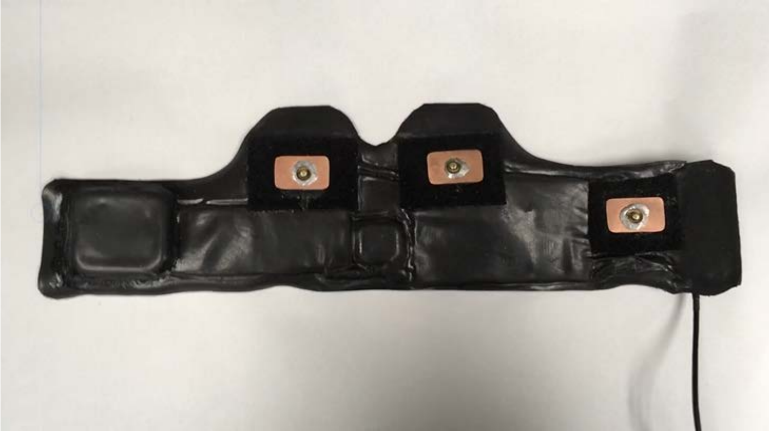

- After at least 72 hrs, unclamp the vest.
- Trim the vest right above where the two rubber sheets are joined.
- Clean the rubber cement and carpet tape adhesive using dish soap and water while protecting the Micro-Controller.
- Use olive oil or silicon rubber oil to further clean the rubber and restore its color.

ADDING MORE SENSORS

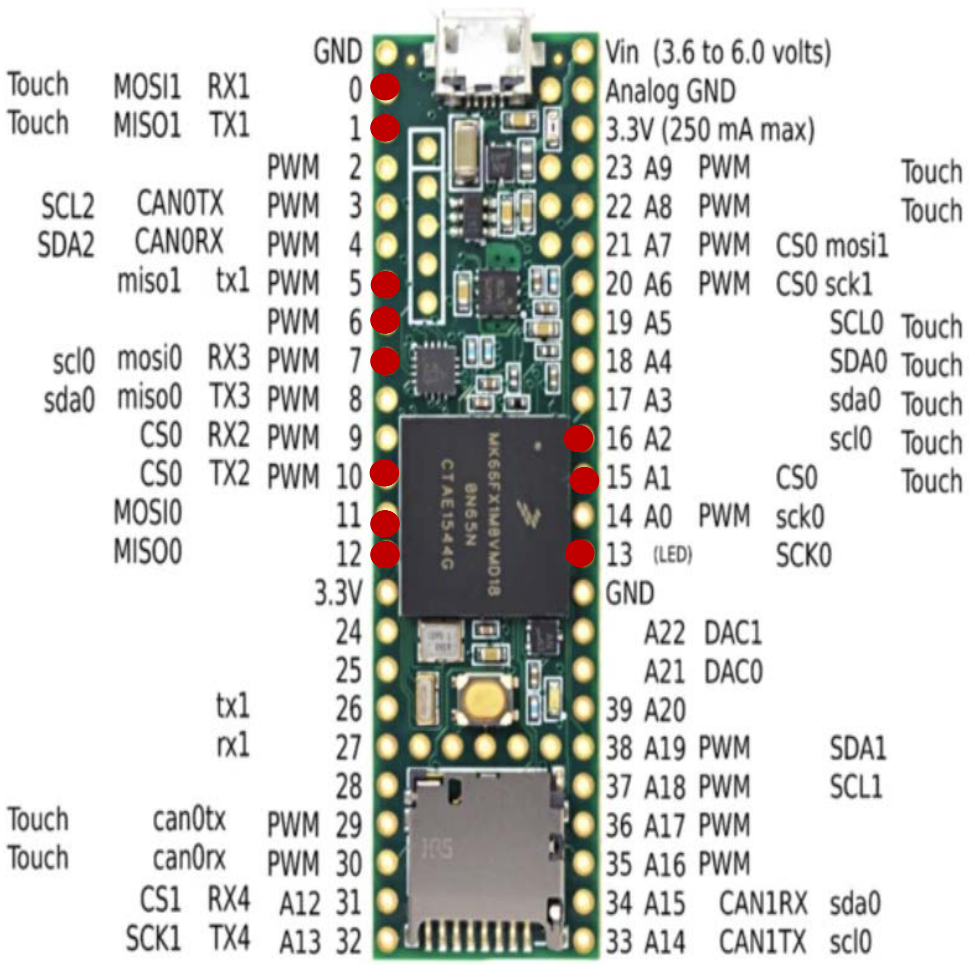

The Micro-Controller used in this project is Teensy 3.6.
It has 62 Input/output pins in total, but the marked pins are pins that are currently being used by the Autonomic Vest.

Teensy 3.6 has 3 SPI Communication Ports in total. SPI communication ports can be shared by multiple devices, so any other SPI-device added onto the vest can use these pins; 13-SCL (Clock), 12-MISO 0 (Data IN), and 11-MOSI 0 (Data Out), where zero 0 denotes the SPI module/port 0.

The module SPI 1 (Pin 0-MOSI 1 , Pin1-MISO 1) is used for Bluetooth communication as a software serial and therefore cannot be shared.

There are four I2C ports in this microcontroller but port 0 is not available since pin 7 is used for Leads Off detection in the Heart Monitor.

ADDING MORE SENSORS

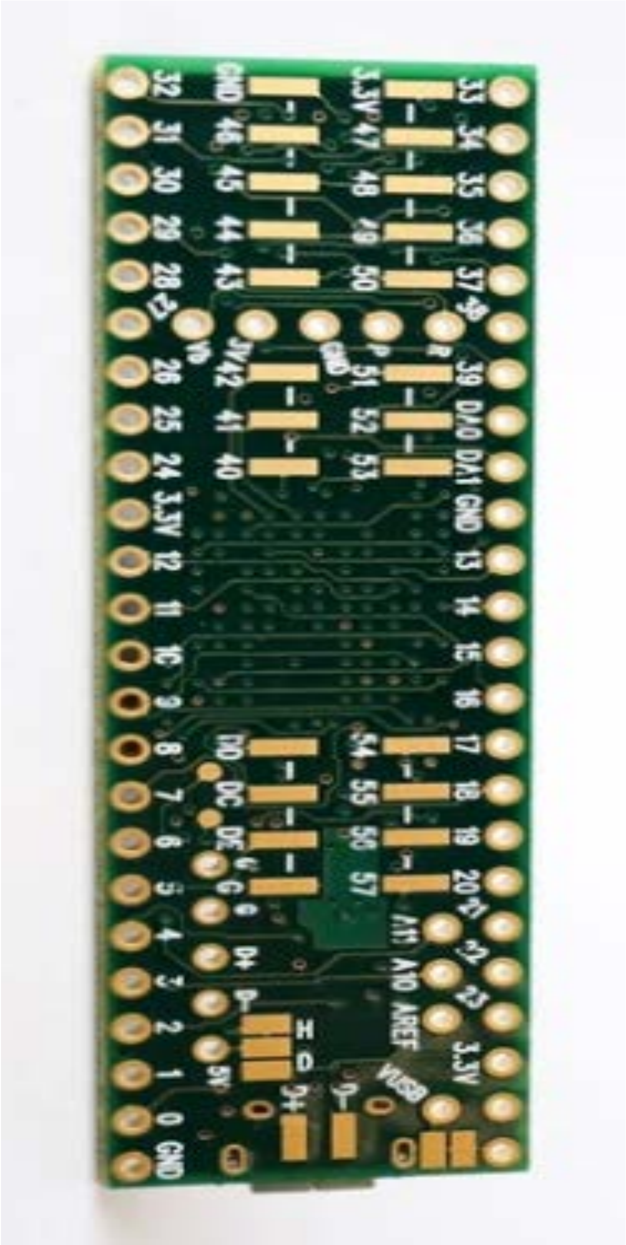

There are more pins at the back of the Micro-controller. Most of the pins can be configured as analog pins or digital pins for both Input/output tasks. All the pins can only handle 3.3V volts, so logic level circuits will be needed for 5V devices.

The pin configurations that were use for the Autonomic Vest can be moved to other pins with the same Input/output functionality if their position prevents using any Micro-controller peripheral, but the firmware must be re-written to accommodate the changes.

More information pertaining to usage of this Micro-controller can be found on https://www.pjrc.com.

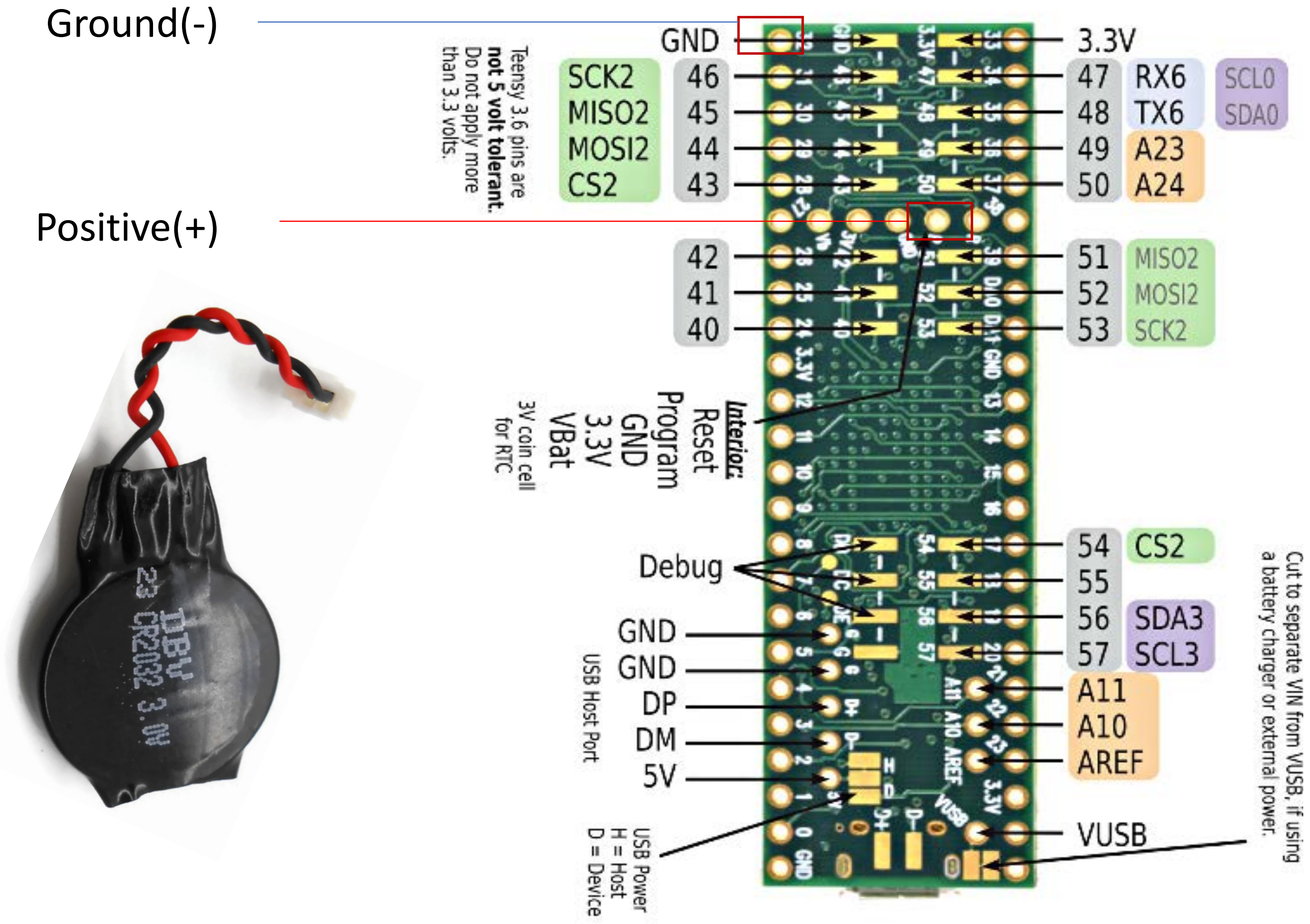

Real time clock (RTC) battery

If anyone is interested in being able to have the clock synchronized with PC time for use in time and date information, a Real Time Clock (RTC) battery will be needed to keep the RTC running even when the Micro-controller is powered off.

RTC clock battery is a 3.0V battery and can be bought from Amazon, Digikey, and various other vendors.

Connect it to the Micro-controller strip Positive lead (red) and solder it to the VBat pin shown (on the back of Teensy 3.6), then strip Ground (black) and solder it to GND.

PROGRAMMING THE MICROCONTROLLER

Download and install the latest Arduino software: http://www.Arduino.cc/en/main/software

- For Windows devices, install the software as an administrator.
- During the installation procedure, install all associated components.

Download and install the Teensyduino support package as an administrator:

https://www.pjrc.com/teensy/teensyduino.html

- Install Teensyduino in the same program path as the Arduino IDE.
- During the installation procedure, install all associated libraries.

UPLOADING THE AUTONOMIC VEST FIRMWARE

- Download the firmware from the Open Science Framework (osf.io/24gp5).
- Extract all contents to the destination of your choice.
- Copy the firmware folder and paste it in the Arduino folder located in Documents.
- The Arduino IDE project is named ‘AutonomicVestFW.ino’. Open it in the Arduino IDE program.

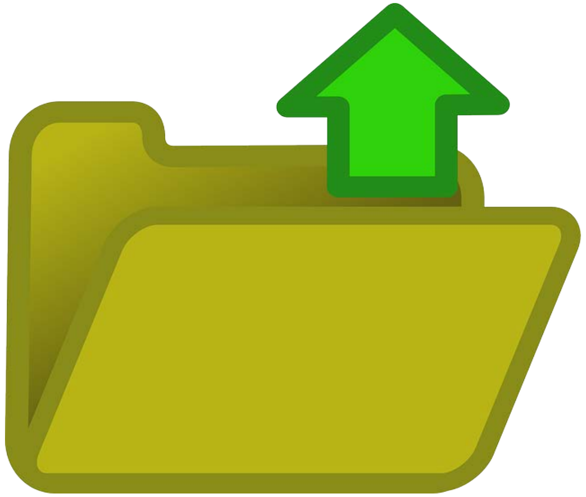

SETTING UP COM PORT & BOARD CONFIGURATION

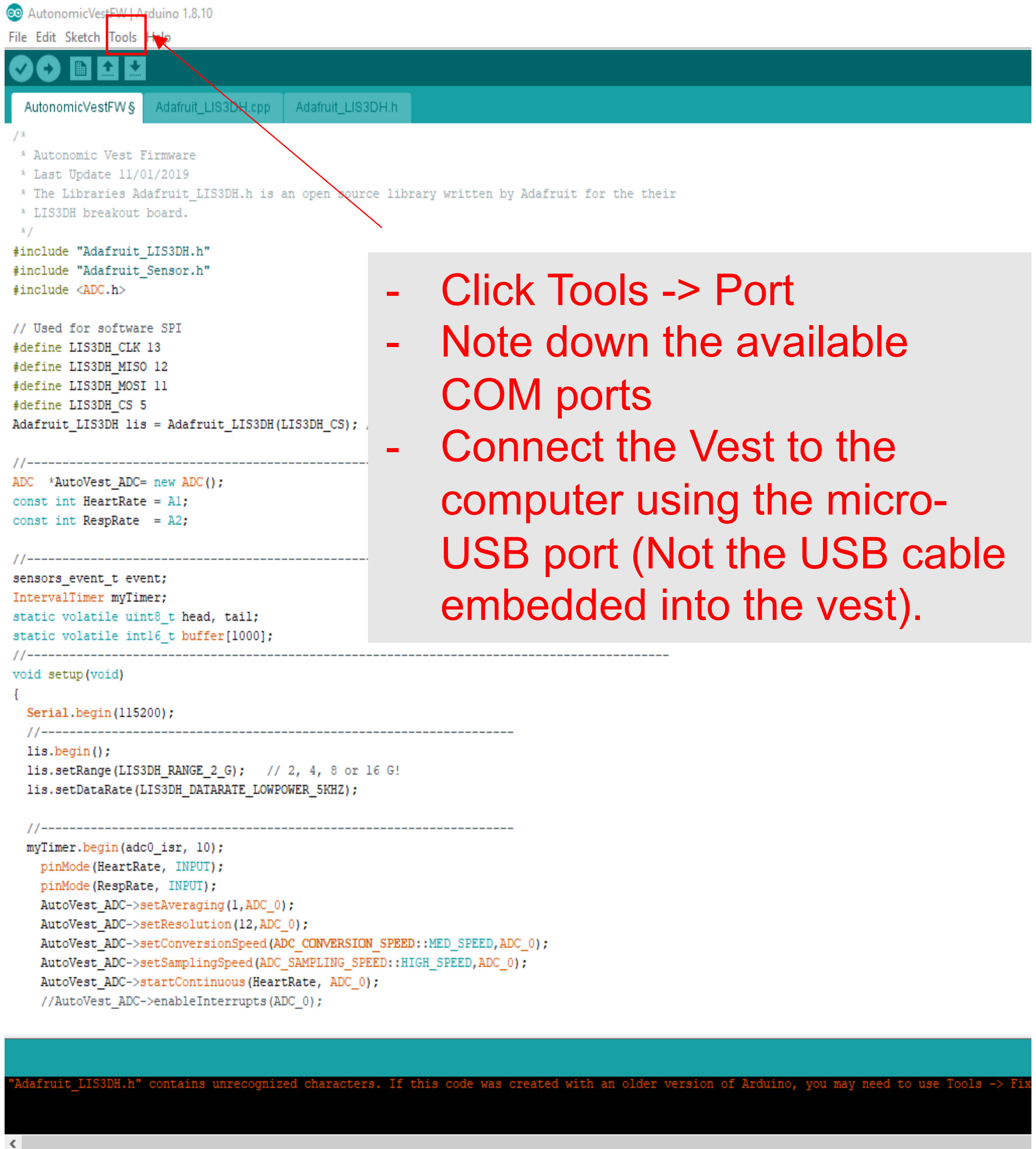

- Click Tools -> Port
- Note down the available COM ports
- Connect the Vest to the computer using the micro-USB port (Not the USB cable embedded into the vest).

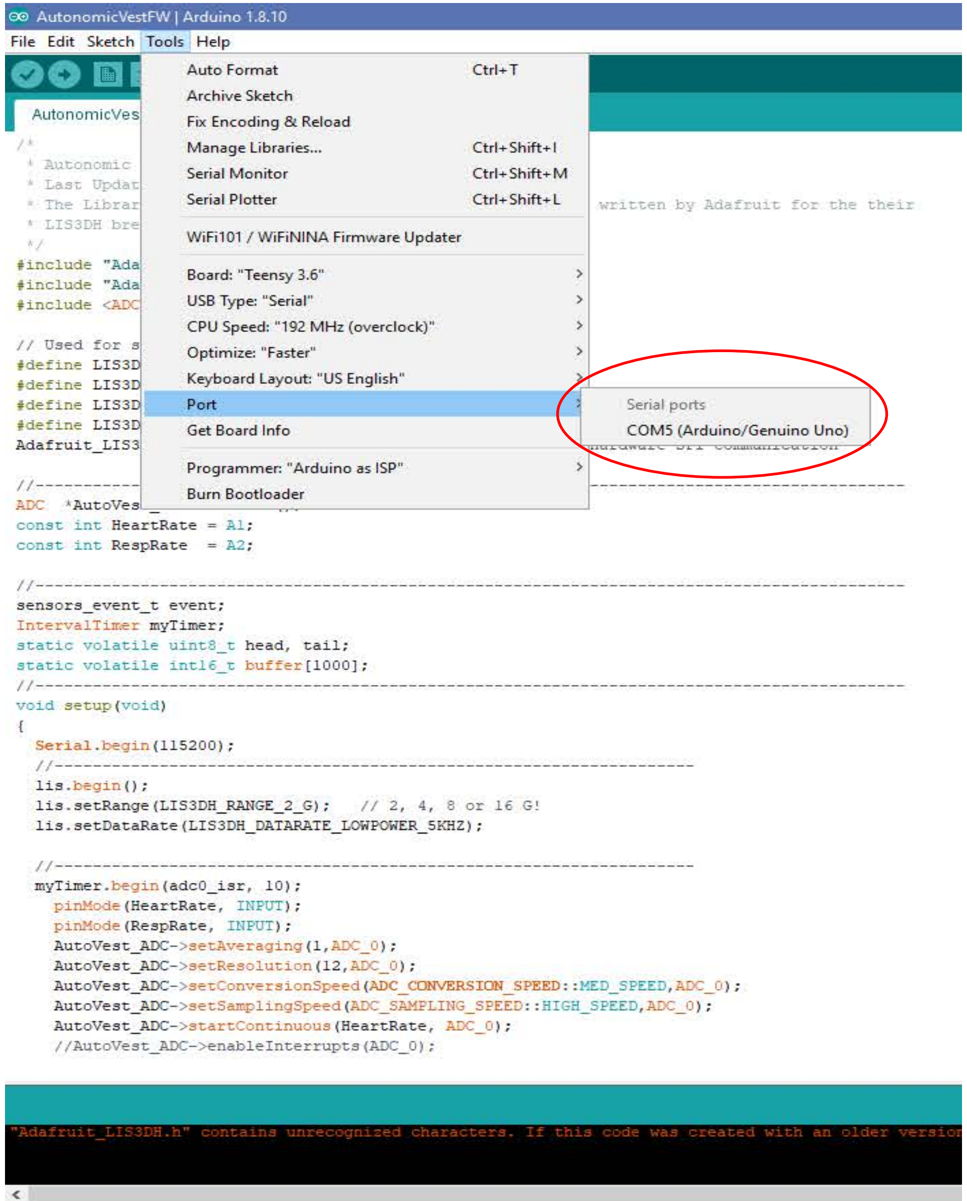

SETTING UP COM PORT & BOARD CONFIGURATION

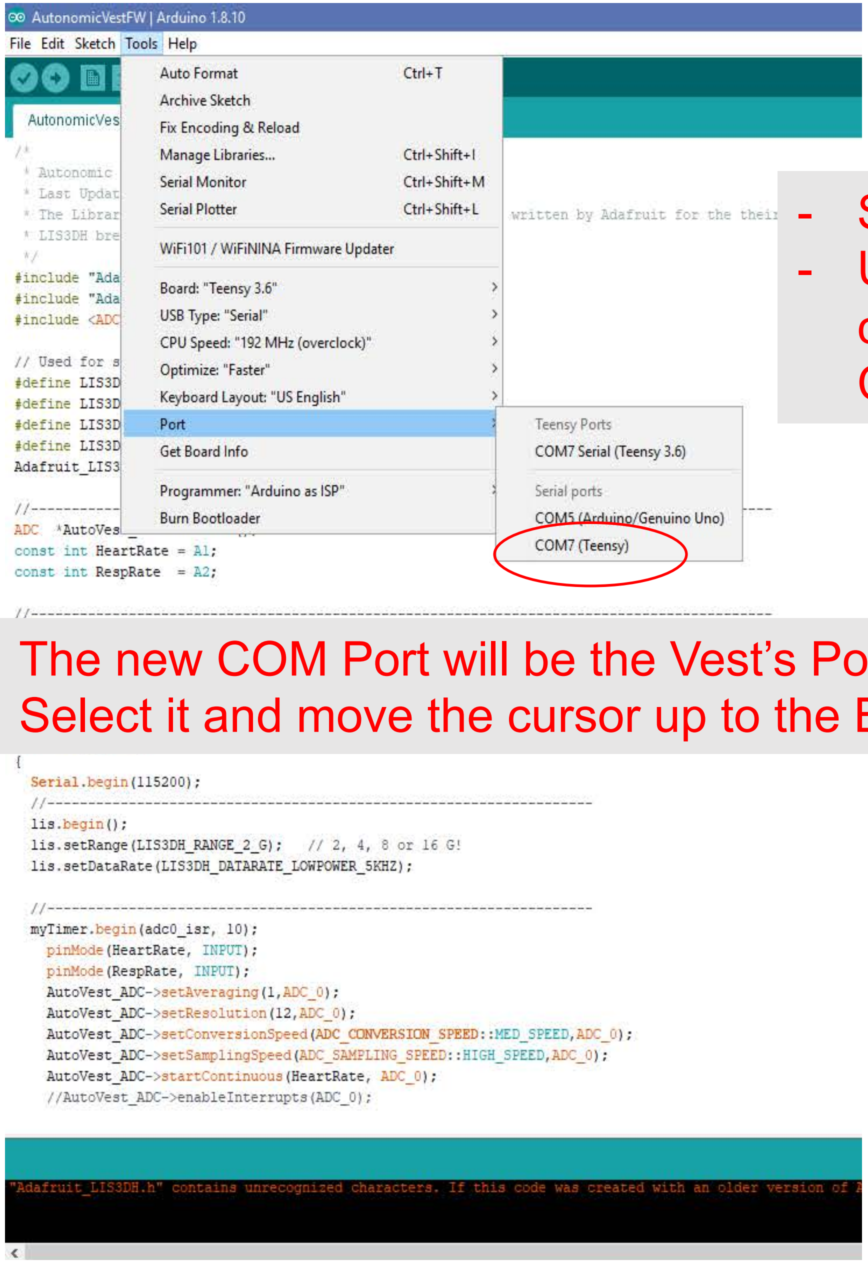

- Select Teensy 3.6
- Using the same procedure for choosing Board type, select CPU Speed to be 180 MHZ.

- The new COM Port will be the Vest's Port.
- Select it and move the cursor up to the Boards.

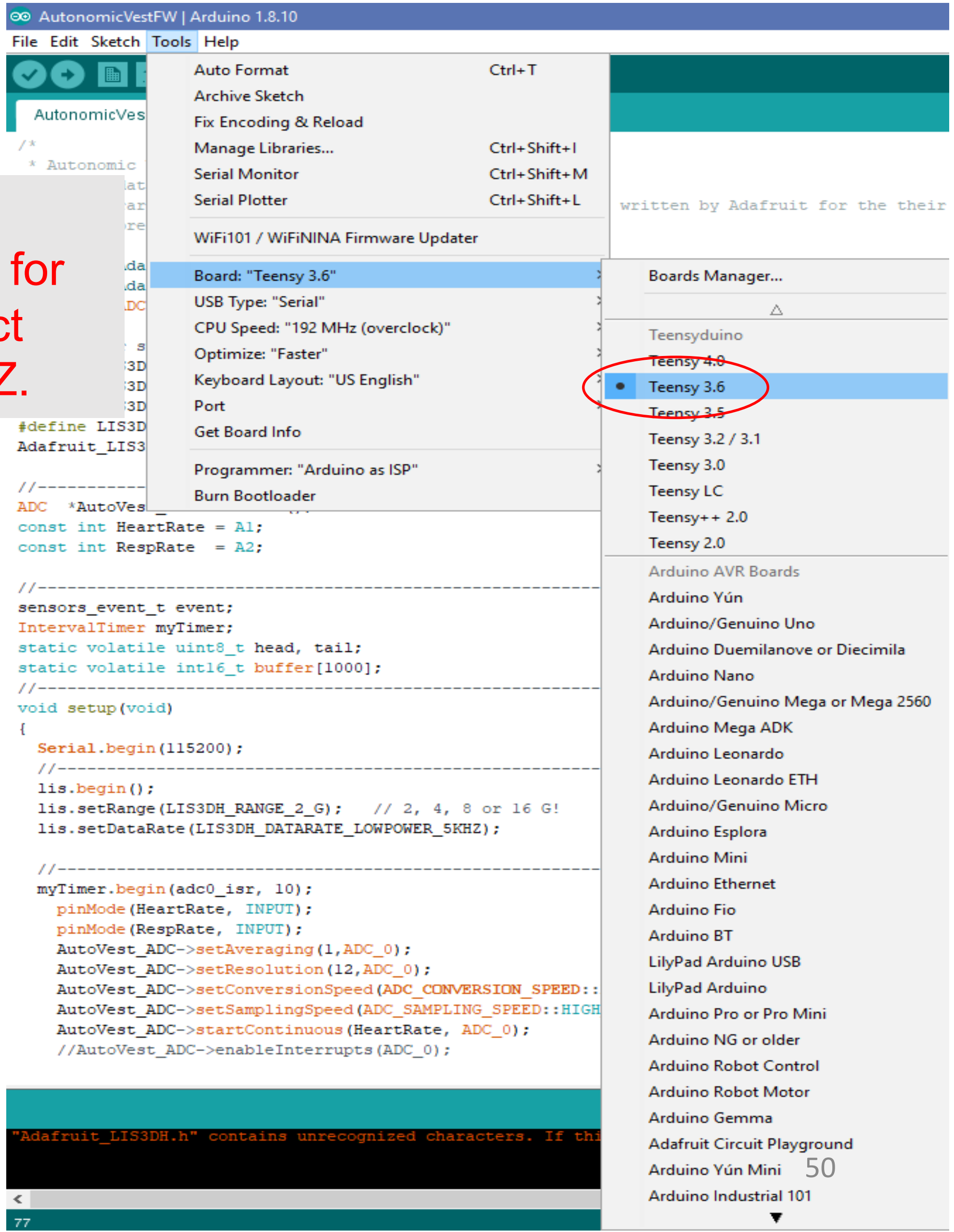

UPLOADING THE FIRMWARE

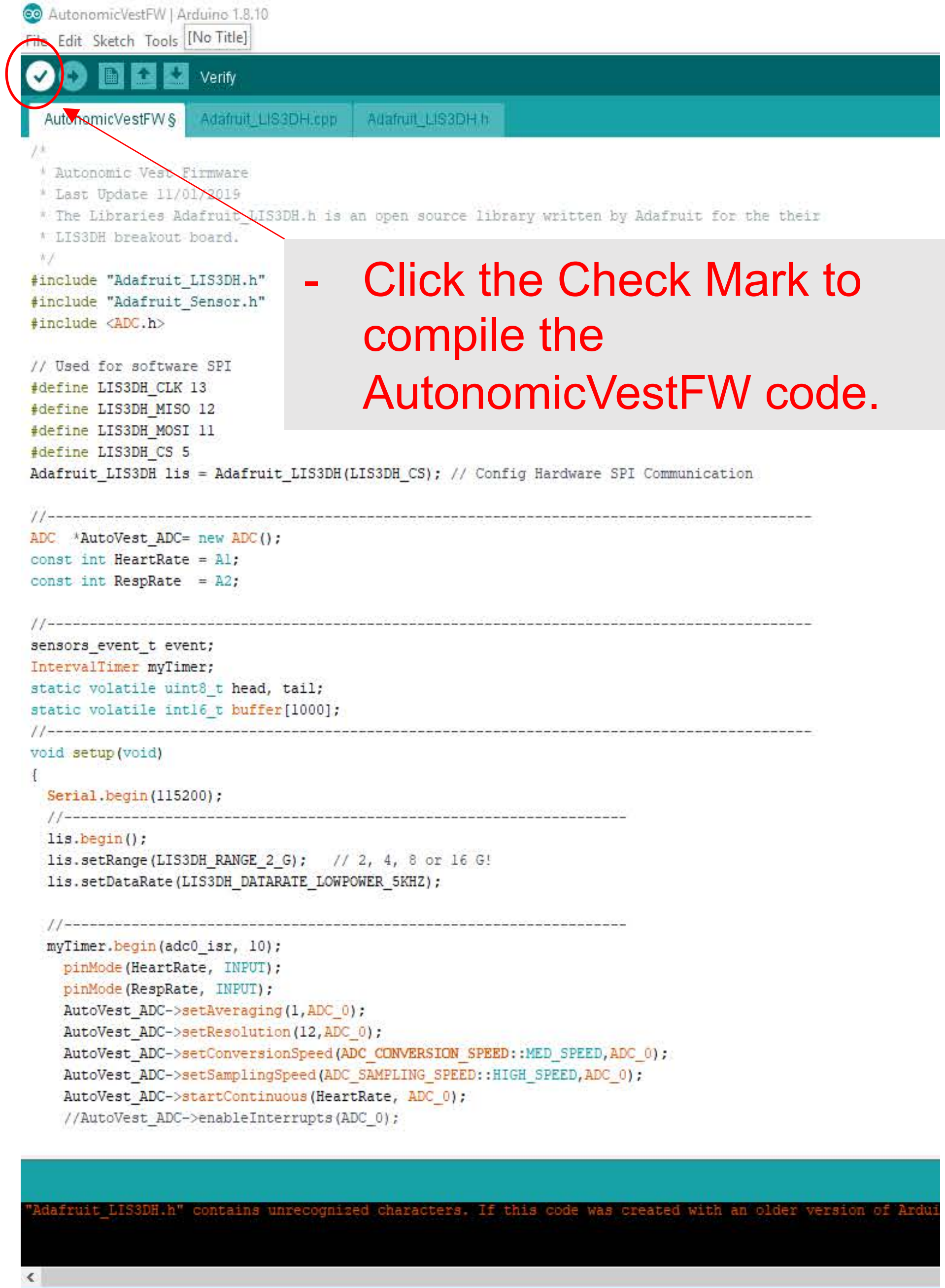

- Click the Check Mark to compile the AutonomicVestFW code.

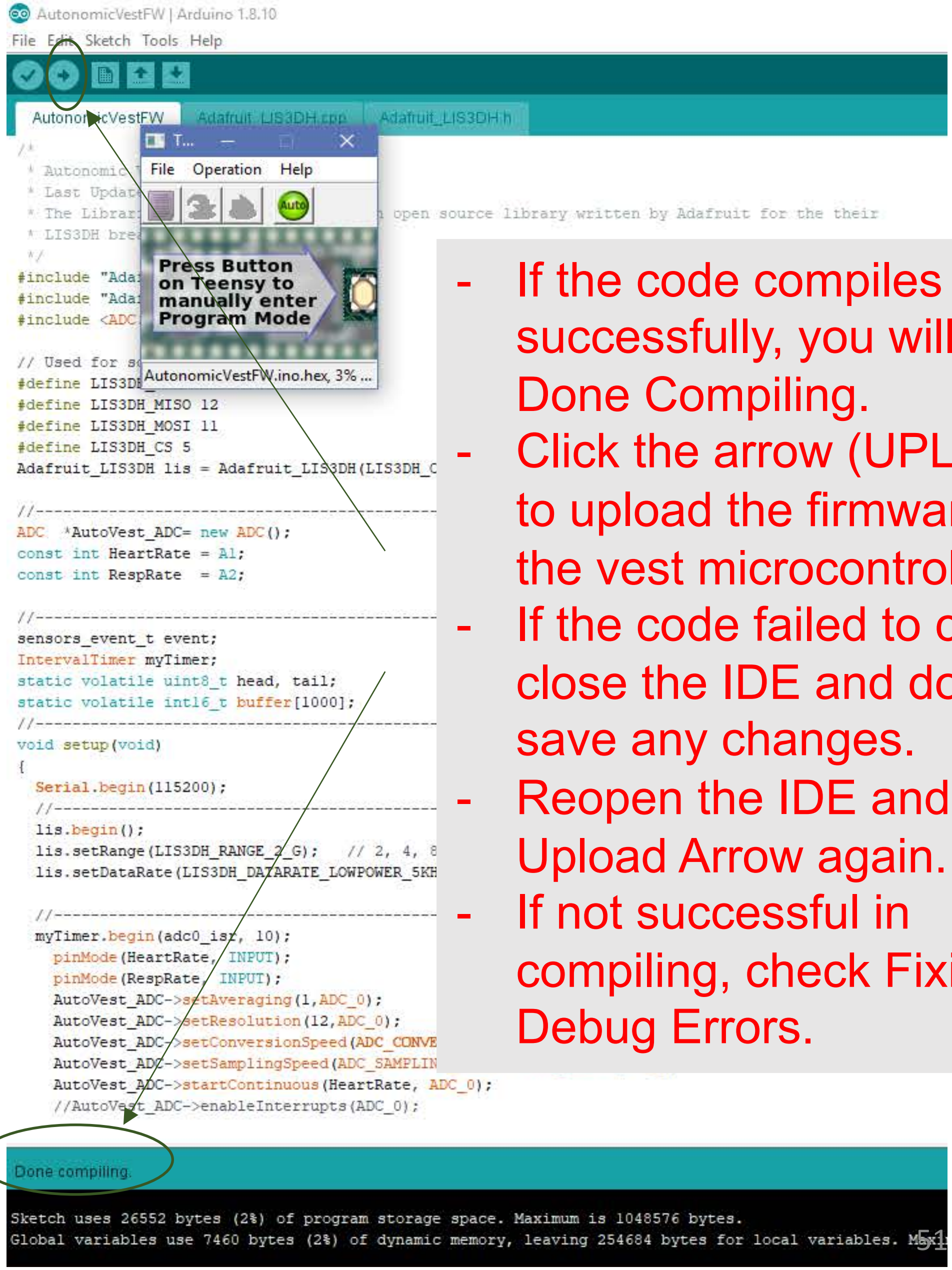

- If the code compiles successfully, you will see Done Compiling.
- Click the arrow (UPLOAD) to upload the firmware into the vest microcontroller.
- If the code failed to compile close the IDE and do NOT save any changes.
- Reopen the IDE and click Upload Arrow again.
- If not successful in compiling, check Fixing Debug Errors.

FIXING DEBUG ERRORS - MISSING LIBRARY

AutonomicVestFW | Arduino 1.8.8

File Edit Sketch Tools Help

AutonomicVestFW | Adafruit_LIS3DH.cpp | Adafruit_LIS3DH.h

```
#include "Adafruit_LIS3DH.h"
#include "Adafruit_Sensor.h"
#include <ADC.h>

#define LIS3DH_CLK 13
#define LIS3DH_MISO 12
#define LIS3DH_MOSI 11
#define LIS3DH_CS 5
Adafruit_LIS3DH lis = Adafruit_LIS3DH(LIS3DH_CS);

//------------------------------------------------------------------------------------------
ADC  *AutoVest_ADC= new ADC();
const int HeartRate = A1;
const int RespRate  = A2;

//------------------------------------------------------------------------------------------
sensors_event_t event;
IntervalTimer myTimer;
static volatile uint8_t head, tail;
static volatile int16_t buffer[1000];
//------------------------------------------------------------------------------------------
void setup(void)
{
  Serial.begin(115200);
  //-----------------------------------------------------------
  lis.begin();
  lis.setRange(LIS3DH_RANGE_2_G);   // 2, 4, 8 or 16 G!
  lis.setDataRate(LIS3DH_DATARATE_LOWPOWER_5KHZ);
```

- The compiler in this case is complaining that Adafruit_Sensor.h library cannot be accessed.
- The file is missing from Arduino libraries.

Error compiling for board Teensy 3.6.

Copy error messages

```
In file included from C:\Users\mamaw\OneDrive\Documents\Arduino\AutonomicVestFW\AutonomicVestFW.ino:2:0:

C:\Users\mamaw\AppData\Local\Temp\arduino_build_450294\sketch\Adafruit_LIS3DH.h:35:29: fatal error: Adafruit_Sensor.h: No such file or directory
```

Teensy 3.6, Serial, 180 MHz, Faster, US English on COM6

FIXING DEBUG ERRORS - MISSING LIBRARY

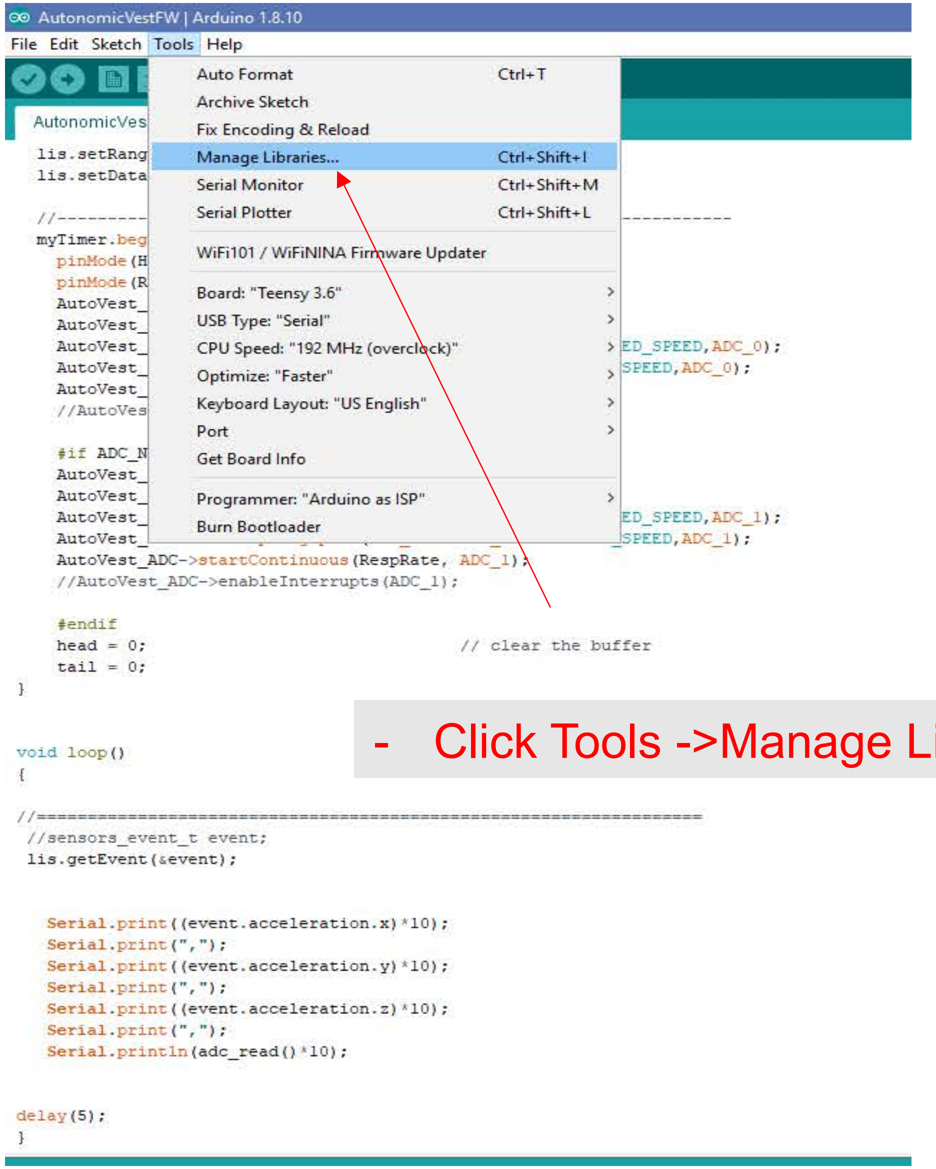

- Click Tools ->Manage Libraries

Library Manager Window

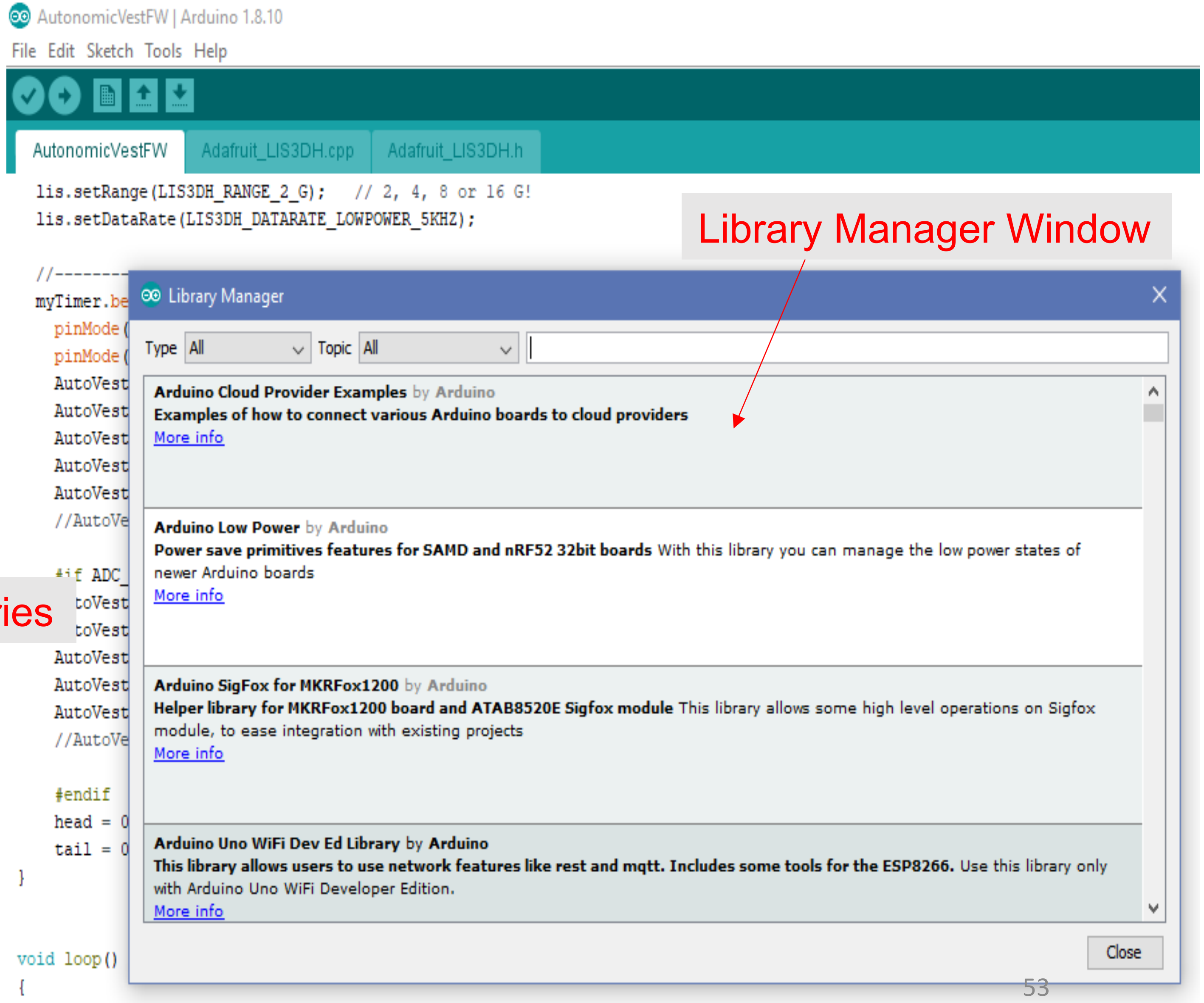

FIXING DEBUG ERRORS - MISSING LIBRARY

- Type Adafruit_sensor.h
- Scroll Up/Down to find "Adafruit Unified Sensor by Adafruit - Required for all Adafruit Unified sensor based libraries"
- Click install button next to it.
- After installation is complete Close the Library manager and the Arduino IDE software.

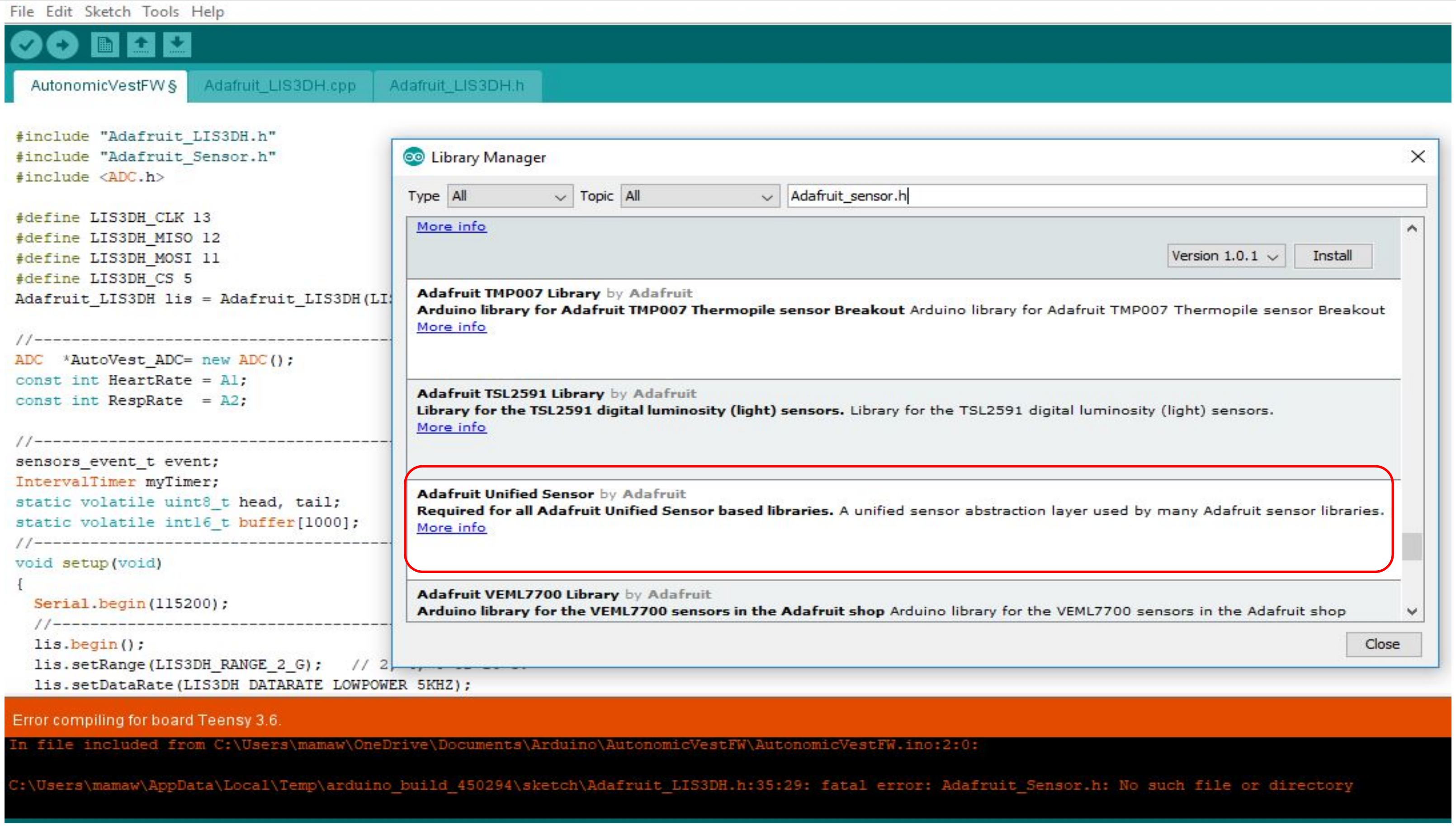

FIXING DEBUG ERRORS - MISSING LIBRARY

AutonomicVestFW | Arduino 1.8.10

File Edit Sketch Tools Help

AutonomicVestFW | Adafruit_LIS3DH.cpp | Adafruit_LIS3DH.h

```
#define LIS3DH_MISO 12
#define LIS3DH_MOSI 11
#define LIS3DH_CS 5
Adafruit_LIS3DH lis = Adafruit_LIS3DH(LIS3DH_CS); // Config Hardware SPI Communication

//-----------------------------------------------------------------------------------------
ADC  *AutoVest_ADC= new ADC();
const int HeartRate = A1;
```

- Open Arduino
- Open project AutonomicVestFW (if not opened Automatically).
- Click Upload Arrow. This will first compile the code then upload it if no errors arise.
- If errors arise at this stage. Delete the firmware folder in the Arduino Folder (…..\Documents\Arduino\AutonomicVestFW).
- Unzip the firmware folder and copy again to the Arduino Folder.
- Re-compile.

```
AutoVest_ADC->setConversionSpeed(ADC_CONVERSION_SPEED::MED_SPEED,ADC_0);
AutoVest_ADC->setSamplingSpeed(ADC_SAMPLING_SPEED::HIGH_SPEED,ADC_0);
AutoVest_ADC->startContinuous(HeartRate, ADC_0);
//AutoVest_ADC->enableInterrupts(ADC_0);

#if ADC_NUM_ADCS>1
AutoVest_ADC->setAveraging(1,ADC_1);
AutoVest_ADC->setResolution(12,ADC_1);
AutoVest_ADC->setConversionSpeed(ADC_CONVERSION_SPEED::MED_SPEED,ADC_1);
AutoVest_ADC->setSamplingSpeed(ADC_SAMPLING_SPEED::HIGH_SPEED,ADC_1);
AutoVest_ADC->startContinuous(RespRate, ADC_1);
//AutoVest_ADC->enableInterrupts(ADC_1);

#endif
head = 0;                         // clear the buffer
tail = 0;
}
```

Done compiling.

CONFIGURING THE BLUETOOTH MODULES

- There are two Bluetooth modules used in this project on; Master/Transceiver for sending Data from the vest and Slave/ Receiver for receiving the Vest Data and make it available for visualization and back up storage using Computer .
- The Master/Transceiver is installed inside the box with charger and the battery while the Slave/Receiver is installed in the Dongle together with FTDI module.
- The steps for configuring the two Bluetooth modules are similar with only slight difference in Choosing the Roles and Binding the two modules.
- Before installing the Master device , configuration should be done first to cut down the number of steps needed to build up the vest .

DESOLDERING HEADER-PINS

- Place the Solder Wick on the soldered pin ends
- Place Hot Soldiering iron to melt the solder
- Wait for approximately 30 seconds then move the soldering iron to the spots where there is solder left.
- Do not overheat the board, move soldering iron away if 2 mins have elapsed since the start of desoldering.
- Repeat the procedure until most of the solder around the pins is wicked.

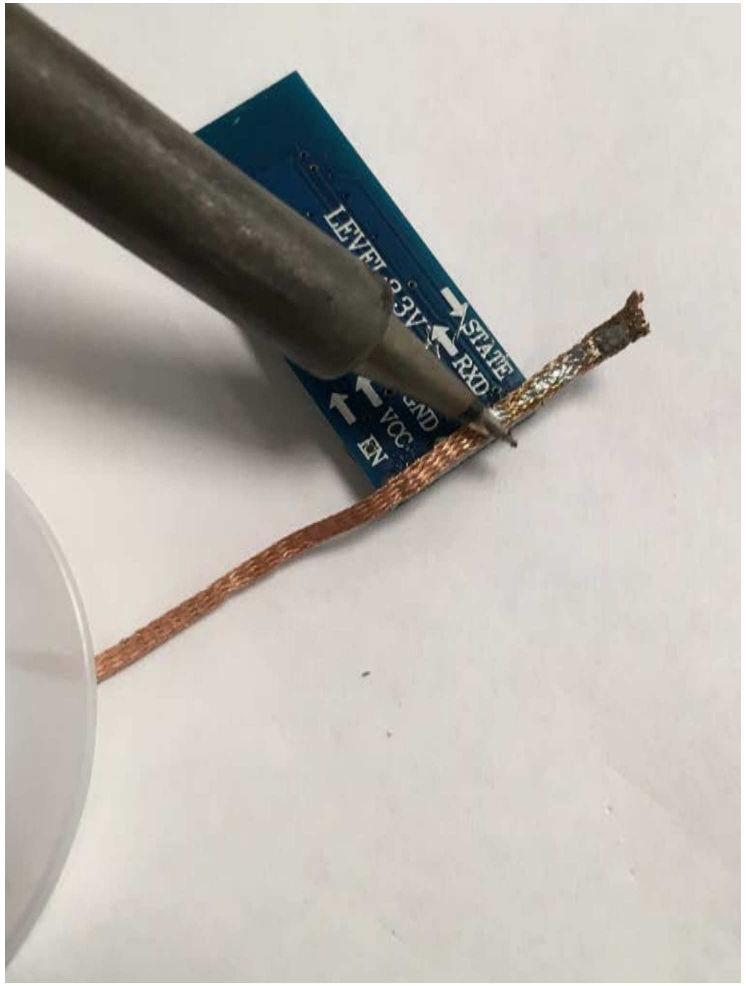

DESOLDERING HEADER-PINS

- Place the Bluetooth module on higher and stable surface.
- While holding the module with one hand , press the pins downward using hot soldering iron.
- Cautiously push the pins down as far possible through the holes.
- Once down flip the module and gently pull the pins out using a pair of pliers.
- If there are pins that are not coming out easily, use the wick again as explained in the beginning . You may solder a little on the wick to make it more absorbent.
- Once all pins are out use the wick to clear the holes.

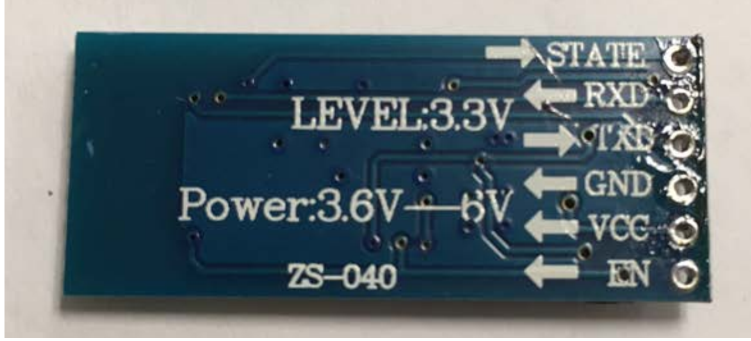

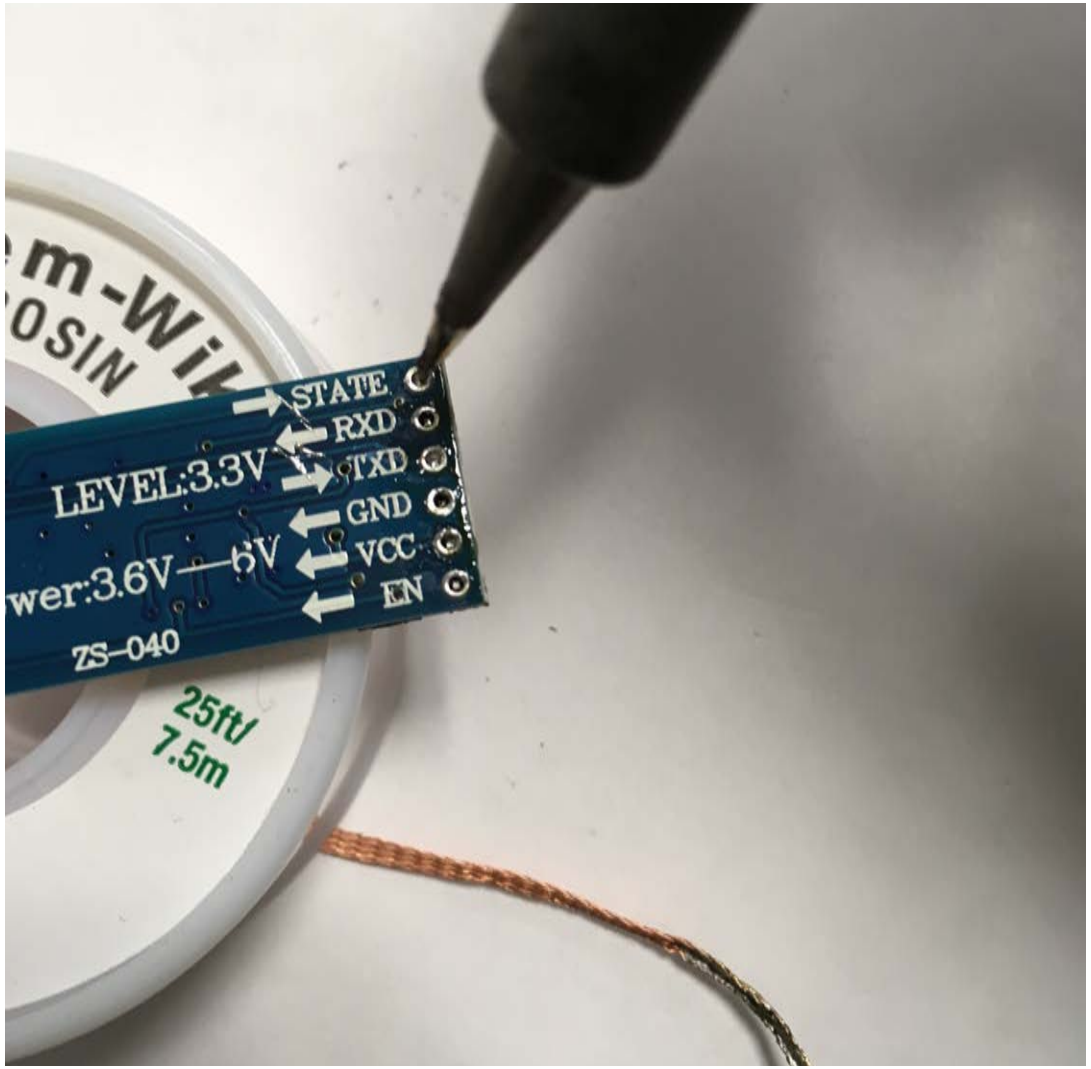

PREPPING CONNECTION WIRES

- Cut four different cables of length 70 mm,
- Strip the ends.
- Solder the stripped ends as shown in picture.
- Only Vcc, Gnd, Rx &Tx contacts of the Bluetooth will be used.

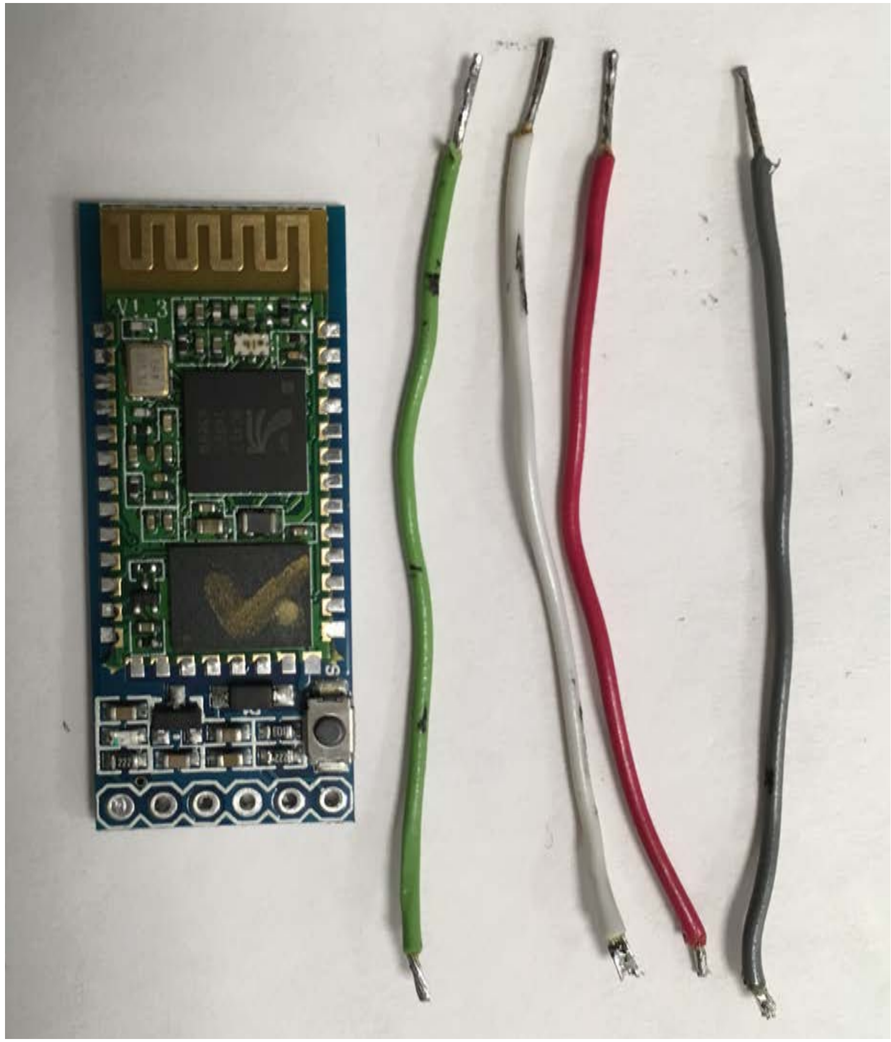

SOLDERING THE CONNECTION-WIRES

- Place one wire's end in one of the hole (VCC,GND,TXD or RXD) as shown in the leftmost picture.
- Flip the module and solder the wire.
- Make sure that no solder contacts the neighboring holes.
- Repeat the rest this procedure for the remaining holes.
- Repeat the same procedure for both Bluetooth Modules(Transceiver & Receiver).

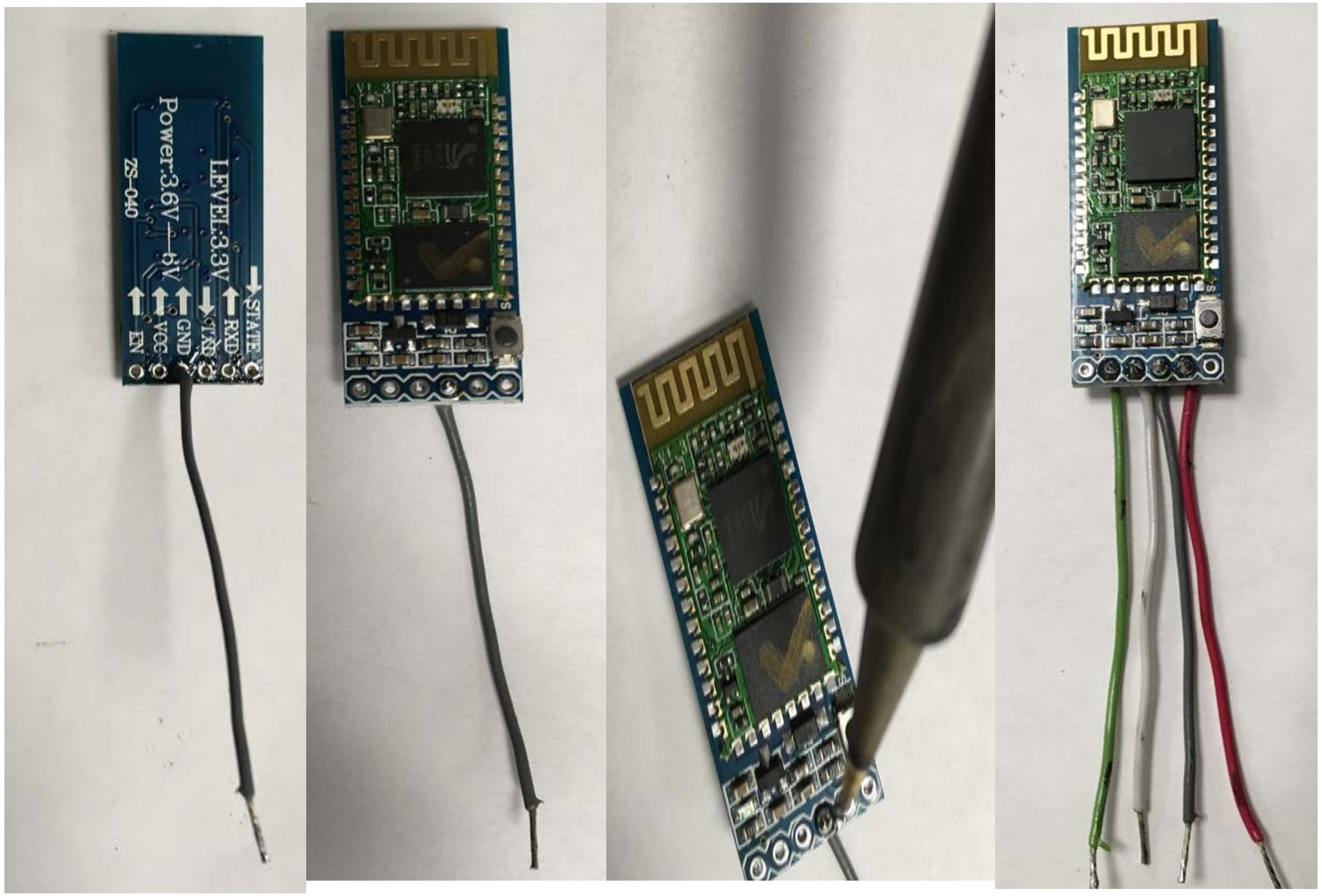

CONNECTING FTDI MODULE

- To Connect any wire to the FTDI just insert the end of the wire into Header pin socket (Hole).
- Connect the FTDI to Bluetooth Module as shown.
- Remember the RXD of FTDI must connected to TXD of the Bluetooth and its TXD must be connected to RXD of the Bluetooth.

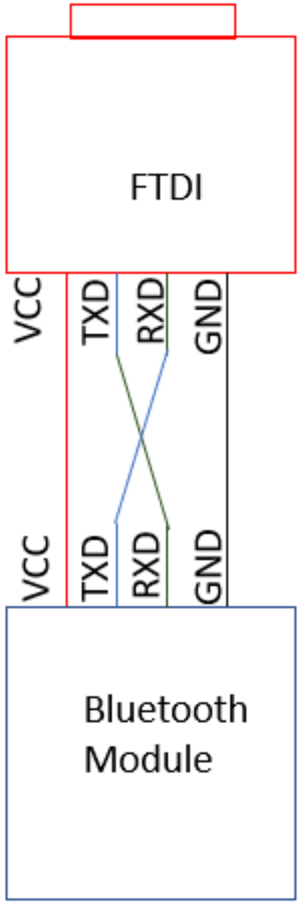

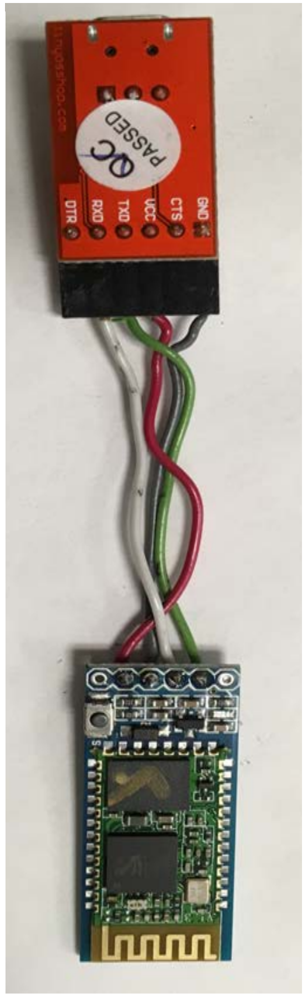

CONFIGURING MASTER/SLAVE MODULE

NOTE: If you've not installed Arduino IDE , do so by following instruction given in Uploading Firmware.

- Open Arduino IDE
- Go to Tools ->Port and note down the available Serial Ports (COMs)

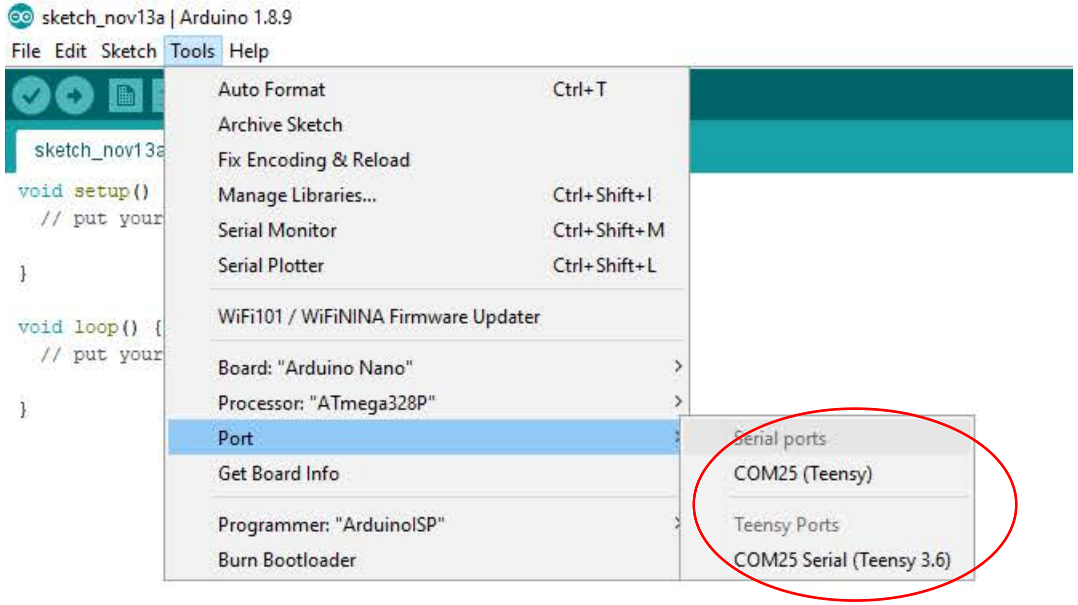

Available COM ports

CONFIGURING MASTER/SLAVE MODULE

NOTE: If you’ve not installed Arduino IDE , do so by following instruction given in Uploading Firmware.

- Connect micro-USB to the FTDI (keep it connected throughout the procedure).
- Go to Arduino IDE Tools ->Port and note down the new available Serial Port (COM).
- Remember this COM port no.
- Unplug the FTDI from the computer.

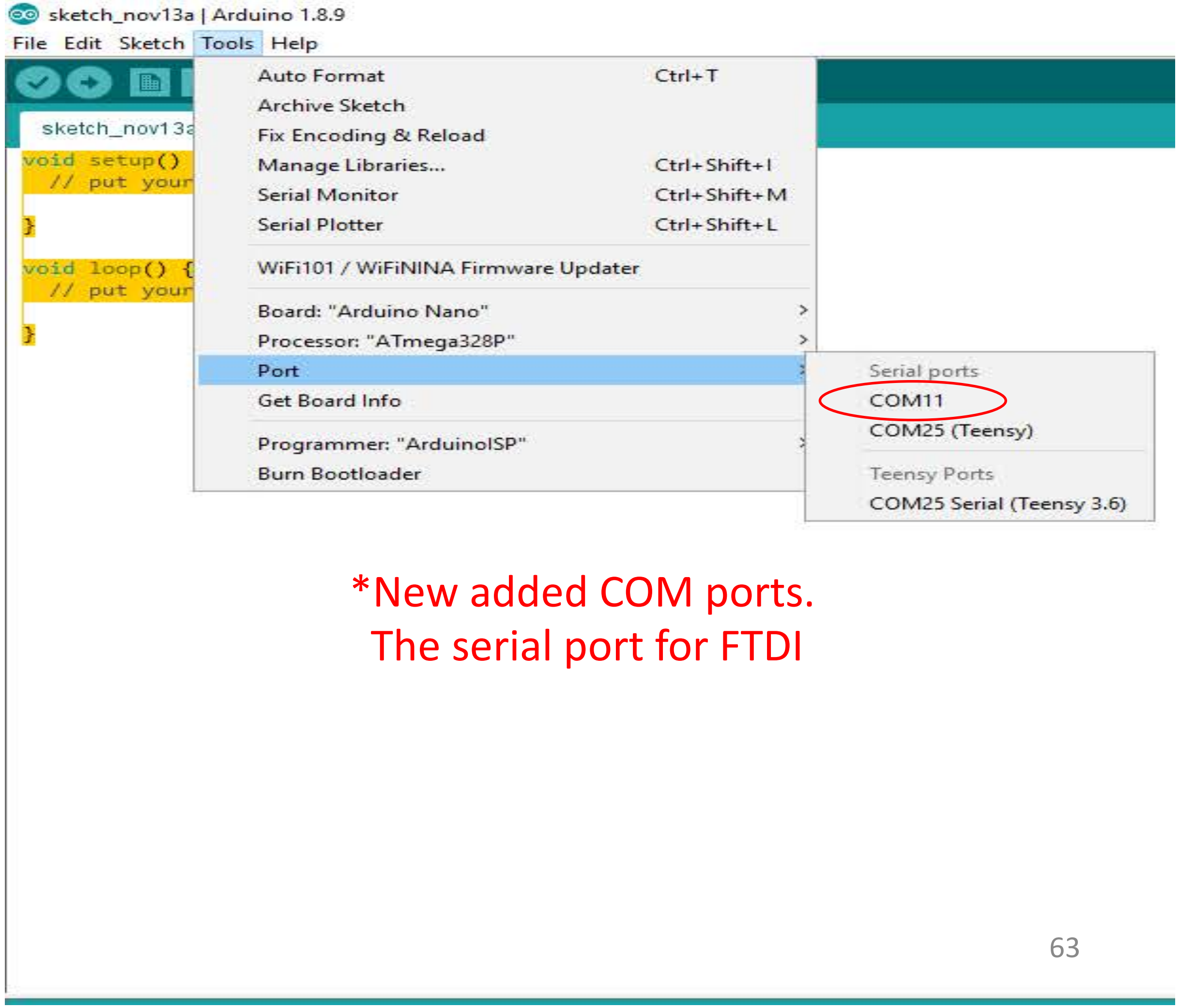

*New added COM ports.
The serial port for FTDI

CONFIGURING MASTER/SLAVE MODULE

- Press the AT Command mode button
- While Pressing the Button connect the FTDI to the computer.
- As soon as the LED on Bluetooth comes on release the Button.
- If the module enters the AT Command Mode, the LED will blink every 2 seconds.

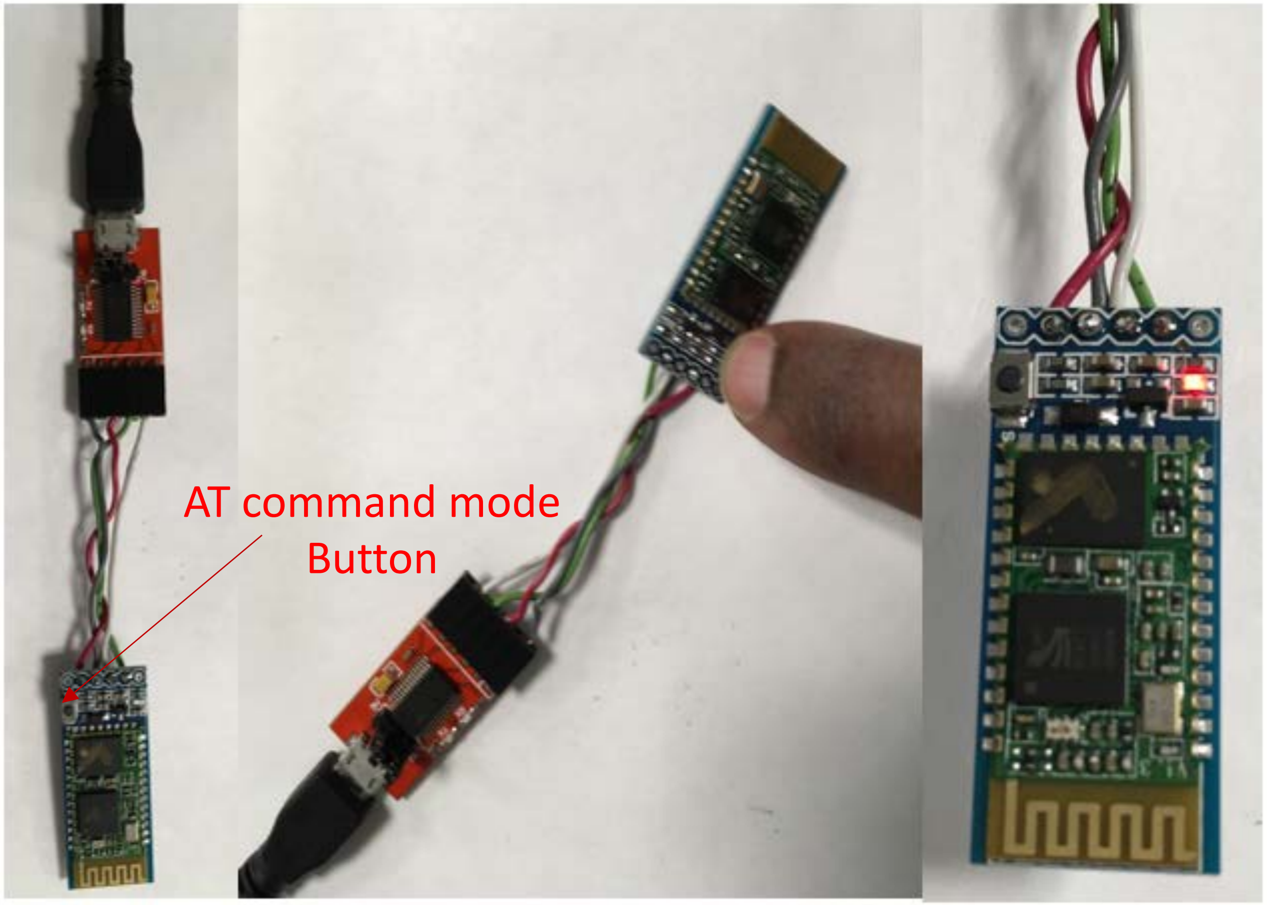

CONFIGURING MASTER/SLAVE MODULE

NOTE: If you've not installed Arduino IDE , do so by following instruction given in Uploading Firmware.

- Go to Arduino IDE Tools ->Port and select the FTDI Serial port e.g. COM11
- Go to Tools and click to open Serial Monitor.

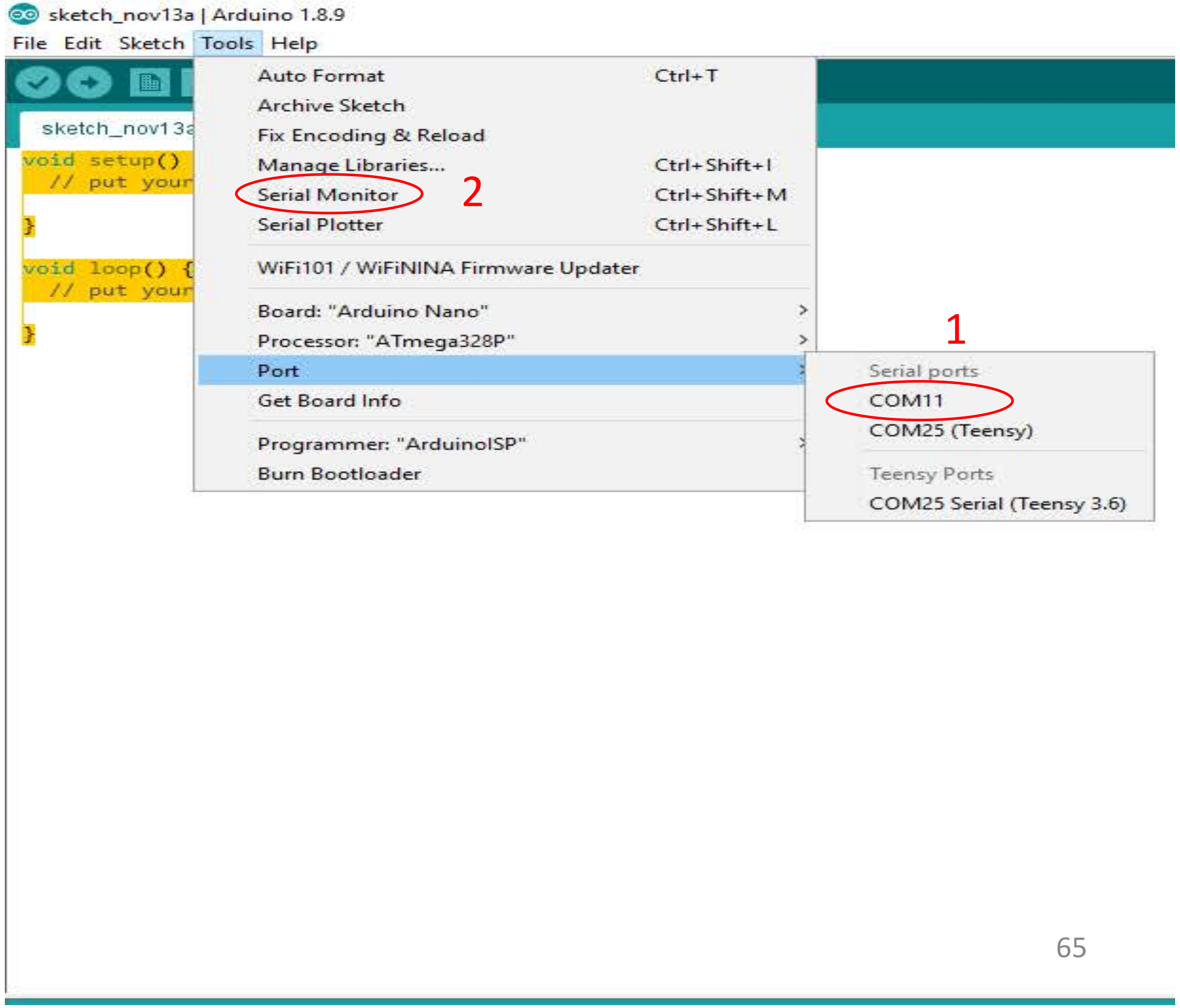

CONFIGURING MASTER/SLAVE MODULE

- Confirm that Serial COM port opened is the right one by checking the no. at top left side of the window. E.g. COM11
- Change BAUD Rate to 38400 baud using scroll menu, second from the Right on the bottom of the COM window.
- Make sure the window is set for Both NL & CR .

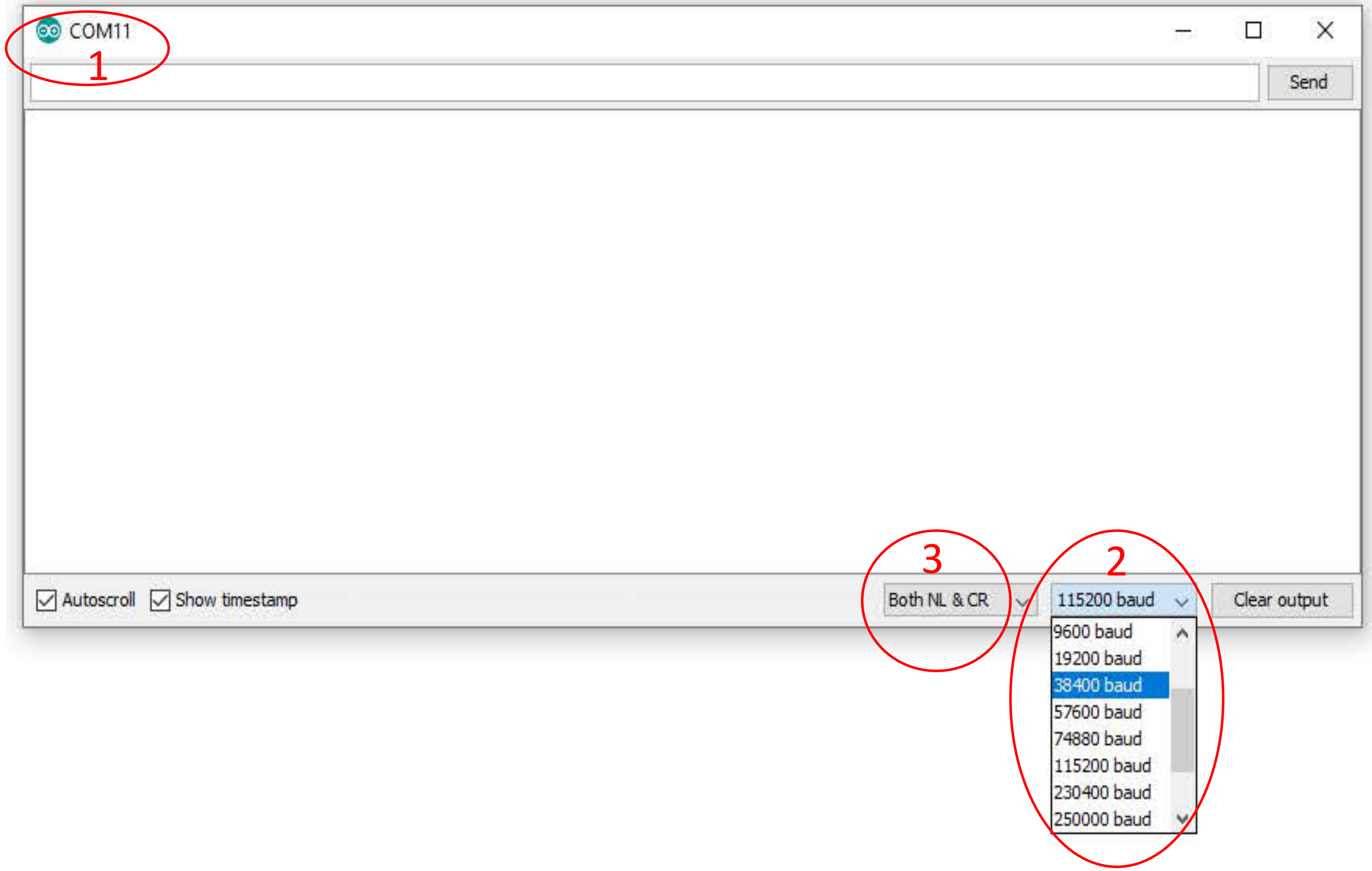

CONFIGURING MASTER/SLAVE

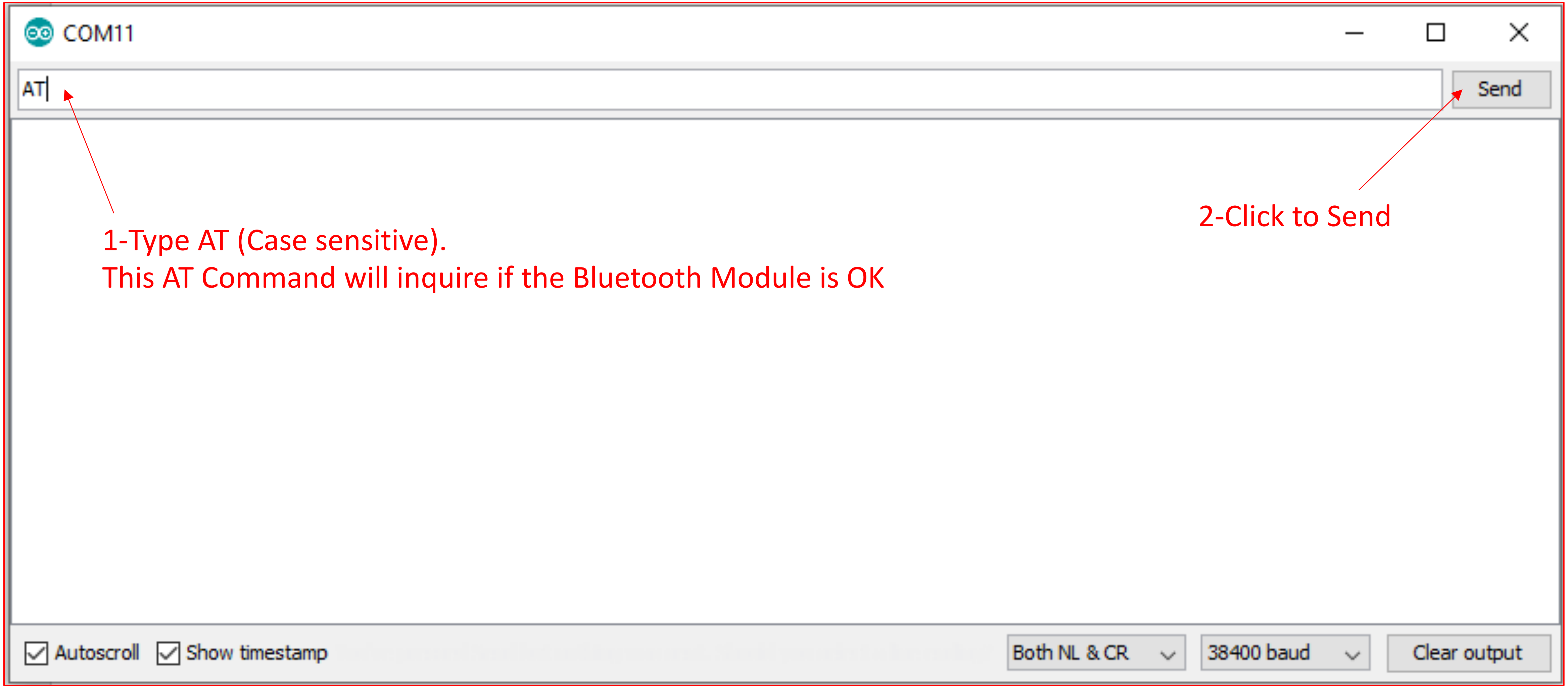

CONFIGURING MASTER/SLAVE

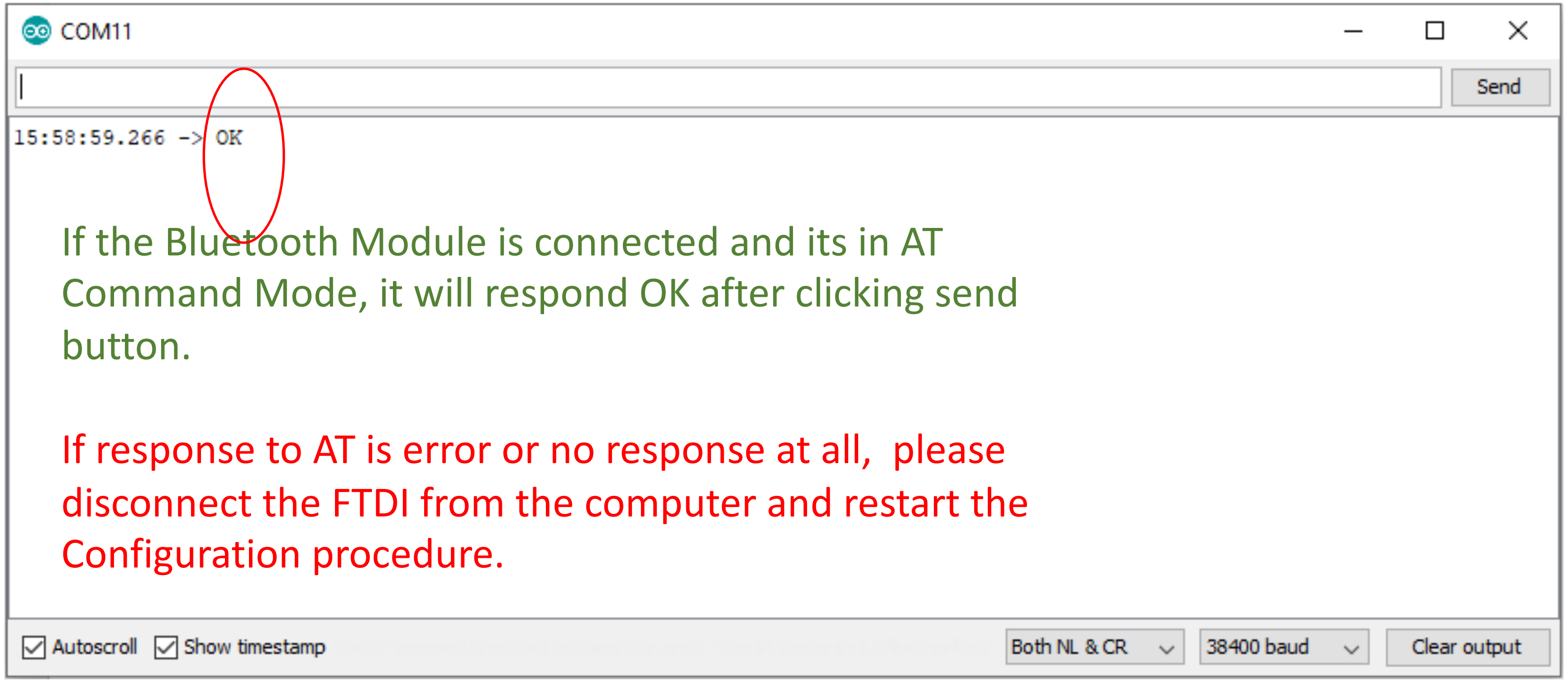

If the Bluetooth Module is connected and its in AT Command Mode, it will respond OK after clicking send button.

If response to AT is error or no response at all, please disconnect the FTDI from the computer and restart the Configuration procedure.

CONFIGURING SLAVE MODULE

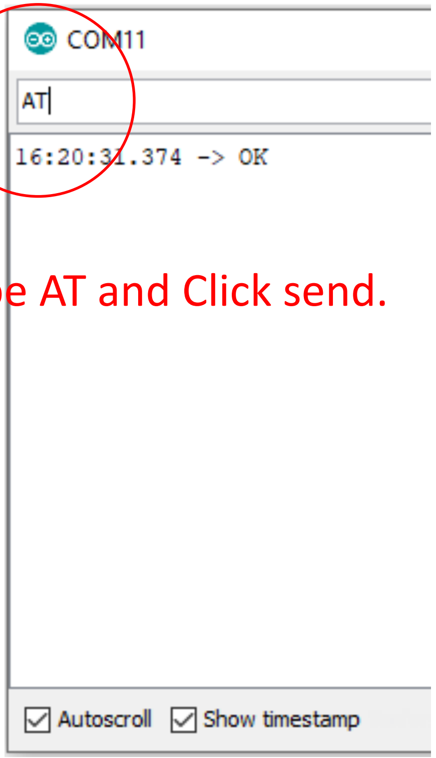

Type AT and Click send.

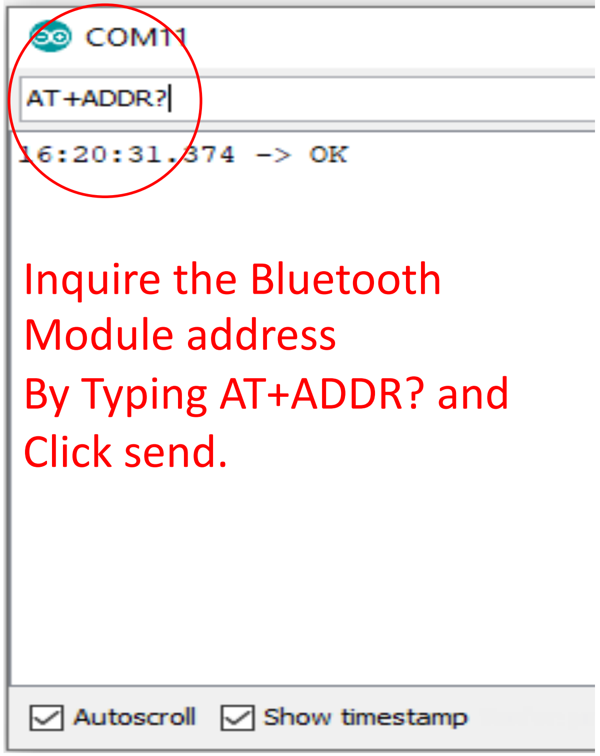

Inquire the Bluetooth Module address
By Typing AT+ADDR? and Click send.

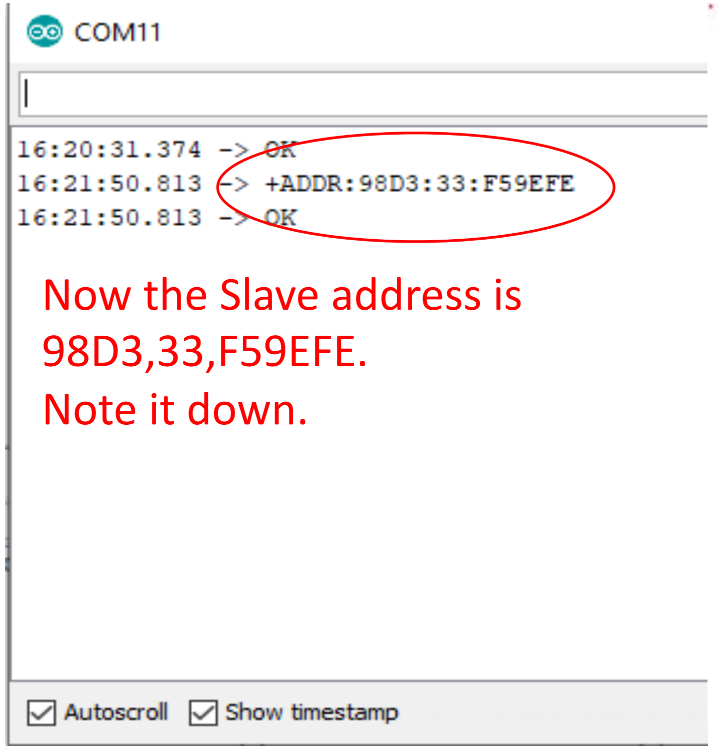

Now the Slave address is 98D3,33,F59EFE.
Note it down.

Label the Slave Module

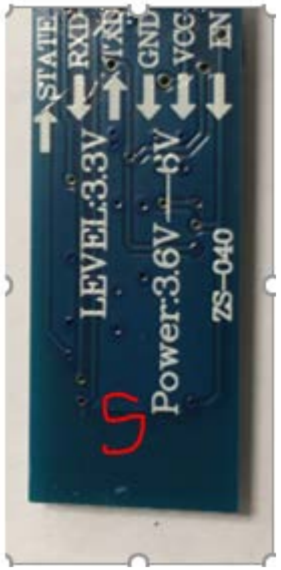

CONFIGURING SLAVE MODULE

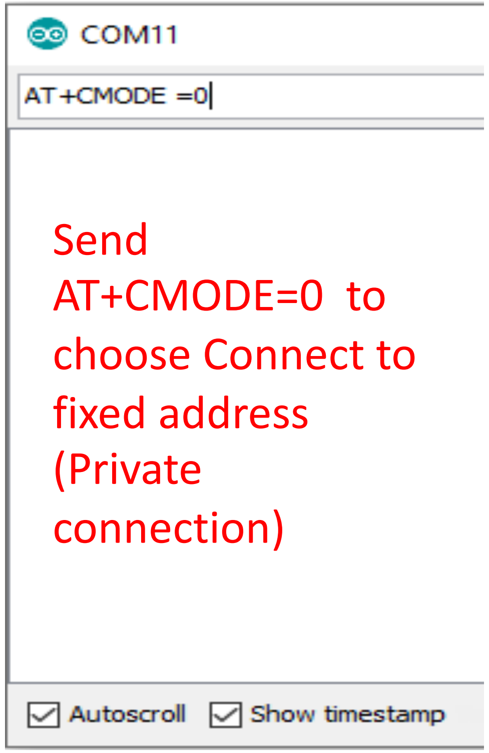

Send AT+CMODE=0 to choose Connect to fixed address (Private connection)

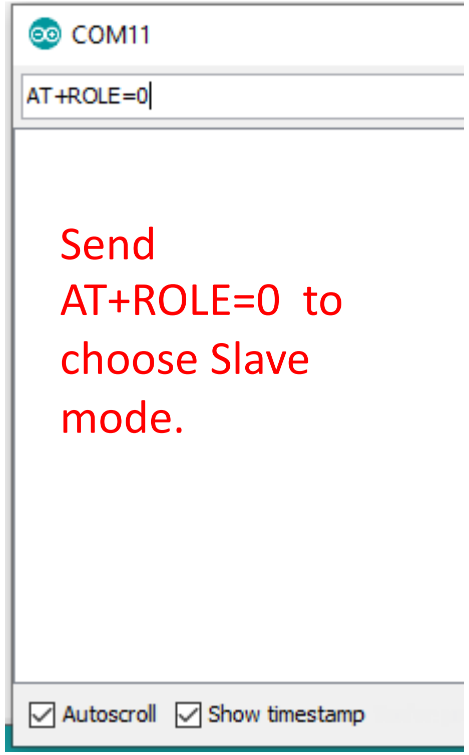

Send AT+ROLE=0 to choose Slave mode.

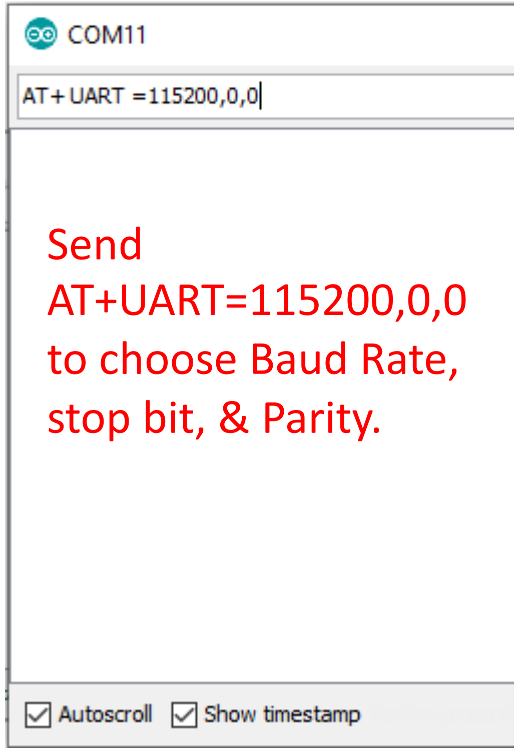

Send AT+UART=115200,0,0 to choose Baud Rate, stop bit, & Parity.

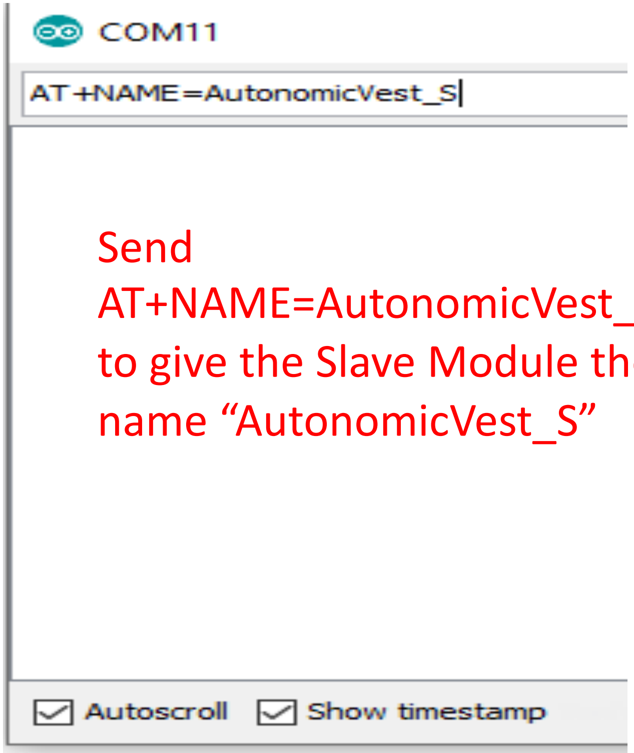

Send AT+NAME=AutonomicVest_S to give the Slave Module the name “AutonomicVest_S”

CONFIGURING MASTER MODULE

- Connect the Master module to FTDI as explained in CONNECTING FTDI MODULE step (Slide 7). Follow the rest of steps up to slide 14.
- Skip to CONFIGURING MASTER MODULE (slide 18).

CONFIGURING MASTER MODULE

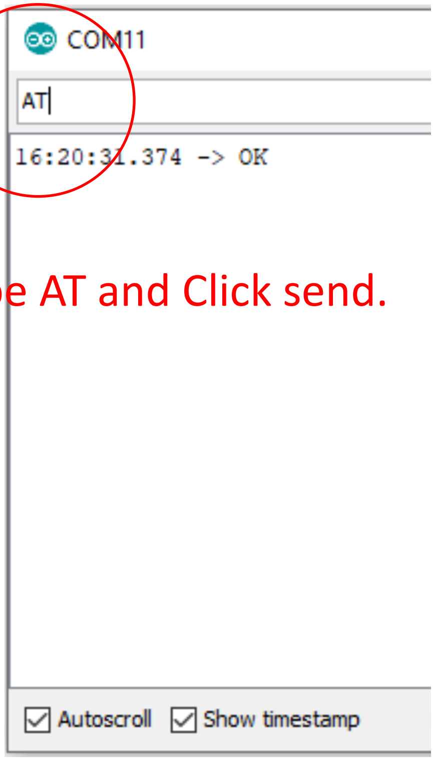

Type AT and Click send.

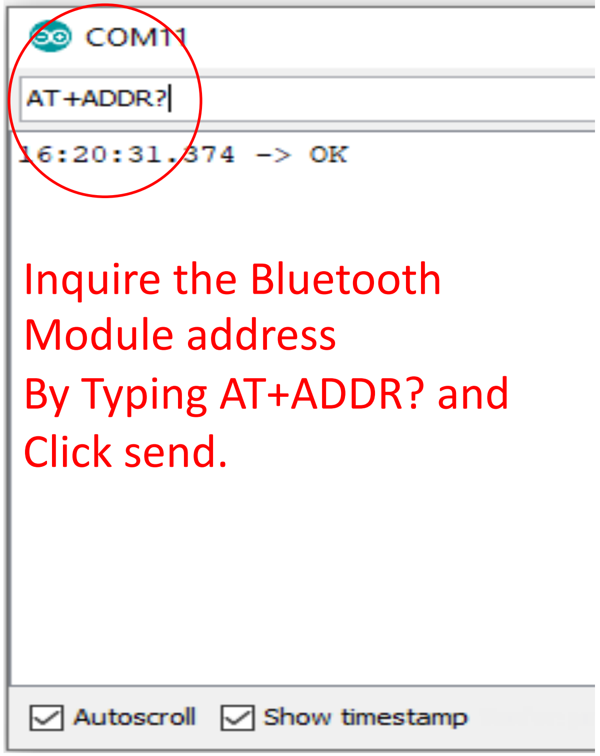

Inquire the Bluetooth Module address
By Typing AT+ADDR? and Click send.

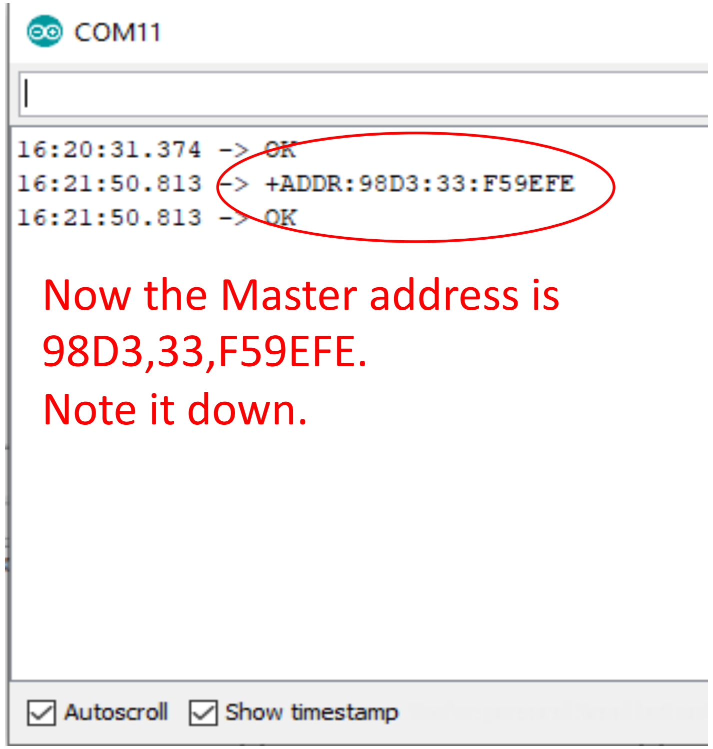

Now the Master address is 98D3,33,F59EFE.
Note it down.

Label the Master Module

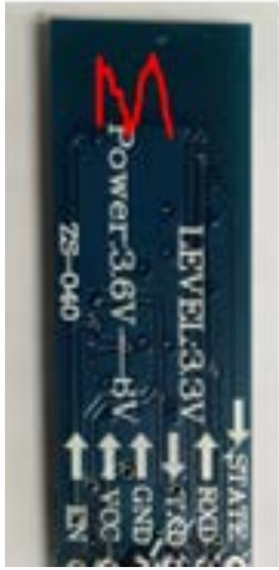

CONFIGURING MASTER MODULE

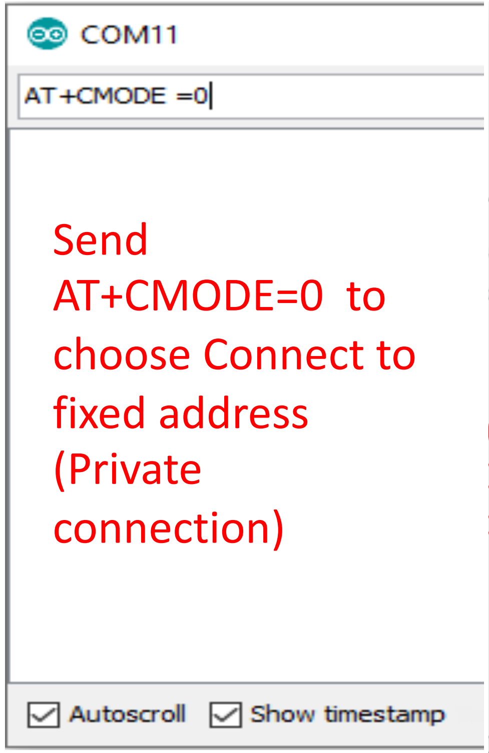

Send
AT+CMODE=0 to choose Connect to fixed address (Private connection)

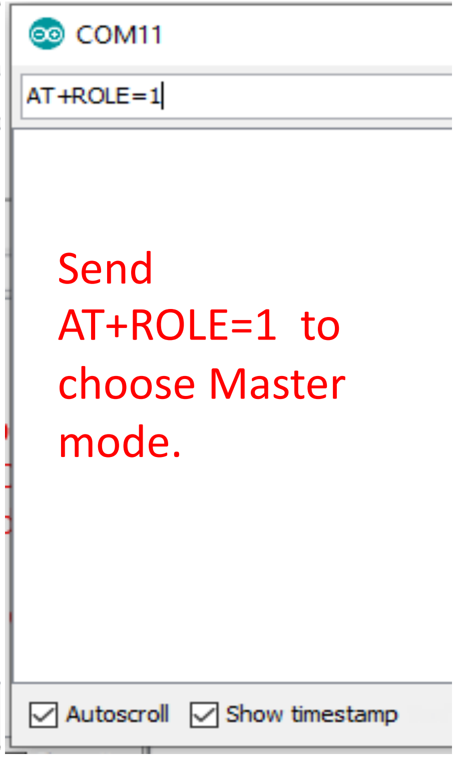

Send
AT+ROLE=1 to choose Master mode.

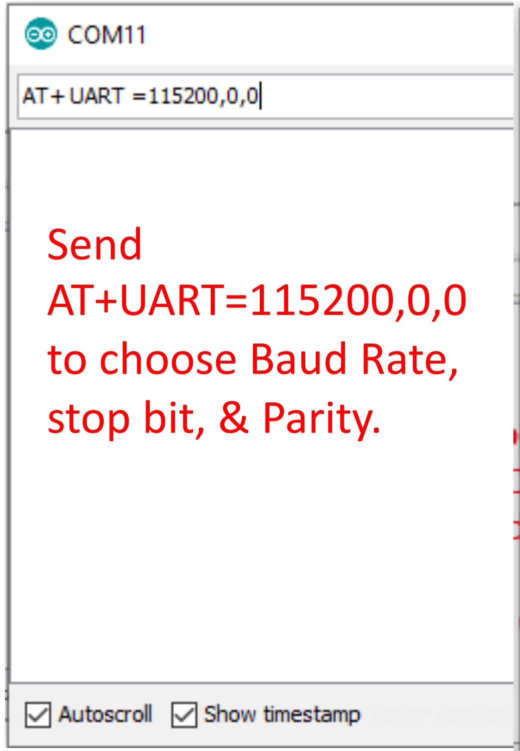

Send
AT+UART=115200,0,0 to choose Baud Rate, stop bit, & Parity.

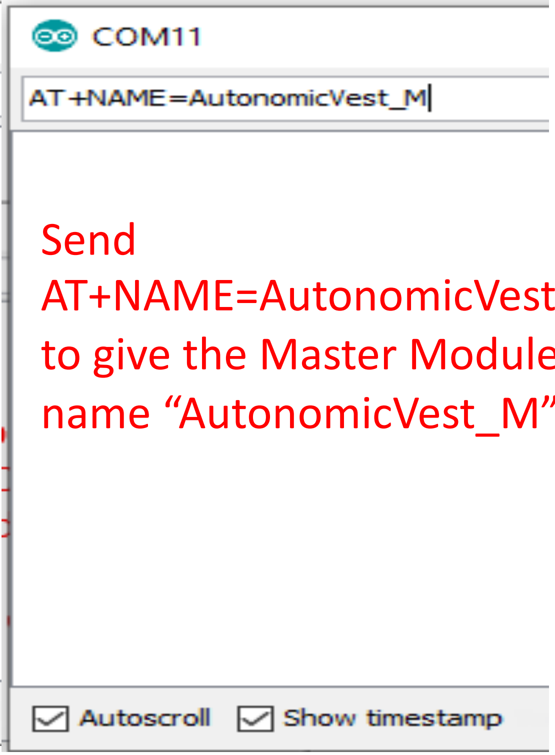

Send
AT+NAME=AutonomicVest_M to give the Master Module the name “AutonomicVest_M”

BINDING MASTER MODULE

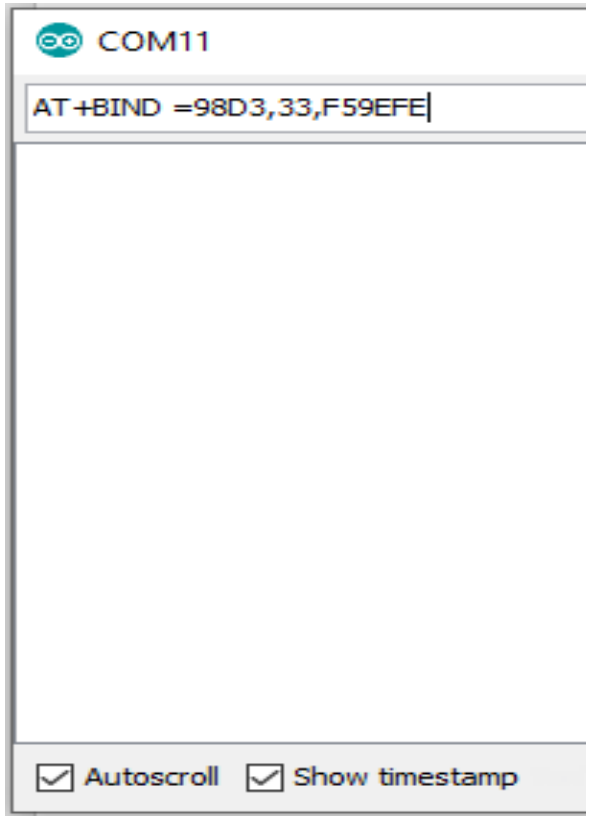

Binding Master to Slave Module

- Type AT+BIND =98D3,33,F59EFE(use the Master's address acquired earlier. Only substitute the Semi Colon with commas.
- Click Send
- Response should OK
- Now Master module is ready to be mounted in the box.

BINDING SLAVE MODULE

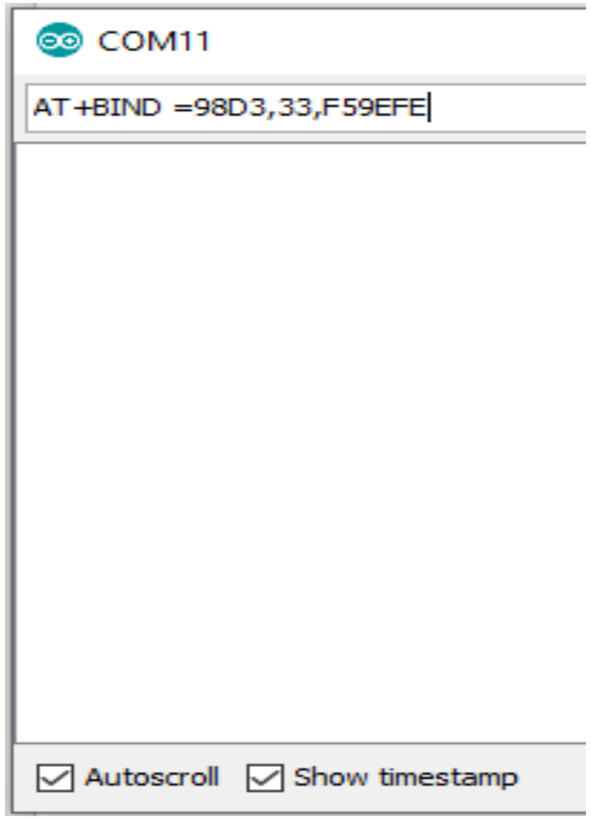

Binding Slave to Master Module

- Reconnect the Slave Module to FTDI as performed earlier and Enter AT Command Mode.
- Type AT+BIND =98D3,33,F59EFE(use the Slave’s address acquired earlier. Only substitute the Semi Colon with commas.
- Click Send
- Response should OK
- Now Slave module is ready to be mounted in the box.

ASSEMBLING BATTERY MANAGEMENT UNIT

NOTE: This procedure must be preceded "Programming the Bluetooth modules"

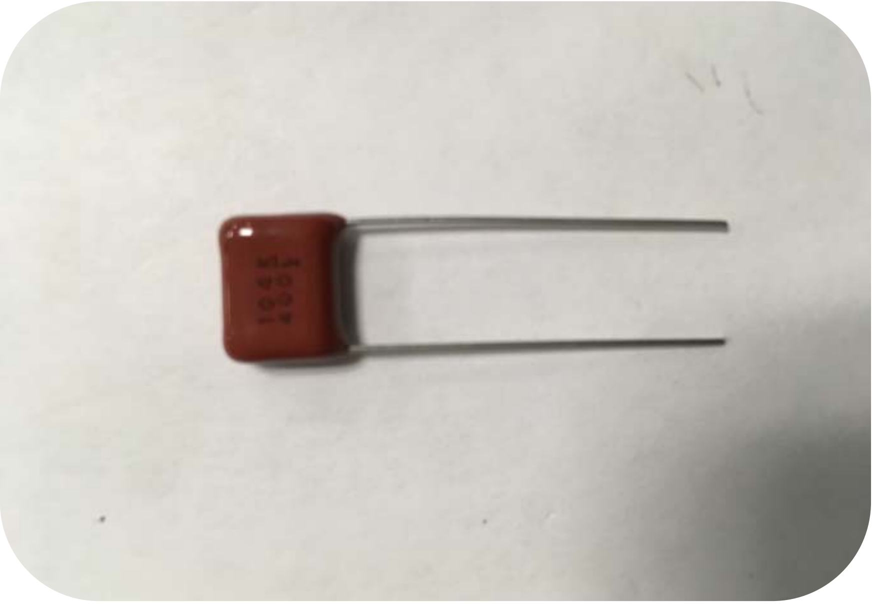

Acquire 100nF capacitor for Noise attenuation.

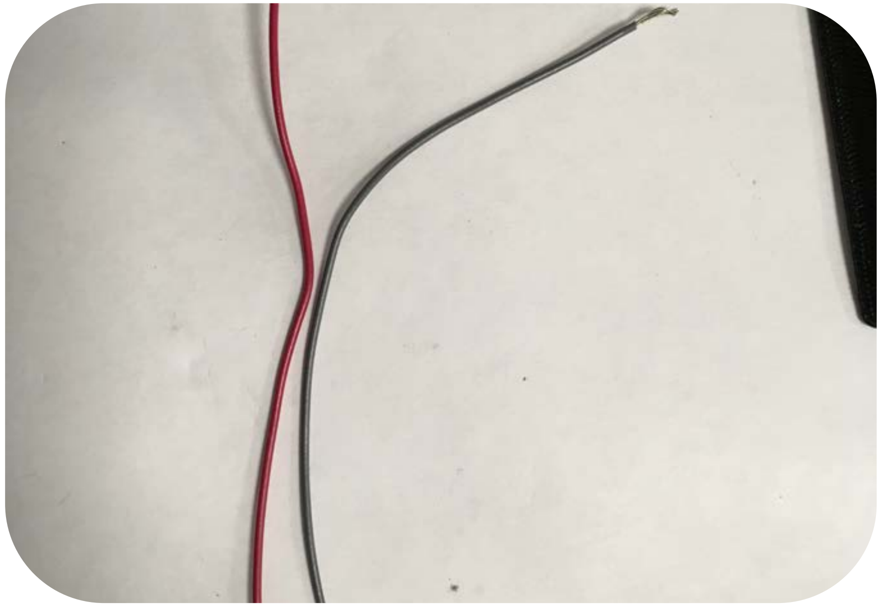

Acquire two different colored wires length about 150mm

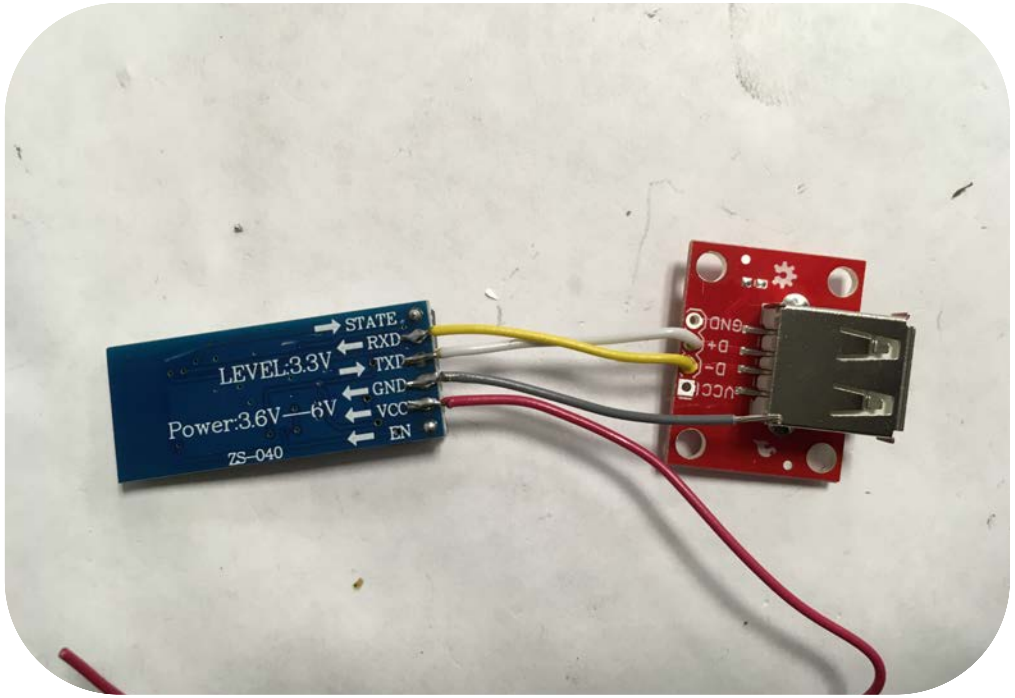

Solder the Master Bluetooth module's RXD wire to USB breakout board D- and TXD to D+.

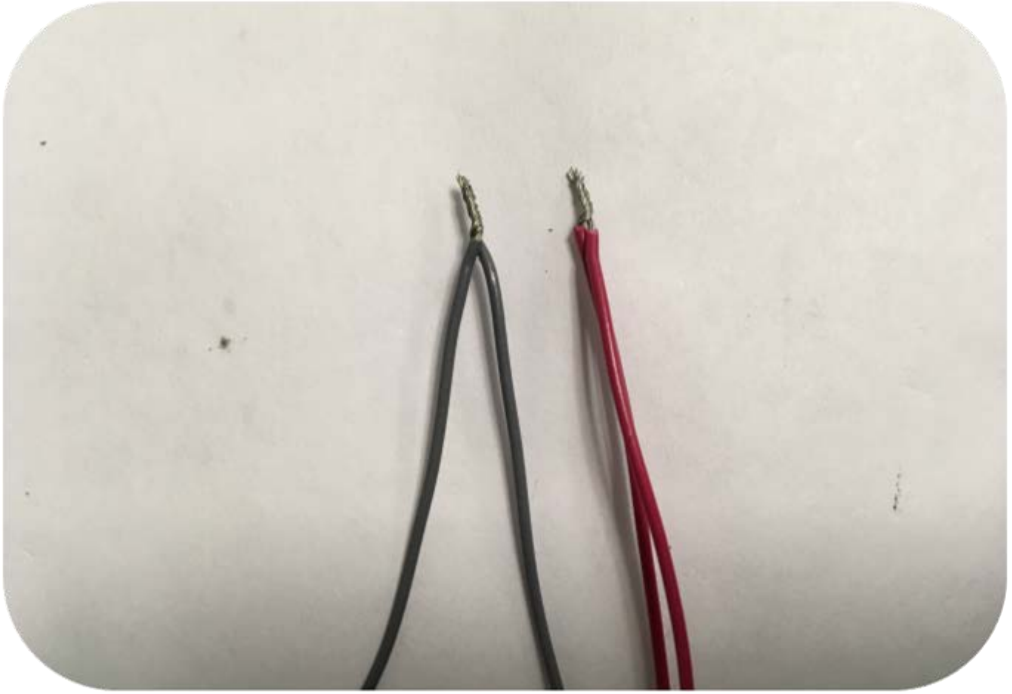

Cut two wires 50mm length for VCC (Red) and GND (Grey/black). Strip and twist the VCC to the Bluetooth Module's VCC and the GND to the module's GND. Solder the twisted joints.

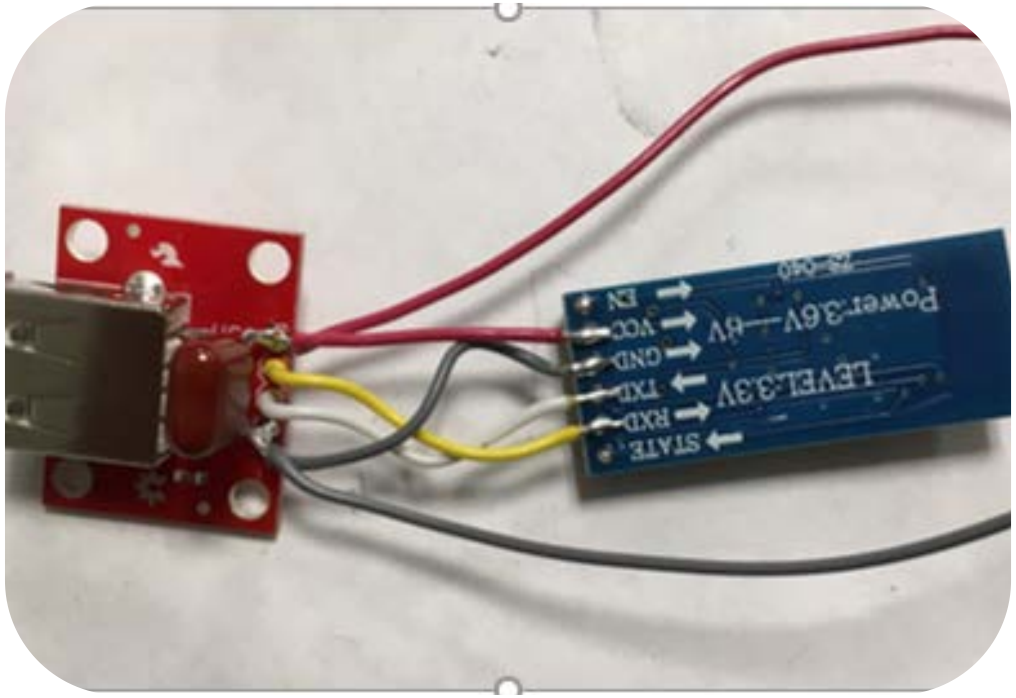

Insert the capacitors leads into the VCC and GND holes in the USB breakout board and solder as shown in the picture. Trim the VCC joint made earlier and solder it to the VCC of the USB board. Do the same with GND.

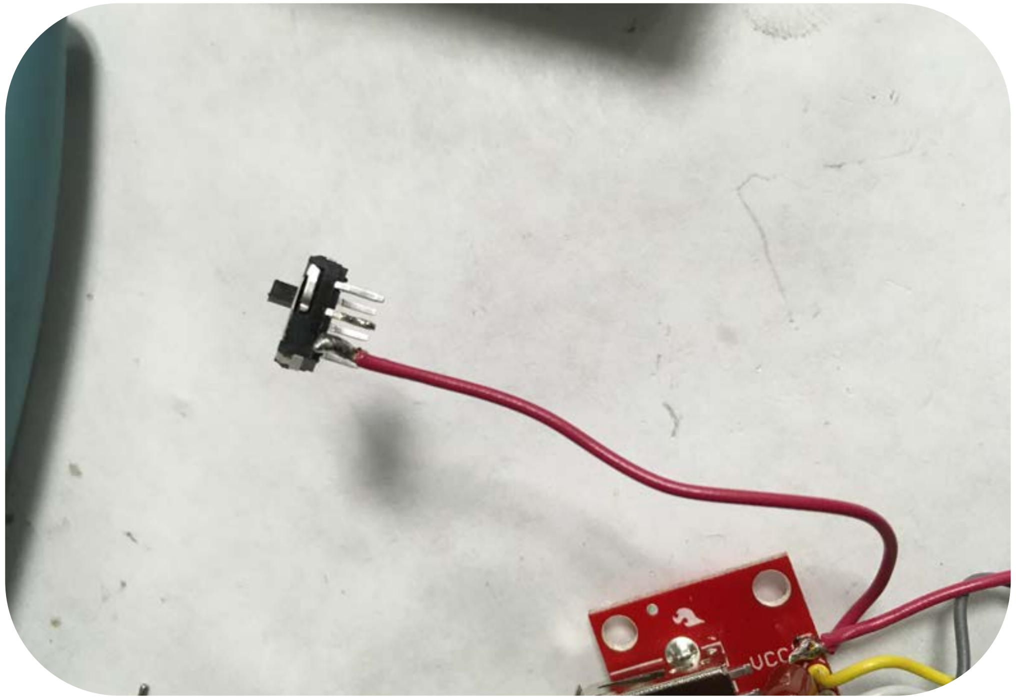

Strip and solder the VCC wire to the Switch as shown

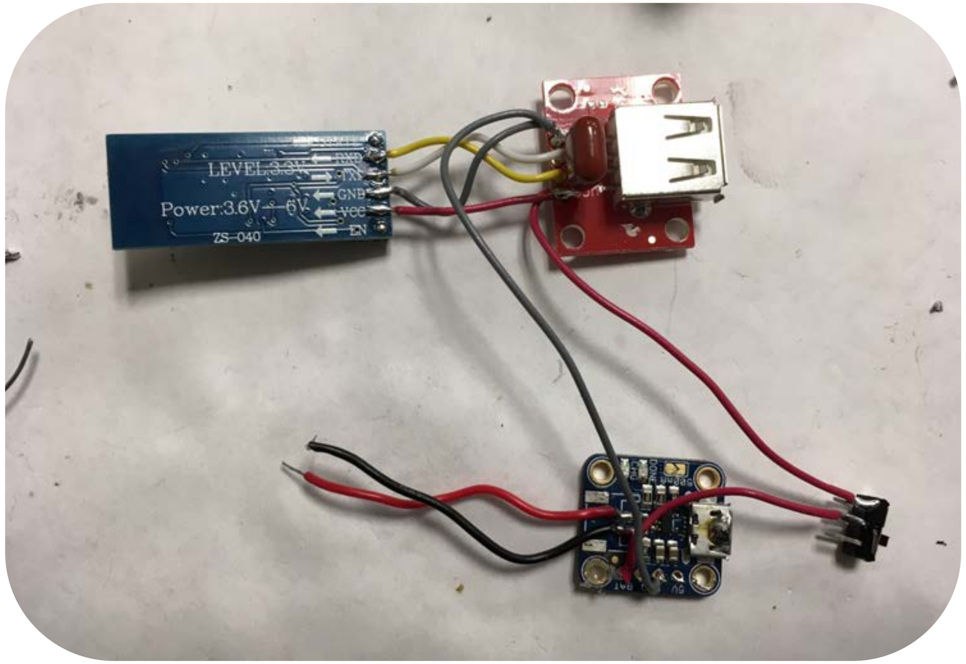

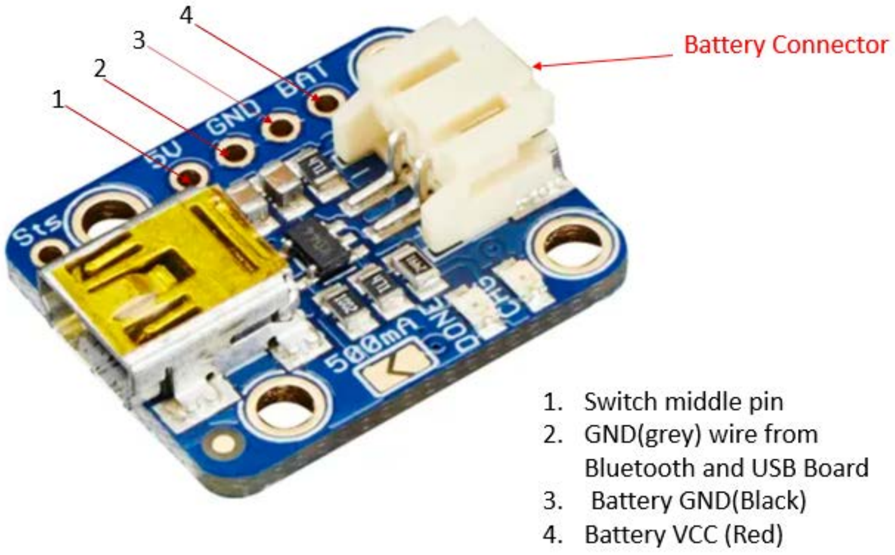

Cut another wire 50mm length for VCC(Red). Strip and solder it to the middle pin of the switch. Solder the other end to the Charger Board on the 5V / VCC . Solder the GND (Grey) wire to the GND next to it.

If you do not have battery connector, solder the Battery VCC to the BAT pin and it's GND to the GND next to it (#3).

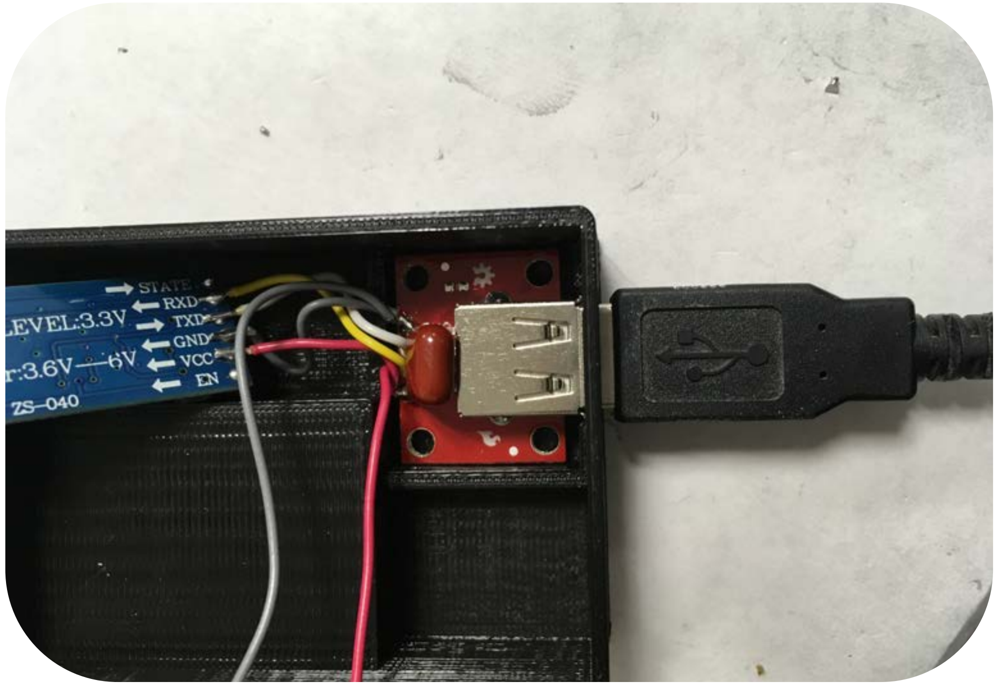

Insert the USB Board in the its compartment and plug in USB cable to help align it. Hot glue the board to stay in place. Do the same to the charger using a micro-USB cable.

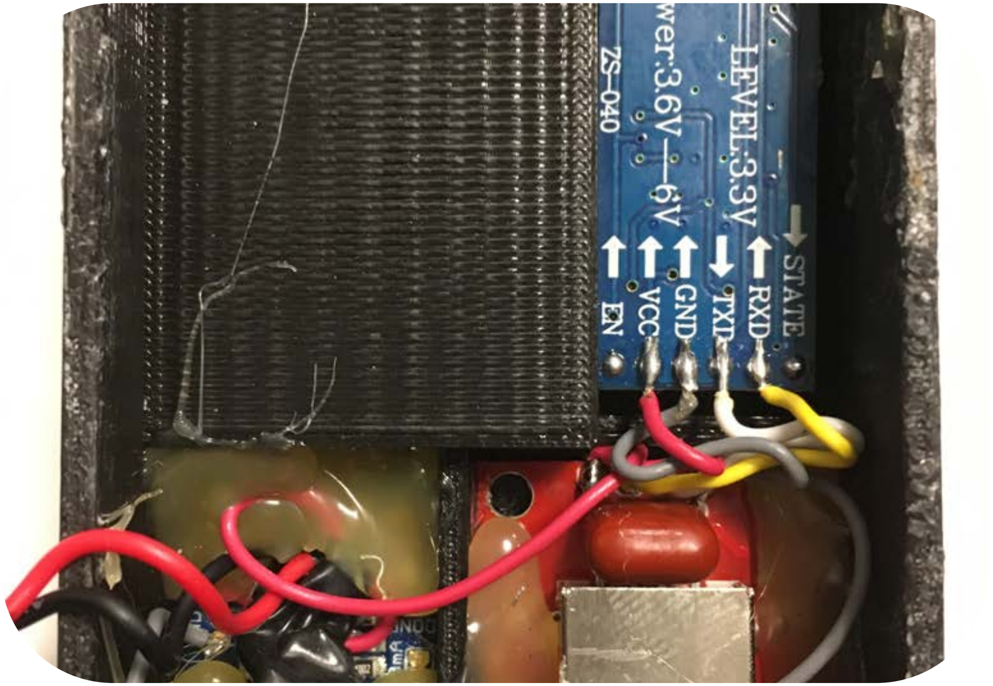

Once the Charger and the USB are secured in their place, Hot glue the Bluetooth module in its place as shown.

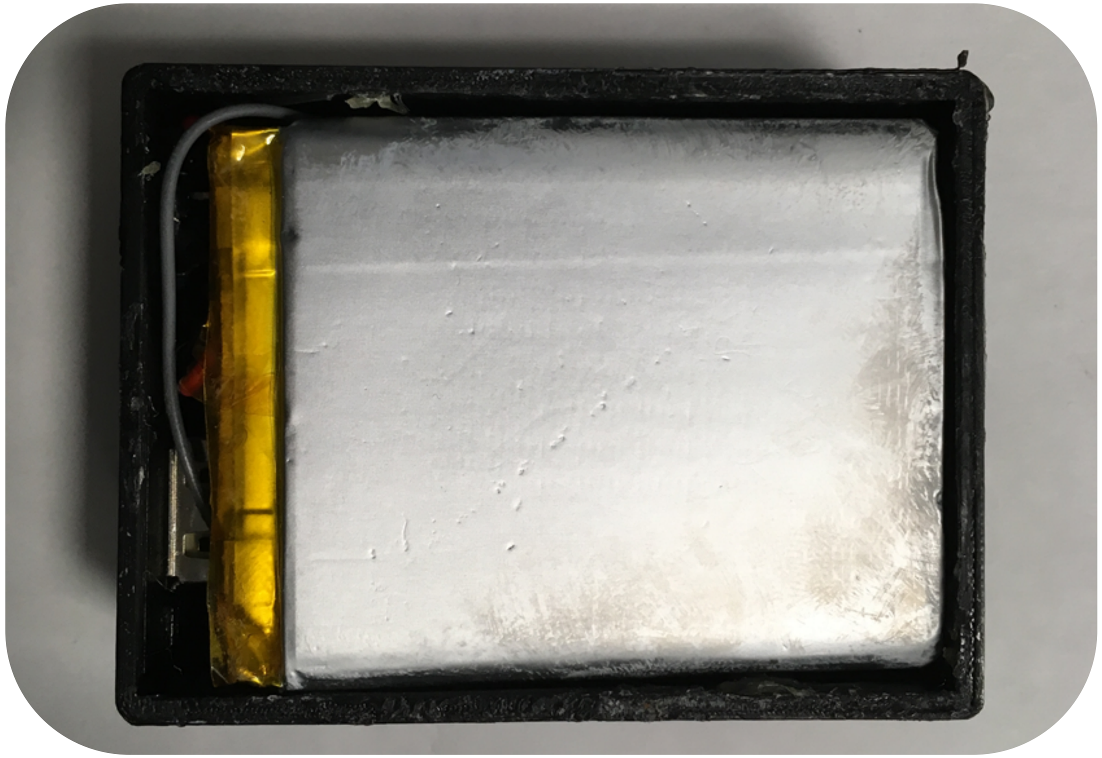

Place the battery inside the box as shown.

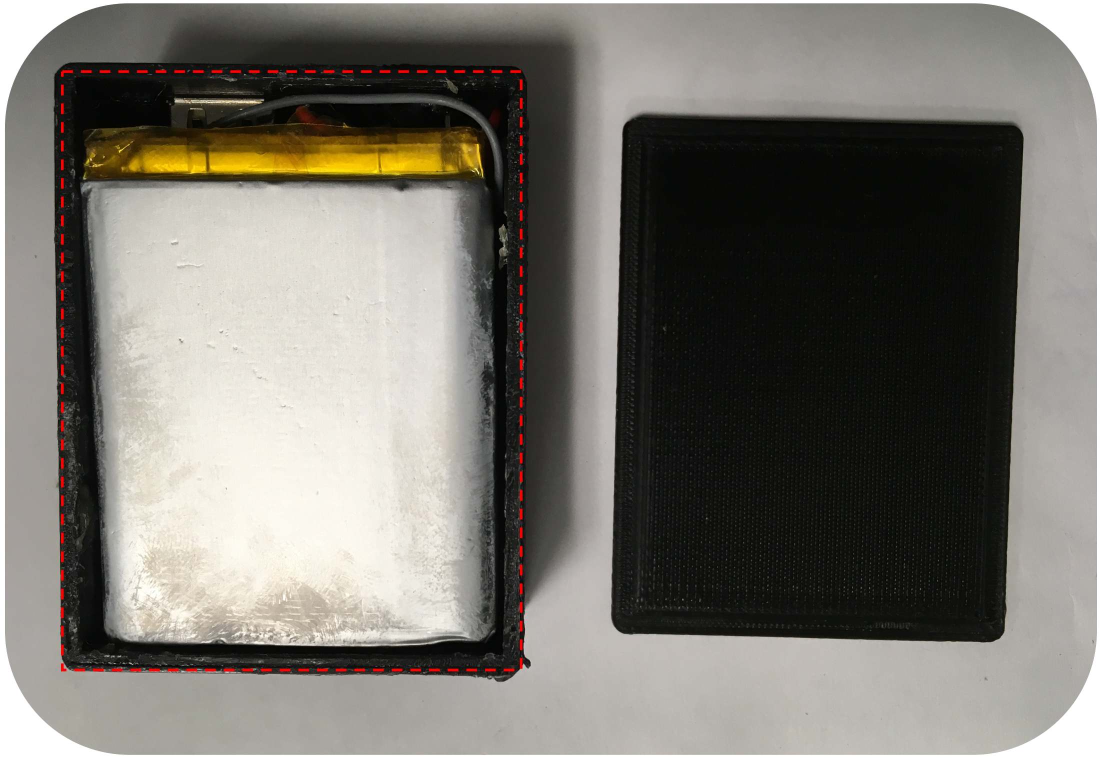

Apply super glue on the box's top surface as shown and place the cover.

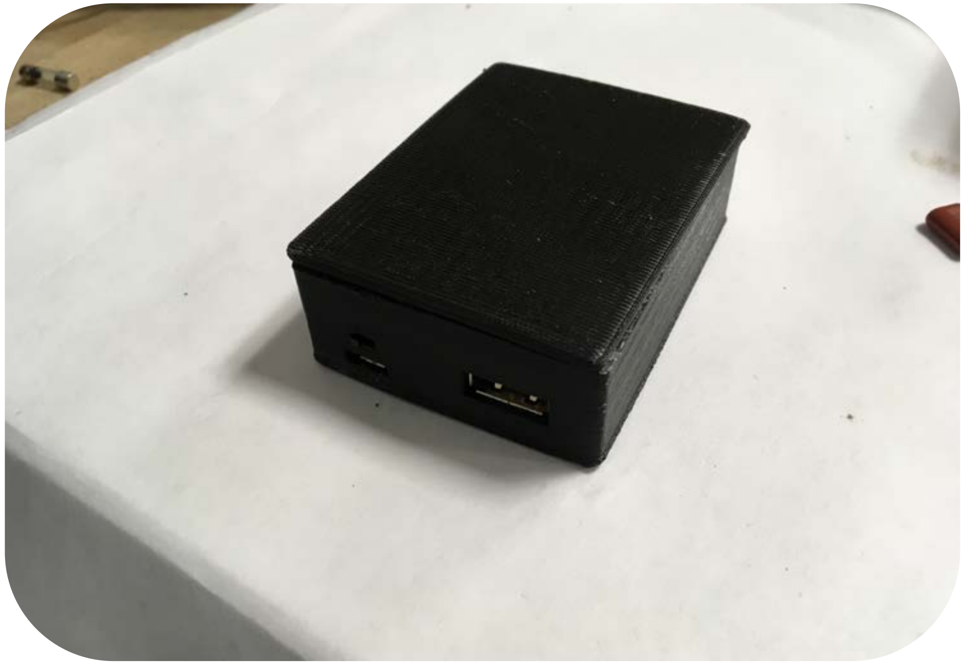

Place some weight on the cover or clamp it and wait 5 minutes for the super glue to cure.
