# Supplementary material for: An open-source, wireless vest for measuring autonomic function in infants
Source: Behav Res Methods. 2020 Apr 24;52(6):2324–37. doi: 10.3758/s13428-020-01394-4 (PMC7584781; doi:10.3758/s13428-020-01394-4)

An open-source, wireless vest for measuring autonomic function in infants:

Fabrics and Materials

Table of Contents:

02 – Materials, Tools & Equipment
03 – Assembling the Fabric Casing of the Vest
38 – Making the Removable Electrodes
46 – Integrating the Vest

All templates can be found on the Open Science Framework
(osf.io/24gp5)

MATERIALS, TOOLS & EQUIPMENT

MATERIALS
- 4-way stretch fabric
- Bar soap/silver sharpie
- Black sharpie
- Fabric pins
- Thread
- Optional: Aleene's Super Fabric Adhesive
- Adhesive Velcro
- Snap buttons (Caftown snap kit)
- Elastic straps
- Parachute buckles
- Silicone rubber sheet
- Ferrous fabric

TOOLS & EQUIPMENT
- Scissors
- Utility/box knife
- Sewing machine with bobbin and needle
- Straight edge
- Drill
- Optional: rotary fabric cutter

ASSEMBLING THE FABRIC CASING OF THE VEST

Before making the fabric portion of the vest, make sure to finish the rubber portion of the vest. Cut a piece of black 4-way stretch fabric roughly 60” x 20” (a piece that is slightly bigger than the finished vest size). 4-way stretch fabric is used to provide maximum comfort while applying consistent pressure to the subject to achieve maximum contact between sensor and body.

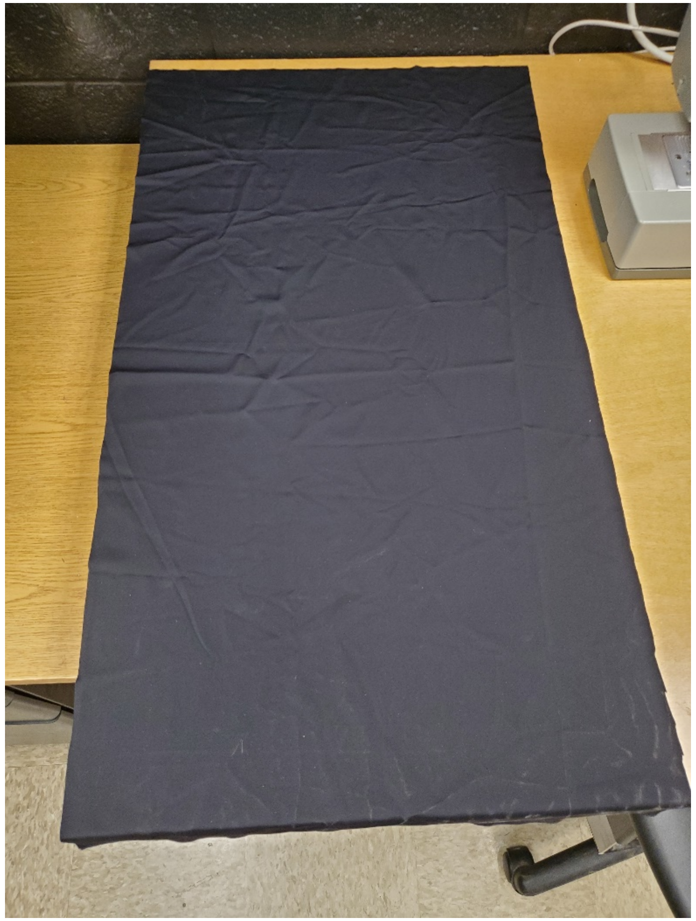

Fold the fabric to make 3 layers to a finished size of 20” x 20”. Orient the folded side to the bottom closest to you.

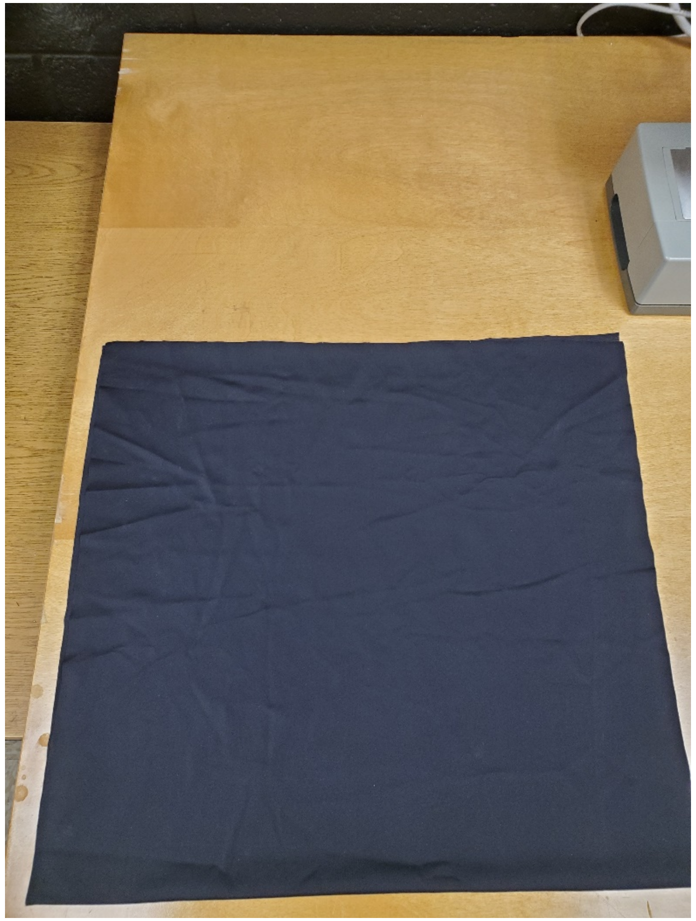

Place the Master Template on top of the folded fabric, making sure the bottom of the template touches the folded part of the fabric. Using a piece of bar soap or silver sharpie, trace the outside outline of template. If the paper template wants to curl, place weighted objects on them to flatten the template against the fabric.

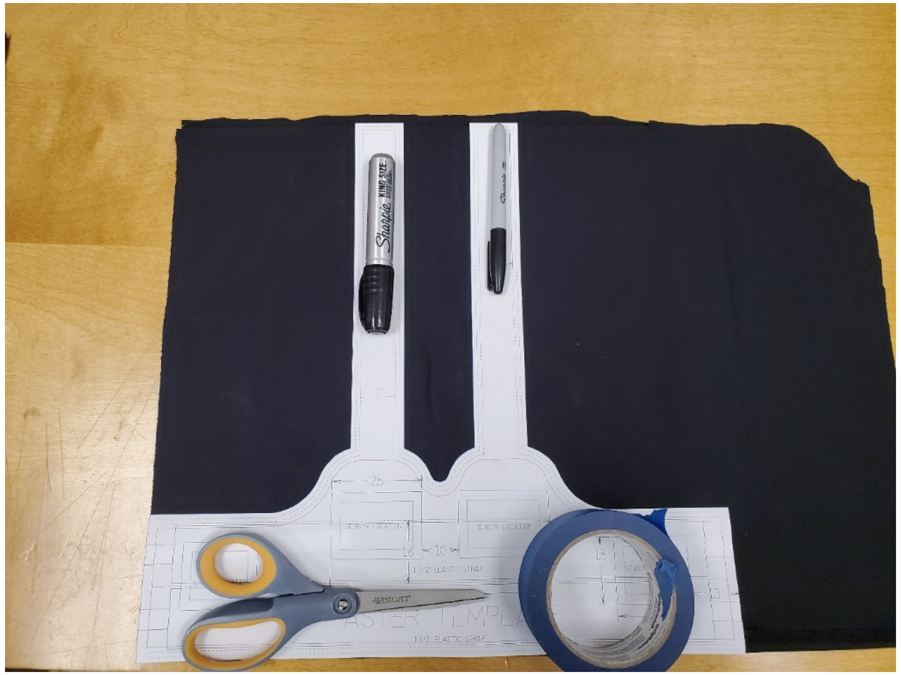

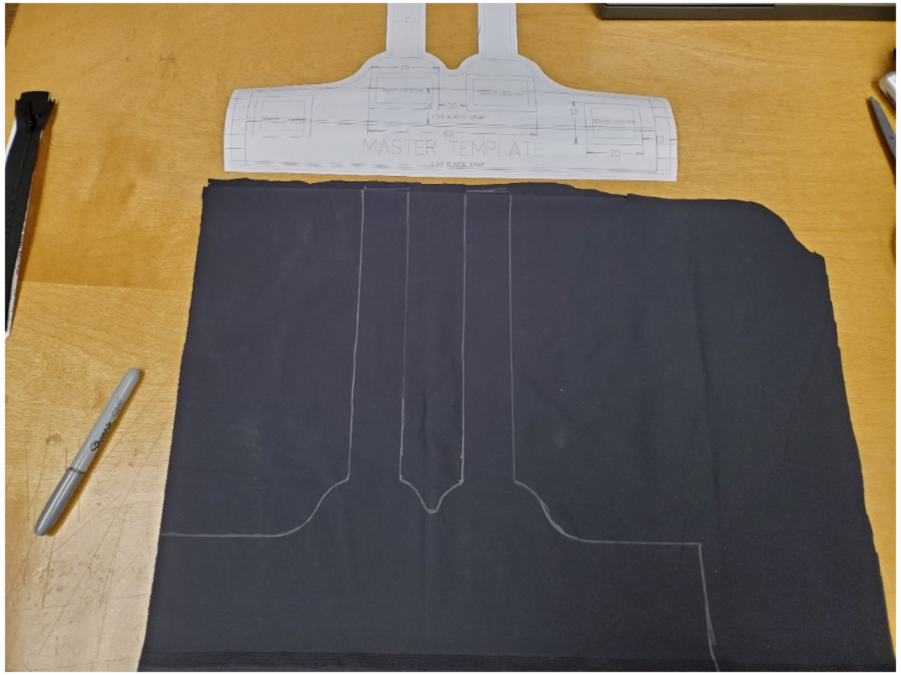

Before cutting out the fabric, use fabric pins to pin the folded piece of fabric together. The pin locations should be just to the inside of the traced outline. This will help ensure an even cut to both the top and the bottom of the vest. This will also help when sewing it together.

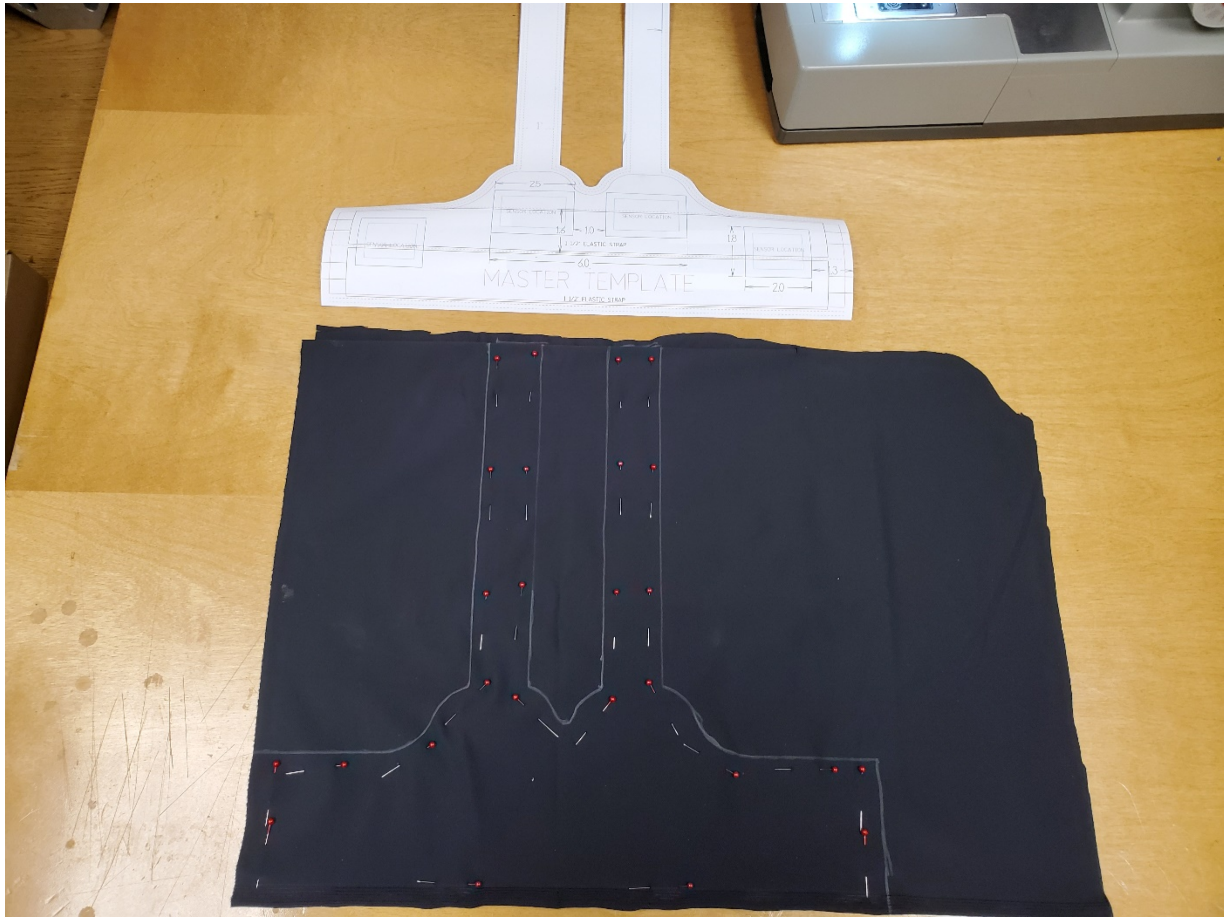

Cut the fabric on the traced outline.

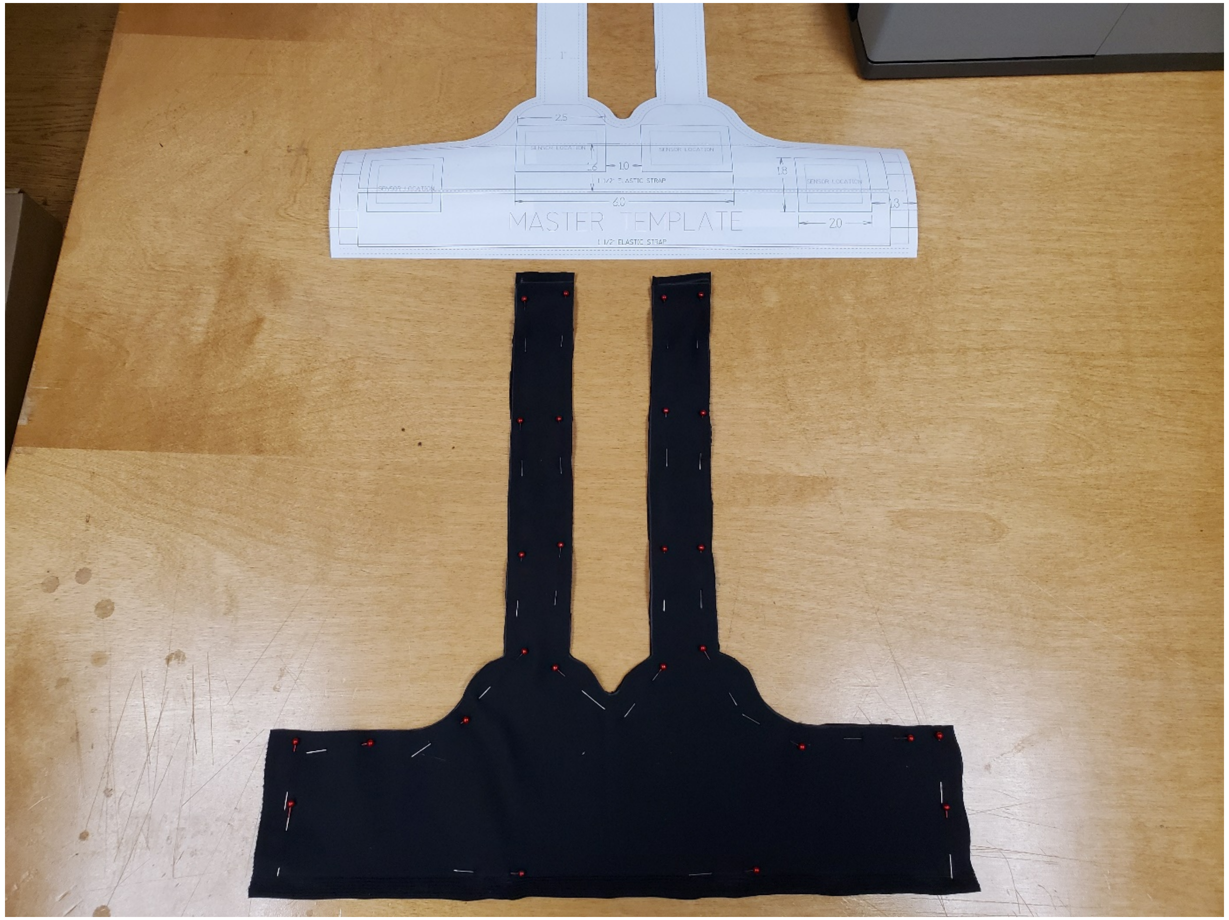

Cut along the folded portion of the bottom of the vest to separate the layers along the bottom. You should have 3 total individual layers.

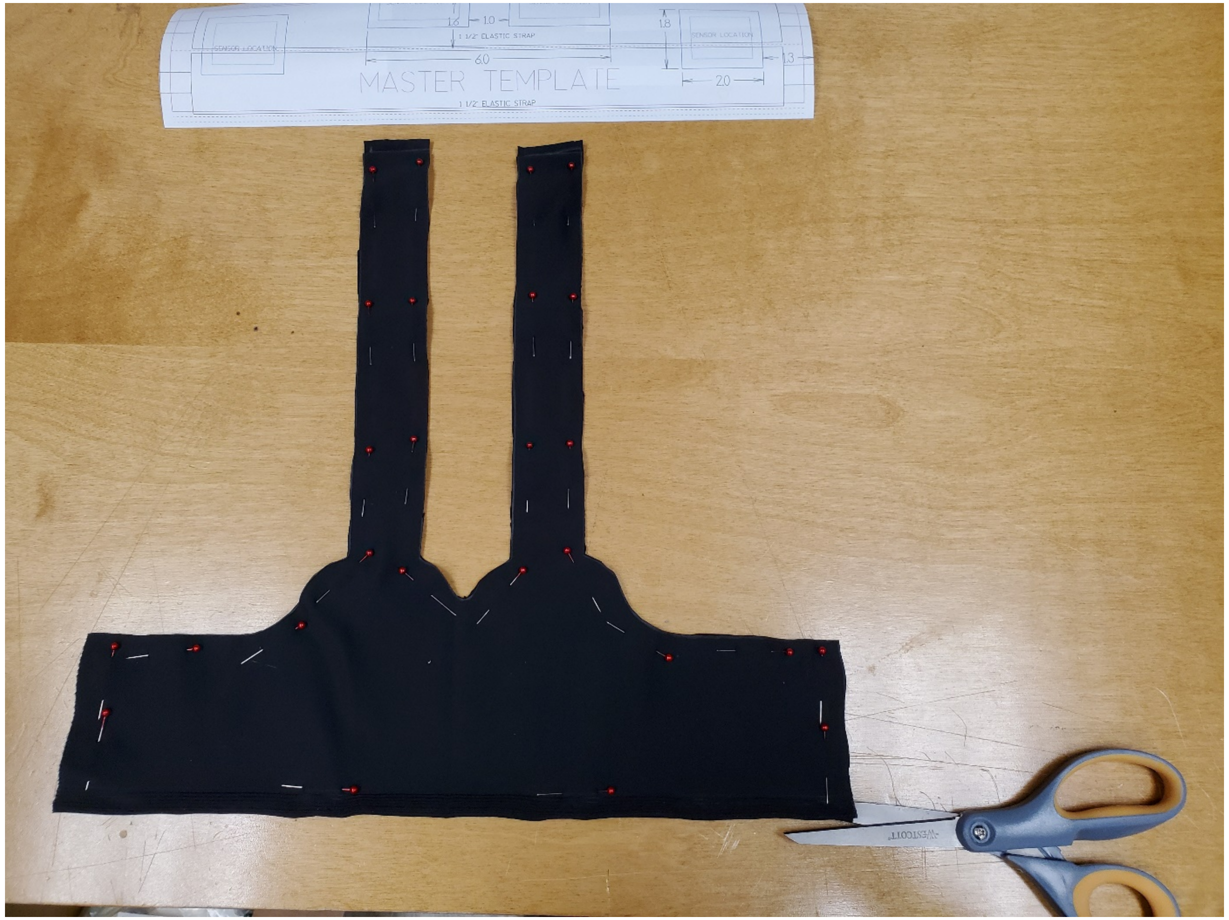

Unpin the 3 layers and note the orientation (the top vs. back of each individual layer and which layer is on top, middle, and bottom) so when you sew the layers back to each other, they are in their original orientation as when you initially cut them. You can use tape and a marker to label the pieces. I used painters tape to label as shown in the picture, and additionally labeled them as #1, #2, and #3 (#1 being the top and #3 being the bottom).

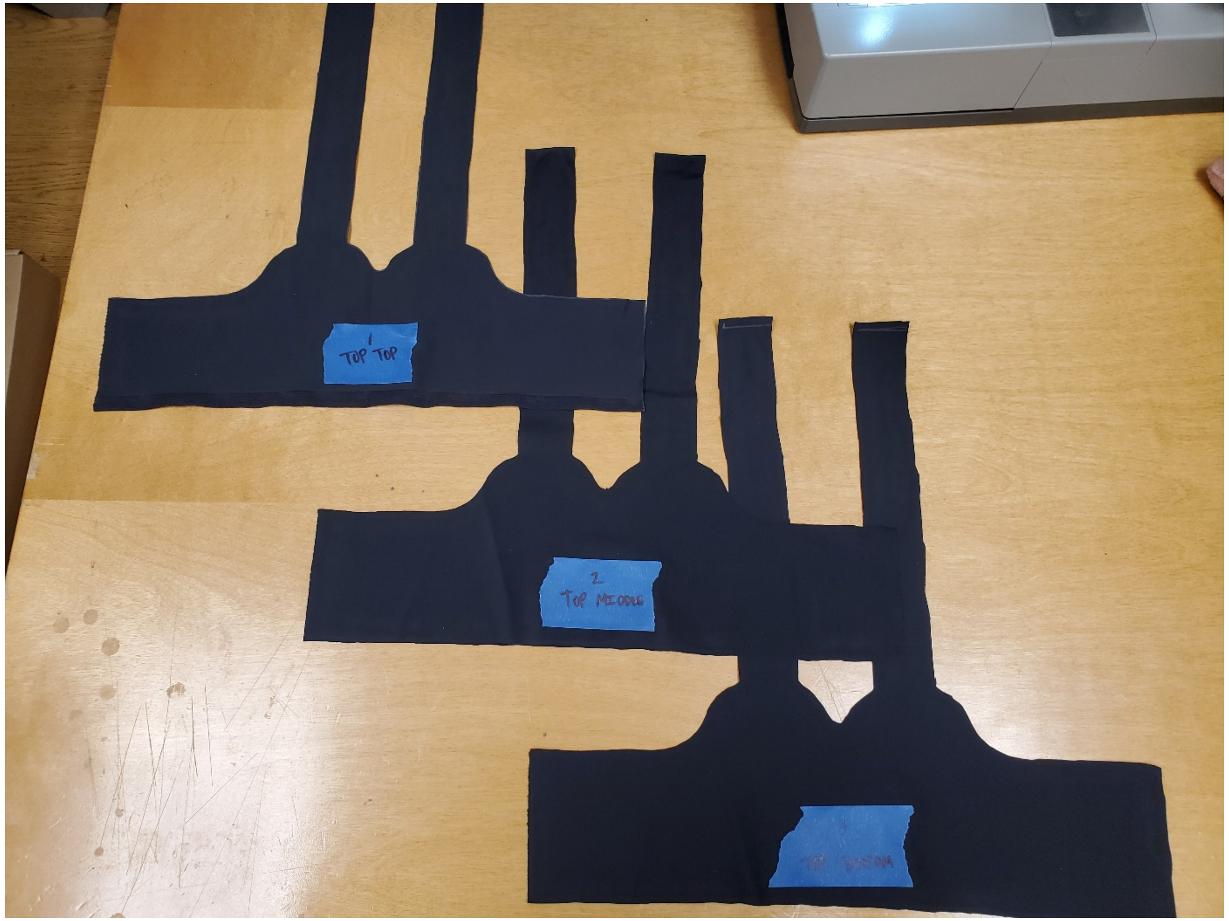

Get your sewing machine set up by placing thread, bobbin, and proper needle in place. For this vest, a black thread and bobbin was used along with a 90/14 sized needle. These materials were used in a Heavy Duty Singer sewing machine Model 4443/4452.

** If one does not have a sewing machine or does not want to sew, another option would be to use Aleene's Super Fabric Adhesive. In this case, please skip ahead to slide 12 to continue. You will reference the red dotted line on the Master Template and apply glue where the red dotted line is. In some applications, weight may need to be placed on top of the 2 glue parts to ensure complete contact from surface to surface. Allow time for drying before moving on to additional steps and ensure glue is fully dry. **

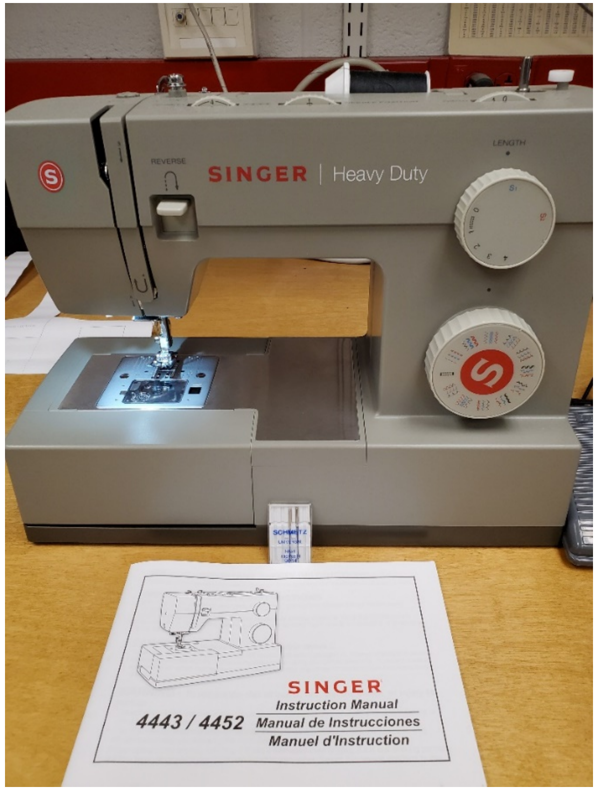

These instructions are for the Heavy Duty Singer sewing machine Model 4443/4452. They may not apply to your machine. The top knob should be set to a length of 3 while the stitch setting should also be set to the third position. This stitch setting is meant to be used for stretchy fabric material and works well to strengthen the stitch. If you do not have this setting on your sewing machine, any setting will work but you will want to make sure you make adjustments to your machine, so it sews a tighter more closely spaced stitch.

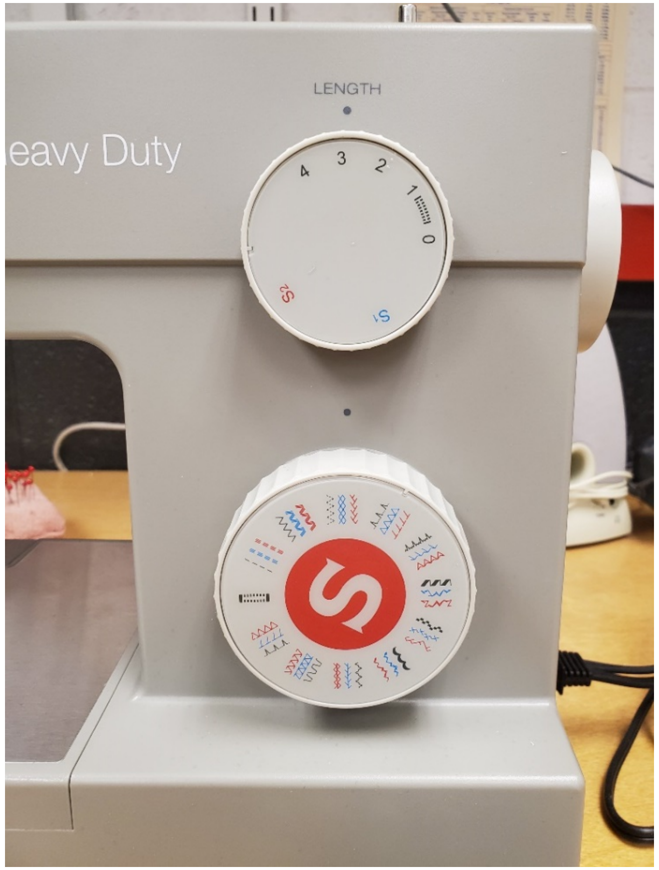

Reference the Velcro and Strap Template and proceed to cut Velcro strips to the size represented on the template using the 1 ½” wide Velcro.

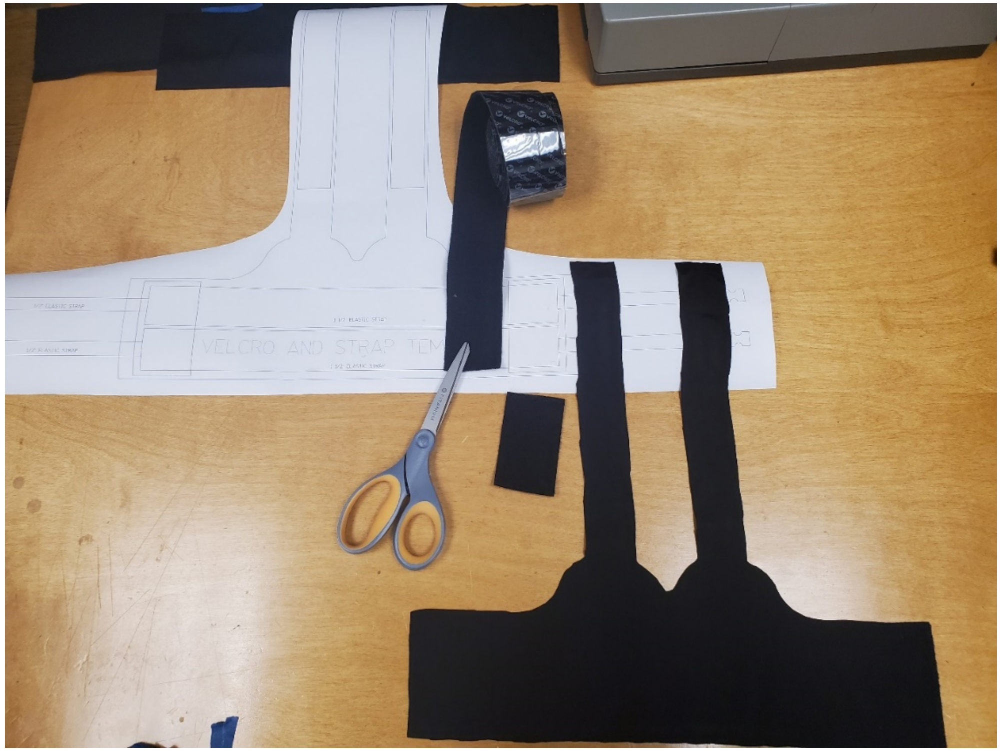

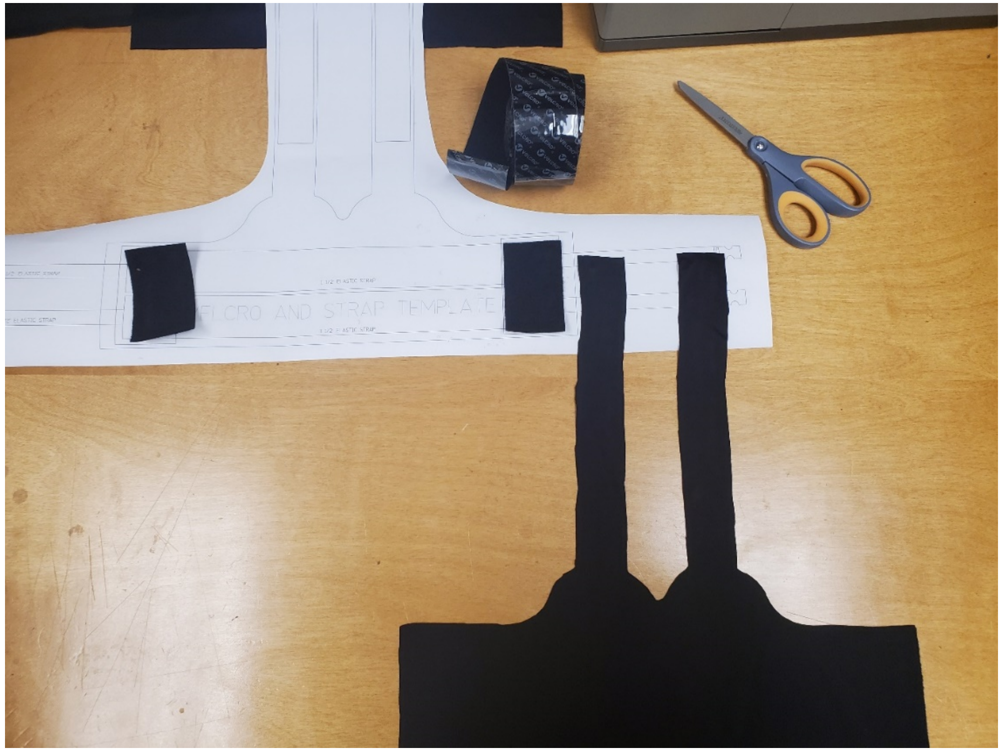

Locate your bottom layer of fabric (#3) and flip the fabric over to the back side and sew or glue the pieces of 1 ½" wide Velcro in the locations represented in the picture and on the Velcro and Strap Template.

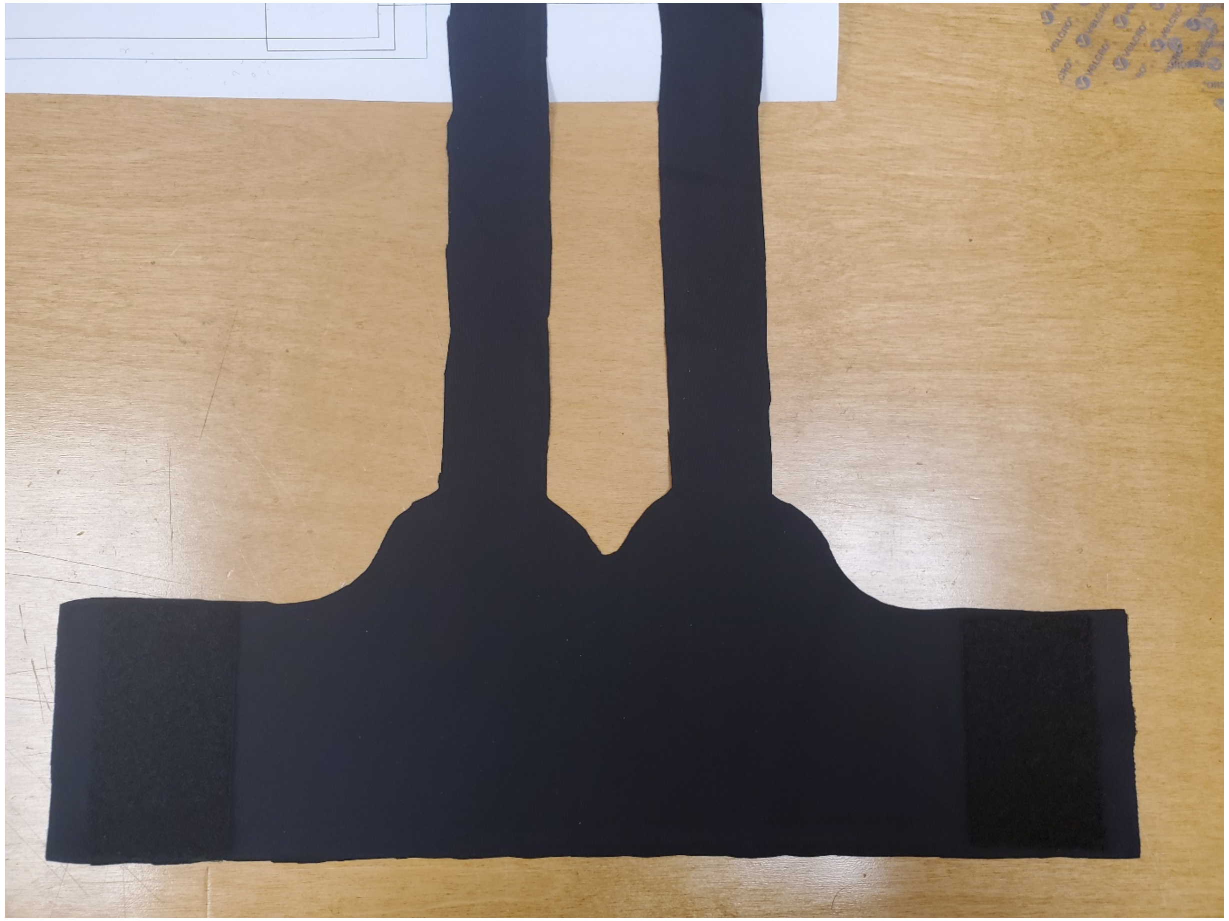

Locate the sewn middle layer of the vest labeled #2. On the Master Template, identify the 3 black circles. These circles represent the snap locations. Install the snaps (3 female ends and 3 male ends). The first half of the snaps (3 male ends) are going to be installed on the middle layer of vest fabric labeled #2. There are easy to follow directions in the snap kit showing you how to install them. You can use the awl in the snap tool kit to poke holes in the Master Template to transfer locations on to the fabric. The second picture represents what it should look like after installing the snaps.

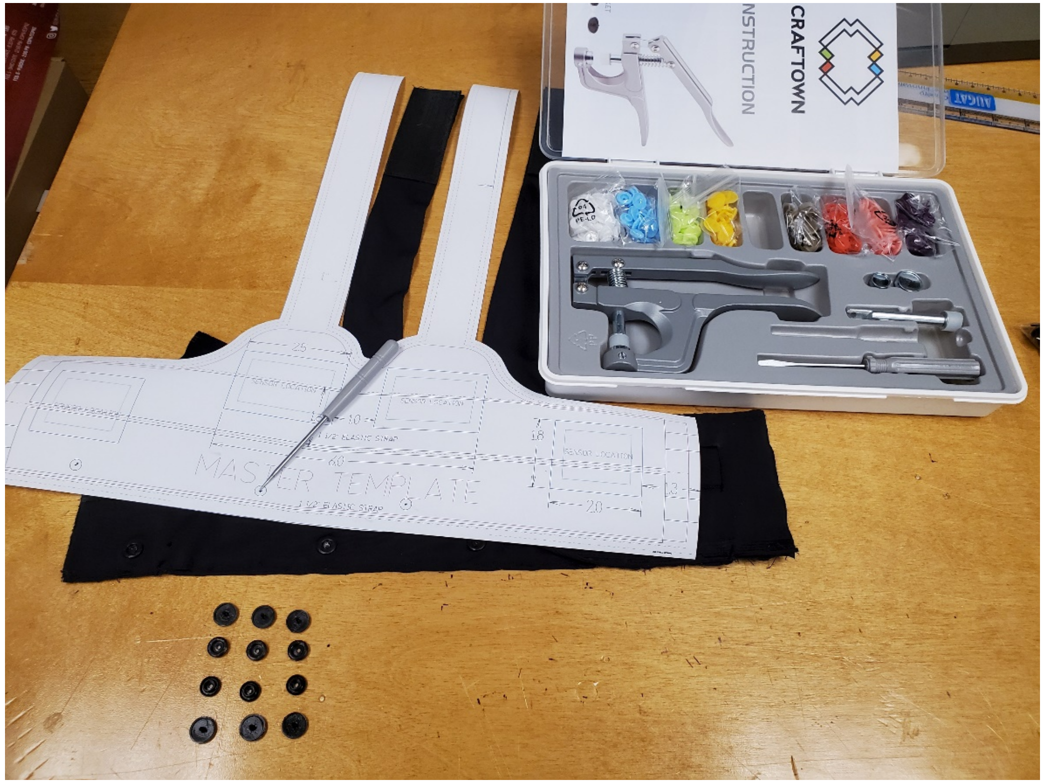

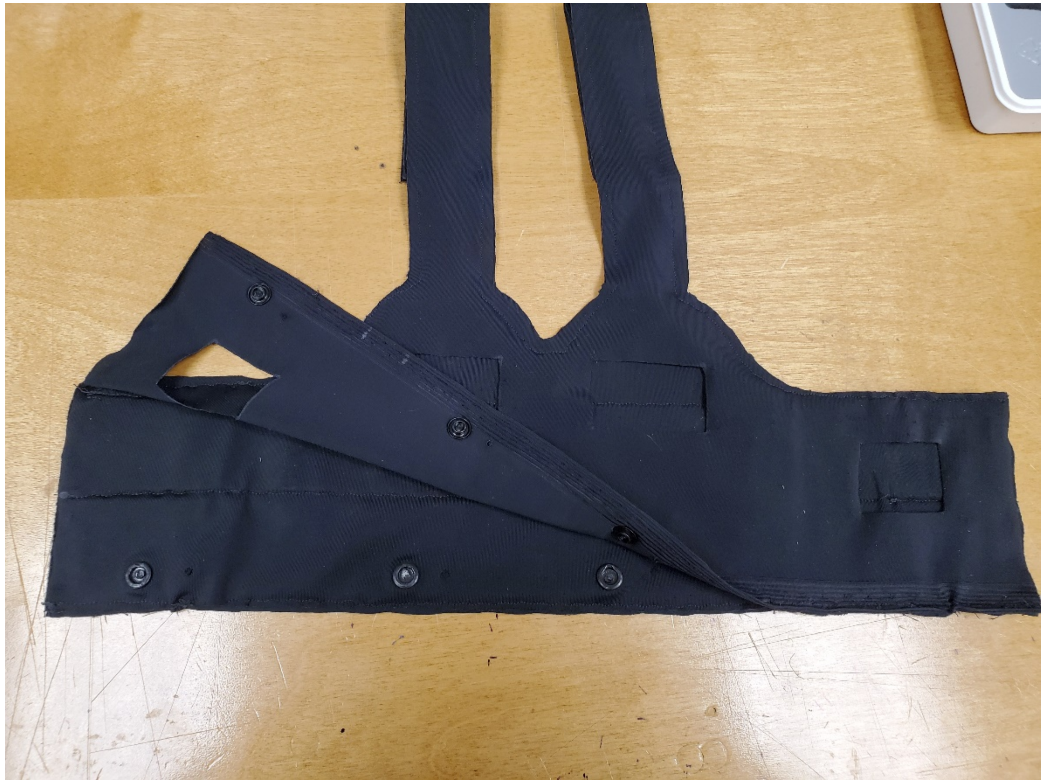

If gluing: The bottom and middle layer can be glued together. Keep the middle and bottom layer separate. Refer to the Master Template to locate the red dotted line. You can remove the label on the bottom layer at this point. Place the bottom layer on the table and run a glue line with a medium sized bead on the bottom layer of fabric in the same location as the red dotted line shown in the Master Template. Place the second layer over the third layer and run your fingers along the glue line to ensure contact between both layers. The proceed to slide 19.

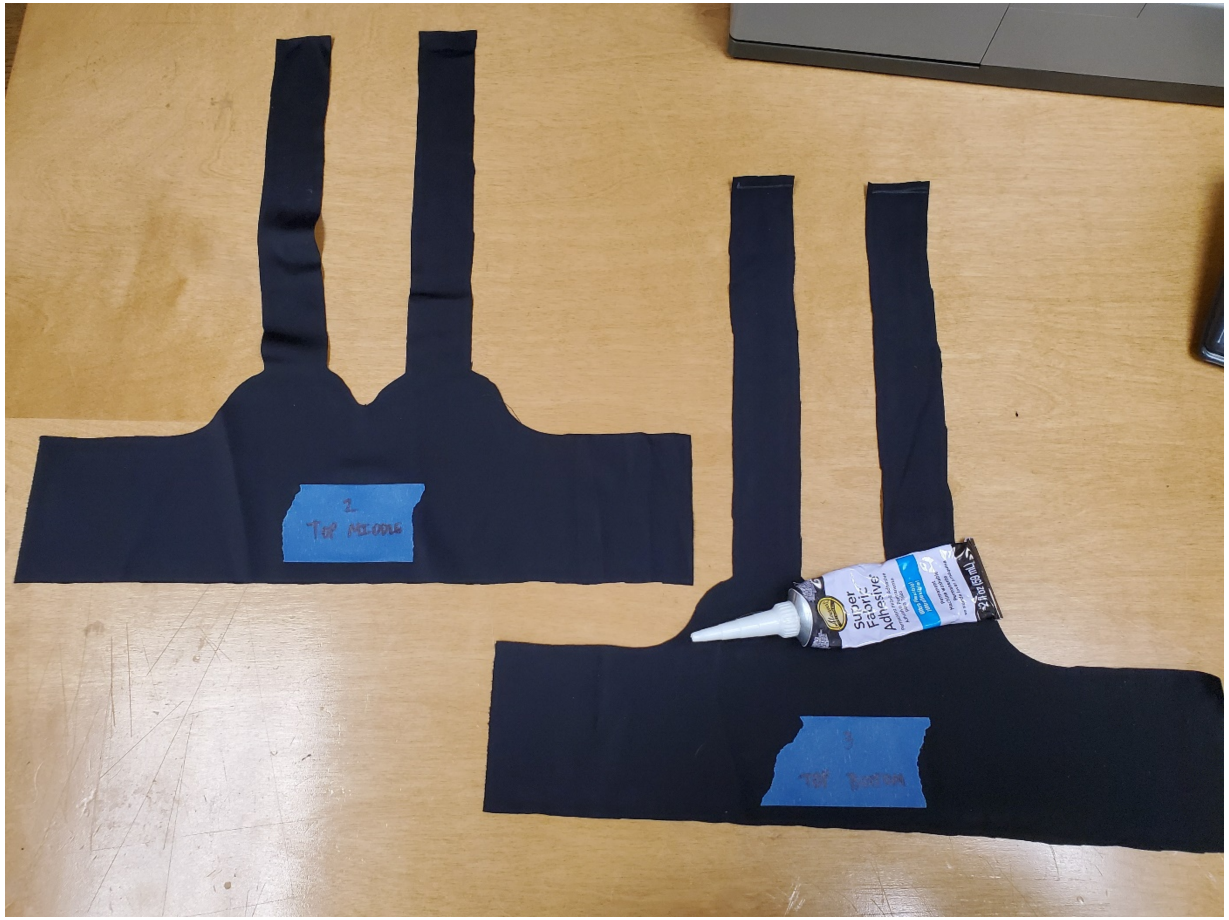

If sewing: Repin the middle and bottom layer together and refer back to the Master template. You can remove the label on the bottom layer after repining.

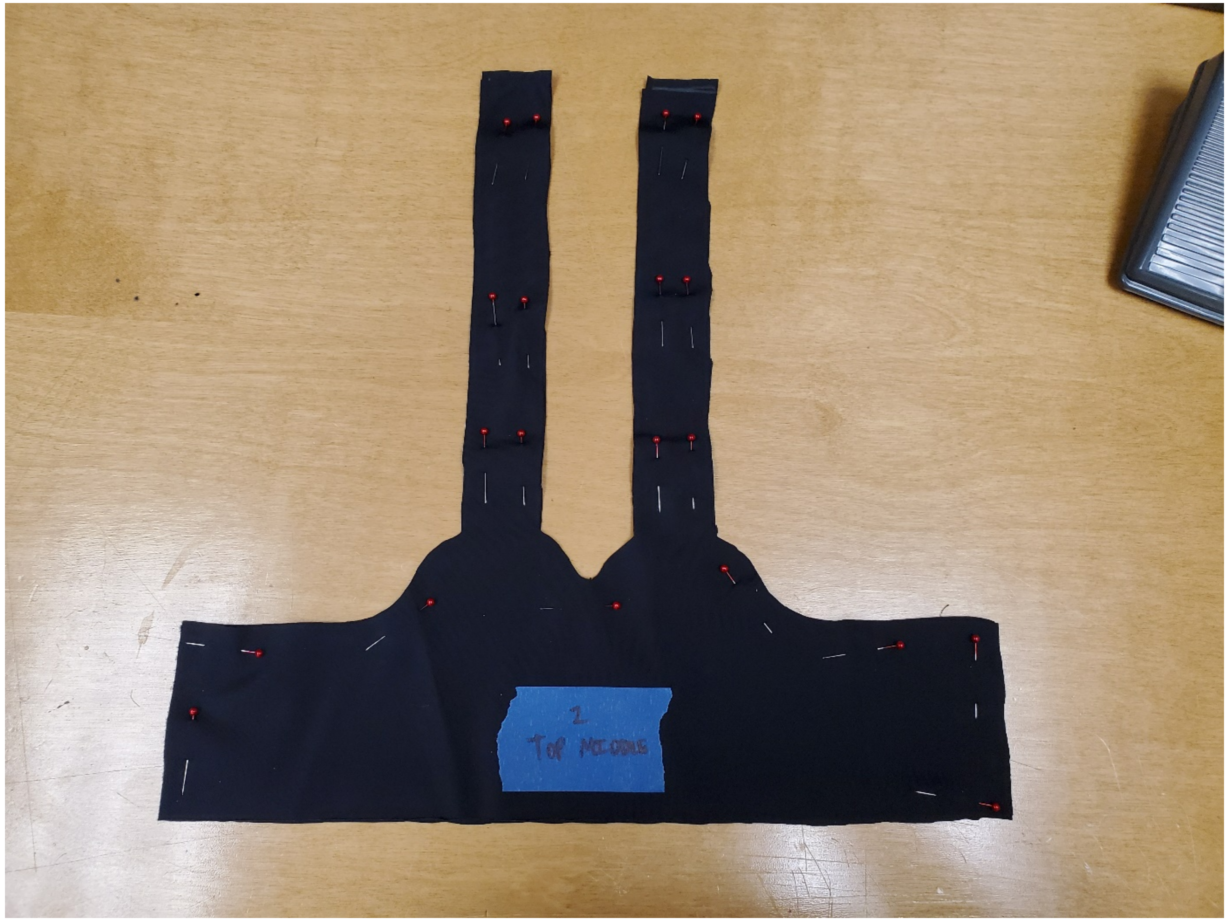

The spacing and location of the stitches should match the dotted lines on the Master Template. To ensure that middle stitches are in the proper location, place the Master Template over the vest and make a mark with a silver sharpie where the stitch needs to go on the far right of the vest as seen in the picture. Make a mark on the left side as you did the right side. Connect the lines using a straightedge as shown in the second picture. Use black sharpie to make a line. A black sharpie will give you a visible guideline to sew on but not be so visible that it is noticeable on the final product. The delineated locations will ensure adequate space for 2 pieces of elastic strap to be placed through the fabric vest.

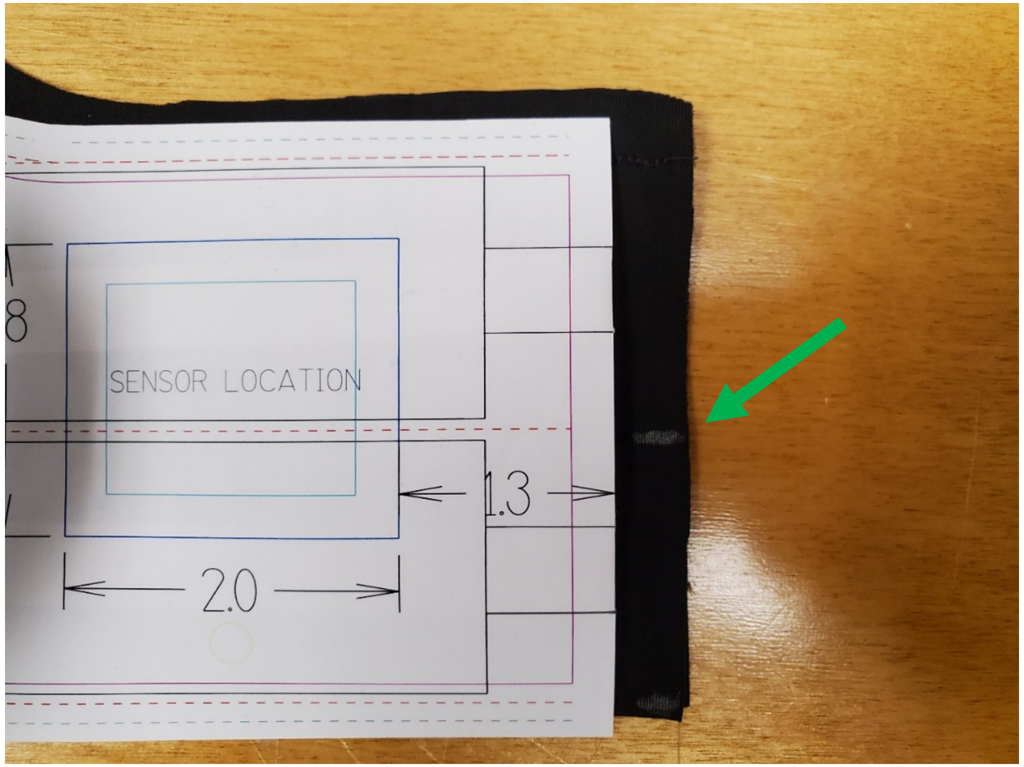

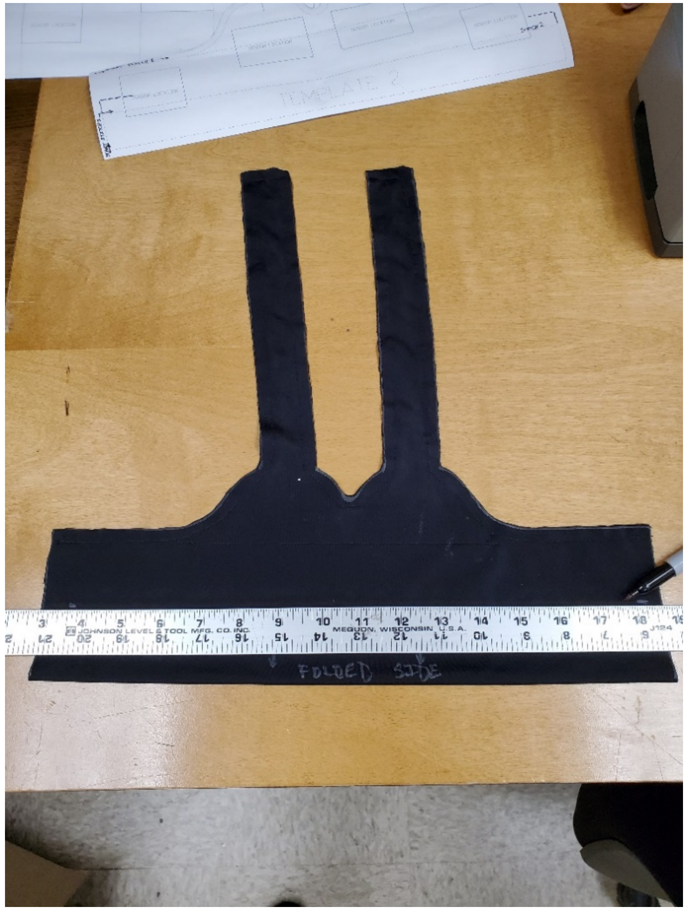

Sew along the red dotted line. Sew approximately a quarter of an inch from the outside as the dotted line shows on the Template. A good reference for ¼" is that you want the material edge in line with the edge of the foot (as shown in Picture). Remove the pins gradually as you are sewing and don't forget to backstitch on the beginning and then end of each stitch. Each sewing machine has a backstitch (reverse) button or tab. Using a backstitch will prevent any thread from coming apart in the beginning and the end of stitch.

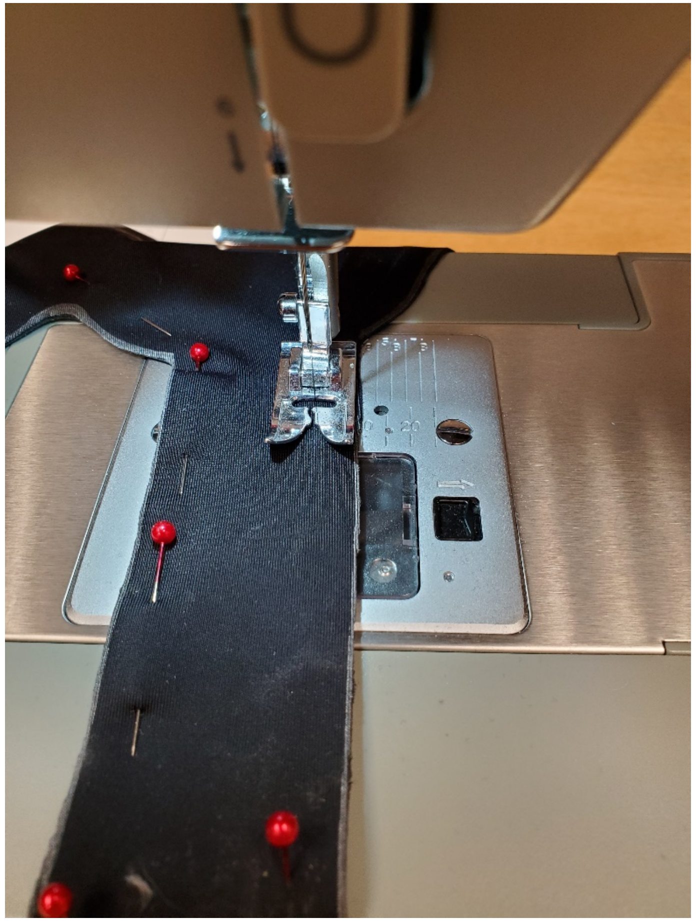

This is what the stitch should look like after sewing or gluing on the red dotted line.

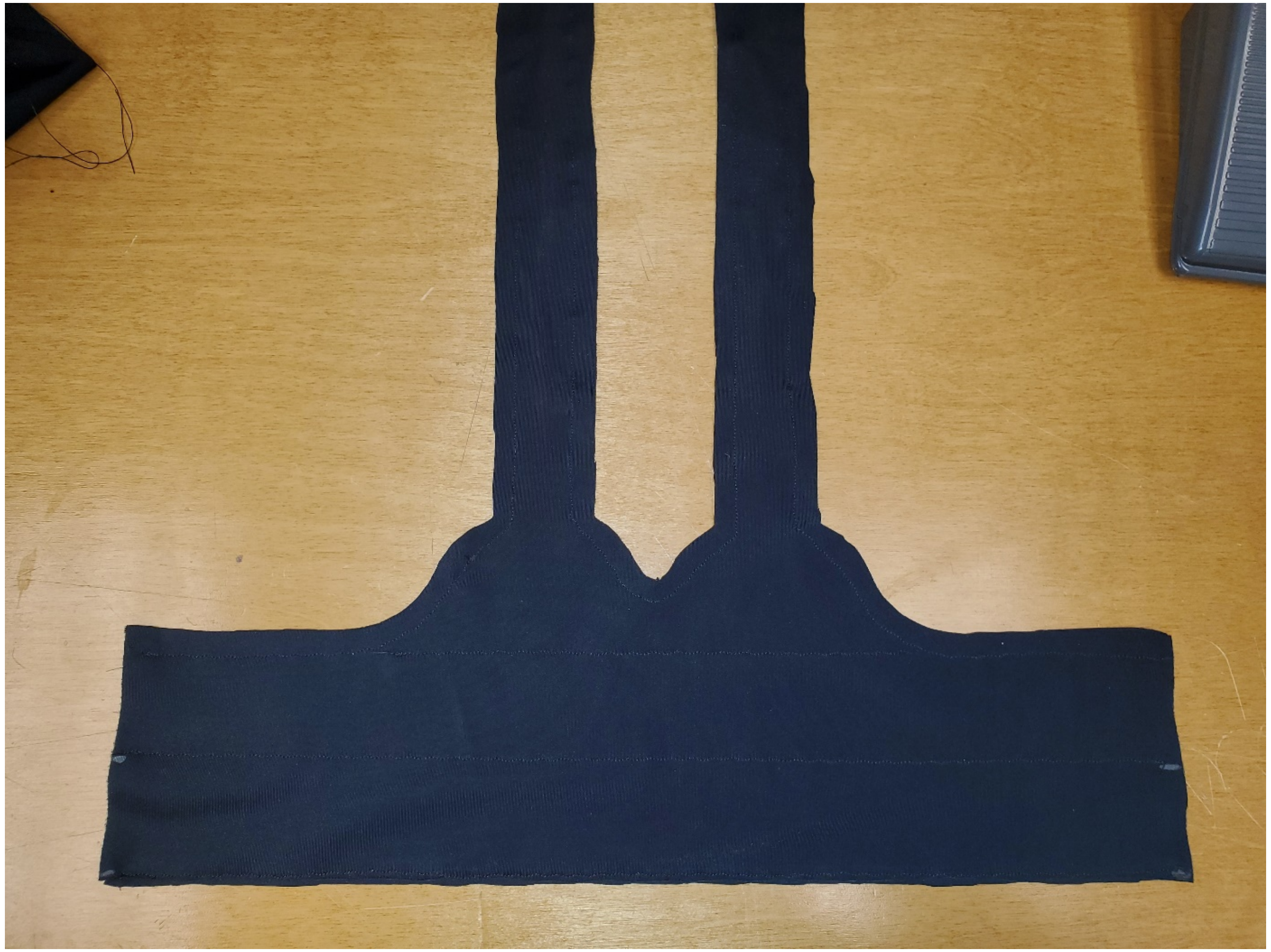

After the middle layer and bottom layer are sewn, the vest is now ready for the elastic strap to be installed. Reference the Velcro and Strap Template again and look for the label that says 1 ½” Elastic Strap. You can use the template to measure out your elastic to the size represented on the template.

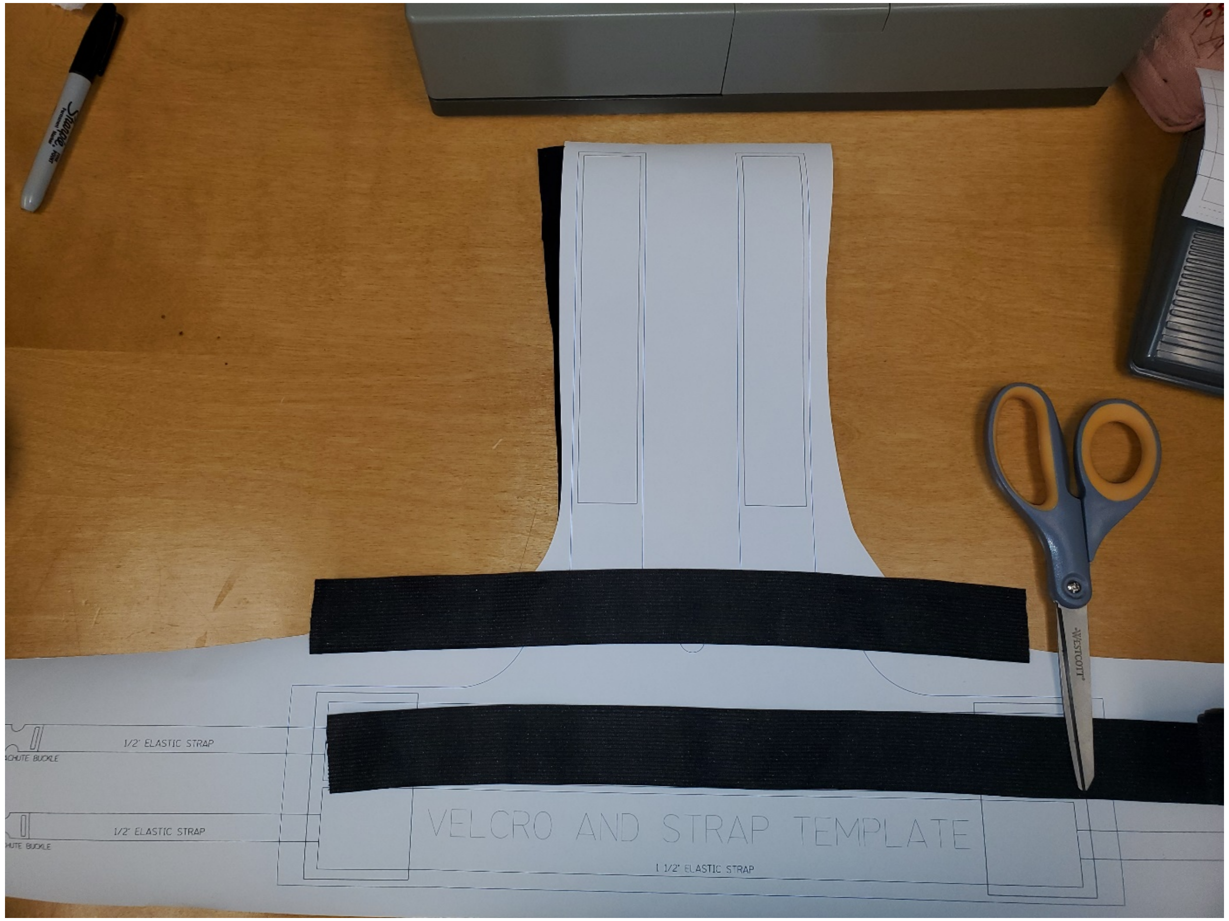

Reference the Velcro and Strap Template and look for the label that says ½” Elastic Strap. You can use the template to measure out your elastic to the size represented on the Template. Unfold the paper to get correct measurement. Note: Measure to the end of the buckle as shown in the picture.

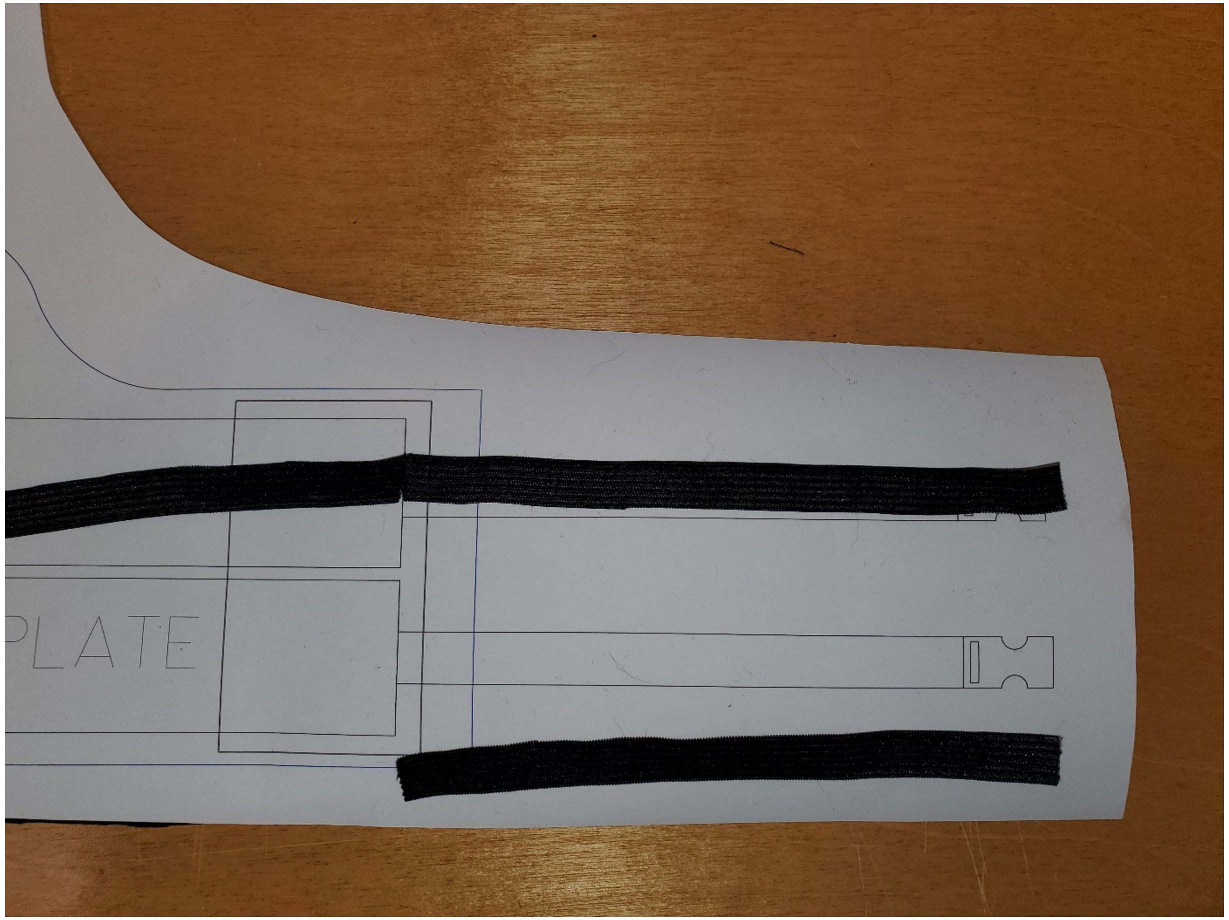

The ½” elastic strap needs to be sewn (or glued) on the 1 ½” elastic strap. Proceed to sew in a rectangular pattern securing the ½” elastic strap to the 1 ½” elastic strap as represented in the picture.

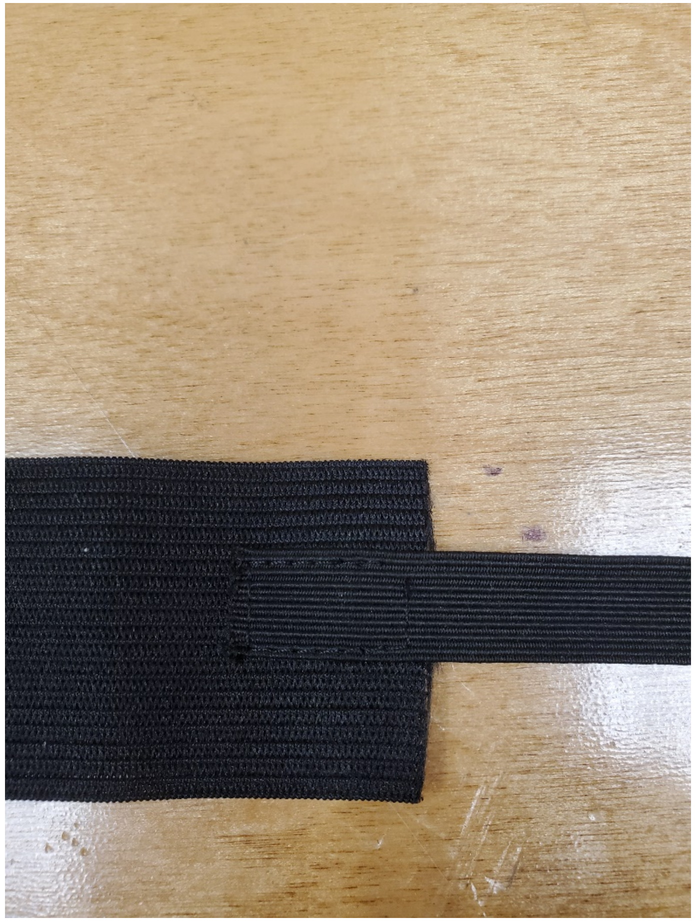

Proceed by sewing (or gluing) the other side of the strap the exact same way. After you finish sewing the elastic bands together, it should look close to the image represented in the picture. There should be a total of 2 straps going through the vest.

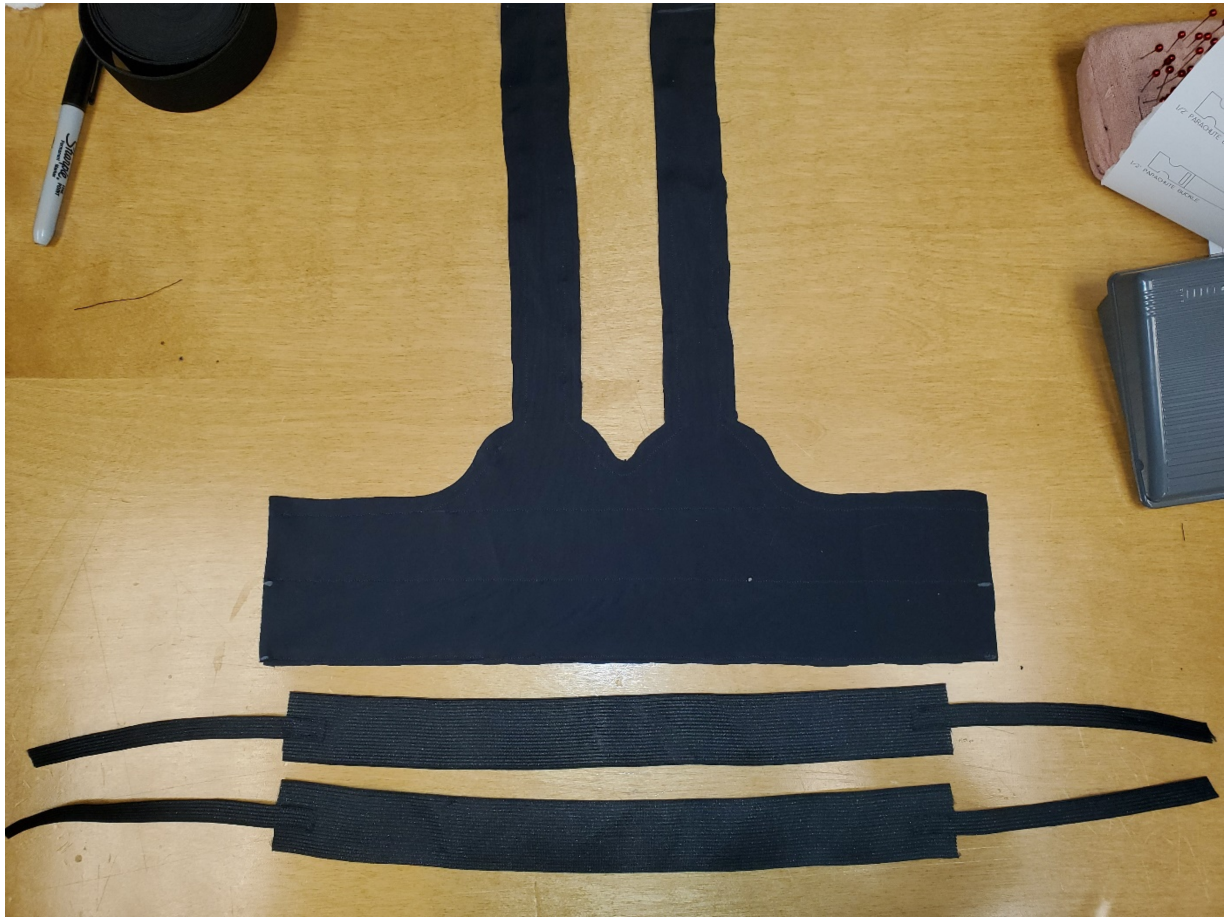

Velcro has a hook and loop side. The hook is the rougher/spiky part of the Velcro and the loop is the softer/fuzzy part of the Velcro. Cut 2 pieces of ½" velcro (loop side) approximately 7/8" long. Cut 2 pieces of ½" velcro (hook side) approximately ¾" long. One piece of each size will be sewed (or glued) as represented in the picture.

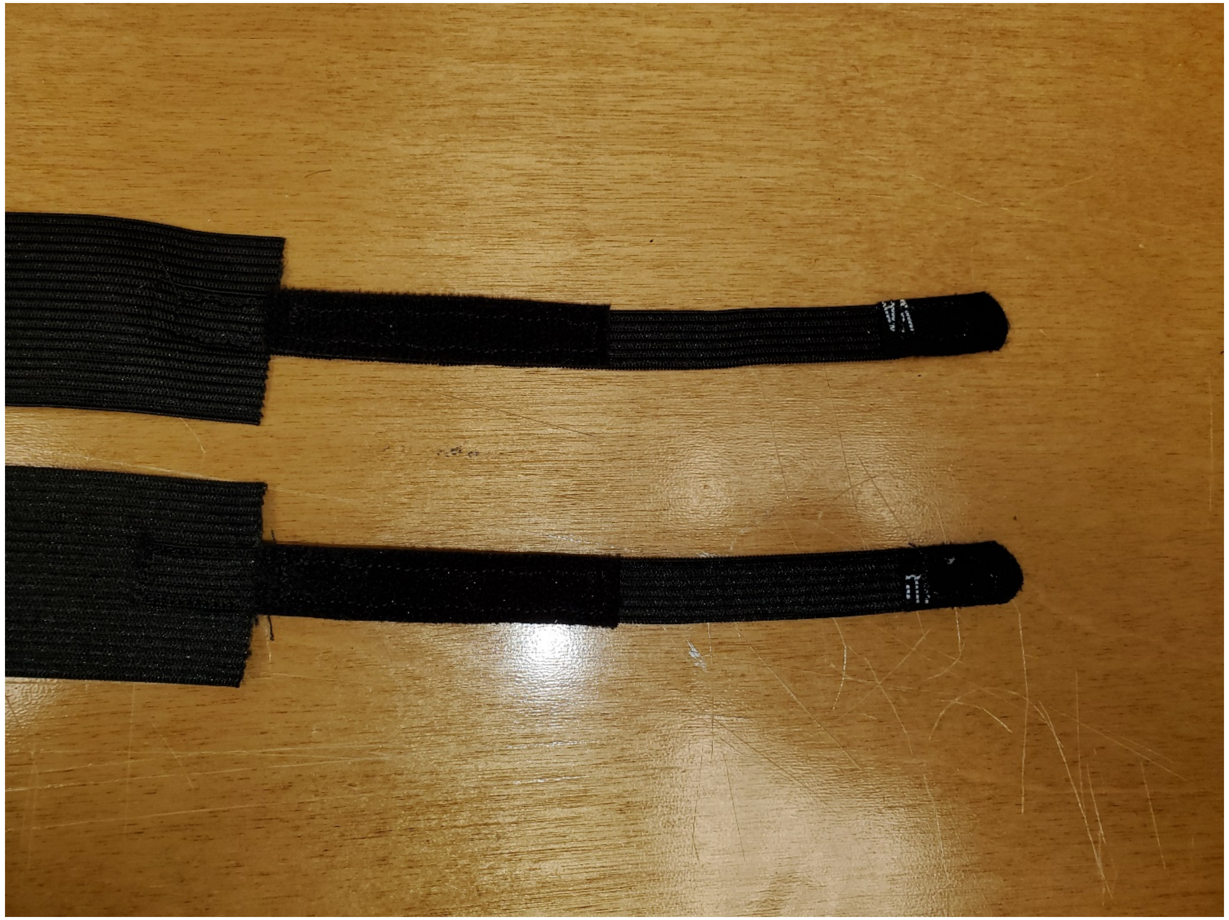

Slide elastic strap in the 1 ½" opening. To make this process easier you can insert something long into the opening and tie it to the strap. Pull the strap through until it is centered on the vest. It should look like what is represented in the pictures. After you've finished, set that portion of the vest aside.

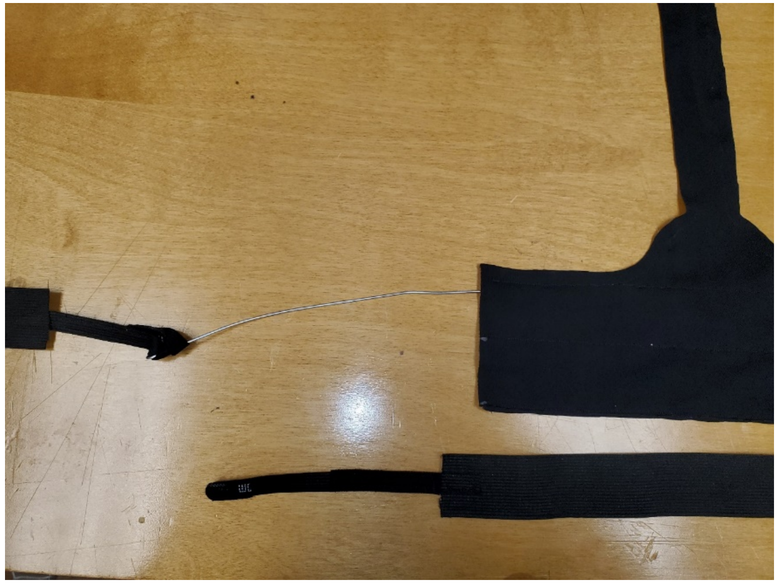

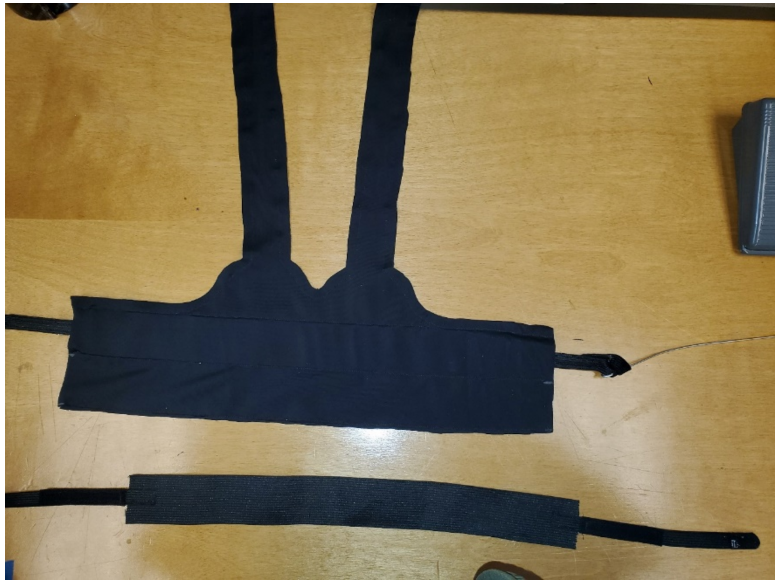

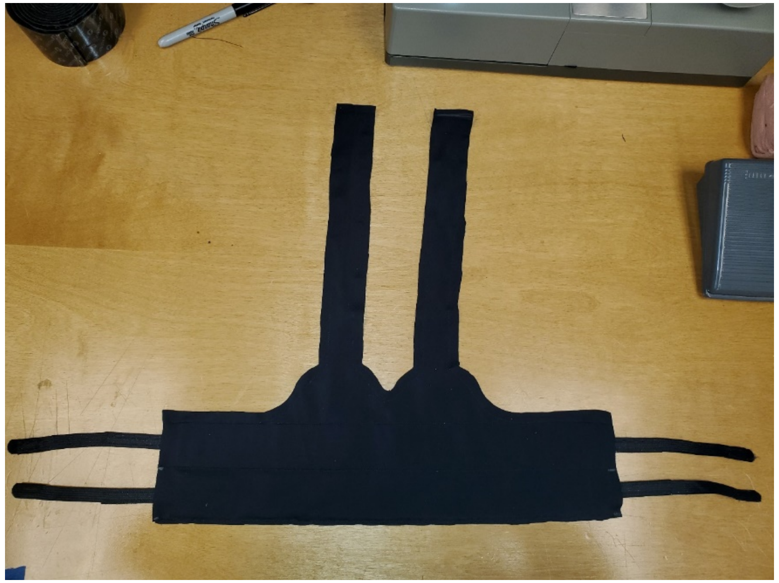

Cut out the Sensor Rubber Template by cutting along the pink solid line. Cut out the inner rectangles/squares along the bright blue dotted lines as shown in the picture.

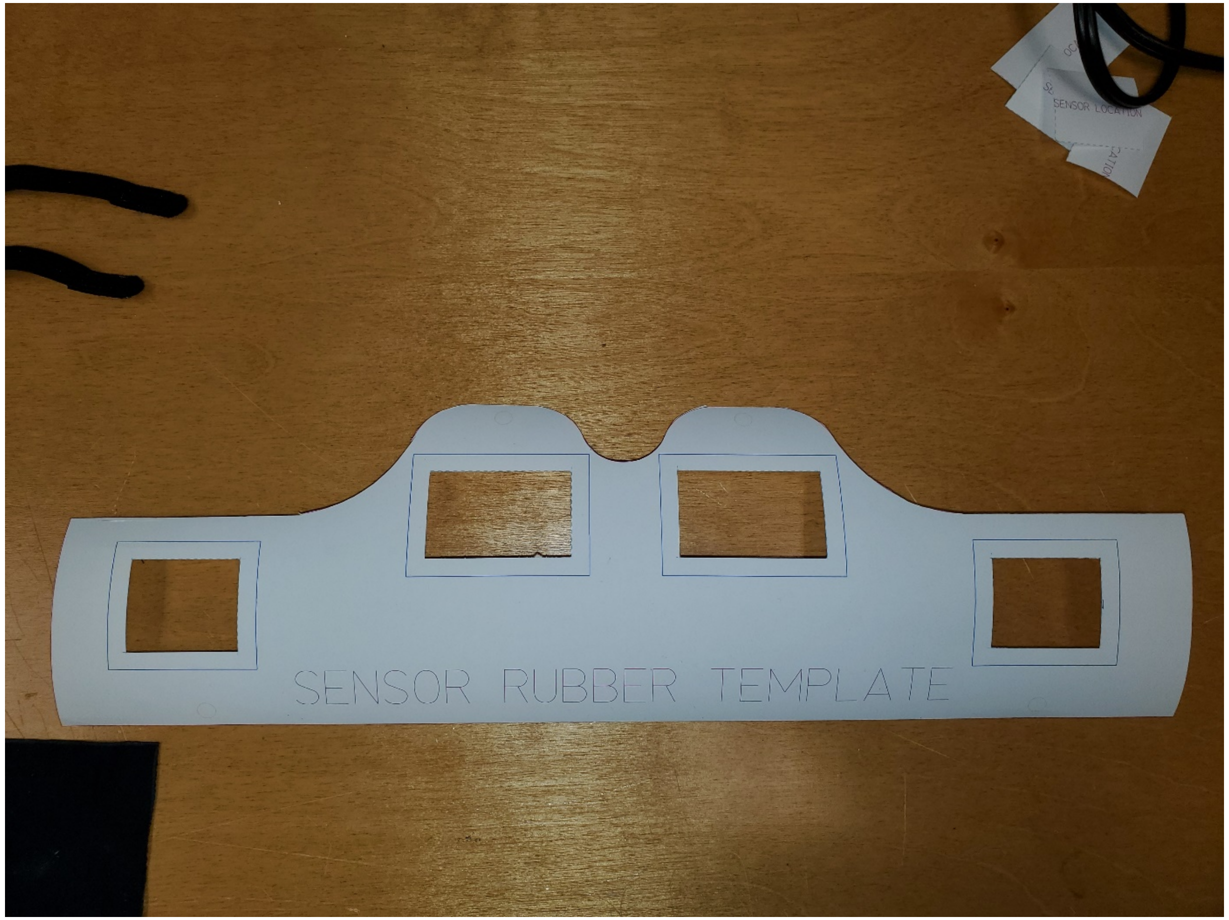

Locate the first layer of fabric that you set aside in the beginning of the project that you labeled #1 (the top of the top layer). Place the Sensor Rubber Template on the fabric and be sure to center the template left and right. The template should be a ½" from the bottom on the fabric leaving a border around the template as represented in the picture.

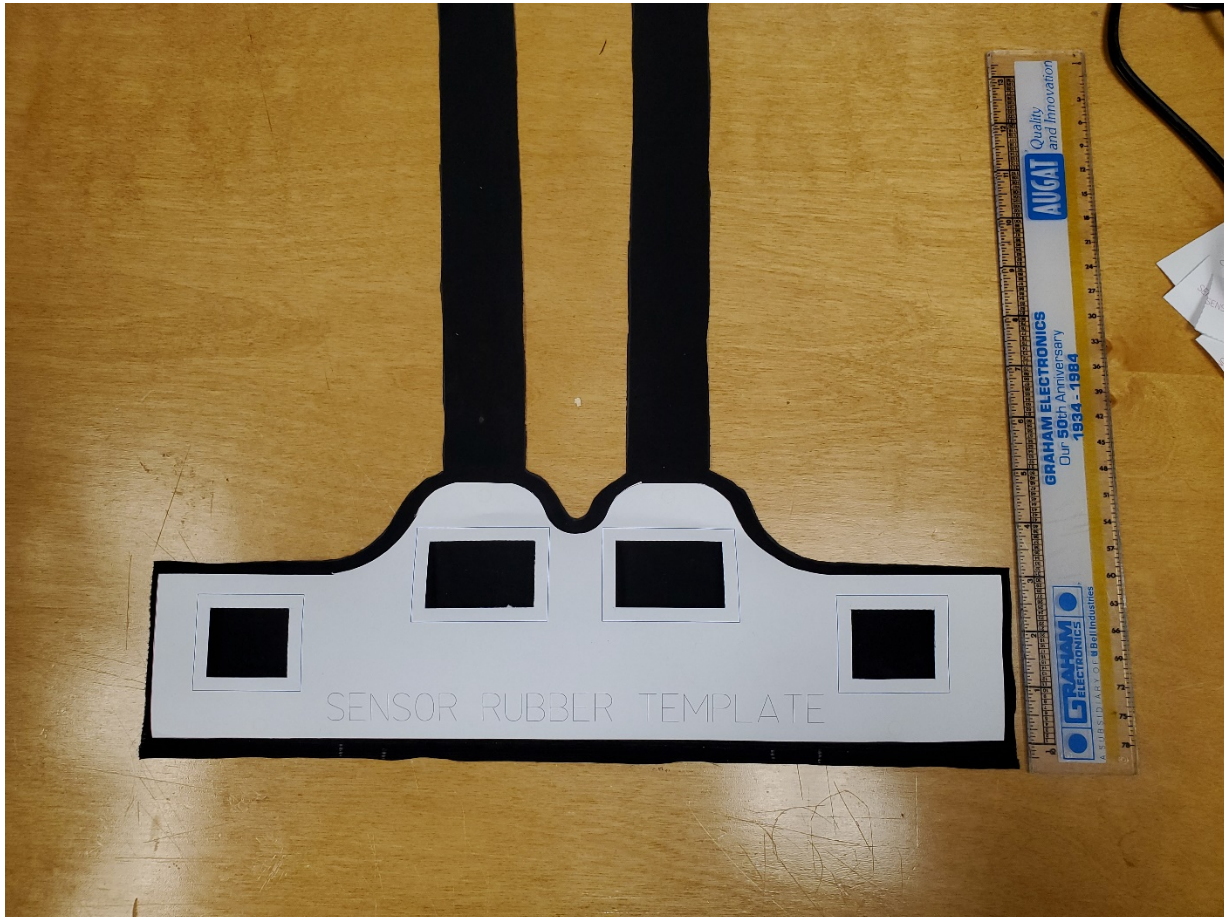

Proceed to trace the rectangles and squares onto the fabric using a silver sharpie.

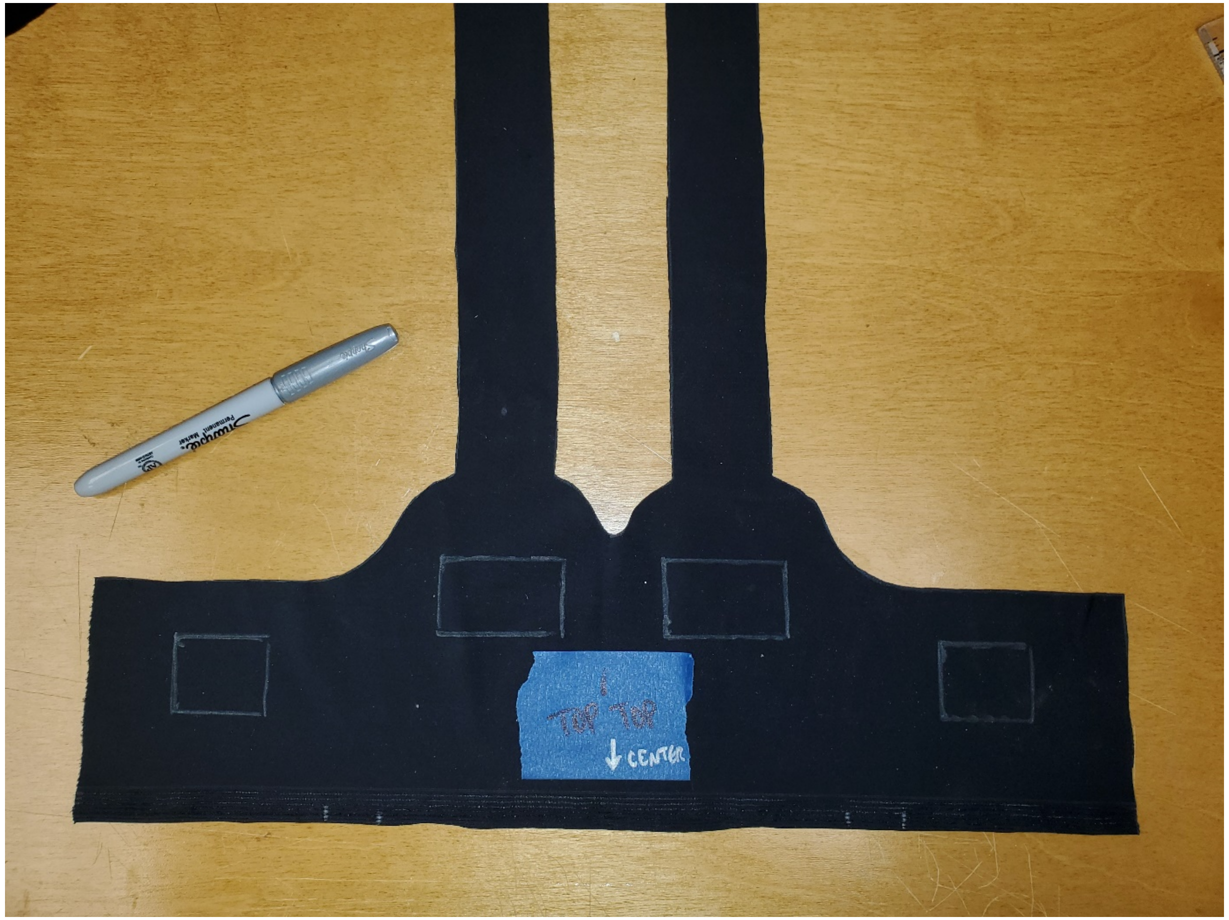

Cut out the rectangles and squares with a pair of scissors.

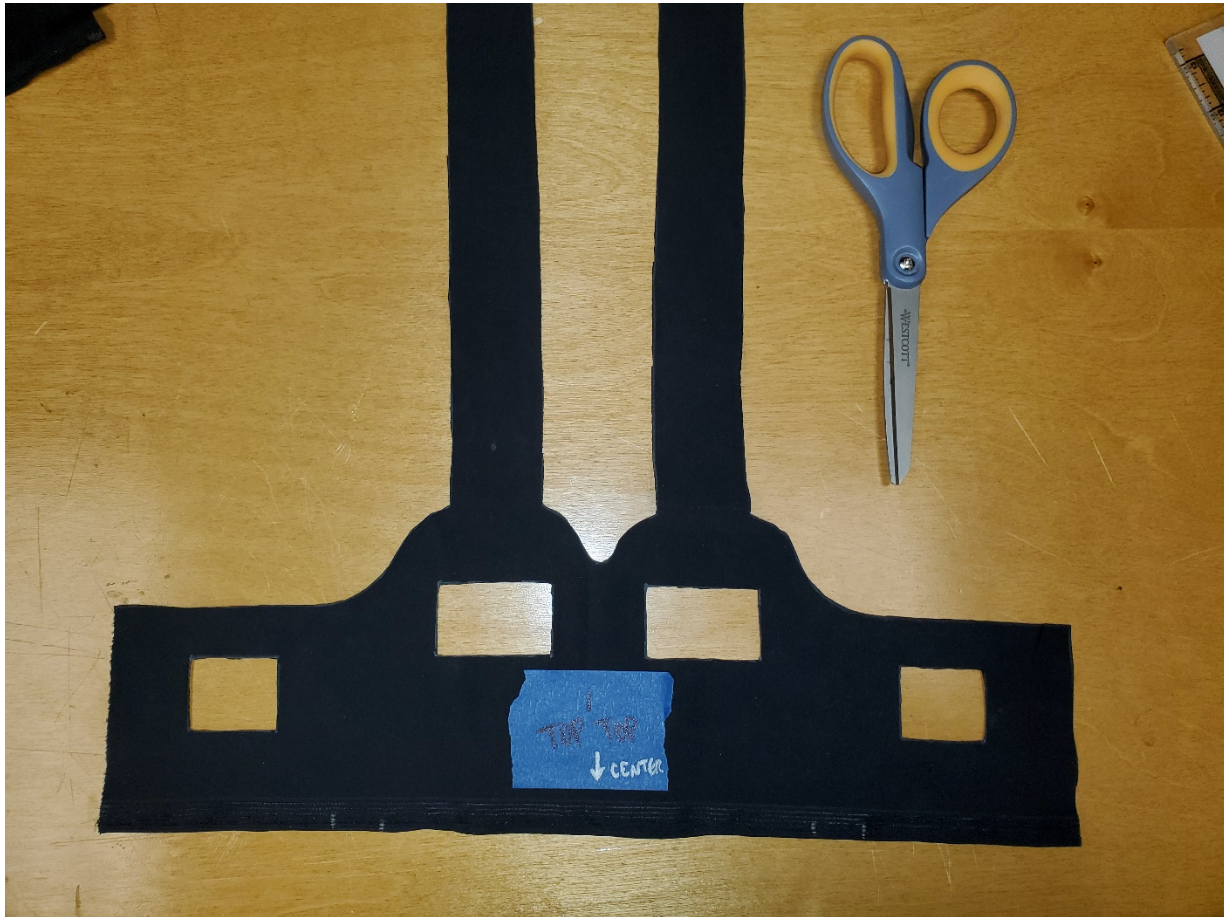

Locate the top layer of fabric and the Master Template. Install the other 3 female snaps in the top layer and flip it back over with top side facing up. It should look like what is represented in the second picture.

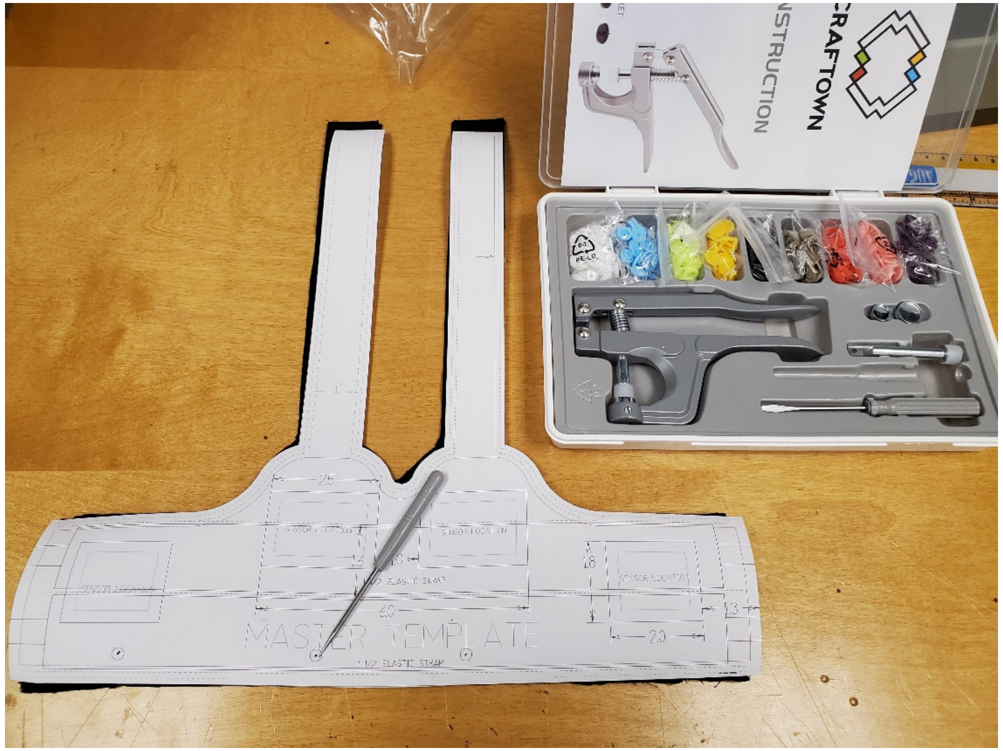

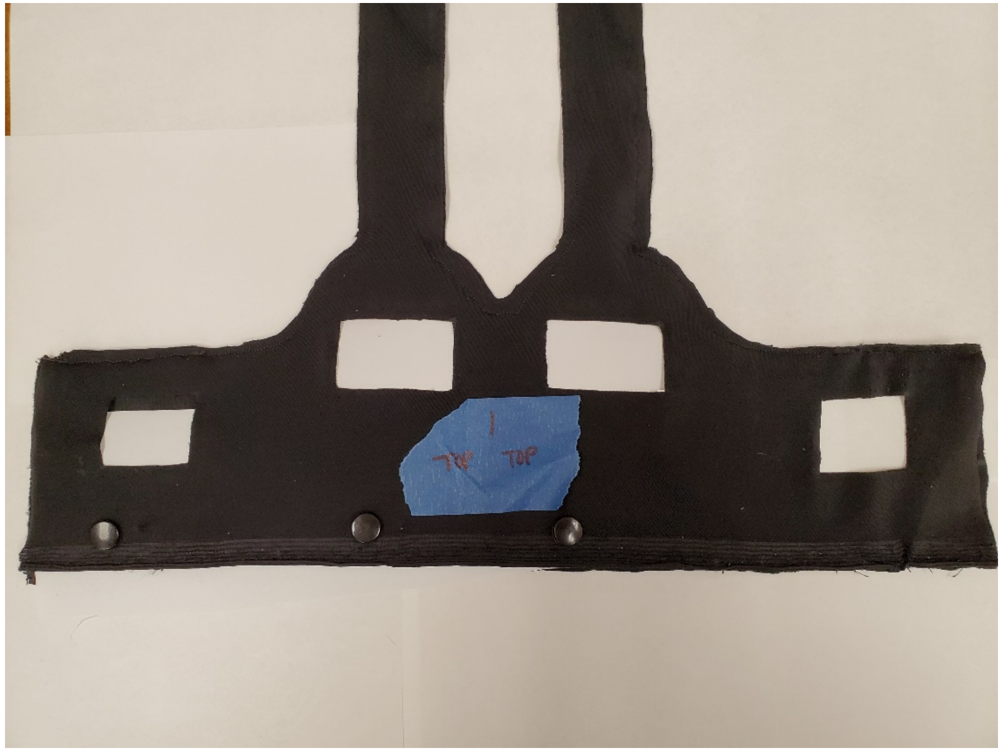

This next step requires the top layer of material to be placed on top of the sewn vest. Be sure to pin the top layer to the rest of the vest lining up the bottoms of the materials to prevent the layers from moving.

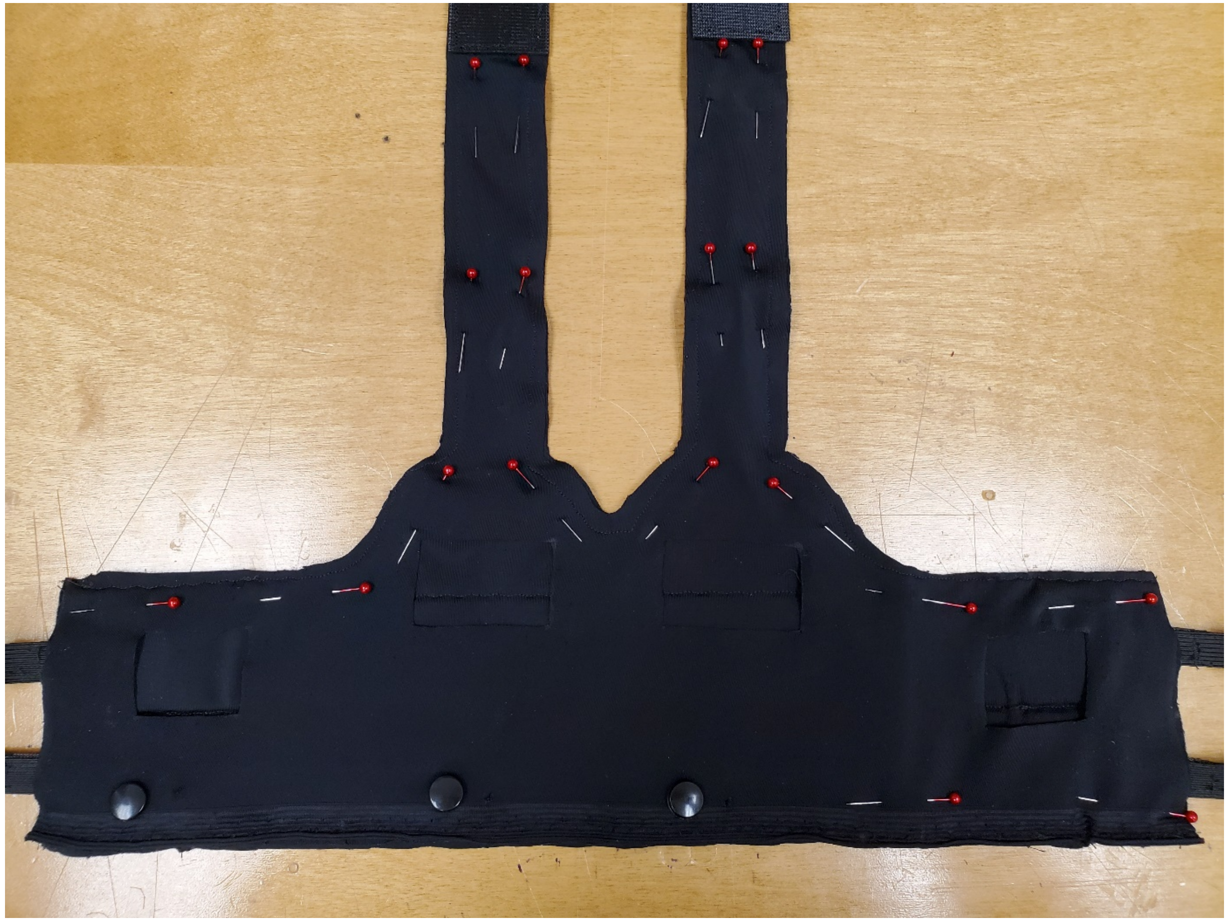

Refer back to the Master Template and continue sewing or gluing the top layer to the other 2 layers following the bright green dotted line pattern on the template. When done, you can remove the label from vest.

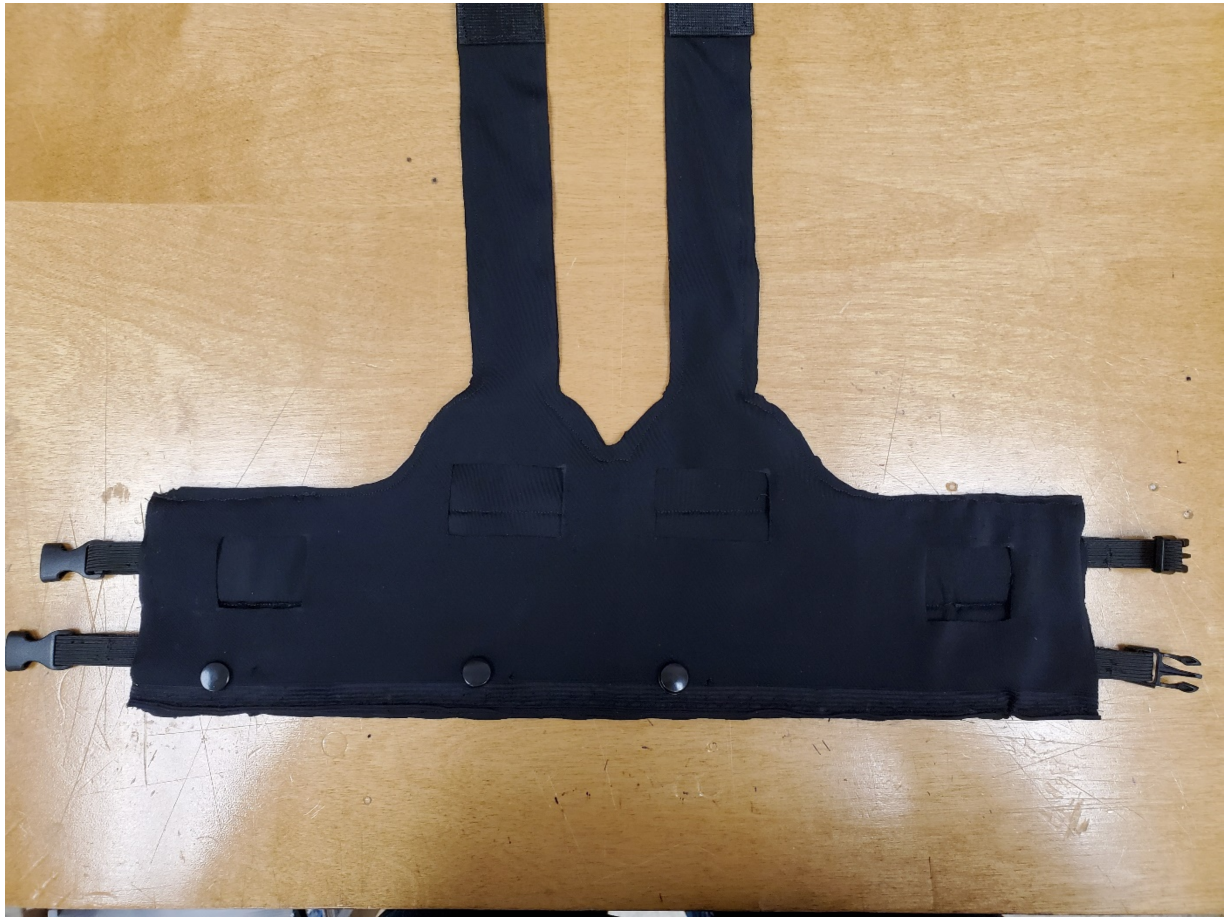

Using the Velcro and Strap Template, measure out the Velcro labeled 1 ½” Velcro Straps.

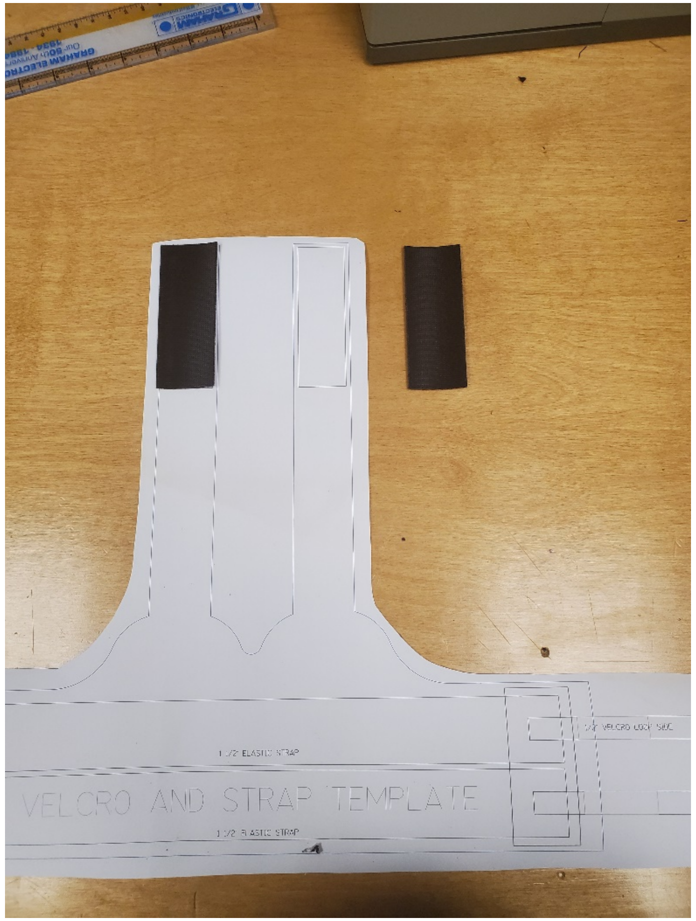

Orient the vest as shown in the picture where you see the cut out rectangles and squares. Sew or glue the 1 ½” Velcro straps in the locations shown on the Velcro and Strap Template (hook side showing).

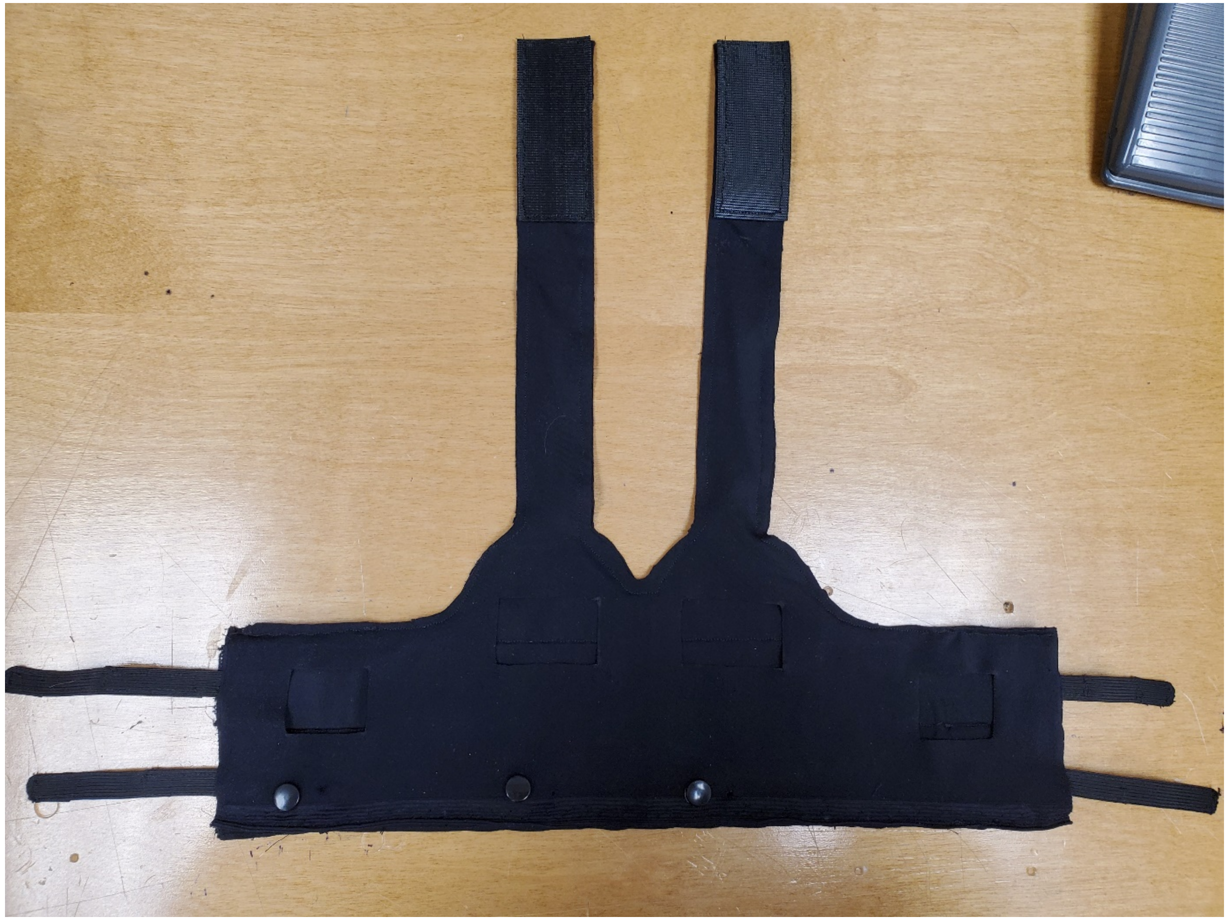

Parachute buckles have a male and female end. For the female end, looping the elastic through that buckle is self-explanatory. For the male end, refer to the picture for proper installation. You will implement an under over method to loop through the parachute buckle.

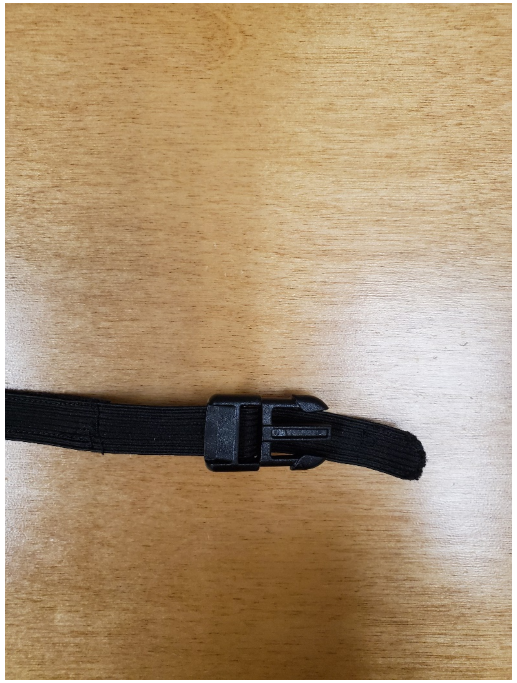

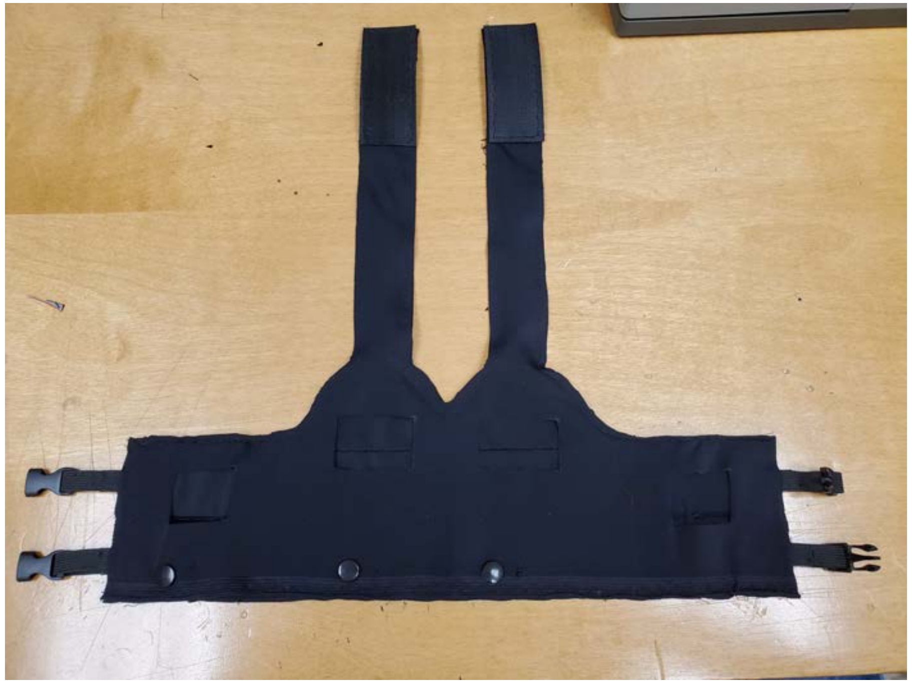

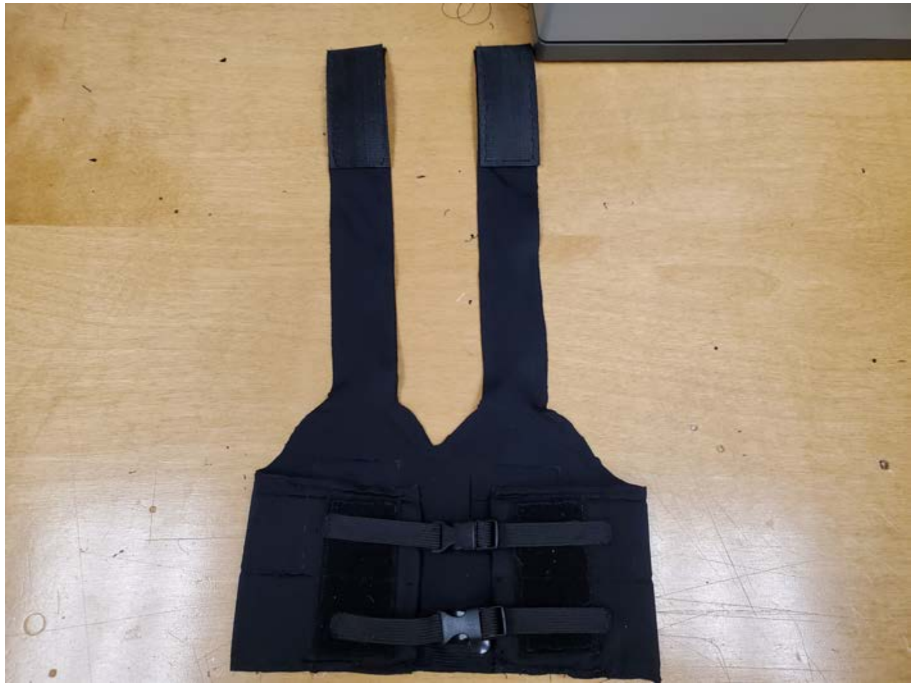

To tidy up the perimeter edges of the vest, an optional method would be using a rotary fabric cutter. Using a straightedge, use the rotary fabric cutter to cut and clean up any uneven edges. This will give an overall better appearance to the vest.

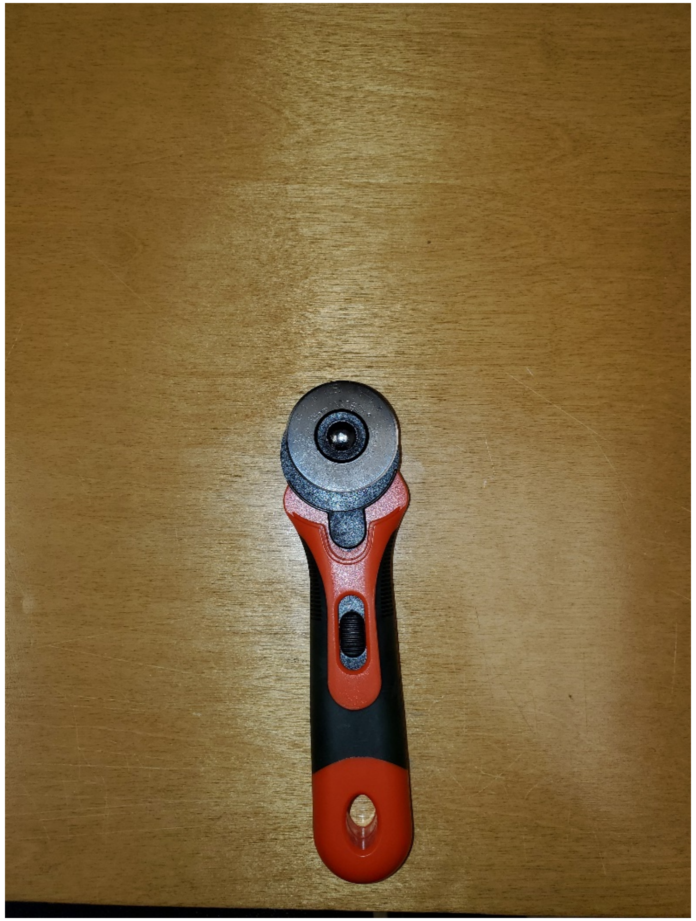

This is what the fabric portion of the vest should look like when completed.

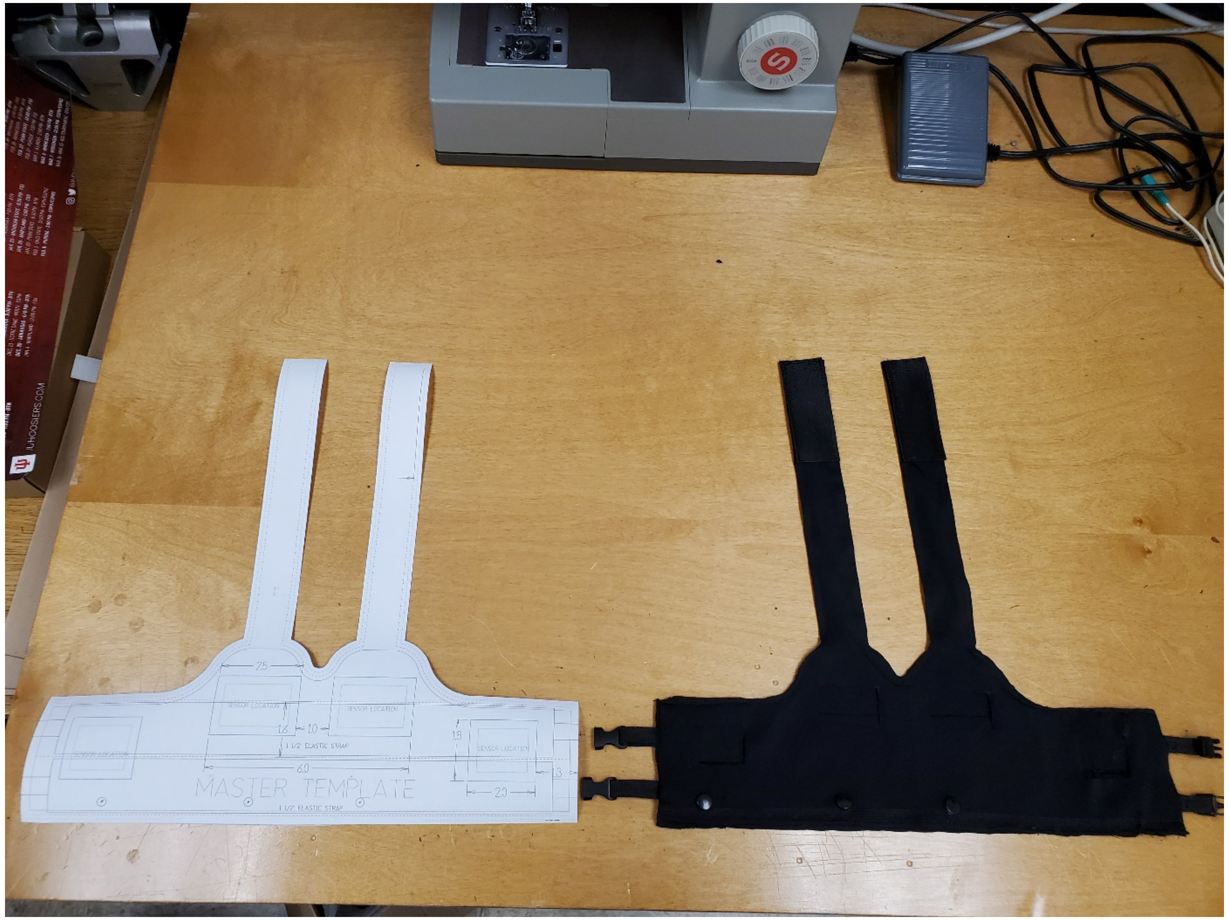

MAKING THE REMOVABLE ELECTRODES

Locate the Master Template. Cut 3 pieces of silicone rubber from rubber sheet approximately 2 ¼” x 1 1/2’ using razor knife or rotary fabric cutter.

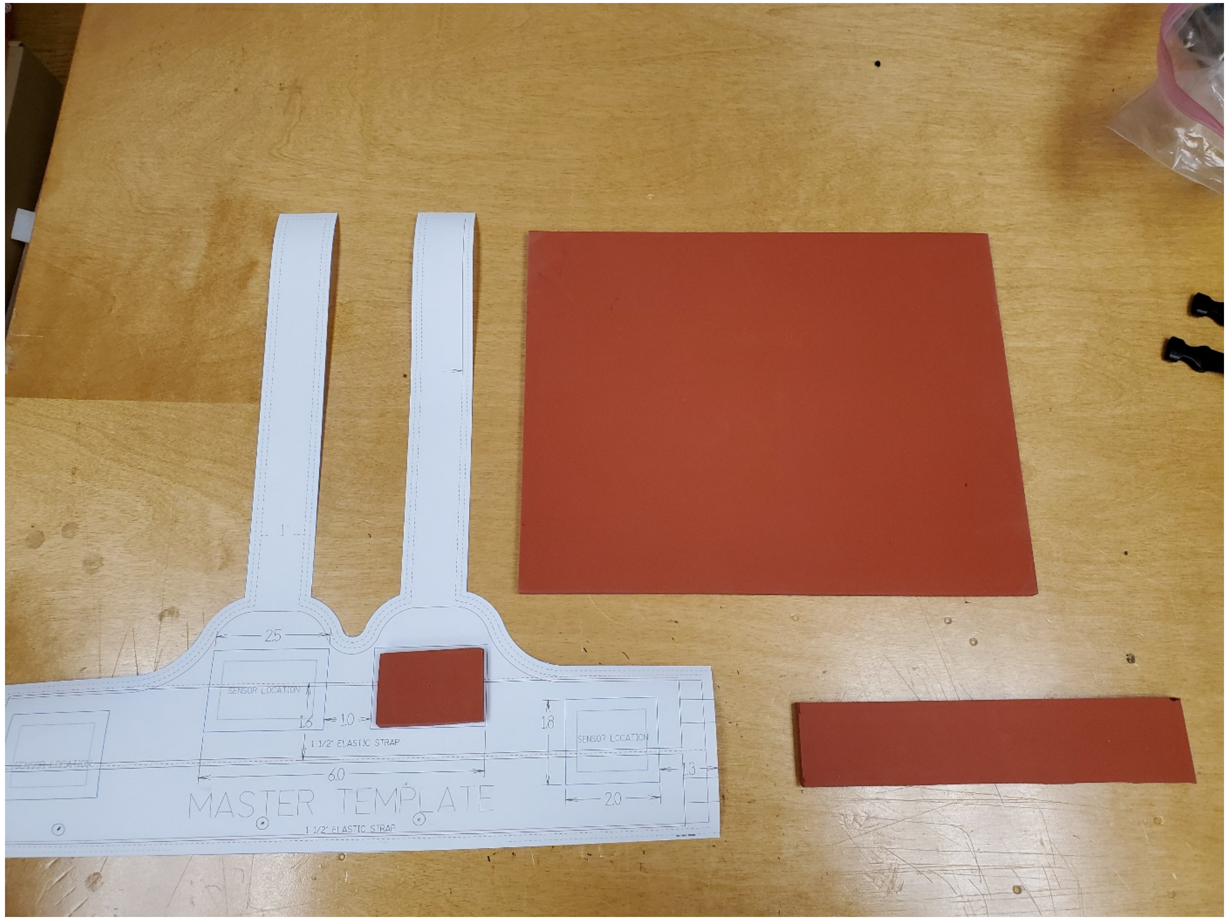

Drill ½" holes in all 3 pieces of silicone rubber almost all the way through but making sure not to go all the way through the material.

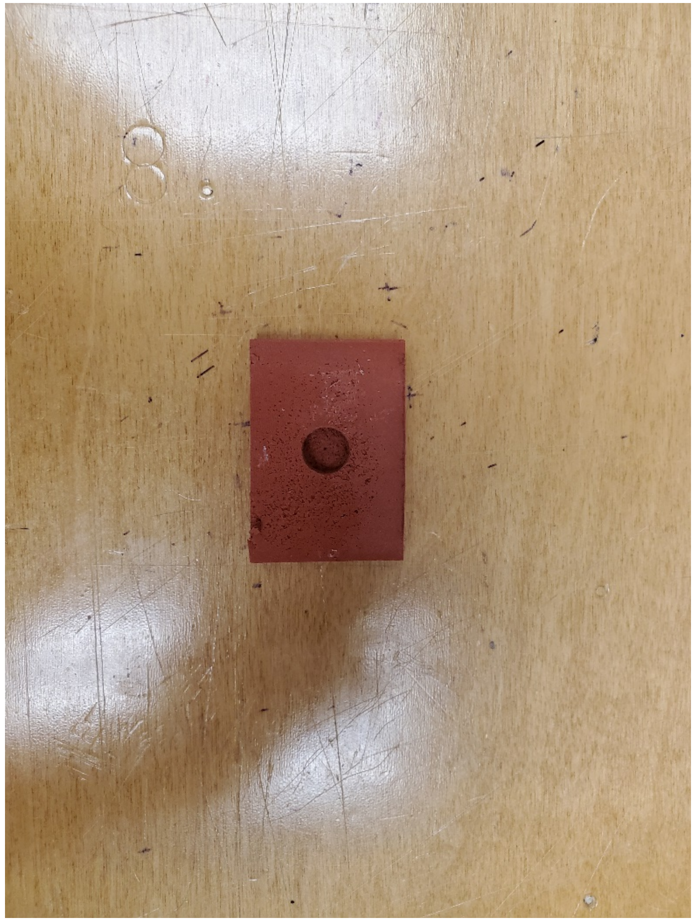

Locate the ferrous fabric and place the silicone rectangle on top of the fabric. The fabric piece should be approximately 4 ½" x 6 ¾". Make marks on the corners of the silicone rubber as shown in the picture.

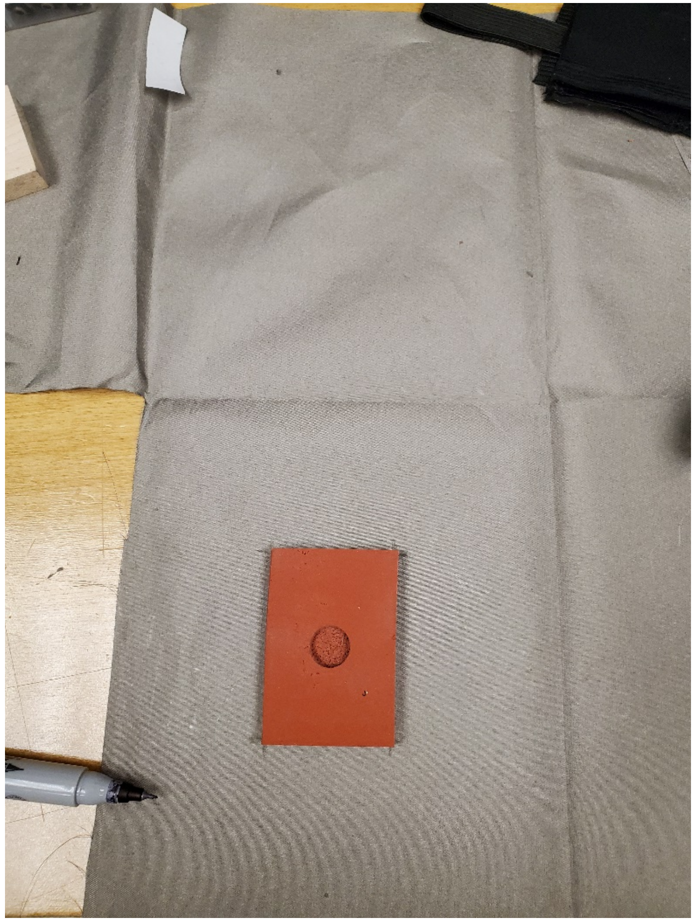

Connect the lines you made in the corners of the silicone rubber and draw lines extending out towards the edge of the fabric as shown in the picture.

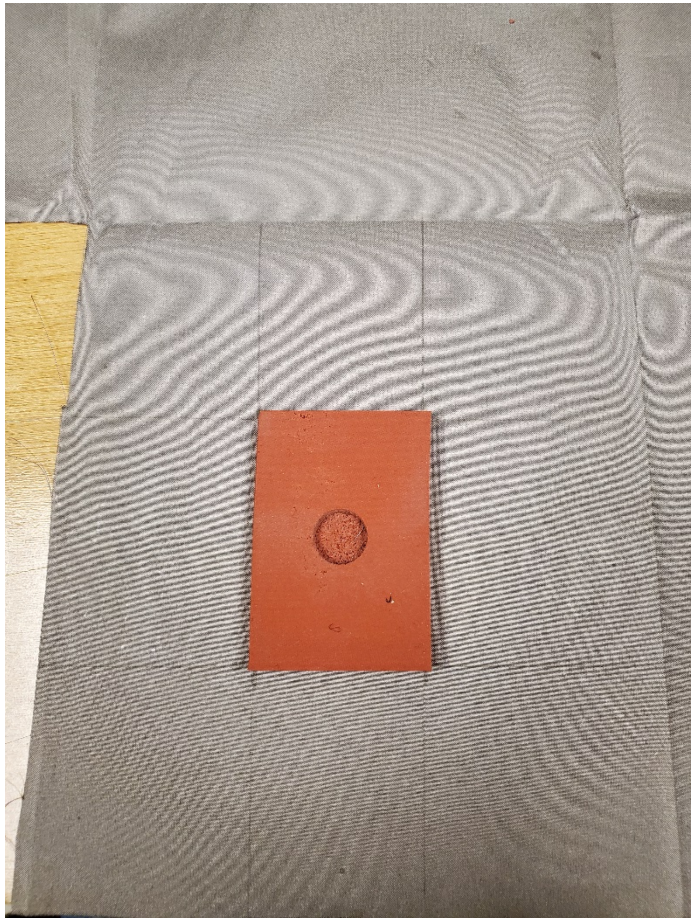

Cut out the 4 corners of the fabric.

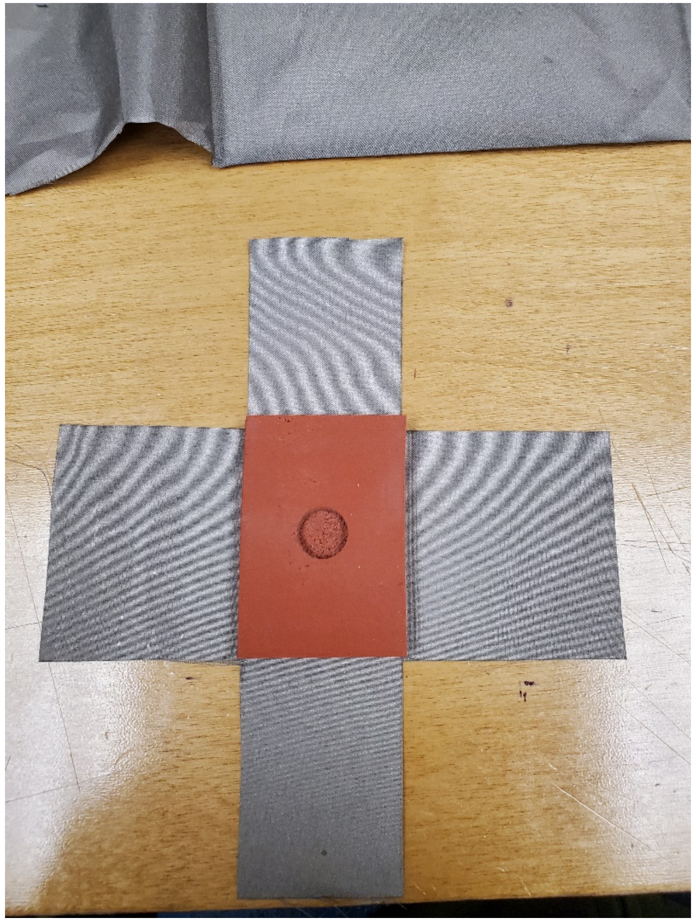

Install the female end of 1 metal snap and punch 3 ½" holes as represented in the picture. Do not use a snap from the Craftown snap kit for this step.

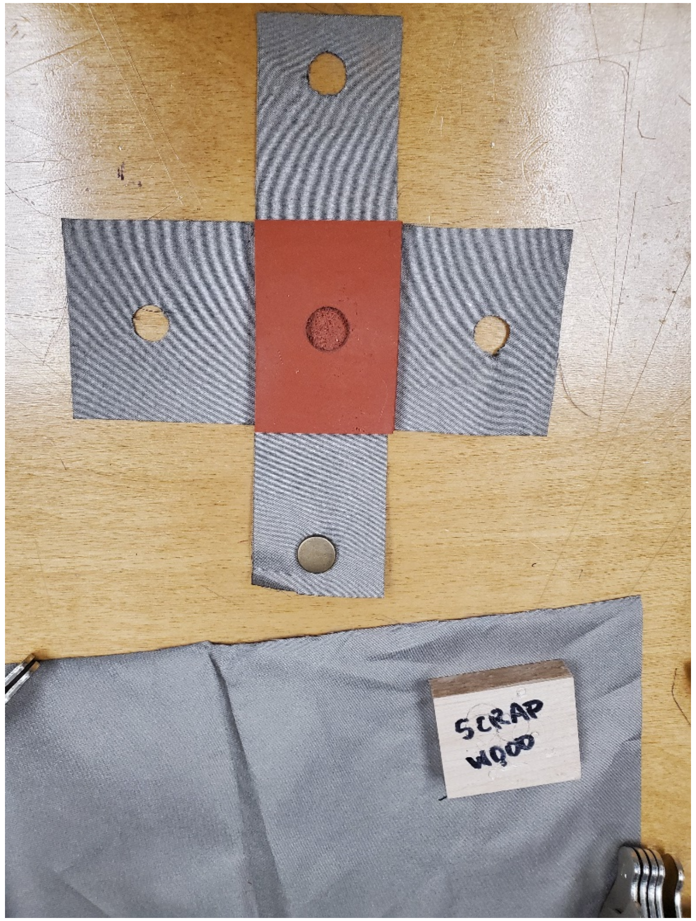

Fold the flaps into the center as shown in the picture. The flap with the snap should be the first one folded into the center with the other layers overlapping them. Use the fabric glue to adhere sides.

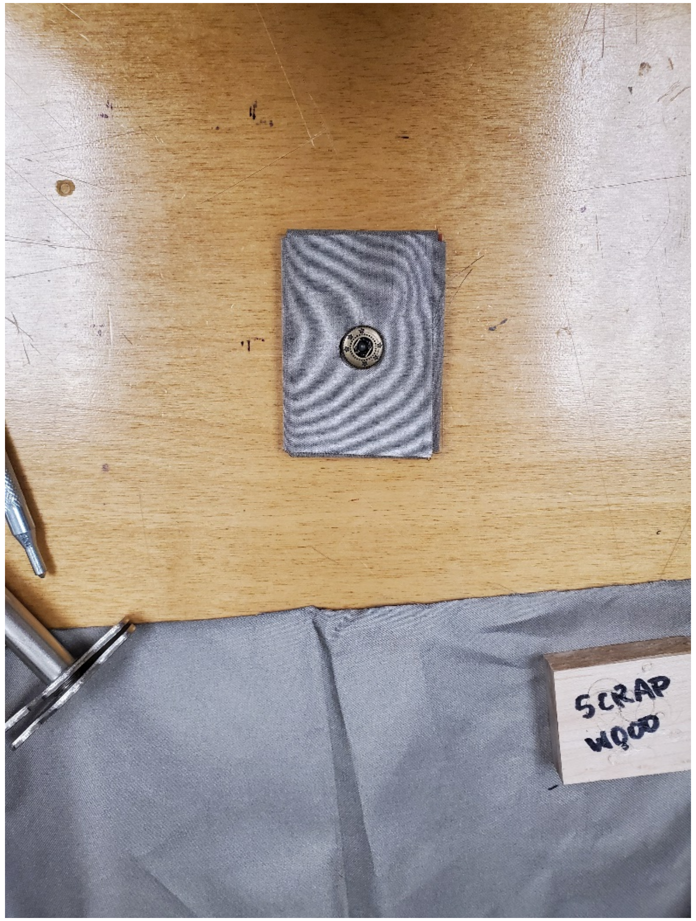

Cut thin strips of low profile Velcro (hook end) and use fabric glue to glue them to the electrode as shown in the picture. You are finished with making the electrodes.

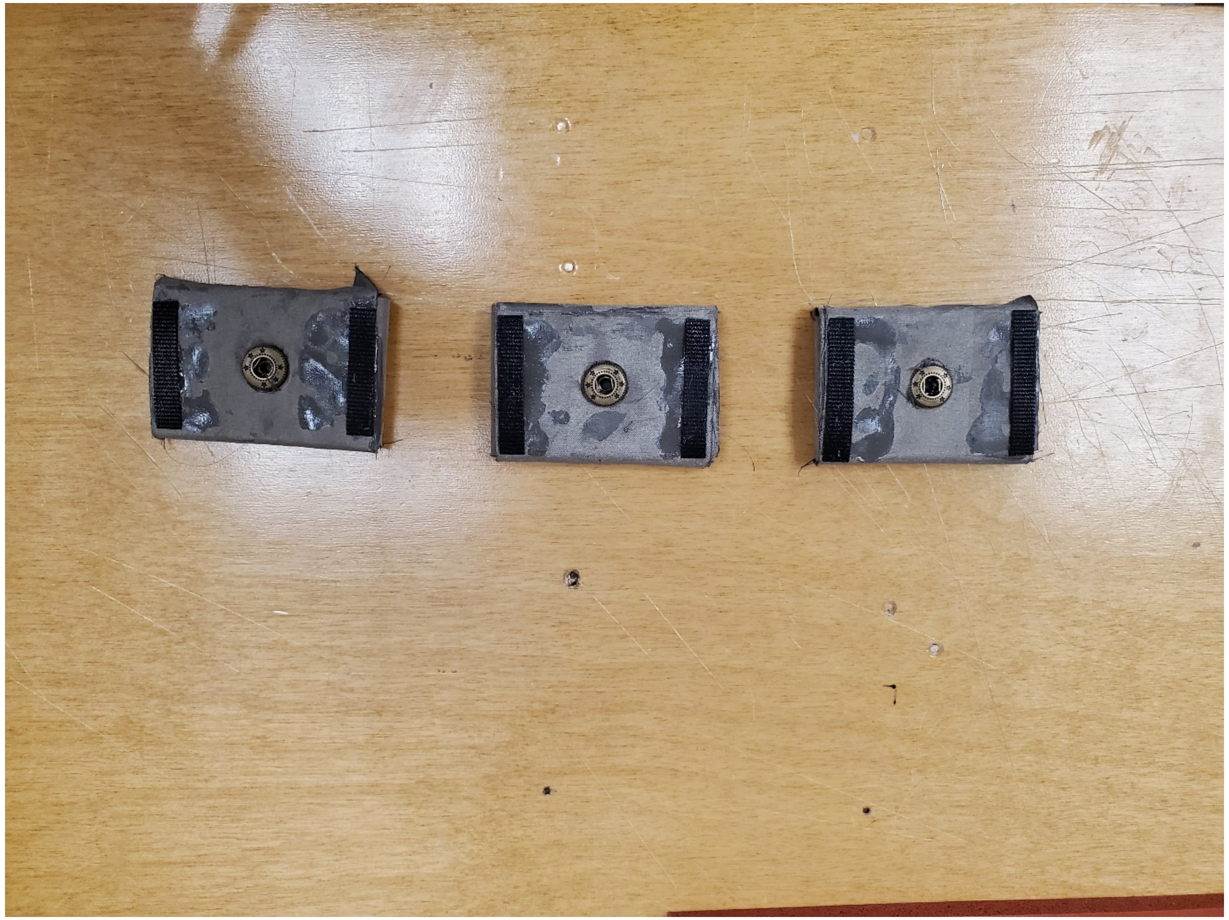

INTEGRATING THE VEST

Gather the fabric portion of the vest, the rubber portion, and the 3 electrodes

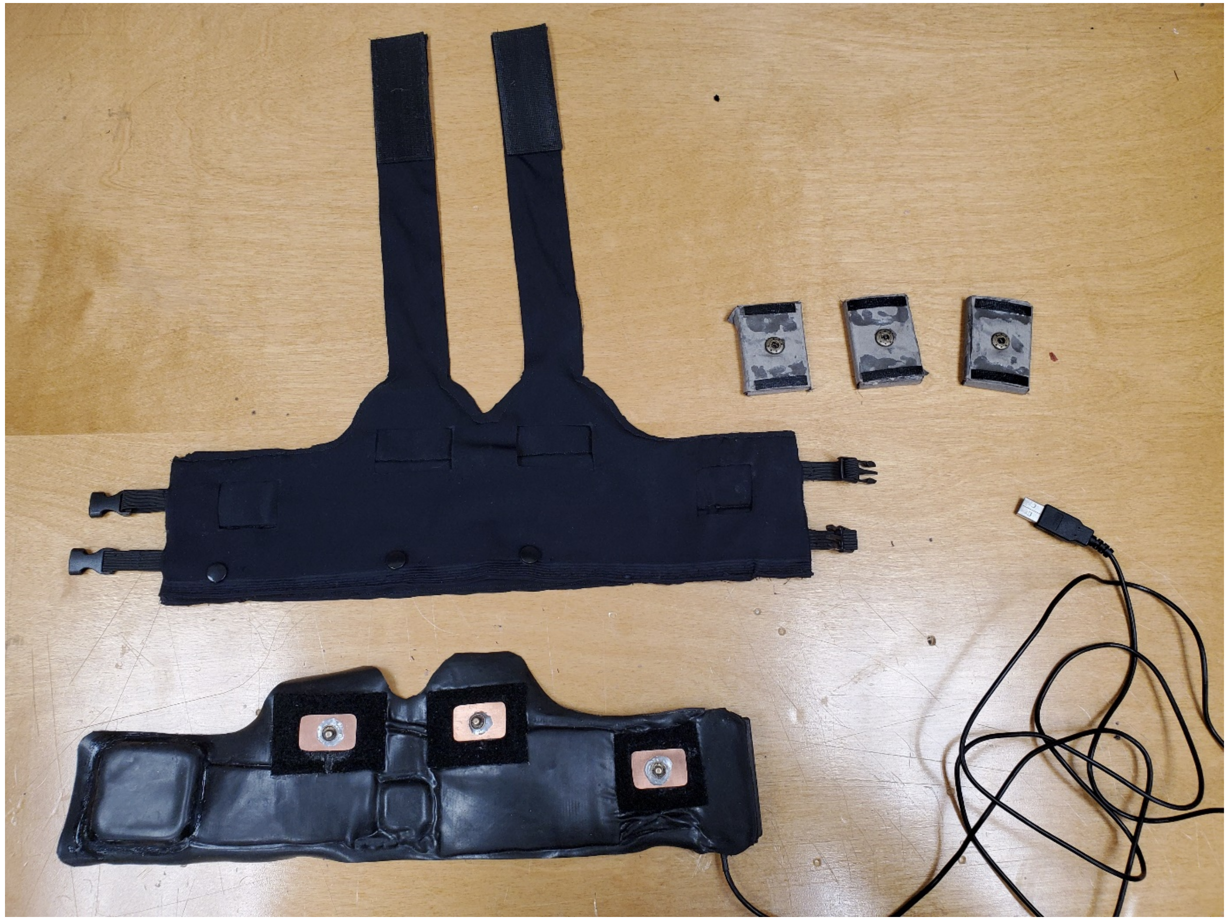

Start by opening the fabric portion of the vest and guiding the USB cable inside the vest to exit out the lower right portion of the vest. Pull the slack out of the cable until most of the cable is through the vest as shown in the pictures.

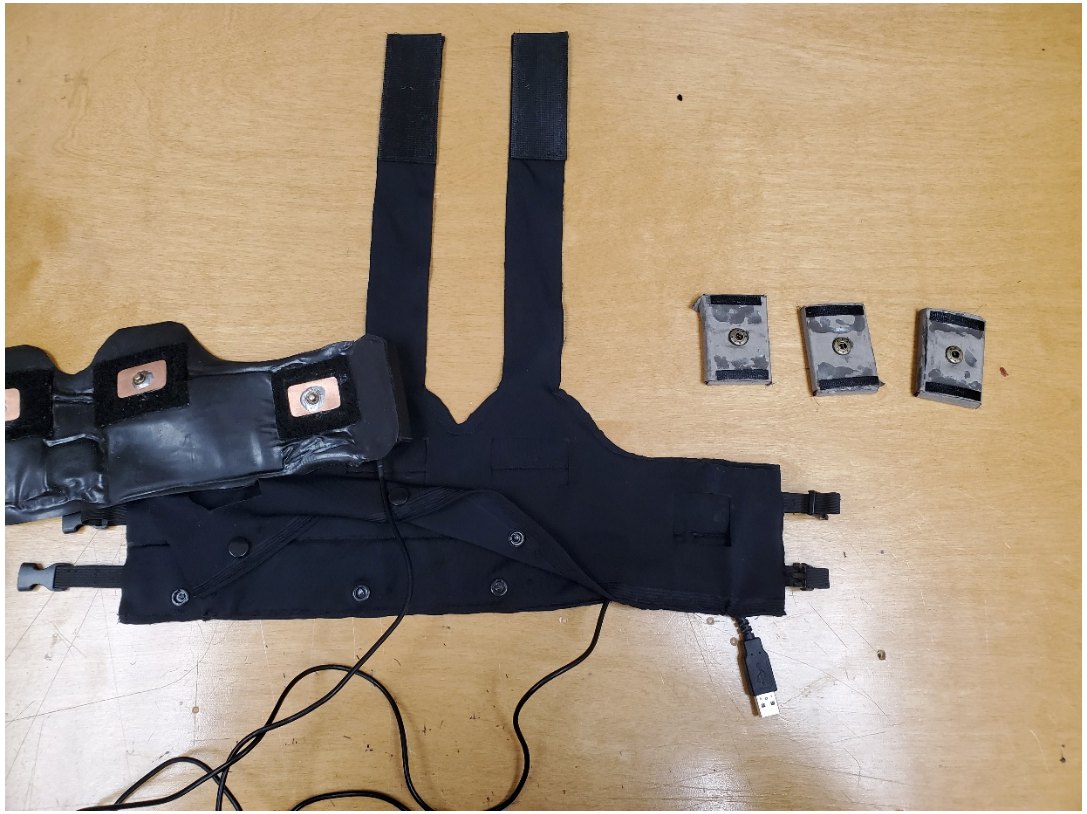

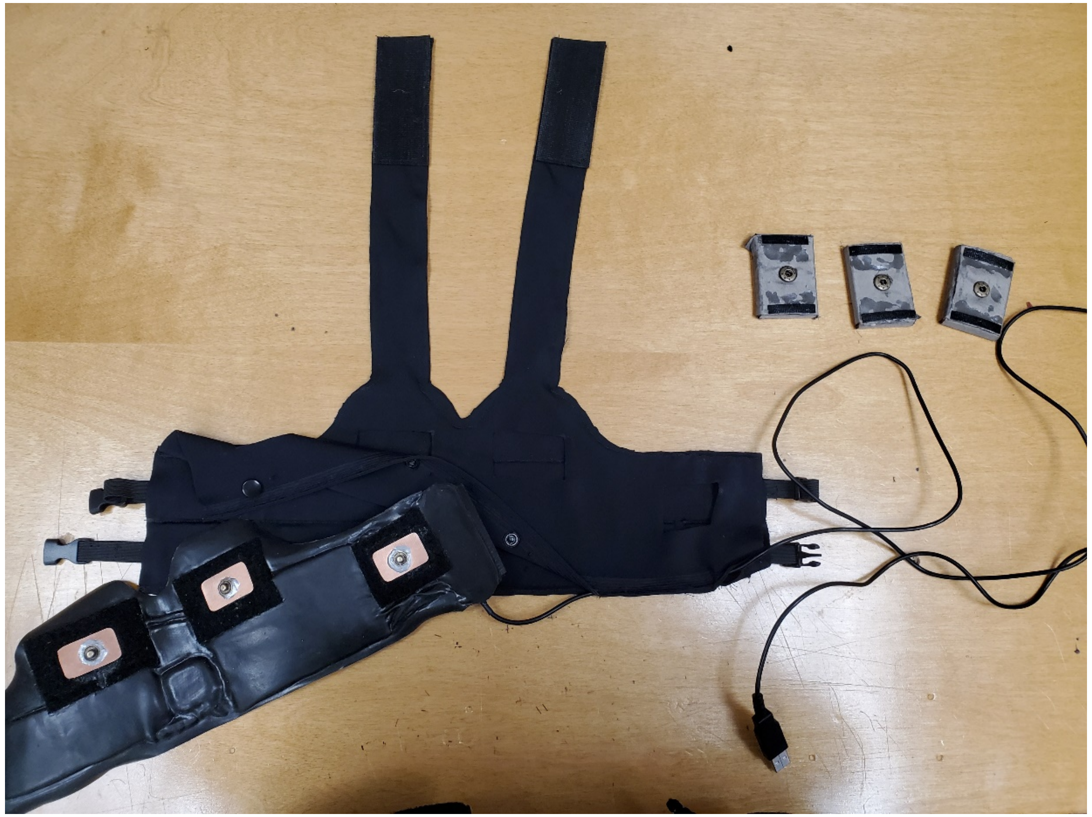

Slide the rest of the rubber into the vest and snap the snaps to close the lower part of the vest up.

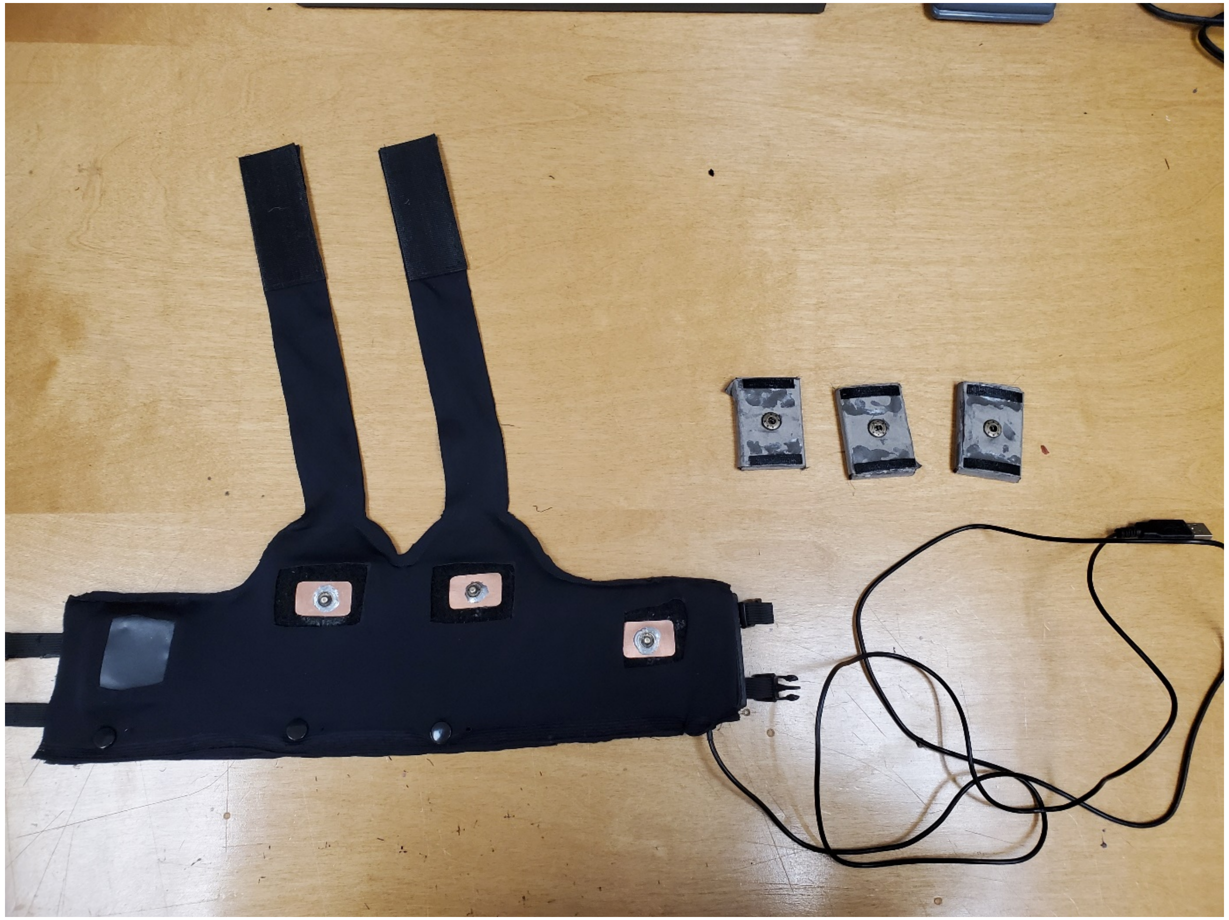

Snap the electrodes into place. Your vest is complete!

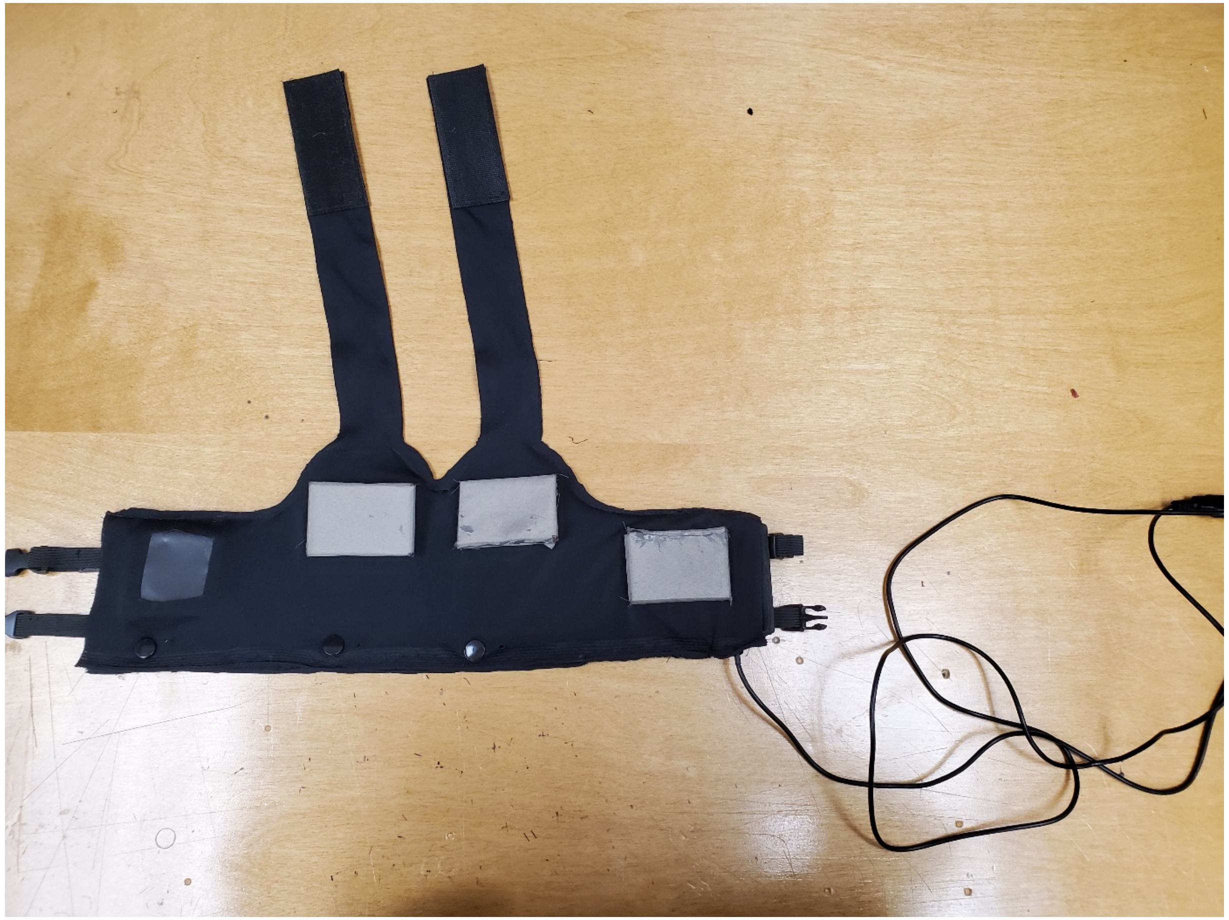

Here are additional pictures of the fully assembled and ready to be used vest.

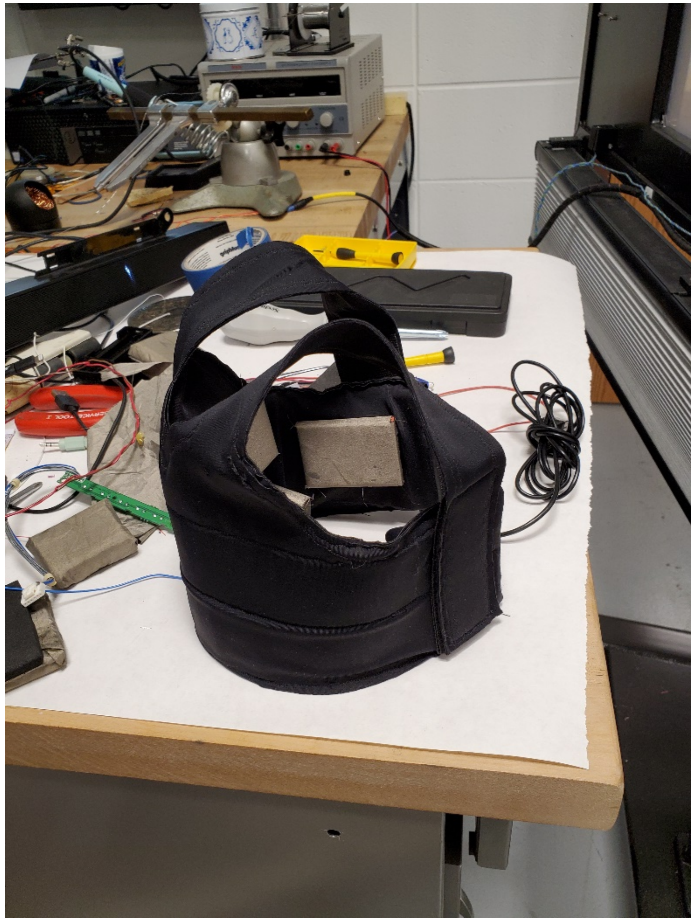

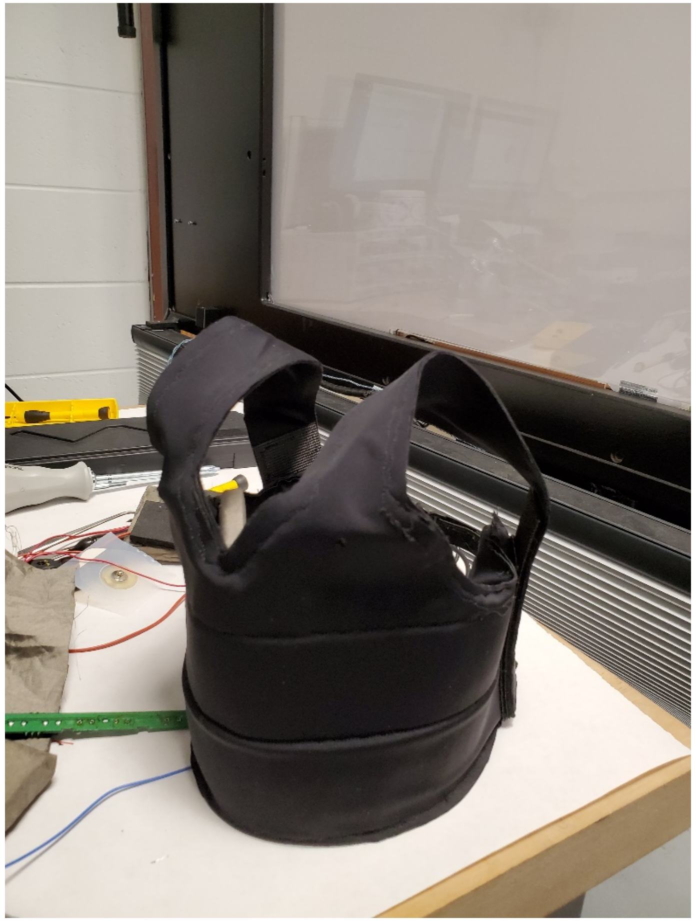

Supplement: Supplementary file 2 — (PDF 22733 kb) [file 13428_2020_1394_MOESM2_ESM.pdf]
